# Genomic Island-Encoded Histidine Kinase and Response Regulator Coordinate Mannose Utilization with Virulence in Enterohemorrhagic Escherichia coli

**DOI:** 10.1128/mbio.03152-22

**Published:** 2023-02-14

**Authors:** Dawei Yang, Yongwu Yang, Pengfei Qiao, Fengwei Jiang, Xinyang Zhang, Zihui Zhao, Tao Cai, Ganwu Li, Wentong Cai

**Affiliations:** a Key Laboratory of Veterinary Public Health of the Ministry of Agriculture, State Key Laboratory of Veterinary Biotechnology, Harbin Veterinary Research Institute, Chinese Academy of Agricultural Sciences, Harbin, China; b Department of General Surgery, the Second Affiliated Hospital of Harbin Medical University, Harbin, China; c Department of Strategic and Integrative Research, Tianjin Institute of Industrial Biotechnology, Chinese Academy of Sciences, Tianjin, China; d Department of Veterinary Diagnostic and Production Animal Medicine, College of Veterinary Medicine, Iowa State University, Ames, Iowa, USA; University of Virginia School of Medicine; Harvard Medical School

**Keywords:** enterohemorrhagic *Escherichia coli*, mannose metabolism, pathogenesis, type III secretion system, virulence regulation

## Abstract

Enterohemorrhagic Escherichia coli (EHEC) is a highly adaptive pathogen and has acquired diverse genetic elements, such as genomic islands and prophages, via horizontal gene transfer to promote fitness *in vivo*. Two-component signaling systems (TCSs) allow bacteria to sense, respond to, and adapt to various environments. This study identified a putative two-component signaling system composed of the histidine kinase EDL5436 (renamed LmvK) and the response regulator EDL5428 (renamed LmvR) in EHEC. *lmvK* and *lmvR* along with *EDL5429* to *EDL5434* (*EDL5429*–*5434*) between them constitute the OI167 genomic island and are highly associated with the EHEC pathotype. *EDL5429*–*5434* encode transporters and metabolic enzymes that contribute to growth on mannose and are directly upregulated by LmvK/LmvR in the presence of mannose, as revealed by quantitative PCR (qPCR) and DNase I footprint assays. Moreover, LmvR directly activates the expression of the type III secretion system in response to mannose and promotes the formation of attaching and effacing lesions on HeLa cells. Using human colonoid and mouse infection models, we show that *lmvK* and *lmvR* contributed greatly to adherence and microcolony (MC) formation *ex vivo* and colonization *in vivo*. Finally, RNA sequencing and chromatin immunoprecipitation coupled with sequencing analyses identified additional direct targets of LmvR, most of which are involved in metabolism. Given that mannose is a mucus-derived sugar that induces virulence and is preferentially used by EHEC during infection, our data revealed a previously unknown mechanism by which EHEC recognizes the host metabolic landscape and regulates virulence expression accordingly. Our findings provide insights into how pathogenic bacteria evolve by acquiring genetic elements horizontally to adapt to host environments.

## INTRODUCTION

Pathogenic bacteria have an extraordinary ability to adapt to host environments, which is achieved through two mechanisms: mutations and horizontal gene transfer (HGT) (1). Genomic islands (GEIs) are mobile genetic elements that are shuttled between bacteria via HGT and enhance the fitness of host bacteria (2). Some GEIs confer virulence determinants and define a certain class of pathogens, such as the locus of enterocyte effacement (LEE) in attaching and effacing (A/E) pathogens, including enteropathogenic Escherichia coli (EPEC), enterohemorrhagic E. coli (EHEC), and Citrobacter rodentium (3). Other GEIs encode regulators that coordinate virulence gene expression to promote host niche recognition (4–6). Therefore, the characterization of GEIs helps to elucidate virulence mechanisms and pathogen evolution, which in turn promotes the understanding of pathogen emergence and the development of antimicrobials.

EHEC is a highly adaptive foodborne bacterial pathogen in humans that requires only 10 to 100 CFUs to establish an infection. Once a host is infected, these bacteria cause bloody diarrhea, hemorrhagic colitis, and fatal hemolytic-uremic syndrome (HUS) in some severe cases (7, 8). EHEC is notoriously responsible for several major outbreaks in history, which resulted in significant morbidity and mortality (9, 10). EHEC produces two major virulence factors, namely, a type III secretion system (T3SS) and Shiga toxin. Shiga toxins are potent AB_5_-type exotoxins that are encoded on EHEC prophages. After entry into the eukaryotic cell cytosol, these toxins inhibit protein synthesis and induce cell death. The absorption of Shiga toxins into the blood leads to its systemic circulation, which may damage the renal microvasculature and result in deadly HUS (11). The T3SS is encoded on a large pathogenicity island (PAI) termed the LEE, and it mediates EHEC colonization by facilitating intimate attachment to host cells, leading to the formation of A/E lesions. A/E lesions are formed due to the effacement of the microvilli and the rearrangement of the underlying host cytoskeleton, which produces a pedestal-like structure underneath the bacterium (12). Notably, EHEC tends to form cluster-like microcolonies (MCs) associated with A/E lesions, and this adherence pattern requires the T3SS (13, 14).

Two-component signaling systems (TCSs), which are typically composed of a membrane-bound histidine kinase (HK) sensor and a cytoplasmic response regulator (RR), have been implicated in regulating bacterial responses to a variety of signals and cues. The recognition of physical or chemical signals by the HK sensor domain triggers the modulation of HK autophosphorylation activity. The phosphoryl group is then transferred to the RR, which is usually a DNA binding protein that acts to alter gene expression (15, 16). A complex variant of the TCS paradigm involves a hybrid HK and its associated phosphorelay. The hybrid HK contains aspartate and/or additional histidines that are engaged in intramolecular phosphotransfer. In some cases, a phosphotransfer protein is required to relay the phosphoryl group from the HK to the RR (17). For example, in response to outer membrane stress, the hybrid HK RcsC phosphorylates itself and then, via its C-terminal RR domain, passes the phosphoryl group to the phosphorelay protein RcsD, and from there, the phosphoryl group transfers to RcsB. Phosphorylated RcsB is then activated for DNA binding and transcriptional regulation (18).

The regulation of the T3SS in EHEC is a highly complex process that involves regulators (19, 20), chemicals (21–23), and mechanical cues such as host cell contact (24). The intricate regulatory network in response to a variety of signals and cues allows EHEC to recognize host niches and trigger T3SS expression at temporally and spatially appropriate times (25). A mucus layer separates the luminal contents from epithelial cells and provides an environment with mixed signals and cues for EHEC, including the presence of oxygen (26–28) and various sugars (29). The TCS FusKR senses the presence of fucose, a sugar contained in mucus, and regulates T3SS expression to maximize fitness *in vivo* (4). Mannose is another monosaccharide that is highly abundant in mucus because N-linked glycosylated glycoproteins contain mannose groups (30). Mannose is a preferred mucin-derived sugar that is used by EHEC during colonization of the mammalian intestine, which is unfavored by nonpathogenic commensal E. coli (29). The expression of *espB*, which is an important effector of the T3SS, is induced in the presence of mannose (27). However, the mechanism by which mannose induces T3SS expression is poorly understood, and how EHEC links mannose utilization with virulence is not known. The present study identified a novel TCS, the HK EDL5436 and the RR EDL5428, which is highly associated with the EHEC pathotype, and this TCS couples with the *EDL5429* to *EDL5434* (*EDL5429*–*5434*) genes to constitute the OI167 genomic island in EHEC strain EDL933. The EDL5428 regulon was probed using RNA sequencing (RNA-seq) and chromatin immunoprecipitation coupled with sequencing (ChIP-seq). We demonstrate that EDL5436 and EDL5428 promote the utilization of mannose via *EDL5429*–*5434* and mediate mannose-induced T3SS expression. Furthermore, we show that *EDL5436* and *EDL5428* contribute substantially to adherence and microcolony formation on human colonoids *ex vivo* and colonization *in vivo*. Based on these findings, we rename *EDL5436* and *EDL5428* as *lmvK* and *lmvR* (lyxose/mannose utilization and virulence kinase and regulator) and *EDL5429*–*5434* as *lmuKAIZYX* (lyxose/mannose utilization kinase, aldolase, isomerase, and transporter subunits Z, Y, and X).

## RESULTS

### The novel HK and RR encoded on the OI167 genomic island of EHEC strain EDL933.

**FIG 1:**
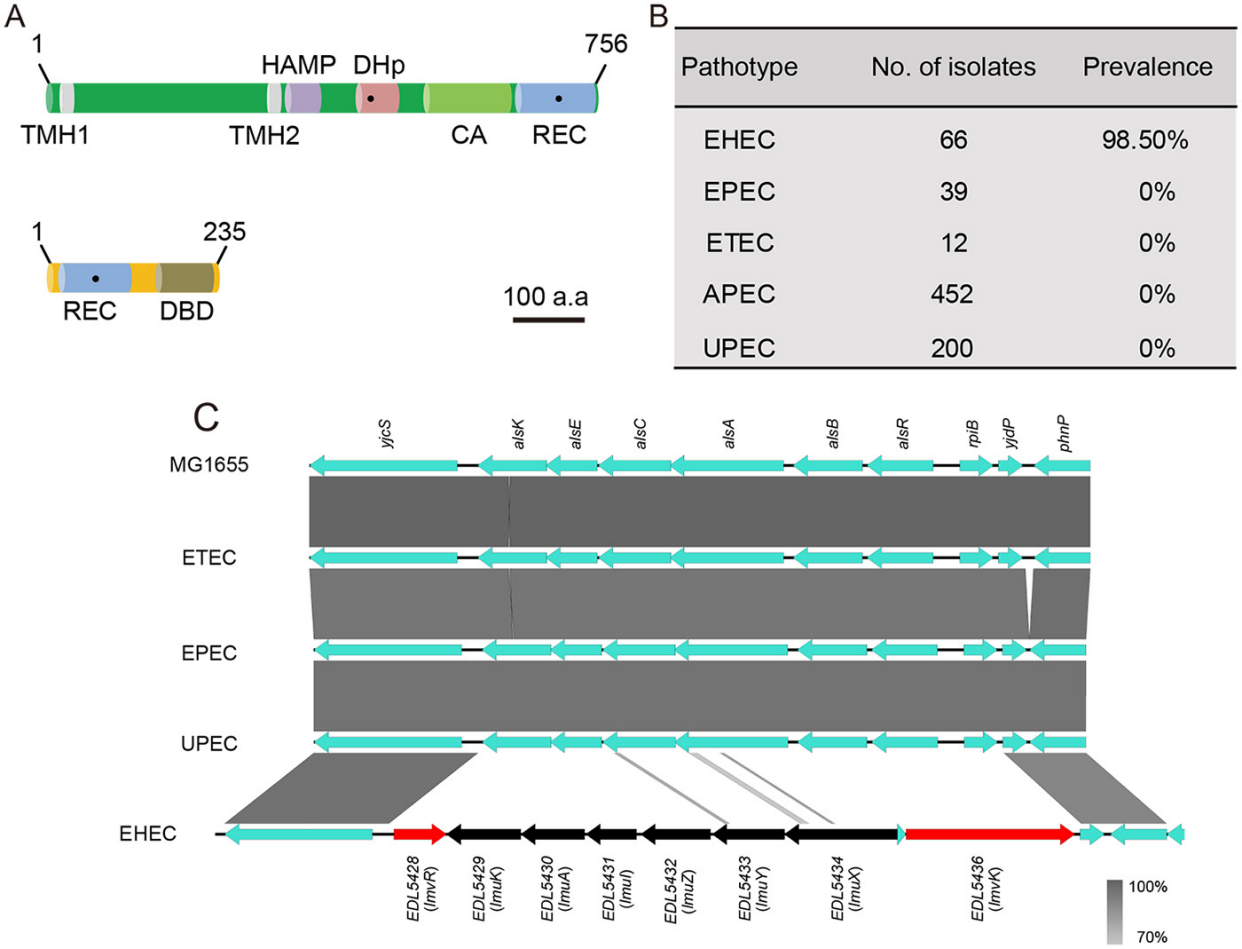
Identification of the novel HK and RR in EHEC. (A) Domain organization of LmvK and LmvR. The numbers indicate the amino acid positions. The domains were predicted from the InterPro database. TMH1, transmembrane helix 1; HAMP, transmitter linker domain often found in HKs, adenylate cyclases, methyltransferases, and phosphodiesterases; DHp, dimerization and histidine phosphorylation domain containing His410 for autophosphorylation; CA, catalytic ATPase domain; REC, receiver domain containing the putative aspartate residue for receiving the phosphoryl group (Asp692 in HK and Asp54 in RR); DBD, helix-turn-helix DNA binding domain. Black dots denote phosphorylation sites. The bar indicates 100 amino acids (a.a). (B) Prevalence of *lmvK* and *lmvR* in various E. coli pathotypes. A duplex PCR method was used to detect the presence of the *lmvR* and *lmvK* genes in a laboratory collection of E. coli isolates. APEC, avian-pathogenic E. coli. (C) Nucleotide sequence alignment of the *lmvK* and *lmvR* loci and their adjacent genes from various E. coli strains. Sequences were derived from MG1655, enterotoxigenic E. coli (ETEC) H10407, EPEC E2348/69, uropathogenic E. coli (UPEC) CFT073, and EHEC EDL933. The *lmvR*–*lmvK* gene cluster constitutes the OI167 genomic island in EHEC. The scale indicates homology. The image was created using Easyfig. Each island gene was given a new name, as shown in parentheses.

Previously, we characterized two TCSs encoded by pathogenic E. coli, highlighting their prominent role in bacterial adaptation to host physiology during infection (5, 31). In a search for novel TCSs in pathogenic E. coli, we identified a previously uncharacterized locus, *lmvK*, encoding a putative HK, and *lmvR*, encoding a putative RR, which were separated by six genes, *lmuKAIZYX*. LmvK is a hybrid type of HK that harbors two transmembrane segments (transmembrane helix 1 [TMH1] and TMH2), a HAMP domain (a transmitter linker domain often found in HKs, adenylate cyclases, methyltransferases, and phosphodiesterases), a dimerization and histidine phosphorylation domain containing a histidine residue (H410), a catalytic ATPase domain, and an RR REC receiver domain containing an aspartate residue (D692) (Fig. 1A). LmvR is an OmpR/PhoB family RR that possesses a CheY-like REC receiver domain containing an aspartate residue (D54) and a regulatory DNA binding domain (DBD) (Fig. 1A).

BLASTN analysis revealed that *lmvK* and *lmvR* were primarily carried by EHEC O157:H7 and rarely occurred in E. coli O55 (considered the ancestor of O157) and non-O157 EHEC (O145) strains. Analysis of gene presence in clinical isolates of our laboratory stock showed that 98.5% of EHEC isolates were positive and all other pathotypes were negative for *lmvK* and *lmvR*, which strongly suggested that *lmvK* and *lmvR* were highly associated with the EHEC pathotype (Fig. 1B). Alignment of the *lmvK* and *lmvR* loci and their adjacent genes from nonpathogenic MG1655, EPEC, enterotoxigenic E. coli (ETEC), and uropathogenic E. coli (UPEC) strains indicated that the *lmvR*–*lmvK* gene cluster constituted the OI167 genomic island inserted between *yjcS* and *yjdP*, in place of the allose utilization loci (Fig. 1C) (32). Taken together, these data identified an EHEC-associated novel hybrid HK and an RR, which, together with *lmuKAIZYX*, formed the OI167 genomic island.

### RNA-seq identified genes differentially expressed in response to *lmvR* overexpression.

**FIG 2:**
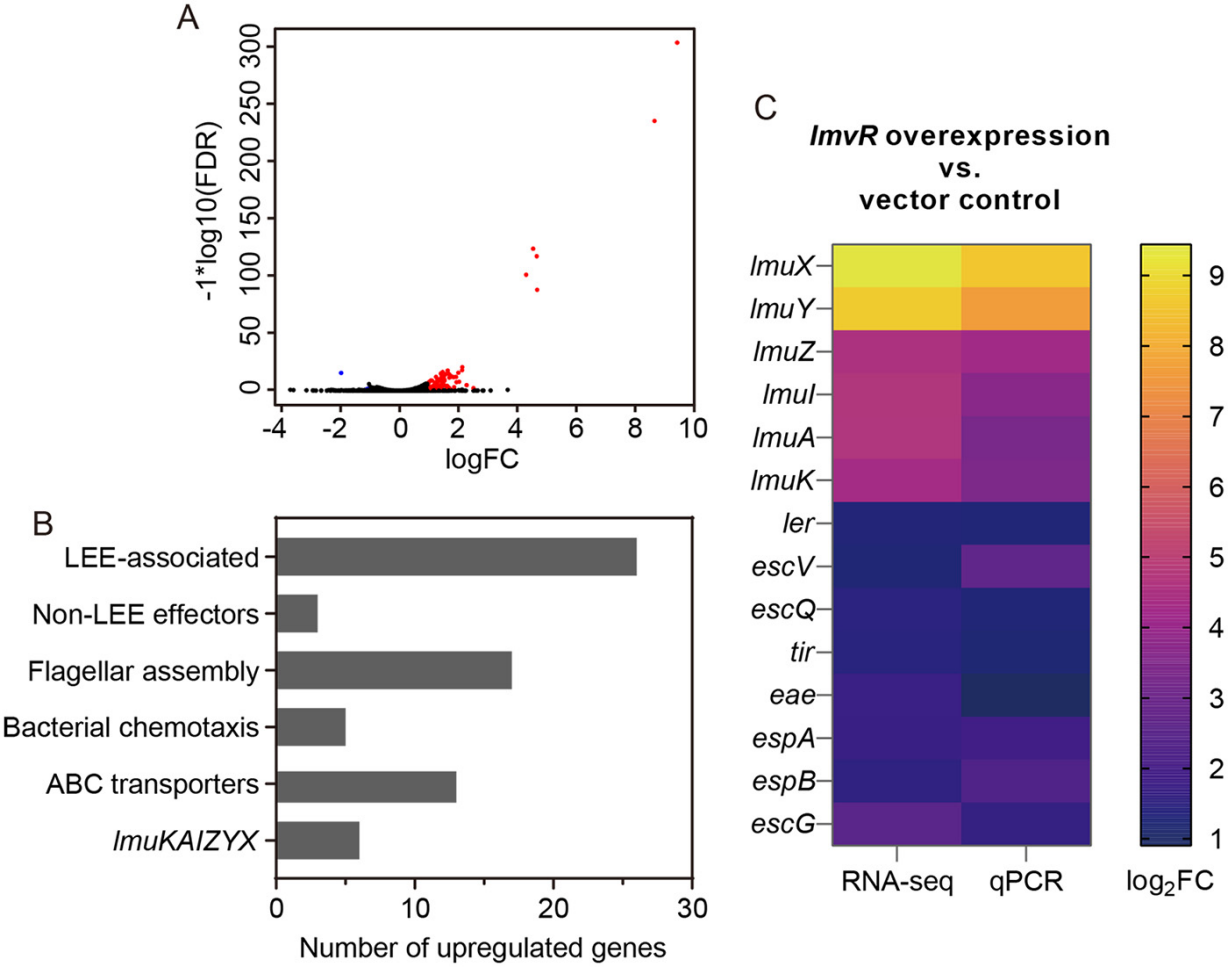
RNA-seq analysis identified genes regulated by the RR LmvR. (A) Volcano plot showing transcriptomic changes due to the overexpression of *lmvR*. A false discovery rate (FDR) of <0.05 and a |log_2_(fold change)| value of ≥1 were used as cutoffs for significance. FC, fold change. Red and blue dots indicate upregulated and downregulated genes in response to the overexpression of *lmvR*, respectively. RNA-seq was performed using 3 biological replicates. (B) Summary of functional categories of differentially expressed genes. Of note, only two genes were downregulated, and they were omitted from this graph. (C) qPCR verification of RNA-seq results. The 2^−ΔΔ^*^CT^* method was employed to determine the fold change, and a |log_2_(fold change)| value of ≥1 was considered significant. Data represent the means from three biological replicates. The corresponding RNA-seq results were also incorporated into this graph.

We next tried to identify genes regulated by LmvR. Response regulators that are overexpressed can activate genes independently of stimuli (33); therefore, we transformed the pLmvR plasmid (*lmvR* overexpression upon isopropyl-β-d-thiogalactopyranoside [IPTG] induction) into the wild type (WT) and compared the transcriptomes of the WT/vector and WT/pLmvR strains using RNA-seq. With a false discovery rate (FDR) of <0.05 and an absolute log_2_ fold change [|log_2_(fold change)|] value of ≥1, 88 genes were differentially expressed in WT/pLmvR compared to WT/vector. Two of these genes were downregulated, and 86 genes were upregulated (Fig. 2A; see also Table S3 in the supplemental material for a full list of differentially expressed genes). Based on biological functions, the majority of the upregulated genes were categorized into four classes: (i) *lmuKAIZYX* carried on OI167; (ii) 26 T3SS-related genes, including 23 LEE and 3 non-LEE genes; (iii) 22 flagellum- and chemotaxis-related genes; and (iv) 6 genes encoding ABC transporters (Fig. 2B). A few genes were selected, and their upregulation was confirmed by quantitative PCR (qPCR) analysis (Fig. 2C).

10.1128/mbio.03152-22.8 TABLE S3 Comparative transcriptomics of EDL933/pLmvR versus wild-type EDL933 (vector) using RNA-seq. Download Table S3, DOCX file, 0.03 MB. Copyright © 2023 Yang et al. 2023 Yang et al. https://creativecommons.org/licenses/by/4.0/ This content is distributed under the terms of the Creative Commons Attribution 4.0 International license.

### *lmvR*, *lmvK*, and *lmuKAIZYX* contribute to the utilization of lyxose and mannose.

**FIG 3:**
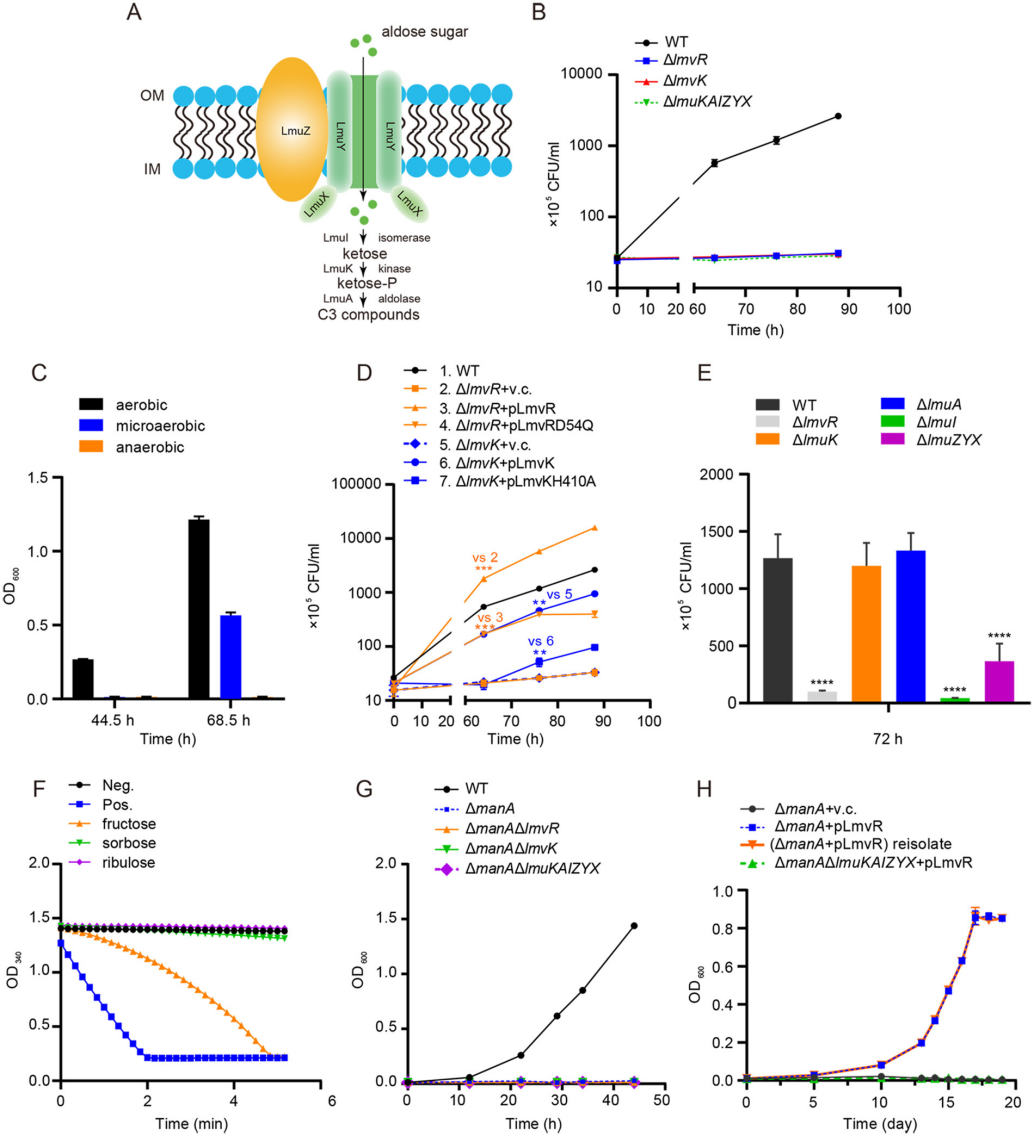
Role of OI167 genes in the utilization of lyxose and mannose. (A) Schematic representation showing the putative functions of *lmuKAIZYX* and the associated hypothetical pathway. In this model, the ABC transporters LmuXYZ import an aldose sugar, which is isomerized to a ketose by LmuI; this ketose is then phosphorylated by LmuK and further broken down by the aldolase LmuA. OM, outer membrane; IM, inner membrane. (B) Growth of wild-type (WT) EDL933 and its isogenic mutants on lyxose. Bacteria were grown in M9 minimal medium containing lyxose (10 mM) as the sole carbon source under microaerobic conditions. An aliquot was taken at different times, and CFUs were determined (*n *= 3). (C) Growth of the WT on lyxose at different oxygen levels. WT EDL933 was grown under aerobic, microaerobic, and anaerobic conditions, and the OD_600_ was measured at the indicated times. (D) Complementation of the mutants with the corresponding wild-type or mutant locus and its effects on growth on lyxose. Comparisons and associated *P* values are indicated on the graph. Statistical analysis was performed by one-way analysis of variance (ANOVA) followed by Tukey’s test. **, *P < *0.01; ***, *P < *0.001 (*n *= 3). v.c., vector control. (E) Roles of the *lmuKAIZYX* genes in growth on lyxose. Statistical analysis was performed by one-way ANOVA followed by Dunnett’s test (multiple comparisons to the WT). ****, *P < *0.0001 (*n *= 3). (F) Carbohydrate kinase activity assay. The consumption of ATP by sugar kinase (LmuK) is represented by the ADP content that was coupled to the oxidation of NADH via reactions catalyzed by pyruvate kinase (PK) and lactate dehydrogenase (LDH). The substrate sugars were present at a final concentration of 5 mM. The depletion of NADH was monitored and is indicated by the reduction in the absorbance at 340 nm. Pos. indicates the positive control (glucose kinase using glucose as the substrate), and Neg. indicates the negative control (no sugar kinase added). Experiments were repeated 3 times. (G) Growth of the WT and its isogenic mutants on mannose. Bacteria were grown in M9 minimal medium containing mannose (10 mM) as the sole carbon source under microaerobic conditions. An aliquot was taken at different times, and the OD_600_ was measured (*n *= 3). (H) Overexpression of *lmvR* promoted *lmuKAIZYX*-mediated growth on mannose. Data represent the means ± standard deviations (SD) (*n *= 3).

*lmuKAIZYX* were among the most differentially expressed genes in response to *lmvR* overexpression. LmuXYZ show ~60% identities to the mannose import ATP binding protein HSERO_RS03640, the mannose ABC transporter permease HSERO_RS03645, and the ABC transporter mannose binding protein HSERO_RS03635, respectively. LmuI is a d-lyxose/d-mannose isomerase (34). LmuA shows 41% identity to the Staphylococcus aureus fructose bisphosphate aldolase (SaFBA), and LmuK shows 23% identity to a carbohydrate kinase family protein, phosphofructokinase (PFK). Comparison of the functions of LmuKAIZYX to the degradation pathways for ribose (35), mannose, and glucose (36) suggested that the *lmuKAIZYX* cluster was involved in the import and catabolism of certain aldose sugars instead of ketoses (see the schematic in Fig. 3A). *lmvK* may encode the sensor of a sugar, and LmvR may activate gene expression for sugar utilization accordingly. To investigate the roles of *lmvR*, *lmvK*, and *lmuKAIZYX* in the utilization of various monosaccharide aldoses, we constructed the corresponding mutants of EHEC strain EDL933 (Δ*lmvR*, Δ*lmvK*, and Δ*lmuKAIZYX*) and assayed the growth of EDL933 (WT) and its derivative strains using these sugars as the sole carbon sources. We observed no differences in growth between these strains under aerobic or anaerobic conditions when using glucose, ribose, arabinose, or xylose as the sole carbon source (data not shown). In contrast, the deletion of *lmvR*, *lmvK*, or *lmuKAIZYX* impaired the growth of EDL933 on lyxose under microaerobic conditions (Fig. 3B). Notably, the growth of EDL933 on lyxose was observed under aerobic and microaerobic conditions but not under anaerobic conditions, suggesting a requirement for oxygen for the utilization of lyxose (Fig. 3C). The introduction of a plasmid carrying a P_tac_-driven wild-type locus into the corresponding mutant markedly increased growth, which confirmed the role of *lmvR* and *lmvK* in growth on lyxose (Fig. 3D). His410 on LmvK and Asp54 on LmvR were predicted to be involved in autophosphorylation and phosphorelay, respectively, and these two sites are critical for HK and RR functionality (Fig. 1A). Compared to the complemented strains with wild-type loci, the Δ*lmvR* and Δ*lmvK* strains expressing the D54Q mutant of *lmvR* and the H410A mutant of *lmvK*, respectively, showed significantly less growth, suggesting that D54 and H410 are critical for LmvR and LmvK functionality, respectively (Fig. 3D). Next, we analyzed the roles of *lmuK*, *lmuA*, *lmuI*, and *lmuZYX* in the promotion of growth on lyxose and found that the deletion of *lmuI* or *lmuZYX* attenuated growth but that *lmuK* and *lmuA* were dispensable (Fig. 3E).

LmuI converts mannose to fructose (34), and biochemical analysis indicated that LmuK had fructose kinase activities (Fig. 3F). Therefore, we suspected that the OI167 island contributed to mannose utilization. Interestingly, the deletion of *lmvR*, *lmvK*, or *lmuKAIZYX* in the wild-type background did not affect EHEC growth on mannose (Fig. S1). Because E. coli carries a conserved gene, *manA*, which encodes a mannose isomerase, we thus hypothesized that the ManA system masked the effects of the island. Therefore, to study the roles of OI167 genes in the Δ*manA* background, we deleted *lmvR*, *lmvK*, and *lmuKAIZYX* in the Δ*manA* strain. Additionally, we also introduced an *lmvR*-overexpressing plasmid, pLmvR, into the Δ*manA* strain to render high expression levels of *lmuKAIZYX* (Fig. 3G and H). We observed that the WT grew normally, but the loss of *manA* resulted in no growth on mannose, suggesting that *manA* is required for the utilization of mannose within 50 h under the conditions tested (Fig. 3G). Notably, the deletion of *lmvR*, *lmvK*, or *lmuKAIZYX* in the *manA* background had no effect on growth on mannose within 50 h (Fig. 3G). However, pLmvR greatly enhanced the growth of the Δ*manA* mutant, while the vector control showed no growth after 10 days. This growth enhancement was dependent on the presence of *lmuKAIZYX* (Fig. 3H). Of note, a reisolate of the Δ*manA*/pLmvR strain exhibited growth kinetics similar to those of the original strain, thereby ruling out the possible contribution of mutations (Fig. 3H). Together, our data demonstrated that *lmvR*, *lmvK*, and *lmuKAIZYX* contributed to the utilization of lyxose and mannose. As lyxose occurs rarely in nature (34), we focus on the role of mannose here.

10.1128/mbio.03152-22.1 FIG S1 Growth of EDL933 and its derivatives on mannose. Bacteria were cultured in M9 minimal medium with mannose (10 mM) as the sole carbon source under static conditions. The optical density at 600 nm was measured at the indicated time points. This experiment was performed with 3 biological replicates. Data represent means ± SD. Download FIG S1, TIF file, 0.2 MB. Copyright © 2023 Yang et al. 2023 Yang et al. https://creativecommons.org/licenses/by/4.0/ This content is distributed under the terms of the Creative Commons Attribution 4.0 International license.

### LmvK and LmvR directly activate *lmuKAIZYX* expression in the presence of mannose.

**FIG 4:**
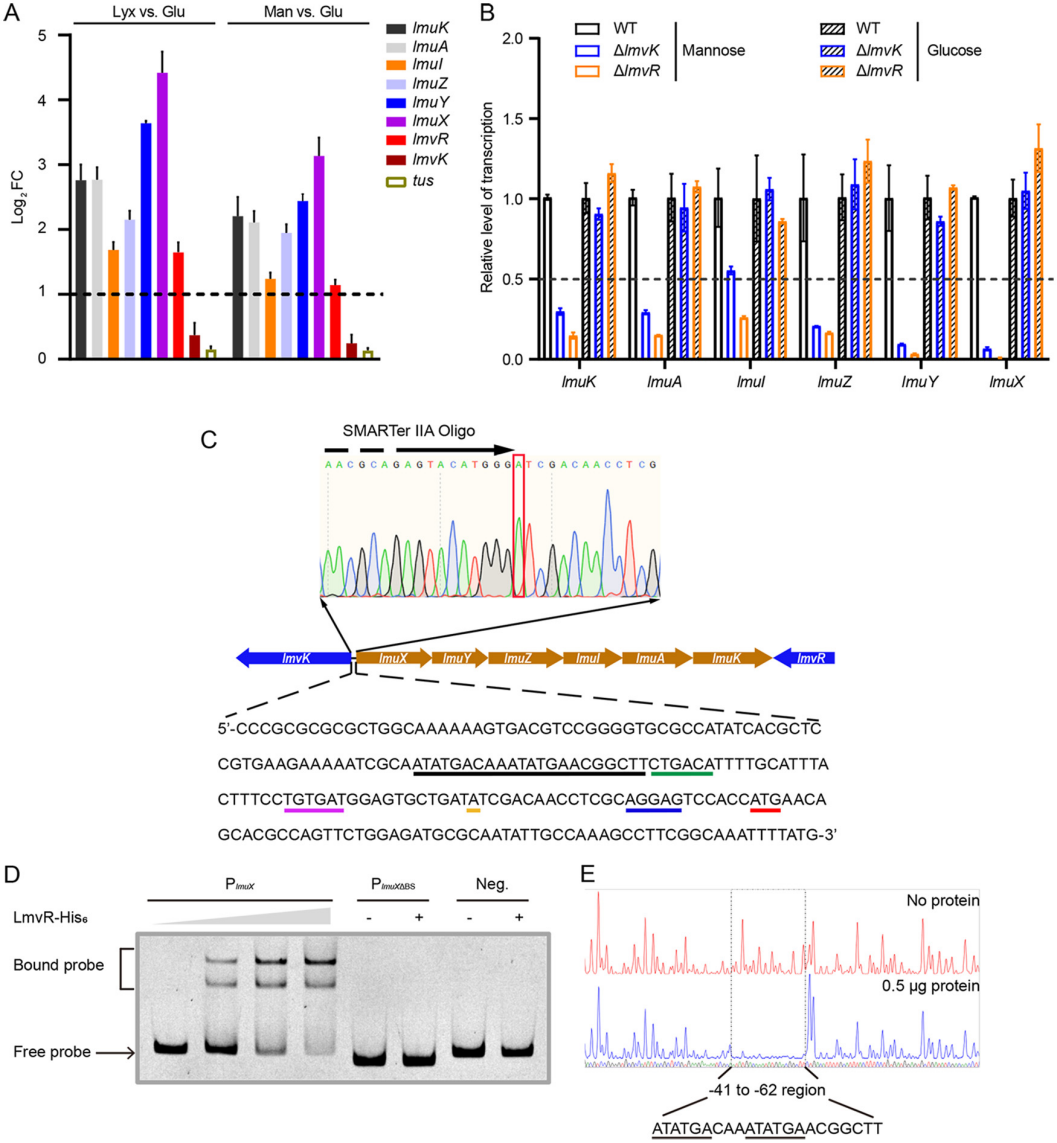
LmvR directly activates *lmuKAIZYX* expression in the presence of lyxose or mannose. (A) *lmuKAIZYX* and *lmvR* expression is induced in the presence of lyxose (Lyx) or mannose (Man). WT EDL933 was grown in M9 medium to early stationary phase using lyxose, mannose, or glucose (Glu) as the sole carbon source under microaerobic conditions. qPCR was used to analyze gene transcription changes. The *rpoB* gene was used as an internal control, and the 2^−ΔΔ^*^CT^* method was employed to determine fold changes. Dashed lines represent a 2-fold change cutoff, and all comparisons with |log_2_(fold change)| values of ≥1 have *P* values of <0.01 by Student’s *t* test. *lmvK* and the negative-control gene *tus* were not significantly induced by lyxose or mannose (*n *= 3). (B) LmvR and LmvK positively regulate *lmuKAIZYX* expression in the presence of mannose. Various EDL933 strains were cultured as described above for panel A, and qPCR was used to analyze relative gene transcription. The *rpoB* gene was used as an internal control, and the gene transcription levels of the WT were set to a value of 1. Dashed lines represent a 2-fold change cutoff, and a fold change of ≥2 was considered significant. Data represent the means ± SD (*n *= 3). (C) Features of the *lmuX* promoter region. The electropherogram indicates the identification of the TSS using 5′ RACE, and SMARTer IIA Oligo stands for the adapter sequence. Colored lines amid the DNA sequence represent the start codon (red), the ribosome binding site (blue), the TSS (orange), the −10 motif (purple), the −35 motif (green), and the LmvR binding site (black). (D) EMSA showing the binding of LmvR to the *lmuX* promoter. A wild-type probe (P*_lmuX_*) or a mutant probe lacking the LmvR binding site (P*_lmuX_*_ΔBS_) was used. Neg. indicates a negative-control probe derived from the promoter of the *bla* (ampicillin resistance [Amp^r^]) gene. The triangle indicates the protein concentration gradient, which was 0 nM, 13.5 nM, 25 nM, and 38.5 nM. “+” denotes the presence of 38.5 nM LmvR protein. (E) DNase I footprint assay. Comparative electropherograms show the protected region (positions −41 to −62) in the *lmuX* promoter following DNase I digestion in the absence and presence of the LmvR protein.

We next examined whether the *lmvK*, *lmvR*, and *lmuKAIZYX* genes were induced in the presence of mannose or lyxose. Wild-type EDL933 was cultured in M9 medium with or without mannose or lyxose, and relative gene transcription was assessed using qPCR. Compared to glucose, both mannose and lyxose markedly stimulated *lmvR* and *lmuKAIZYX* expression (Fig. 4A). To evaluate whether LmvR and LmvK regulated *lmuKAIZYX* expression, the transcription levels of *lmuKAIZYX* were monitored in the WT, Δ*lmvR*, and Δ*lmvK* strains during growth on mannose. As shown in Fig. 4B, the deletion of *lmvR* or *lmvK* significantly reduced *lmuKAIZYX* expression, but the lack of *lmvR* or *lmvK* did not influence *lmuKAIZYX* expression during growth on glucose. These data indicate that LmvR and LmvK positively regulate *lmuKAIZYX* expression in the presence of mannose.

The reverse transcription-PCR (RT-PCR) results showed that *lmuK*, *lmuA*, *lmuI*, *lmuZ*, *lmuY*, and *lmuX* were cotranscribed and formed an operon (Fig. S2). The transcriptional start site (TSS) of the *lmuXYZKAI* operon was determined using 5′ rapid amplification of cDNA ends (RACE) PCR, and the ribosomal binding site (RBS) and the −10 and −35 boxes were deduced accordingly (Fig. 4C). Next, we performed an electrophoretic mobility shift assay (EMSA) with a 240-nucleotide (nt) DNA fragment containing the *lmuX* promoter region as a probe and found that the LmvR protein directly bound to the *lmuX* promoter (Fig. 4D). A negative-control probe was not shifted by the LmvR protein. To map the binding region of LmvR, a DNase I footprint assay was performed. As shown in Fig. 4E, the region from positions −41 to −62 (22 nt in length) was protected from DNase I digestion by the LmvR protein, which indicated the direct binding of LmvR to this region. Furthermore, LmvR did not bind to a DNA probe lacking the 22-nt region (Fig. 4D). The 22-nt binding region contains perfect direct repeats (ATATGA-N_3_-ATATGA), which is consistent with previous findings that the OmpR/PhoB family RRs generally form dimers and bind to direct repeats to regulate gene expression, with each monomer binding to a half-site (37). Taken together, these data demonstrated that LmvK/LmvR directly activated *lmuKAIZYX* expression in the presence of mannose.

10.1128/mbio.03152-22.2 FIG S2 Operon formation by *lmuKAIZYX* examined by RT-PCR. RNA was isolated from EDL933 grown on mannose and reverse transcribed to cDNA. RNA that was not reverse transcribed served as a negative control, and genomic DNA served as a positive control. The positions of primers P1 to P8 are displayed in panel A. For each primer pair, the three lanes represent the positive control, the negative control, and cDNA templates. Download FIG S2, TIF file, 0.9 MB. Copyright © 2023 Yang et al. 2023 Yang et al. https://creativecommons.org/licenses/by/4.0/ This content is distributed under the terms of the Creative Commons Attribution 4.0 International license.

### LmvR directly activates LEE expression in the presence of mannose.

**FIG 5:**
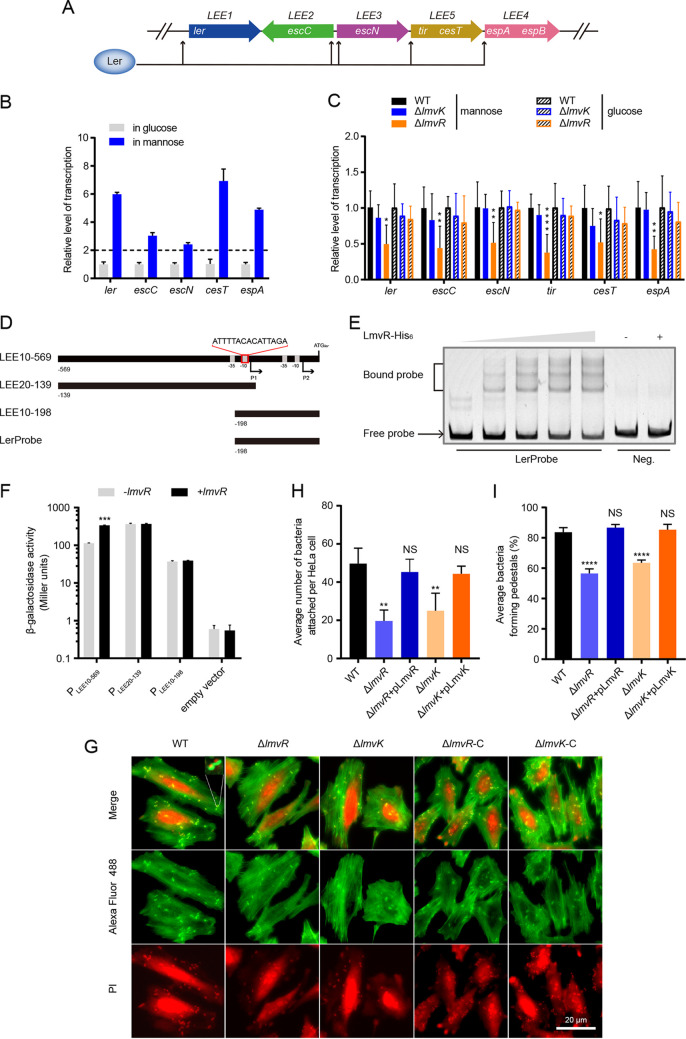
LmvR directly activates LEE expression in the presence of mannose. (A) Genetic organization of representative T3SS genes on the LEE island. Ler is a master regulator that controls the expression of *LEE1* to *LEE5*. (B) qPCR analysis of transcription levels of LEE genes. Wild-type EDL933 was grown in sugar-free DMEM supplemented with mannose or glucose (25 mM) to early stationary phase under microaerobic conditions. The *rpoB* gene was used as an internal control, and gene transcription levels of EDL933 grown in the presence of glucose were set to a value of 1. Dashed lines represent a 2-fold change cutoff, and a fold change of ≥2 was considered significant. Data represent the means ± SD (*n *= 3). (C) Deletion of *lmvR* reduced the transcription of *LEE1* to *LEE5* in the presence of mannose. Various EDL933 strains were cultured as described above for panel B, and qPCR was used to analyze relative gene transcription. The *rpoB* gene was used as an internal control, and the gene transcription levels of the WT were set to a value of 1. Data represent the means ± SD. Statistical significance was analyzed by a two-sided unpaired Mann-Whitney test (*n *= 10). (D) Schematic showing features of the *ler* promoter region and the promoter segments used for the *lacZ* fusion assay. The TSS, −10, and −35 sequences of the two promoters (P1 and P2) are shown. The red box along the sequence above indicates the predicted LmvR binding site. The coordinates were adapted from the ones reported previously (87). (E) EMSA showing the binding of LmvR to the *ler* promoter. The length and position of the Ler probe (LerProbe) containing the putative LmvR binding site are shown in panel D. Neg. indicates a negative-control probe derived from the promoter of *bla*. The triangle represents increasing concentrations of LmvR. (F) Regulation by LmvR requires the presence of both P1 and P2 promoters. β-Galactosidase activity was measured in Miller units during growth in DMEM supplemented with mannose. The empty vector carries promoterless *lacZ*. Data are presented as the means (±SD) from three biological replicates (*n *= 3). ***, *P < *0.001 by Student’s *t* test. (G) FAS analysis. HeLa cells were infected with wild-type EDL933 or its derivative strains in the presence of mannose, and the cells were stained with Alexa Fluor 488-phalloidin to visualize actin (green) and propidium iodide (PI) to visualize bacteria and cell nuclei (red). Under fluorescence microscopy, localized actin aggregation was seen beneath colonized bacteria that formed pedestals and A/E lesions. The inset at the top left shows an amplification of a representative pedestal (magnification, ×63). (H and I) Adherence (H) and pedestal formation (I) were then quantified. Δ*lmvR*-C, complemented Δ*lmvR* strain; Δ*lmvK*-C, complemented Δ*lmvK* strain. (H) Quantification of the average number of bacteria attached per HeLa cell. At least 25 random fields of view were obtained per biological replicate, and data are presented as the means (± standard errors of the means [SEM]) from three biological replicates (*n *= 3). **, *P < *0.01; NS, not significant. (I) Determination of the average percentage of attached EHEC bacteria forming pedestals. At least 25 random fields of view were obtained per biological replicate, and data represent the means (±SEM) from three biological replicates (*n *= 3). ****, *P < *0.0001; NS, not significant.

To probe the details of LEE regulation by LmvR, we tested whether the deletion of *lmvR* affected LEE expression in the absence and presence of mannose. We statically cultured EHEC bacteria in sugar-free Dulbecco’s modified Eagle’s medium (DMEM) supplemented with glucose or mannose. The presence of mannose markedly induced LEE expression compared to the absence of mannose (Fig. 5A and B). As shown in Fig. 5C, the deletion of *lmvR* significantly downregulated LEE virulence genes when mannose was present, but the loss of *lmvR* did not alter LEE expression in the absence of mannose (with 25 mM glucose). In contrast, the lack of *lmvK* did not influence LEE expression in the absence or presence of mannose. Therefore, these results indicate that LmvR activates LEE expression in the presence of mannose.

Because LmvR regulated *ler* and other LEE operons, we suspected that LmvR bound directly to the *ler* promoter to exert regulatory effects. We identified a sequence (ATTTTA-N_3_-ATTAGA) (Fig. 5D) that was similar to that of the LmvR binding site on P*_lmuX_*. LEE1 is driven by two promoters, P1 and P2, and the predicted binding sequence lies 93 bp upstream of the −35 motif of the P2 promoter and overlaps the −10 motif of the P1 promoter of LEE1. Therefore, we generated a probe containing the potential binding site and tested the binding of LmvR to the probe by an EMSA. Figure 5E shows that LmvR apparently shifted the probe as the protein concentration increased, which indicates direct binding. To address whether the P1 or P2 promoter was regulated by LmvR, we made P1-*lacZ*, P2-*lacZ*, and P1P2-*lacZ* reporter fusion constructs. These constructs were transformed into the WT and Δ*lmvR* strains for β-galactosidase activity measurements. Our results showed that regulation by LmvR did not occur with P1 or P2 alone but required the presence of both P1 and P2 promoters (Fig. 5D and F).

The LEE is required for A/E lesion formation. The remodeling of the cell cytoskeleton and the formation of a pedestal-like structure beneath the bacteria are characteristics of A/E lesions (12). Because LmvR regulated LEE expression, we examined whether LmvR played a role in the formation of A/E pedestals by EHEC on host cells by adhesion and fluorescein actin staining (FAS) assays. The deletion of *lmvR* or *lmvK* significantly reduced EHEC adherence to HeLa cells compared to the WT (Fig. 5G and H). The proportion of the remaining adhered Δ*lmvR* or Δ*lmvK* mutant cells formed far fewer pedestals than the WT (Fig. 5G and I). Altogether, these data demonstrated that LmvR directly activated LEE expression and contributed to A/E lesion formation in the presence of mannose.

### Genome-wide analysis of LmvR binding sites using ChIP-seq.

**FIG 6:**
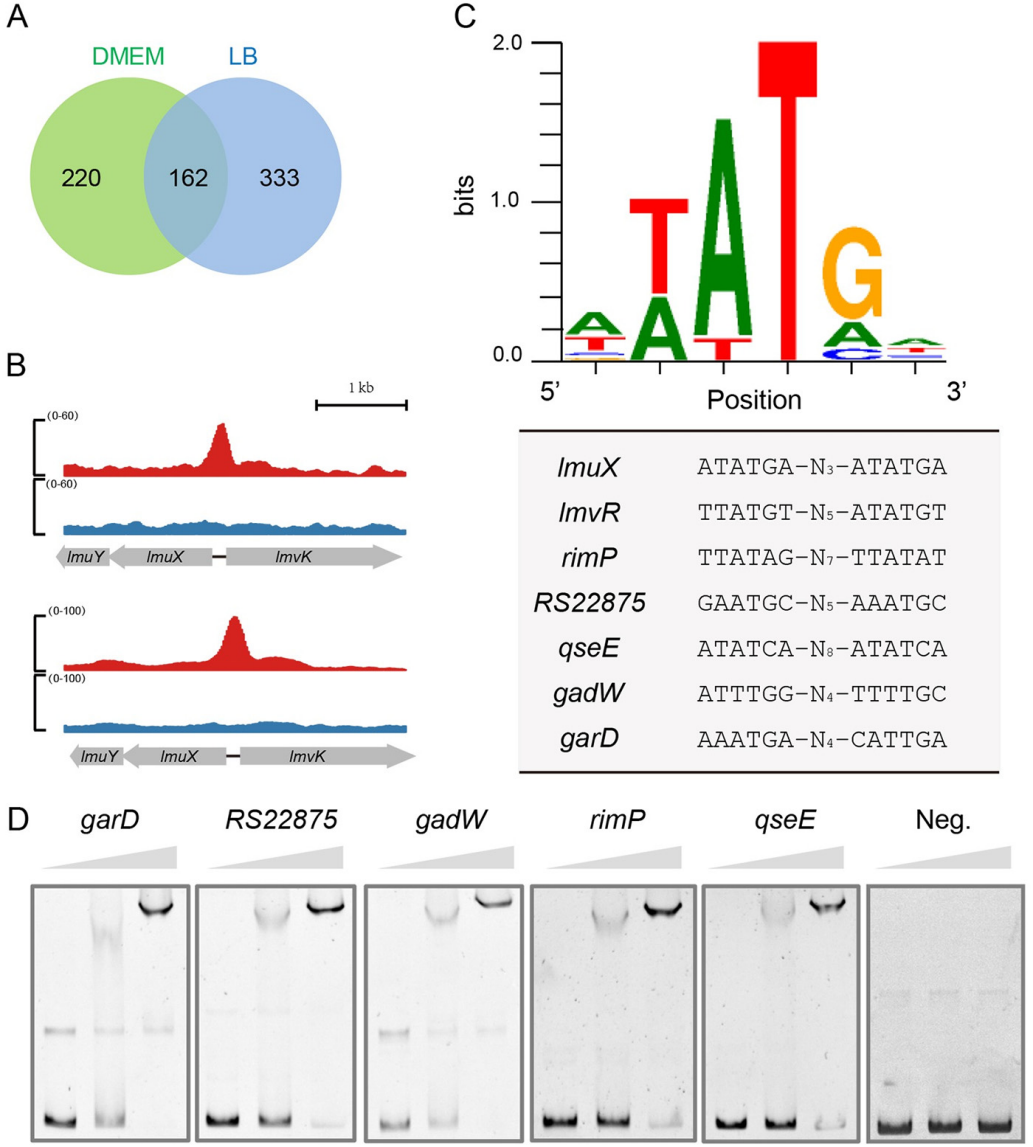
ChIP-seq analysis of LmvR binding sites. (A) Venn diagram showing unique and common enriched peaks during EHEC growth in LB and DMEM (mannose). The Δ*lmvR* mutant carrying pLmvR-2Flag was grown in LB and DMEM (25 mM mannose) to early stationary phase, followed by cross-linking, cell lysis, ChIP enrichment, and DNA sequencing. Using a fold enrichment cutoff value of 1.25, 495 and 382 significantly enriched peaks were identified in LB- and DMEM-grown EHEC, respectively, compared to the input library. (B) Enriched peaks in the *lmuX* promoter region during EHEC growth in LB and DMEM. The *y* axis represents read counts, and the *x* axis represents the position in the genome. Blue indicates reads of the input library, and red indicates reads enriched by ChIP. (C) WebLogo generated from multiple binding sites to show the motif of the LmvR binding half-site. N stands for any nucleotide, and the subscript numbers represent the number of nucleotides between the two half-sites. (D) EMSA validation of LmvR binding to the promoter regions. The triangle indicates the protein concentration gradient, which was 0 μM, 0.5 μM, and 1 μM. Neg. indicates a negative-control probe derived from the promoter of *bla*. Images are representative of results from three independent experiments.

To identify more direct targets of LmvR in addition to *lmuKAIZYX* and the LEE, we performed ChIP-seq analysis using Flag-tagged LmvR (LmvR-2Flag). Wild-type EDL933 carrying P_tac_-driven LmvR-2Flag was cultured in lysogenic broth (LB) and DMEM (mannose). Using a fold enrichment cutoff value of 1.25, we identified 495 and 382 significantly enriched peaks in LB- and DMEM-cultured bacteria, respectively, compared to the input library. The peak characteristics, gene loci, and fold enrichment values are shown in Table S4. A total of 162 genes were identified as having enriched peaks under both growth conditions (Fig. 6A and Table S5). Peaks related to *lmuX* were identified under both conditions (Fig. 6B), which was consistent with the finding that LmvR directly regulated *lmuX*. We also generated a WebLogo consensus for the LmvR half-binding site based on the enriched peaks. The existence of an LmvR binding site in the promoter region of *lmvR* implies that LmvR could regulate its own expression. Indeed, using EMSAs and promoter-*lacZ* fusion assays, we found that LmvR was autoregulated (Fig. S3). Furthermore, several representative binding sites besides LmvR are shown in Fig. 6C, and the binding of LmvR to these loci was verified by EMSAs (Fig. 6D).

10.1128/mbio.03152-22.3 FIG S3 *lmvR* is autoregulated. (A) EMSA showing the binding of LmvR to the P*_lmvR_* promoter. Neg. indicates a negative-control probe derived from the promoter of the *bla* (Amp^r^) gene. The triangle indicates the protein concentration gradient, which was 0 μM, 0.05 μM, 0.1 μM, 0.2 μM, and 0.4 μM. (B) LmvR regulates its own expression. Vc1 is the vector control for pLmvR. The empty vector is the vector control for P*_lmvR_*-*lacZ*, which carries promoterless *lacZ*. **, *P < *0.01; ***, *P < *0.001 (*n *= 3). Download FIG S3, TIF file, 0.8 MB. Copyright © 2023 Yang et al. 2023 Yang et al. https://creativecommons.org/licenses/by/4.0/ This content is distributed under the terms of the Creative Commons Attribution 4.0 International license.

10.1128/mbio.03152-22.9 TABLE S4 ChIP-seq analysis of the RR LmvR during bacterial growth in DMEM and LB. Download Table S4, DOCX file, 0.2 MB. Copyright © 2023 Yang et al. 2023 Yang et al. https://creativecommons.org/licenses/by/4.0/ This content is distributed under the terms of the Creative Commons Attribution 4.0 International license.

10.1128/mbio.03152-22.10 TABLE S5 Information on shared genes with LmvR ChIP peaks during bacterial growth in LB and DMEM. Download Table S5, DOCX file, 0.04 MB. Copyright © 2023 Yang et al. 2023 Yang et al. https://creativecommons.org/licenses/by/4.0/ This content is distributed under the terms of the Creative Commons Attribution 4.0 International license.

A combinatorial analysis of RNA-seq and ChIP-seq data revealed that 14 genes with enriched peaks also showed differential expression in response to LmvR overexpression (Table 1). These 14 genes were implicated in type III secretion, metabolism, and porin production.

**TABLE 1:** Genes with enriched peaks that are differentially expressed in the RNA-seq analysis

Locus tag	Old locus tag	Gene start position	Gene end position	Peak summit position	Fold enrichment	Fold change by RNA-seq^*a*^	Functional annotation
EDL933_RS26910	EDL933_5434	5215205	5213685	5215274	3.08	694	Sugar ABC transporter ATP binding protein LmuX
EDL933_RS02035	EDL933_0405	424286	423204	423542	1.39	2.8	Transcriptional repressor LacI
EDL933_RS04255	EDL933_0859	898620	897661	898688	1.64	2.3	Lipid A deacylase LpxR family protein
EDL933_RS05865	EDL933_1192	1203785	1202697	1203894	1.26	2.4	Porin OmpF
EDL933_RS07100	EDL933_1438	1406552	1408060	1406890	1.79	2.5	Sodium/proline symporter PutP
EDL933_RS12005	EDL933_2451	2293529	2294854	2293532	1.23	2.7	Type III secretion system effector NleA
EDL933_RS16220	EDL933_3310	3061066	3060056	3061296	1.27	2.6	Galactose/methyl galactoside ABC transporter permease MglC
EDL933_RS19525	EDL933_3982	3744261	3745577	3744540	1.8	2.04	l-Fucose:H^+^ symporter permease
EDL933_RS24320	EDL933_4948	4686886	4684082	4686880	1.64	3.2	Intimin type gamma Eae
EDL933_RS21350	EDL933_4348	4102144	4103715	4102105	1.59	2.3	Galactarate dehydratase
EDL933_RS11950	EDL933_2442	2284718	2284128	2284723	1.47	2.1	T3SS effector guanine nucleotide exchange factor EspM1
EDL933_RS26880	EDL933_5428	5208439	5209146	5208778	2.33	NA	Response regulator transcription factor LmvR
EDL933_RS24330	None	4690265	4689654	4690517	2.02	2.7	Type III secretion system LEE effector Map
EDL933_RS24300	None	4681341	4680763	4680977	1.25	3.2	Type III secretion system LEE translocon filament protein EspA
EDL933_RS24375	None	4697204	4697503	4697094	1.59	2.8	Type III secretion system LEE cytoprotective effector EspZ
EDL933_RS24440	EDL933_4971	4706267	4705614	4705123	1.27	2.5	Type III secretion system LEE stator protein EspL
EDL933_RS24460	EDL933_4975	4707781	4707410	4707794	1.29	2.4	Type III secretion system LEE master regulator Ler

a NA, not applicable.

### Contribution of the OI167 island to EHEC virulence.

**FIG 7:**
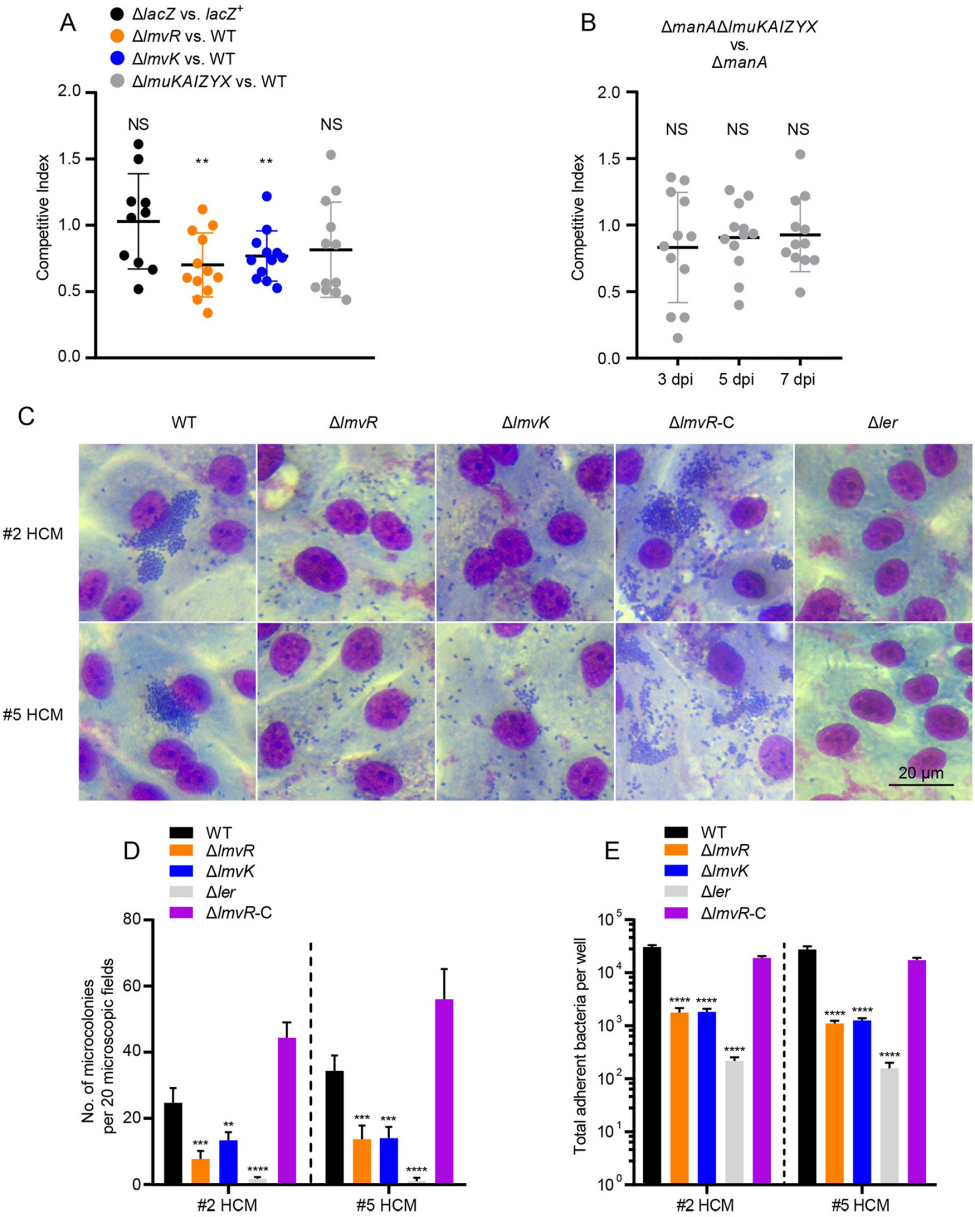
Contribution of OI167 island genes to EHEC virulence. (A) Competition assays in a murine model. Six-week-old female BALB/c mice were orogastrically infected with a 1:1 mixture of the WT and a mutant. At 3 days postinfection, a section of the cecum was harvested, and the WT and mutants were distinguished by the presence or absence of *lacZ* on MacConkey plates. Competitive indices were determined by dividing the ratio of the two strains recovered by the ratio of the same two strains in the inoculum. Horizontal lines represent the medians, and a Wilcoxon signed-rank test was used to determine the significance of the competitive index against a hypothetical median value of 1. The threshold for statistical significance was defined as a *P* value of <0.05. **, *P < *0.01; NS, not significant (*n *= 10 to 12). (B) *lmuKAIZYX* do not contribute to EHEC virulence even in the Δ*manA* background. An *in vivo* competition assay and data analysis were performed as described above for panel A. Samples were collected at three time points, 3, 5, and 7 dpi. (C) Micrographs showing EHEC adherence and microcolony formation on human colonoids. (D and E) HCMs were infected with EHEC strains at an MOI of 10 for 4 h, images were then taken, microcolonies were enumerated (D), and adhered bacteria were counted (E). #2 and #5 HCM indicate biological replicates derived from two human subjects. Shown are images representative of results from 3 biological replicates. (D) Determination of microcolony formation by EDL933 and its derivative strains on human colonoids. Bacterial clusters on HCMs containing >8 bacteria were considered MCs, and the number of MCs was scored as the sum from 20 random microscopic fields. **, *P < *0.01; ***, *P < *0.001; ****, *P < *0.0001. Statistical analysis was performed by one-way ANOVA followed by Dunnett’s test (*n *= 3). (E) Determination of adherence by EDL933 and its derivative strains on human colonoids. To assess the adherence of EHEC, colonoid monolayers were washed 3 times with PBS and then lysed with 1% Triton X-100. Bacteria were enumerated by determining the CFU in each well. ****, *P < *0.0001. Statistical analysis was performed by one-way ANOVA followed by Dunnett’s test (*n *= 3).

Given the importance of mannose utilization and the T3SS for EHEC colonization, we evaluated the contribution of *lmuKAIZYX*, *lmvK*, and *lmvR* to EHEC virulence in a mouse coinfection model and a human colonoid infection model. For the streptomycin-treated mouse model, groups of 6-week-old female BALB/c mice were orogastrically infected with a 1:1 mixture of the WT and a mutant, and the colonization levels in the cecum were represented by the number of bacterial CFU isolated. Our data showed that the loss of *lmvR* or *lmvK* led to a competitive disadvantage at 3 days postinfection (dpi) compared to the WT, which was reflected by the CFU in cecum tissue samples (Fig. 7A) (*P < *0.01). In contrast, the lack of *lmuKAIZYX* did not impact EHEC colonization of the murine large intestine (Fig. 7A). To rule out possible masking effects of ManA, two mutant strains, Δ*manA* and Δ*manA* Δ*lmuKAIZYX*, were used to coinfect the mice in the competition assay. At 3, 5, and 7 dpi, the two strains colonized mice at comparable levels (Fig. 7B), indicating that *lmuKAIZYX* played no role in colonization even in the absence of the ManA system. As expected, no significant difference in colonization was observed for the control group (Δ*lacZ* versus *lacZ*^+^) (Fig. 7A). Thus, these results indicated that *lmvR* and *lmvK*, but not *lmuKAIZYX*, were important for colon colonization by EHEC *in vivo*.

Enteroids are stem cell-derived miniature guts that contain multiple epithelial cell types (enterocytes and goblet cells, etc.), thus representing a unique and advantageous model for studying intestinal pathogen-gut interactions (38). To better understand the role of *lmvK*-*lmvR* in a human-derived system, we developed a colonoid model from human colon biopsy specimens. The colonoids were first generated in three dimensions (3D) (Fig. S4A) and then cultured as human colonoid monolayers (HCMs) in multiwell plates (Fig. S4B). Marker staining demonstrated that different cell types existed, including goblet cells (Fig. S4C). HCMs from two human subjects were infected with EHEC strains, and adherence was visualized and quantitatively determined. As shown in Fig. 7C, the WT adhered to the colonoid and frequently formed microcolony-like clusters; in contrast, the Δ*lmvR* and Δ*lmvK* mutants adhered to the colonoid in a diffuse manner and formed significantly fewer microcolonies (*P < *0.01) (Fig. 7D). Evaluation of the adherence levels showed that the Δ*lmvR* and Δ*lmvK* mutants adhered to the colonoid at much lower levels than the WT (>10-fold decrease; *P < *0.0001) (Fig. 7E). The introduction of the pLmvR plasmid into the Δ*lmvR* mutant complemented these phenotypes. The Δ*ler* mutant was included as a negative control, and the deletion of *ler* abolished EHEC adherence to the colonoid, which is consistent with its well-established role in EHEC adherence (39). Taken together, these results demonstrated that *lmvR* and *lmvK* substantially contributed to EHEC virulence.

10.1128/mbio.03152-22.4 FIG S4 Development of a human colonoid model for EHEC infection. (A) Representative images showing the time course development of human colonoids. (B) Representative image showing a two-dimensional (2D) colonoid monolayer used to assess the adherence of EHEC strains. (C) Identification of different cell lineages in 2D colonoid monolayers based on cell markers. Colonoid monolayer cultures were fixed and stained for specific cell lineages using 4′,6-diamidino-2-phenylindole (DAPI) (nucleus DNA staining) (blue) and antibodies against the following surface markers: Ki-67 for proliferating cells (green), mucin 2 (MUC2) for goblet cells (green), chromogranin A (CHGA) for enteroendocrine cells (green), lysozyme for Paneth cells (green), LGR5 for stem cells (red), and villin for enterocytes (red). Scale bars are shown on the images. Download FIG S4, TIF file, 2.6 MB. Copyright © 2023 Yang et al. 2023 Yang et al. https://creativecommons.org/licenses/by/4.0/ This content is distributed under the terms of the Creative Commons Attribution 4.0 International license.

## DISCUSSION

The gastrointestinal (GI) tract is a complex environment that is influenced by diverse factors such as diet, microbiota, and host genetics (40). EHEC has evolved to integrate different environmental cues to control its virulence program and maximize its fitness (25). These cues include diet-derived arginine (41), microbiota-derived indole (21), and host mucus-derived fucose (4). Human intestinal mucus is composed primarily of mucin, a class of glycoprotein, and it releases a variety of sugars, including mannose, and provides nutrients to commensal members of the microbiota and enteric pathogens. Here, we showed that a novel TCS, LmvK/LmvR, which was encoded on the OI167 genomic island and highly associated with the EHEC pathotype, mediated mannose utilization and mannose-induced T3SS expression. The T3SS is one of the most important virulence traits of EHEC, which is involved in intimate adherence and microcolony formation (13). We found that *lmvK* and *lmvR* substantially contributed to adherence and microcolony formation on human colonoids *ex vivo* and colonization *in vivo*. Our study thus details a previously unknown layer of exquisite virulence regulation in EHEC and highlights the crucial role of HGT in shaping EHEC as a successful enteric pathogen.

**FIG 8:**
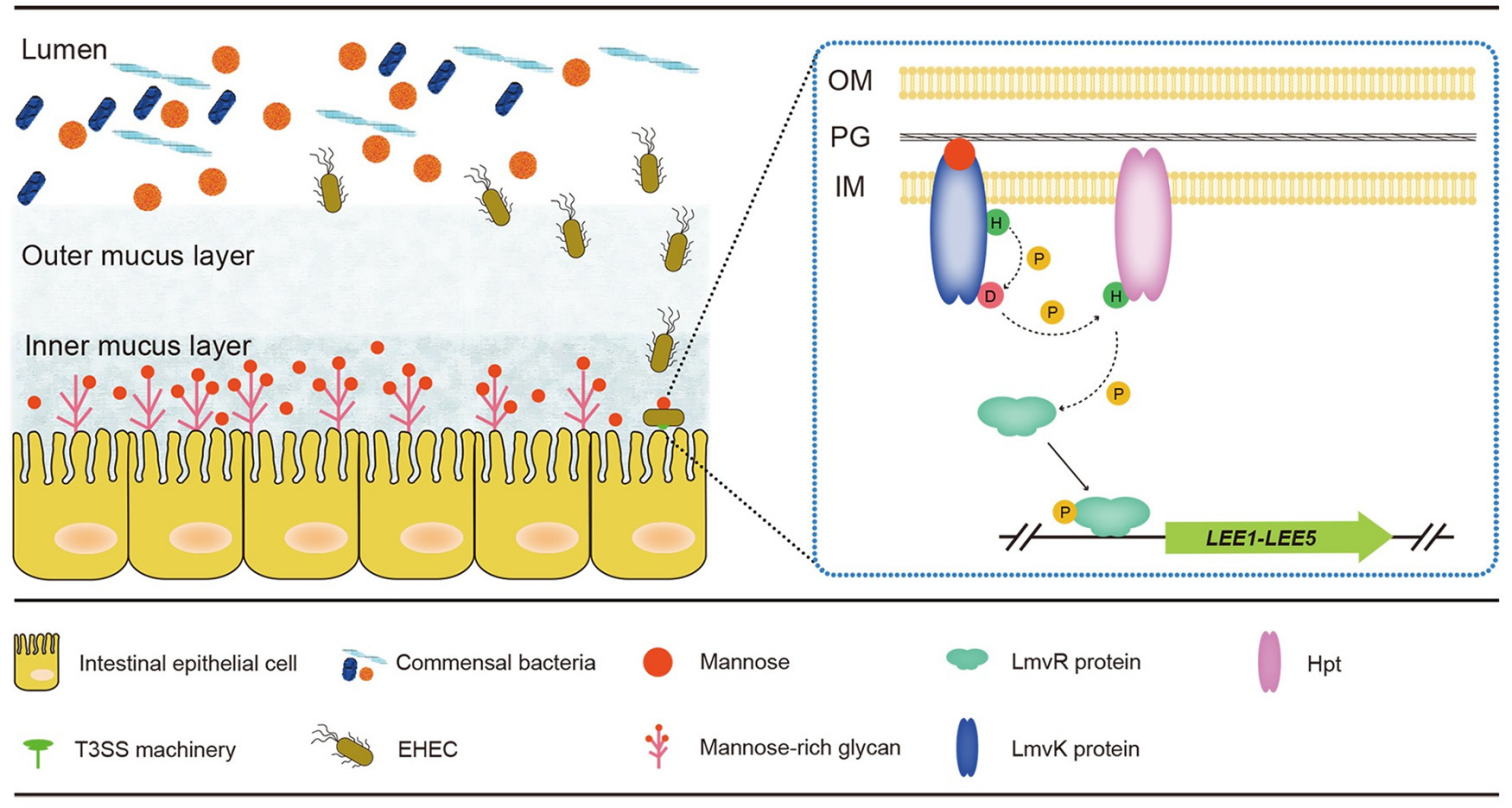
Proposed schematic model illustrating how *lmvK* and *lmvR* promote EHEC virulence *in vivo*. As EHEC bacteria swim through the mucus layer and near epithelial cells, they sense the presence of mannose via the HK LmvK. LmvK transphosphorylates the RR LmvR via an unknown Hpt (protein with a histidine phosphotransfer domain), and phosphorylated LmvR then binds to the *ler* promoter and upregulates T3SS expression. This mechanism allows EHEC to recognize the host metabolic niche and tune virulence expression. OM, outer membrane; IM, inner membrane; PG, peptidoglycan; H, histidine; D, aspartate; P, phosphoryl group. Dashed arrows indicate steps that require more experimental evidence.

The human GI tract is covered by mucins, and in many cases, this is taken advantage of by enteric pathogens, which can use mucin as a cue to upregulate virulence gene expression (42, 43). Human mucin comprises two distinct subgroups: secreted mucins and membrane-associated mucins. The outer layer of mucus contains primarily secreted mucin 2 (MUC2), which is rich in O-glycan with copious fucose groups (44). This area is close to the lumen yet distant from the cell surface; moreover, this layer of mucus is populated by gut microbes, mostly anaerobes due to the lack of oxygen (45). When EHEC enters this region, it uses the HK FusK to sense the presence of fucose and the RR FusR to repress the expression of T3SS and fucose utilization genes to avoid futile energy expenditures and competition with the microbiota for fucose (4). As a “safety net,” the T3SS is further inhibited in the absence of oxygen (27). As EHEC nears epithelial cells, it encounters membrane-associated mucins, which are characterized by N-glycosylation with abundant mannose groups (30). Expectedly, EHEC encodes the metalloprotease StcE (46, 47) and serine protease autotransporters of *Enterobacteriaceae* (SPATEs), which have mucin-degrading activities and liberate sugars from mucin (48). This inner layer of mucus is considered to be microaerobic due to oxygen diffusion from the cells (26–28). Notably, the function of LmvK/LmvR requires oxygen (Fig. 3). Together, our data suggest a model in which LmvK senses the presence of mannose, allows EHEC to recognize that it is in the proximity of host cells, and then triggers LmvR to upregulate T3SS expression to initiate intimate attachment (Fig. 8). Therefore, the acquisition and maintenance of the TCS *lmvK*-*lmvR* in EHEC facilitates niche recognition and tunes virulence expression on desired temporal and spatial occasions, using energy efficiently and promoting overall fitness. Our work illustrates an elegant example of how EHEC integrates host environmental cues into a virulence regulatory network.

Consistent with its abundance on the cell surface, mannose is a preferred mucin-derived sugar utilized by EHEC during colonization of mice, which is unfavored by nonpathogenic commensal E. coli strains (29). It seems unsurprising that EHEC carries an extra set of genes (*lmuKAIZYX*) involved in mannose utilization in addition to ManA. Interestingly, our results indicated that *manA* is critically important for growth on mannose *in vitro*, contributing substantially more than *lmuKAIZYX*. This was also reflected in an *in vivo* mouse infection model in which *manA* was required for wild-type levels of colonization (29). In contrast, *lmuKAIZYX* was dispensable for EHEC infection in a mouse model, even in the Δ*manA* background (Fig. 7), although we did not test the role of *lmuKAIZYX* in the HCM infection model. The neglectable role of *lmuKAIZYX* could be at least partially explained by the *K_m_* value of ManA (1.34 mM), which is much lower than that of LmuI (19.8 mM), suggesting a higher substrate affinity of ManA (49). Therefore, the major advantage provided by the OI167 island is the upregulation of the T3SS by LmvK/LmvR in the presence of mannose instead of the utilization of mannose as a nutrient. It is not uncommon in A/E pathogens that certain nutrients contribute to virulence via signaling to modulate virulence rather than via metabolism. l-Arginine induces T3SS expression via its sensing regulator ArgR, regardless of arginine metabolism (41). The nitrogen source ethanolamine is used by EHEC as a direct signal regulating T3SS expression independent of metabolism (50). In C. rodentium, a natural murine pathogen and a surrogate model for EHEC, 1,2-propanediol produced by the microbiota is converted to propionate, which is an inducer of T3SS expression; however, the utilization of 1,2-propanediol as a carbon source plays no role in virulence (22). Our findings reinforce the ability of EHEC to integrate information on the nutritional landscape with the regulation of virulence expression.

DNase I footprint assays *in vitro* identified the LmvR binding site at the *lmuX* promoter region (Fig. 4E). Additionally, ChIP-seq was employed to study the *in vivo* binding sites of LmvR on a global scale. These techniques combined with RNA-seq allowed us to define the LmvR regulon and elucidate regulatory mechanisms, thereby providing a comprehensive regulatory picture of LmvR. Our RNA-seq data indicated that in addition to OI167 island genes and T3SS-related genes, LmvR had the potential to influence more genes at the transcriptional level. Notably, more than 15 genes related to flagellar biosynthesis were upregulated by overexpressed *lmvR*; however, the loss of *lmvR* did not affect flagellar gene expression in DMEM supplemented with glucose (1 g/L) or mannose (1 g/L) (see Fig. S5 in the supplemental material). This may be explained by the fact that when T3SS genes are highly expressed, flagella are repressed (51, 52); thus, certain repressing factors could mask the role of LmvR under these conditions. Our data also unraveled that LmvR directly regulated a few other genes involved primarily in metabolism (Table 1). The upregulation of *lacI* by LmvR could promote the further repression of the lactose utilization operon, suggesting that lactose is not preferentially used *in vivo*. LmvR upregulated RS04255, which encodes an LpxR family lipid A deacylase, and this protein modifies lipid A and plays a role in modulating host responses (53, 54). OmpF and GadW promote the acid resistance of E. coli (55, 56); thus, these genes could increase EHEC fitness *in vivo* during passage through acidic gastric environments and within acidic macrophage lysosomes. QseE, another target gene of LmvR, encodes an HK that positively regulates the T3SS and pedestal formation in response to signals such as epinephrine and phosphate (57, 58); therefore, LmvR may affect virulence in an indirect manner. Despite these findings, more experiments are needed to delineate the roles of these regulators in EHEC virulence and physiology.

10.1128/mbio.03152-22.5 FIG S5 Loss of *lmvR* did not affect flagellar gene expression in the presence of mannose or glucose. Bacteria were cultured in DMEM supplemented with the indicated sugar (1 g/L). qPCR was used to analyze relative gene transcription. The *rpoB* gene was used as an internal control, and gene transcription levels in the WT were set to a value of 1. Dashed lines represent a 2-fold change cutoff, and a fold change of ≥2 was considered significant. Data represent the means ± standard deviations (*n *= 3). Download FIG S5, TIF file, 0.4 MB. Copyright © 2023 Yang et al. 2023 Yang et al. https://creativecommons.org/licenses/by/4.0/ This content is distributed under the terms of the Creative Commons Attribution 4.0 International license.

An intriguing finding of this study is that the activation of the LEE by LmvR requires the presence of both P1 and P2 promoters (Fig. 5). It is well recognized that the regulation of the LEE is highly complex and dynamic, with >10 protein regulators being directly involved. Certain regulators exert effects on a single promoter, for example, GrlA on P1 and FusR on P2, while other regulators bind to and function at both P1 and P2, such as QseA (59, 60). Our data revealed that the LmvR binding site lies upstream of the P2 promoter but overlaps the −10 motif of the P1 promoter. Such a genetic organization may enable LmvR to interact with both P1 and P2 promoters as well as RNA polymerases. Future work on the identification of proteins that interact with LmvR would help to elucidate the underlying molecular regulatory mechanism. The finding that the existence of P1 and P2 allows LmvR to activate LEE expression potentially helps us understand why EHEC retains two promoters whereas EPEC and C. rodentium carry only P1 (61, 62) and do not carry *lmvK* or *lmvR*.

In summary, the present work describes a new layer of virulence regulation in response to host-derived signals, illustrating how EHEC recognizes the host interface and establishes infections. Our study emphasizes the importance of integrating multiple signal inputs (mannose and oxygen, etc.) when studying bacterial pathogens with complex virulence regulatory networks. Importantly, our findings could aid in the design of novel interventions to combat EHEC infections as well as shed light on virulence regulation and bacterial pathogenesis in general.

## MATERIALS AND METHODS

### Ethics statement.

Mouse handling and care were performed according to Beijing administration guidelines for the use of laboratory animals. The entire protocol for animal experiments was approved by the Review Board of Harbin Veterinary Research Institute and the Animal Care and Use Committee of Heilongjiang Province [SYXK(H)2006-032].

All human tissue was obtained with informed consent from individuals at the Second Affiliated Hospital of Harbin Medical University. The institutional review board (protocol KY2021-144) of Harbin Medical University approved this study.

### Bacterial strains, plasmids, cell culture, and growth conditions.

The strains and plasmids used in this study are listed in Table S1 in the supplemental material. For genetic manipulations, all E. coli strains were grown routinely in lysogenic broth (LB) at 37°C in a shaking incubator. Aerobic growth was achieved by shaking in air at 180 rpm, and anaerobic and microaerobic conditions were achieved by incubation in a sealed jar with MGC AnaeroPack pouches (Mitsubishi Gas Chemical Company, Japan) and static growth (21), respectively. Trimethylamine *N*-oxide (TMAO) (20 mM) was added as an electron acceptor during anaerobic growth in M9 minimal medium (31). To assay for virulence gene expression, EHEC strains were cultured in sugar-free DMEM supplemented with the indicated sugar. To evaluate the utilization of carbon sources by EHEC, bacteria were grown in M9 minimal medium containing the indicated sugar (10 mM) as the sole carbon source and supplemented with M9 salts, 2 mM MgSO_4_, 0.1 mM CaCl_2_, and 1 mg/mL vitamin B_1_. The following selective antibiotics were added when necessary: ampicillin (Amp) at 100 μg/mL, kanamycin (Kan) at 50 μg/mL, and chloramphenicol (Chl) at 25 μg/mL. HeLa cells were cultured in high-glucose (4.5 g/L) DMEM.

10.1128/mbio.03152-22.6 TABLE S1 Bacterial strains and plasmids used in this study. Download Table S1, DOCX file, 0.02 MB. Copyright © 2023 Yang et al. 2023 Yang et al. https://creativecommons.org/licenses/by/4.0/ This content is distributed under the terms of the Creative Commons Attribution 4.0 International license.

### Genetic engineering and construction of recombinant plasmids.

DNA amplification, ligation, and electroporation were performed according to standard protocols. All oligonucleotides were synthesized and provided by the Comate Bioscience Company (Changchun, China) and are listed in Table S2. The various constructs were confirmed by PCR and DNA sequencing (Comate, China).

10.1128/mbio.03152-22.7 TABLE S2 Oligonucleotides used in this study. Download Table S2, DOCX file, 0.02 MB. Copyright © 2023 Yang et al. 2023 Yang et al. https://creativecommons.org/licenses/by/4.0/ This content is distributed under the terms of the Creative Commons Attribution 4.0 International license.

Gene deletions in EHEC were achieved using the lambda Red recombination system described previously (31, 63). To construct the pLmvR complementation plasmid, the coding region of *lmvR* was amplified from EDL933 genomic DNA (gDNA) and cloned into pGEN-Ptac using the NdeI and HindIII sites. The resultant plasmid carrying P_tac_-driven *lmvR* was transformed into the Δ*lmvR* mutant (for complementation) or the WT (for RNA-seq) and used without IPTG due to leaky expression (64). To construct the pLmvK complementation plasmid, the coding region of *lmvK* plus 300 bp upstream containing the putative promoter of *lmvK* was amplified from EDL933 genomic DNA and cloned into single-copy plasmid pCC1BAC (Epicentre) using the EcoRI and BamHI sites. The resultant plasmid, pLmvK, was transformed into the Δ*lmvK* mutant for complementation. To construct promoter-*lacZ* transcriptional fusion plasmids, various promoter fragments were cloned into pCJ112 (65) using the EcoRI and XbaI sites.

### PCR genotyping.

Avian and uropathogenic E. coli isolates were from our laboratory stock (66, 67), and EPEC, EHEC, and enterotoxigenic E. coli isolates were kindly provided by Harley Moon and Nancy Cornick (68, 69). Duplex PCR was performed according to methods described previously (5, 67).

### Growth assays.

For growth assays with a sole carbon source, cultures grown overnight were diluted 1:100 in LB and grown to an optical density at 600 nm (OD_600_) of ~1, followed by three washes with phosphate-buffered saline (PBS) to remove residual LB medium. Bacterial pellets were resuspended in carbon source-free M9 minimal medium and diluted 1:100 in M9 medium with lyxose or mannose as the sole carbon source (10 mM) in six-well plates with 2.5 mL of medium in each well. Plates were statically incubated at 28°C, and an aliquot of the culture was taken from a well at each time point for CFU or OD_600_ measurements.

### Protein expression and purification.

Protein expression and purification were performed according to methods described previously by Cai et al. (31), with minor modifications. Briefly, the coding sequence of *lmvR* or *lmuK* was cloned into the pET28a vector using the NdeI and HindIII sites, which resulted in the fusion of 6×His to the N terminus of LmvR or LmuK. To achieve a high expression level of fusion proteins, the recombinant plasmid was transformed into E. coli BL21(DE3) competent cells, and expression was induced by IPTG at a final concentration of 0.1 mM at 16°C for 10 h. Protein purification based on affinity chromatography was performed using a HisTrap HP column (Cytiva) according to the manufacturer’s instructions. The eluted recombinant protein was dialyzed against binding buffer for LmvR (10 mM Tris [pH 7.5], 1 mM EDTA, 1 mM dithiothreitol, 50 mM KCl, 50 mM MgCl_2_, 1 μg/mL bovine serum albumin [BSA], and 10% glycerol) or kinase reaction buffer for EDL5429. Protein concentrations were determined using a bicinchoninic acid (BCA) protein assay kit (Pierce), and protein purity was assessed by SDS (sodium dodecyl sulfate)-PAGE.

### Carbohydrate kinase activity assay.

A carbohydrate kinase activity assay with the purified LmuK protein was performed according to methods described previously (70), in which the consumption of ATP by sugar kinase was represented by the ADP content that was coupled to the oxidation of NADH via reactions catalyzed by pyruvate kinase (PK) and lactate dehydrogenase (LDH). The depletion of NADH was monitored and indicated by the reduction in the absorbance at 340 nm.

### Transcriptomics by RNA sequencing.

RNA sequencing (RNA-seq) analysis was performed using a standard protocol, with minor modifications (5, 71). WT/vector and WT/pLmvR were cultured aerobically in LB at 37°C to early stationary phase, followed by RNA extraction and library construction (NEBNext library prep kit for Illumina). Sequencing of the libraries was performed using a 2× 150-bp paired-end (PE) configuration on an Illumina HiSeq platform. The clean reads were aligned to the EDL933 genome (GenBank accession number NZ_CP008957.1) using Bowtie 2 (version 2.1.9, with standard options). Differential gene expression analysis was performed using DESeq2 (version 1.6.3) with R version 3.3.2 according to a standard workflow (71). All genes with a |log_2_(fold change)| value of ≥1 and a Benjamini-Hochberg-adjusted *P* value (*q* value) of <0.05 were considered differentially expressed (72).

### Reverse transcription-PCR and reverse transcription-quantitative PCR.

Total RNA was extracted using TRIzol reagent according to the manufacturer’s instructions (Invitrogen). RNA samples were treated to remove genomic DNA and reverse transcribed to cDNA using a PrimeScript reverse transcription (RT) reagent kit with gDNA eraser (Clontech). For the cotranscription test using RT-PCR, primer pairs were designed to span adjacent genes. RNA that was not reverse transcribed served as a negative control, while genomic DNA served as a positive control (31, 73).

For SYBR green-based qPCR analysis, EHEC cells were cultured statically in M9 medium (25 mM lyxose or mannose at 28°C) or DMEM (25 mM glucose or mannose at 37°C) to early stationary phase. cDNA was used as a template, using TB green premix Ex *Taq* II reagent (Clontech) and an ABI Quant 5 thermocycler (Applied Biosystems). Melting curve analyses were performed after each reaction to ensure amplification specificity. Fold changes in transcript levels were calculated using the 2^−ΔΔ^*^CT^* method (74), and the levels were normalized to the *rpoB* expression level.

### Electrophoretic mobility shift assay and DNase I footprint assay.

To study the binding of LmvR to DNA probes, electrophoretic mobility shift assays (EMSAs) were performed using a commercial EMSA kit (Invitrogen, CA) (31, 75). EMSAs were performed by the addition of increasing amounts of protein to the DNA probe (10 ng) in binding buffer supplemented with acetyl phosphate, followed by a 30-min incubation at 37°C. The reaction mixtures were subjected to electrophoresis on a 6% polyacrylamide gel in 0.5× Tris-borate-EDTA (TBE) buffer on ice for 30 min at 100 V plus 60 min at 150 V. The gel was stained in 0.5× TBE buffer containing 1× SYBR gold nucleic acid stain (Invitrogen) for 15 min. The gels were visualized and photographed using a ChampGel 7000 imager (SageCreation).

The DNase I footprint assay was performed as previously reported (76, 77), with minor modifications. The *lmuX* promoter region (P*_lmuX_*) was cloned into the pBlunt T vector, which resulted in the pBlunt-P*_lmuX_* recombinant plasmid. The 6-carboxyfluorescein (FAM)-labeled probes were PCR amplified using FAM-modified primers and pBlunt-P*_lmuX_* as a template, followed by gel purification. For each assay, 1 pmol of the probe was incubated with different amounts of protein in a 40-μL reaction mix. After incubation as described above for the EMSAs, 10 μL of a solution containing ~0.005 U of DNase I (Promega) was added, and the mixture was further incubated for 5 min at 37°C. The reaction was terminated by the addition of 100 μL of 0.5 M EDTA (pH 8.0) to the mixture. Samples were treated with phenol-chloroform, followed by ethanol precipitation to harvest the DNA fragments. The DNA fragments and the GeneScan-LIZ600 size standard (Applied Biosystems) were analyzed using a 3730 DNA analyzer (Thermo Scientific) and Peak Scanner v2 software (Applied Biosystems).

### Rapid amplification of cDNA ends.

The TSS of *lmuX* was identified using the SMARTer 5′/3′ rapid amplification of cDNA ends (RACE) kit (Clontech) according to the manufacturer’s instructions (65). RNA from wild-type EDL933 was isolated as described above. The complete removal of DNA contamination was confirmed by RT-PCR. Approximately 5 μg of RNA was reverse transcribed using gene-specific primers, and nested PCR was performed to obtain PCR products that were subsequently cloned into the pRACE vector provided with the kit. Multiple constructs were selected and subjected to DNA sequencing and sequence analysis for the identification of the TSS.

### β-Galactosidase activity.

A β-galactosidase assay was performed according to methods described previously (31), with minor modifications. Briefly, bacteria were grown in DMEM (25 mM mannose at 37°C) to early stationary phase, followed by harvesting and washing with PBS, and bacteria were then diluted properly in Z buffer. These cultures were diluted 1:10 in Z buffer and assayed for β-galactosidase activity using *ortho*-nitrophenyl-β-galactoside (ONPG) as a substrate.

### ChIP-seq.

A plasmid carrying C-terminally Flag-tagged LmvR (pLmvR-2Flag) driven by P_tac_ was constructed. The Δ*lmvR* mutant carrying pLmvR-2Flag was grown in LB and DMEM (25 mM mannose) to early stationary phase and then cross-linked with formaldehyde at a final concentration of 1% (vol/vol) for 15 min at room temperature, with gentle agitation. Cross-linking was terminated with glycine at a final concentration of 125 mM, followed by 3 washes with ice-cold phosphate-buffered saline buffer. Bacterial pellets were resuspended in lysis buffer (20 mM Tris-HCl [pH 8.1], 150 mM NaCl, 1 mM EDTA, 1% [vol/vol] Triton X-100, 0.1% [wt/vol] sodium deoxycholate [DOC], 0.1% [wt/vol] SDS, a complete protease inhibitor cocktail [Roche], and 1 mM phenylmethylsulfonyl fluoride [PMSF]), and chromatin was fragmented on ice using a sonicator. Bacterial debris was removed via centrifugation for 10 min at 14,000 rpm, and a fraction of the supernatant was stored as the input sample for immunoprecipitation (IP) assays.

Protein G magnetic beads (Thermo Scientific) were incubated with the fragmented chromatin and anti-Flag monoclonal antibody (Sigma) for 4 h at 4°C on a rotator. The beads were washed once with a solution containing 20 mM Tris-HCl (pH 8.1), 50 mM NaCl, 2 mM EDTA, 1% Triton X-100, and 0.1% SDS; twice with a solution containing 10 mM Tris-HCl (pH 8.1), (250 mM LiCl, 1 mM EDTA, 1% NP-40, and 1% deoxycholic acid); and twice with 1× Tris-EDTA (TE) buffer (10 mM Tris-HCl [pH 7.5] and 1 mM EDTA). Bound DNA was reverse cross-linked, eluted from the beads in elution buffer (100 mM NaHCO_3_, 1% SDS) for 16 h at 65°C, and treated with RNase A for 1 h at 37°C and then with proteinase K for 30 min at 55°C. Immunoprecipitated DNA was purified and used to construct sequencing libraries with a Nextflex ChIP-seq kit, followed by sequencing on an Illumina HiSeq X ten platform with PE 150-bp chemistry.

Clean reads were mapped to the EDL933 genome using BWA (78). MACS2 software was used to identify peaks using default parameters (bandwidth, 300 bp; model fold, 5, 50; *q* value, 0.05) (79).

### FAS assays on HeLa cells.

Adhesion and FAS assays on HeLa cells were performed as described previously, with minor modifications (53). HeLa cells were grown in high-glucose DMEM in confocal dishes to ~80% confluence at 37°C in a 5% CO_2_ atmosphere. Bacteria were grown in LB until the OD_600_ reached ~1.0, pelleted, and resuspended in sugar-free DMEM. Before cell infection, the cell culture medium was replaced with DMEM containing mannose (10 mM) or glucose (1 g/L), as indicated. HeLa cells were infected with EHEC at a multiplicity of infection (MOI) of 40 for 2.5 h, and the dishes were washed to remove unattached bacteria and incubated for another 1 h. The dishes were washed, fixed with 4% paraformaldehyde, and permeabilized with 0.1% Triton X-100. To visualize actin aggregation, samples were treated with Alexa Fluor 488-labeled phalloidin (Thermo). To visualize bacterial DNA and HeLa nuclei, samples were stained with propidium iodide (Thermo). Confocal dishes were mounted using fluorescent mounting medium (Prolong Diamond antifade mountant). Imaging was performed using a Zeiss LSM880 confocal laser scanning microscope with Fast Airyscan or phase-contrast fluorescence microscopy (Evos M5000; Thermo). For the quantification of adhesion on each HeLa cell, at least 25 random microscopic fields were obtained per biological replicate, and A/E lesions were counted as areas of intense actin condensation beneath bacterial cells.

### EHEC colonization in a murine model.

Mouse infection studies were performed as previously reported (80, 81), with slight modifications. Following a 7-day acclimation period, groups of 12 mice were starved for 12 h and then given streptomycin (5 g/L) in drinking water for 24 h to disrupt the resident microbiota. EDL933str (Δ*lacZ*) and mutant strains (derived from EDL933str, *lacZ*^+^) in early-stationary-phase growth were pelleted, resuspended in sterile PBS, mixed in equal numbers, and adjusted to create the challenge inocula. To determine the initial CFU per milliliter, dilutions of each inoculum were plated onto MacConkey plates with streptomycin. Six-week-old female BALB/c mice were inoculated via oral gavage with 100 μL (2 × 10^9^ CFU) of the challenge inoculum per mouse. Mice were maintained on streptomycin (0.5 g/L) in drinking water after inoculation. Three days after challenge, 1-cm cecum sections were collected, and the luminal contents were removed. The cecum samples were then gently rinsed in sterile PBS 3 times to remove residual feces and unattached microbes. Dilutions of the tissue homogenates were plated onto MacConkey plates with streptomycin to determine the bacterial concentration (CFU per gram) in the tissue. The competitive index is defined as the ratio of mutant to WT bacteria isolated divided by the ratio of mutant to WT bacteria in the inoculum.

### Preparation and culture of HCMs.

The development of a human colonoid model was performed as previously described (82, 83). Briefly, three-dimensional (3D) human colonoids were grown in a Matrigel matrix (Matrigel basement membrane; Corning) using IntestiCult organoid growth medium (Stemcell). Fresh human colon tissue was placed into PBS containing sodium cefoxitin and transported at 4°C to the laboratory immediately for the isolation of crypt stem cells according to the manufacturer’s instructions (Stemcell). Tissue chunks were finely minced into ~1- to 3-mm pieces using sterile scissors, and the pieces were washed thoroughly with Dulbecco’s PBS (D-PBS) until the liquid became clear. The tissue pieces were transferred to a 15-mL conical tube containing 10 mL of GCDR (gentle cell dissociation reagent). After lysis on ice for 30 min at 40 rpm, the supernatant was removed by centrifugation at 290 × *g* for 5 min at 4°C, and the pellet of tissue fragments was collected. The pellet was pipetted >30 times in 5 mL of DMEM–F-12 medium containing 1% BSA, followed by passage through a Falcon 70-μm cell strainer. The filtrate was collected and centrifuged at 290 × *g* for 5 min at 4°C, which yielded a pellet that contained the initial isolated colonic crypt stem cells. The crypt stem cells were resuspended in a mixture of DMEM–F-12 medium (containing 1% BSA) and matrix gel and plated dropwise into the wells of a 24-well plate. After a 10-min incubation at 37°C, the matrix gel fully solidified, and 750 μL of IntestiCult organoid growth medium (Human) was added to support the growth of cells in a 37°C cell culture chamber with 5% CO_2_.

To generate HCMs on 96-well plates, each well was first coated with a diluted Matrigel matrix (Corning) and incubated for 1 h in a 37°C incubator. Undifferentiated 3D colonoids were collected from the Matrigel dome and washed with ice-cold DMEM–F-12 medium, followed by 5 min of dissociation with 0.05% trypsin–EDTA (0.5 mM) at 37°C. Single-cell suspensions in monolayer growth medium were seeded into individual wells of a 96-well plate. After 5 to 7 days, the cells reached 80 to 90% confluence. Organoid differentiation medium replaced the monolayer growth medium, and the monolayer cells were allowed to differentiate for several days before use for infection assays.

### EHEC infection in a colonoid model.

EHEC infection assays in a human colonoid model were performed as previously described, with minor modifications (84). Briefly, a fresh single colony of EHEC was inoculated into 5 mL of LB medium with or without antibiotics, as indicated, and allowed to grow overnight. The culture was diluted 1:100 in 10 mL of LB and allowed to grow for ~3 h until an OD_600_ of 1.0 was reached. HCMs were prepared and differentiated as described above, and each well of a 96-well plate contained approximately 10^5^ to 5 × 10^5^ colonoid cells before infection. Bacteria resuspended in the differentiation medium were added to colonoid monolayers at an MOI of 10 and incubated for 4 h at 37°C in a 5% CO_2_ atmosphere.

Notably, the optimal MOI and time of infection were determined empirically. A lower MOI resulted in ineffective adherence, and a higher MOI caused cell detachment from the plates. To assess the adherence of EHEC, HCMs were washed 3 times with PBS and then lysed with 1% Triton X-100. Bacteria were enumerated by determining the CFU in each well.

### Giemsa staining.

Giemsa staining of HCMs and bacteria was performed according to methods described previously by Rajan et al. (85) and Tatsuno et al. (86). HCMs were infected with EHEC strains as described above. Bacteria and infected cells were imaged under a Zeiss Primovert inverted microscope at a ×40 magnification. Bacterial clusters on HCMs containing >8 bacteria were considered MCs (86). The number of MCs was scored as the sum from 20 random microscopic fields. A technician counted the MCs in a blind manner to ensure objectivity.

### Data availability.

The RNA-seq and ChIP-seq data that support the findings of this study are openly available at the National Microbiology Data Center (accession numbers NMDC10018018 and NMDC10018019).
