# Supplementary material for: Genomic Island-Encoded Histidine Kinase and Response Regulator Coordinate Mannose Utilization with Virulence in Enterohemorrhagic Escherichia coli
Source: mBio. 2023 Feb 14;14(2):e03152-22. doi: 10.1128/mbio.03152-22 (PMC10128022; doi:10.1128/mbio.03152-22)
Supplement: TABLE S3 [file mbio.03152-22-s0003.docx]

| **Table S3. Comparative transcriptomics of EDL933 (pLmvR) versus wild-type EDL933 (vector) using RNA-seq.** | | | | | | | | | |
| --- | --- | --- | --- | --- | --- | --- | --- | --- | --- |
| geneID | foldChange | log2FoldChange | pval | padj | GeneName | Chr | locus_tag | old_locus_tag | Product |
| gene-EDL933_RS26880 | 719.8535818 | 9.491559682 | 0 | 0 | EDL933_RS26880 | NZ_CP008957.1 | EDL933_RS26880 | EDL933_5428 | response regulator transcription factor |
| gene-EDL933_RS26910 | 694.8665482 | 9.440592119 | 1.42E-307 | 3.64E-304 | EDL933_RS26910 | NZ_CP008957.1 | EDL933_RS26910 | EDL933_5434 | sugar ABC transporter ATP-binding protein |
| gene-EDL933_RS26905 | 407.8142829 | 8.671768494 | 3.54E-239 | 6.02E-236 | EDL933_RS26905 | NZ_CP008957.1 | EDL933_RS26905 | EDL933_5433 | ribose ABC transporter permease |
| gene-EDL933_RS26895 | 25.93374715 | 4.69675877 | 1.36E-91 | 9.90E-89 | EDL933_RS26895 | NZ_CP008957.1 | EDL933_RS26895 | EDL933_5431 | D-lyxose/D-mannose family sugar isomerase |
| gene-EDL933_RS26890 | 25.63223883 | 4.67988759 | 5.29E-121 | 5.40E-118 | EDL933_RS26890 | NZ_CP008957.1 | EDL933_RS26890 | EDL933_5430 | ketose 1%2C6-bisphosphate aldolase |
| gene-EDL933_RS26900 | 23.56344562 | 4.558478611 | 1.11E-127 | 1.42E-124 | EDL933_RS26900 | NZ_CP008957.1 | EDL933_RS26900 | EDL933_5432 | ABC transporter substrate-binding protein |
| gene-EDL933_RS26885 | 19.98742963 | 4.321021049 | 9.39E-105 | 7.99E-102 | EDL933_RS26885 | NZ_CP008957.1 | EDL933_RS26885 | EDL933_5429 | carbohydrate kinase family protein |
| gene-EDL933_RS24275 | 5.808490404 | 2.538163264 | 0.000192127 | 0.006371373 | escG | NZ_CP008957.1 | EDL933_RS24275 | EDL933_4939 | type III secretion system LEE needle protein cochaperone EscG |
| gene-EDL933_RS24380 | 4.945339852 | 2.306069669 | 1.58E-07 | 1.35E-05 | EDL933_RS24380 | NZ_CP008957.1 | EDL933_RS24380 | -- | EscI/YscI/HrpB family type III secretion system inner rod protein |
| gene-EDL933_RS08000 | 4.470457462 | 2.16042247 | 3.77E-24 | 2.40E-21 | flgB | NZ_CP008957.1 | EDL933_RS08000 | EDL933_1650 | flagellar basal body rod protein FlgB |
| gene-EDL933_RS02115 | 4.449429251 | 2.153620287 | 2.02E-21 | 1.15E-18 | EDL933_RS02115 | NZ_CP008957.1 | EDL933_RS02115 | EDL933_0421 | iron ABC transporter permease |
| gene-EDL933_RS08005 | 4.153018184 | 2.054160189 | 1.28E-10 | 1.64E-08 | flgC | NZ_CP008957.1 | EDL933_RS08005 | EDL933_1651 | flagellar basal body rod protein FlgC |
| gene-EDL933_RS08010 | 4.076894469 | 2.027470613 | 4.55E-19 | 1.94E-16 | flgD | NZ_CP008957.1 | EDL933_RS08010 | EDL933_1652 | flagellar hook assembly protein FlgD |
| gene-EDL933_RS24390 | 3.892195953 | 1.960584345 | 2.78E-10 | 3.38E-08 | sepD | NZ_CP008957.1 | EDL933_RS24390 | -- | type III secretion system LEE switch protein SepD |
| gene-EDL933_RS26625 | 3.857522282 | 1.94767449 | 3.71E-15 | 8.24E-13 | malK | NZ_CP008957.1 | EDL933_RS26625 | EDL933_5372 | maltose/maltodextrin ABC transporter ATP-binding protein MalK |
| gene-EDL933_RS24385 | 3.687590693 | 1.882678532 | 1.53E-05 | 0.000787776 | escJ | NZ_CP008957.1 | EDL933_RS24385 | EDL933_4961 | type III secretion system LEE inner membrane ring protein EscJ |
| gene-EDL933_RS24320 | 3.649515708 | 1.867705031 | 6.32E-15 | 1.35E-12 | cesT | NZ_CP008957.1 | EDL933_RS24320 | EDL933_4948 | type III secretion system LEE chaperone CesT |
| gene-EDL933_RS08020 | 3.475654065 | 1.797284497 | 2.90E-15 | 7.41E-13 | EDL933_RS08020 | NZ_CP008957.1 | EDL933_RS08020 | EDL933_1654 | flagellar basal body rod protein FlgF |
| gene-EDL933_RS08015 | 3.427752029 | 1.777262745 | 5.45E-16 | 1.55E-13 | EDL933_RS08015 | NZ_CP008957.1 | EDL933_RS08015 | EDL933_1653 | flagellar hook protein FlgE |
| gene-EDL933_RS08595 | 3.302455682 | 1.723539201 | 0.000162747 | 0.005540985 | EDL933_RS08595 | NZ_CP008957.1 | EDL933_RS08595 | -- | hypothetical protein |
| gene-EDL933_RS24315 | 3.296219447 | 1.720812294 | 1.74E-14 | 3.30E-12 | eae | NZ_CP008957.1 | EDL933_RS24315 | EDL933_4947 | intimin type gamma |
| gene-EDL933_RS24300 | 3.292672525 | 1.719259035 | 1.52E-17 | 4.55E-15 | espA | NZ_CP008957.1 | EDL933_RS24300 | -- | type III secretion system LEE translocon filament protein EspA |
| gene-EDL933_RS10455 | 3.273447362 | 1.71081078 | 0.000102389 | 0.003887323 | EDL933_RS10455 | NZ_CP008957.1 | EDL933_RS10455 | EDL933_2137 | fimbrial protein |
| gene-EDL933_RS04055 | 3.168932608 | 1.663996979 | 5.44E-21 | 2.78E-18 | nadA | NZ_CP008957.1 | EDL933_RS04055 | EDL933_0823 | quinolinate synthase NadA |
| gene-EDL933_RS14445 | 3.138578597 | 1.650111338 | 9.75E-07 | 6.73E-05 | fliL | NZ_CP008957.1 | EDL933_RS14445 | EDL933_2952 | flagellar basal body-associated protein FliL |
| gene-EDL933_RS24310 | 3.108074023 | 1.636020864 | 1.18E-06 | 7.80E-05 | escD | NZ_CP008957.1 | EDL933_RS24310 | EDL933_4946 | type III secretion system LEE inner membrane ring protein EscD |
| gene-EDL933_RS24400 | 3.100328754 | 1.632421205 | 8.90E-06 | 0.000488884 | cesD | NZ_CP008957.1 | EDL933_RS24400 | EDL933_4964 | type III secretion system LEE chaperone CesD |
| gene-EDL933_RS18345 | 3.021997226 | 1.595502336 | 1.38E-17 | 4.40E-15 | nadB | NZ_CP008957.1 | EDL933_RS18345 | EDL933_3739 | L-aspartate oxidase |
| gene-EDL933_RS08025 | 2.940340024 | 1.555983 | 1.05E-12 | 1.68E-10 | EDL933_RS08025 | NZ_CP008957.1 | EDL933_RS08025 | EDL933_1655 | flagellar basal-body rod protein FlgG |
| gene-EDL933_RS08030 | 2.938286134 | 1.554974894 | 3.28E-07 | 2.66E-05 | flgH | NZ_CP008957.1 | EDL933_RS08030 | EDL933_1656 | flagellar basal body L-ring protein FlgH |
| gene-EDL933_RS24270 | 2.929550192 | 1.550679168 | 1.69E-11 | 2.34E-09 | espF | NZ_CP008957.1 | EDL933_RS24270 | -- | type III secretion system LEE effector EspF |
| gene-EDL933_RS02110 | 2.898955354 | 1.535533115 | 1.35E-14 | 2.65E-12 | EDL933_RS02110 | NZ_CP008957.1 | EDL933_RS02110 | EDL933_0420 | Fe(3+) ions import ATP-binding protein FbpC |
| gene-EDL933_RS14415 | 2.853851919 | 1.512910478 | 5.25E-09 | 5.59E-07 | fliF | NZ_CP008957.1 | EDL933_RS14415 | EDL933_2946 | flagellar basal body M-ring protein FliF |
| gene-EDL933_RS24290 | 2.852296196 | 1.512123806 | 7.19E-15 | 1.47E-12 | espB | NZ_CP008957.1 | EDL933_RS24290 | EDL933_4942 | type III secretion system LEE translocon pore-forming subunit EspB |
| gene-EDL933_RS24295 | 2.826562871 | 1.499048787 | 1.43E-15 | 3.85E-13 | espD | NZ_CP008957.1 | EDL933_RS24295 | EDL933_4943 | type III secretion system LEE translocon pore-forming subunit EspD |
| gene-EDL933_RS02035 | 2.81681959 | 1.494067166 | 9.51E-20 | 4.42E-17 | EDL933_RS02035 | NZ_CP008957.1 | EDL933_RS02035 | EDL933_0405 | LacI family DNA-binding transcriptional regulator |
| gene-EDL933_RS24375 | 2.80661396 | 1.48883064 | 5.95E-12 | 8.93E-10 | espZ | NZ_CP008957.1 | EDL933_RS24375 | -- | type III secretion system LEE cytoprotective effector EspZ |
| gene-EDL933_RS24395 | 2.797562157 | 1.484170186 | 8.30E-13 | 1.41E-10 | escC | NZ_CP008957.1 | EDL933_RS24395 | EDL933_4963 | type III secretion system LEE outer membrane ring protein EscC |
| gene-EDL933_RS08600 | 2.795929551 | 1.48332801 | 6.15E-11 | 8.05E-09 | EDL933_RS08600 | NZ_CP008957.1 | EDL933_RS08600 | EDL933_1767 | type III secretion system effector EspK |
| gene-EDL933_RS24345 | 2.795588125 | 1.483151824 | 1.29E-09 | 1.43E-07 | escQ | NZ_CP008957.1 | EDL933_RS24345 | EDL933_4955 | type III secretion system LEE ring protein EscQ |
| gene-EDL933_RS24330 | 2.792560817 | 1.4815887 | 1.02E-09 | 1.15E-07 | map | NZ_CP008957.1 | EDL933_RS24330 | -- | type III secretion system LEE effector Map (Rho guanine exchange factor) |
| gene-EDL933_RS24285 | 2.783221994 | 1.476755985 | 1.07E-06 | 7.19E-05 | cesD2 | NZ_CP008957.1 | EDL933_RS24285 | EDL933_4941 | type III secretion system LEE chaperone CesD2 |
| gene-EDL933_RS33830 | 2.77889956 | 1.474513691 | 8.54E-12 | 1.25E-09 | EDL933_RS33830 | NZ_CP008957.1 | EDL933_RS33830 | -- | ATP-dependent DNA helicase RecQ |
| gene-EDL933_RS02125 | 2.743715108 | 1.456130688 | 3.23E-15 | 7.77E-13 | EDL933_RS02125 | NZ_CP008957.1 | EDL933_RS02125 | EDL933_0423 | MFS transporter |
| gene-EDL933_RS24305 | 2.740999711 | 1.454702176 | 0.000309815 | 0.009418016 | sepL | NZ_CP008957.1 | EDL933_RS24305 | EDL933_4945 | type III secretion system LEE gatekeeper SepL |
| gene-EDL933_RS12005 | 2.728347782 | 1.448027556 | 2.94E-13 | 5.35E-11 | nleA | NZ_CP008957.1 | EDL933_RS12005 | EDL933_2451 | type III secretion system effector NleA |
| gene-EDL933_RS21545 | 2.724926862 | 1.446217508 | 0.00013411 | 0.004789497 | EDL933_RS21545 | NZ_CP008957.1 | EDL933_RS21545 | EDL933_4389 | tryptophan permease |
| gene-EDL933_RS24325 | 2.703875485 | 1.435028716 | 4.91E-18 | 1.67E-15 | tir | NZ_CP008957.1 | EDL933_RS24325 | EDL933_4949 | type III secretion system LEE translocated intimin receptor Tir |
| gene-EDL933_RS02120 | 2.698652028 | 1.432238964 | 2.50E-18 | 9.13E-16 | EDL933_RS02120 | NZ_CP008957.1 | EDL933_RS02120 | EDL933_0422 | ABC transporter substrate-binding protein |
| gene-EDL933_RS02950 | 2.632477574 | 1.396421241 | 9.67E-05 | 0.00373964 | allD | NZ_CP008957.1 | EDL933_RS02950 | EDL933_0602 | ureidoglycolate dehydrogenase |
| gene-EDL933_RS14460 | 2.623527934 | 1.391508151 | 8.45E-05 | 0.003346693 | fliO | NZ_CP008957.1 | EDL933_RS14460 | EDL933_2955 | flagellar type III secretion system protein FliO |
| gene-EDL933_RS16225 | 2.619906304 | 1.389515217 | 3.62E-07 | 2.84E-05 | mglA | NZ_CP008957.1 | EDL933_RS16225 | EDL933_3311 | galactose/methyl galactoside ABC transporter ATP-binding protein MglA |
| gene-EDL933_RS16220 | 2.611174918 | 1.384699105 | 8.73E-13 | 1.44E-10 | mglC | NZ_CP008957.1 | EDL933_RS16220 | EDL933_3310 | galactose/methyl galactoside ABC transporter permease MglC |
| gene-EDL933_RS26630 | 2.58511313 | 1.370227417 | 2.45E-05 | 0.001164024 | lamB | NZ_CP008957.1 | EDL933_RS26630 | EDL933_5373 | maltoporin LamB |
| gene-EDL933_RS24440 | 2.571280958 | 1.362487259 | 0.000334004 | 0.01003386 | espL | NZ_CP008957.1 | EDL933_RS24440 | EDL933_4971 | type III secretion system LEE stator protein EspL |
| gene-EDL933_RS07100 | 2.535417081 | 1.342223093 | 4.84E-11 | 6.50E-09 | putP | NZ_CP008957.1 | EDL933_RS07100 | EDL933_1438 | sodium/proline symporter PutP |
| gene-EDL933_RS26760 | 2.516346176 | 1.331330409 | 2.04E-05 | 0.000993004 | soxS | NZ_CP008957.1 | EDL933_RS26760 | EDL933_5400 | superoxide response transcriptional regulator SoxS |
| gene-EDL933_RS23755 | 2.485311766 | 1.31342684 | 4.08E-13 | 7.19E-11 | xylF | NZ_CP008957.1 | EDL933_RS23755 | EDL933_4831 | D-xylose ABC transporter substrate-binding protein |
| gene-EDL933_RS25505 | 2.481197862 | 1.311036787 | 1.70E-06 | 0.000109783 | fadB | NZ_CP008957.1 | EDL933_RS25505 | EDL933_5166 | fatty acid oxidation complex subunit alpha FadB |
| gene-EDL933_RS24340 | 2.479430605 | 1.310008848 | 9.74E-05 | 0.003741854 | espH | NZ_CP008957.1 | EDL933_RS24340 | EDL933_4954 | type III secretion system LEE effector EspH |
| gene-EDL933_RS24460 | 2.465736498 | 1.302018633 | 0.00011013 | 0.004075592 | ler | NZ_CP008957.1 | EDL933_RS24460 | EDL933_4975 | type III secretion system LEE master regulator Ler |
| gene-EDL933_RS11425 | 2.451197424 | 1.293486686 | 9.26E-08 | 8.30E-06 | EDL933_RS11425 | NZ_CP008957.1 | EDL933_RS11425 | EDL933_2335 | amidohydrolase |
| gene-EDL933_RS20015 | 2.443257038 | 1.288805648 | 2.99E-07 | 2.46E-05 | yqeC | NZ_CP008957.1 | EDL933_RS20015 | EDL933_4077 | putative selenium-dependent hydroxylase accessory protein YqeC |
| gene-EDL933_RS05865 | 2.41266369 | 1.270626827 | 3.35E-15 | 7.77E-13 | ompF | NZ_CP008957.1 | EDL933_RS05865 | EDL933_1192 | porin OmpF |
| gene-EDL933_RS14325 | 2.399227963 | 1.262570242 | 2.58E-08 | 2.49E-06 | EDL933_RS14325 | NZ_CP008957.1 | EDL933_RS14325 | EDL933_2929 | RNA polymerase sigma factor FliA |
| gene-EDL933_RS21310 | 2.39179591 | 1.258094291 | 1.57E-07 | 1.35E-05 | tdcA | NZ_CP008957.1 | EDL933_RS21310 | EDL933_4339 | transcriptional regulator TdcA |
| gene-EDL933_RS14450 | 2.390114579 | 1.257079781 | 1.75E-07 | 1.46E-05 | fliM | NZ_CP008957.1 | EDL933_RS14450 | EDL933_2953 | flagellar motor switch protein FliM |
| gene-EDL933_RS24365 | 2.382319606 | 1.252366975 | 7.18E-10 | 8.53E-08 | escV | NZ_CP008957.1 | EDL933_RS24365 | EDL933_4958 | type III secretion system LEE export apparatus protein EscV |
| gene-EDL933_RS21350 | 2.377220088 | 1.249275477 | 0.000307677 | 0.00940903 | garD | NZ_CP008957.1 | EDL933_RS21350 | EDL933_4348 | galactarate dehydratase |
| gene-EDL933_RS23760 | 2.368248587 | 1.243820523 | 4.80E-05 | 0.002061852 | xylG | NZ_CP008957.1 | EDL933_RS23760 | EDL933_4832 | D-xylose ABC transporter ATP-binding protein |
| gene-EDL933_RS04255 | 2.34878983 | 1.231917626 | 7.88E-10 | 9.15E-08 | EDL933_RS04255 | NZ_CP008957.1 | EDL933_RS04255 | EDL933_0859 | lipid A deacylase LpxR family protein |
| gene-EDL933_RS08040 | 2.325477676 | 1.21752709 | 0.0001353 | 0.004798438 | flgJ | NZ_CP008957.1 | EDL933_RS08040 | EDL933_1658 | flagellar assembly peptidoglycan hydrolase FlgJ |
| gene-EDL933_RS08035 | 2.253802804 | 1.172361293 | 6.94E-07 | 4.92E-05 | EDL933_RS08035 | NZ_CP008957.1 | EDL933_RS08035 | EDL933_1657 | flagellar basal body P-ring protein FlgI |
| gene-EDL933_RS25500 | 2.203139394 | 1.139560778 | 0.000119487 | 0.004299761 | fadA | NZ_CP008957.1 | EDL933_RS25500 | EDL933_5165 | acetyl-CoA C-acyltransferase FadA |
| gene-EDL933_RS11950 | 2.197781979 | 1.136048277 | 9.16E-12 | 1.30E-09 | espM1 | NZ_CP008957.1 | EDL933_RS11950 | EDL933_2442 | T3SS effector guanine nucleotide exchange factor EspM1 |
| gene-EDL933_RS23930 | 2.171974192 | 1.119006961 | 0.000365123 | 0.01053492 | lldP | NZ_CP008957.1 | EDL933_RS23930 | EDL933_4867 | L-lactate permease |
| gene-EDL933_RS23935 | 2.152642951 | 1.106109046 | 0.000350309 | 0.010286705 | lldR | NZ_CP008957.1 | EDL933_RS23935 | EDL933_4868 | transcriptional regulator LldR |
| gene-EDL933_RS26615 | 2.143585736 | 1.100026121 | 0.000144946 | 0.005035639 | malE | NZ_CP008957.1 | EDL933_RS26615 | EDL933_5371 | maltose/maltodextrin ABC transporter substrate-binding protein MalE |
| gene-EDL933_RS16230 | 2.141722007 | 1.098771232 | 5.74E-12 | 8.89E-10 | mglB | NZ_CP008957.1 | EDL933_RS16230 | EDL933_3312 | galactose/glucose ABC transporter substrate-binding protein MglB |
| gene-EDL933_RS14420 | 2.096258208 | 1.067816432 | 4.36E-05 | 0.001904372 | fliG | NZ_CP008957.1 | EDL933_RS14420 | EDL933_2947 | flagellar motor switch protein FliG |
| gene-EDL933_RS14430 | 2.05641897 | 1.040134226 | 0.000288828 | 0.008939669 | fliI | NZ_CP008957.1 | EDL933_RS14430 | EDL933_2949 | flagellum-specific ATP synthase FliI |
| gene-EDL933_RS14335 | 2.054674304 | 1.038909724 | 1.33E-10 | 1.65E-08 | EDL933_RS14335 | NZ_CP008957.1 | EDL933_RS14335 | EDL933_2931 | FliC/FljB family flagellin |
| gene-EDL933_RS19525 | 2.046693692 | 1.033295205 | 3.10E-05 | 0.001431862 | fucP | NZ_CP008957.1 | EDL933_RS19525 | EDL933_3982 | L-fucose:H+ symporter permease |
| gene-EDL933_RS20010 | 2.03384753 | 1.02421153 | 3.36E-07 | 2.68E-05 | EDL933_RS20010 | NZ_CP008957.1 | EDL933_RS20010 | EDL933_4076 | EF2563 family selenium-dependent molybdenum hydroxylase system protein |
| gene-EDL933_RS10450 | 2.033185212 | 1.023741643 | 0.000446723 | 0.012398987 | EDL933_RS10450 | NZ_CP008957.1 | EDL933_RS10450 | EDL933_2136 | type 1 fimbrial protein |
| gene-EDL933_RS26610 | 2.009017298 | 1.006489986 | 0.000626654 | 0.016609734 | malF | NZ_CP008957.1 | EDL933_RS26610 | EDL933_5370 | maltose ABC transporter permease MalF |
| gene-EDL933_RS12905 | 0.480826884 | -1.056410533 | 0.001043189 | 0.023469453 | EDL933_RS12905 | NZ_CP008957.1 | EDL933_RS12905 | EDL933_2642 | hypothetical protein |
| gene-EDL933_RS26915 | 0.259615532 | -1.945551398 | 6.96E-19 | 2.73E-16 | EDL933_RS26915 | NZ_CP008957.1 | EDL933_RS26915 | EDL933_5436 | HAMP domain-containing protein |
| **Note: Chr, chromosome accession; pval, *P* value; padj, FDR adjusted *P* value.** | | | | |  |  |  |  |  |
