# Supplementary figures and images for: Genomic Island-Encoded Histidine Kinase and Response Regulator Coordinate Mannose Utilization with Virulence in Enterohemorrhagic Escherichia coli
Source: mBio. 2023 Feb 14;14(2):e03152-22. doi: 10.1128/mbio.03152-22 (PMC10128022; doi:10.1128/mbio.03152-22)

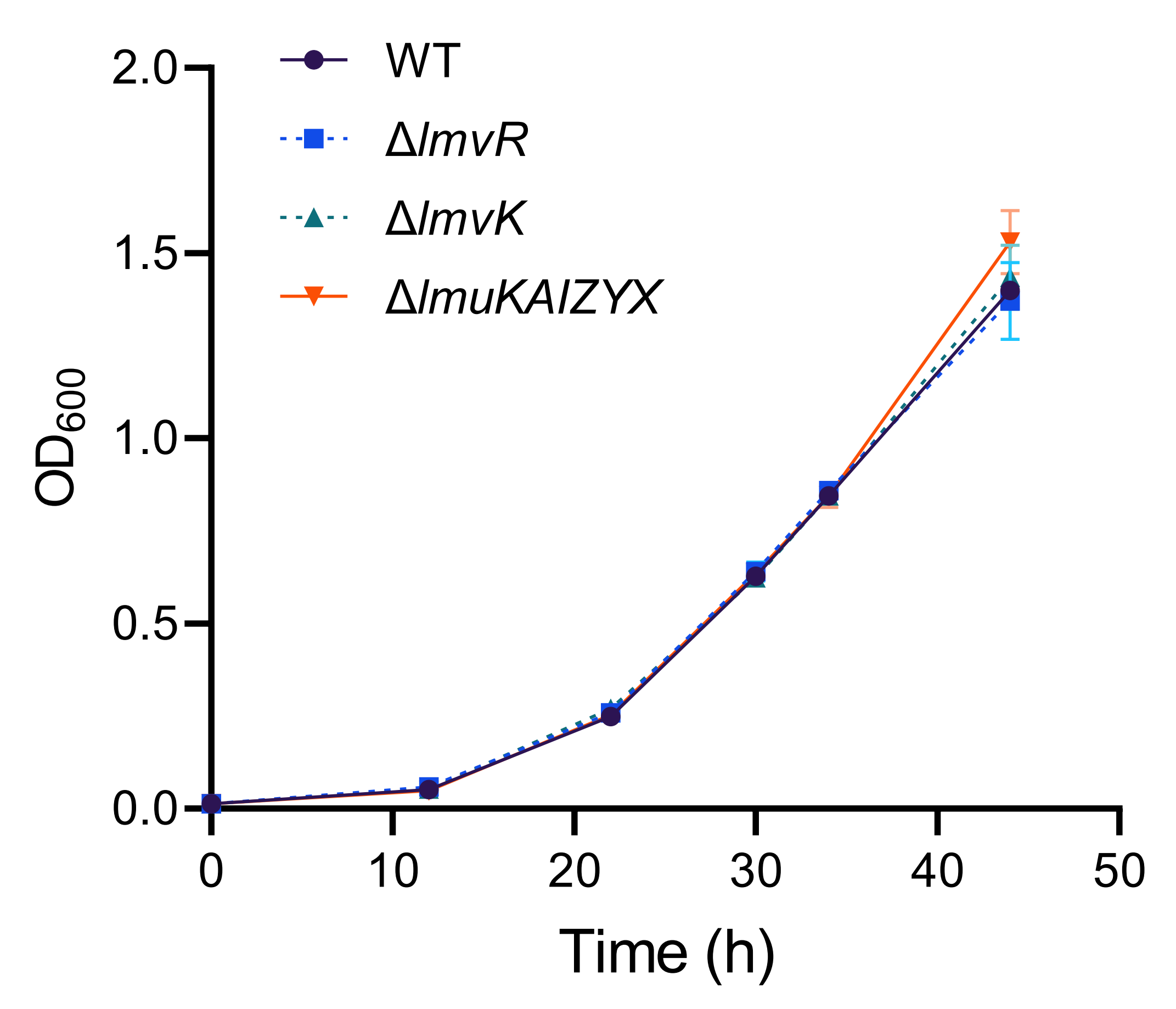

Supplement: FIG S1 [file mbio.03152-22-s0006.tif]

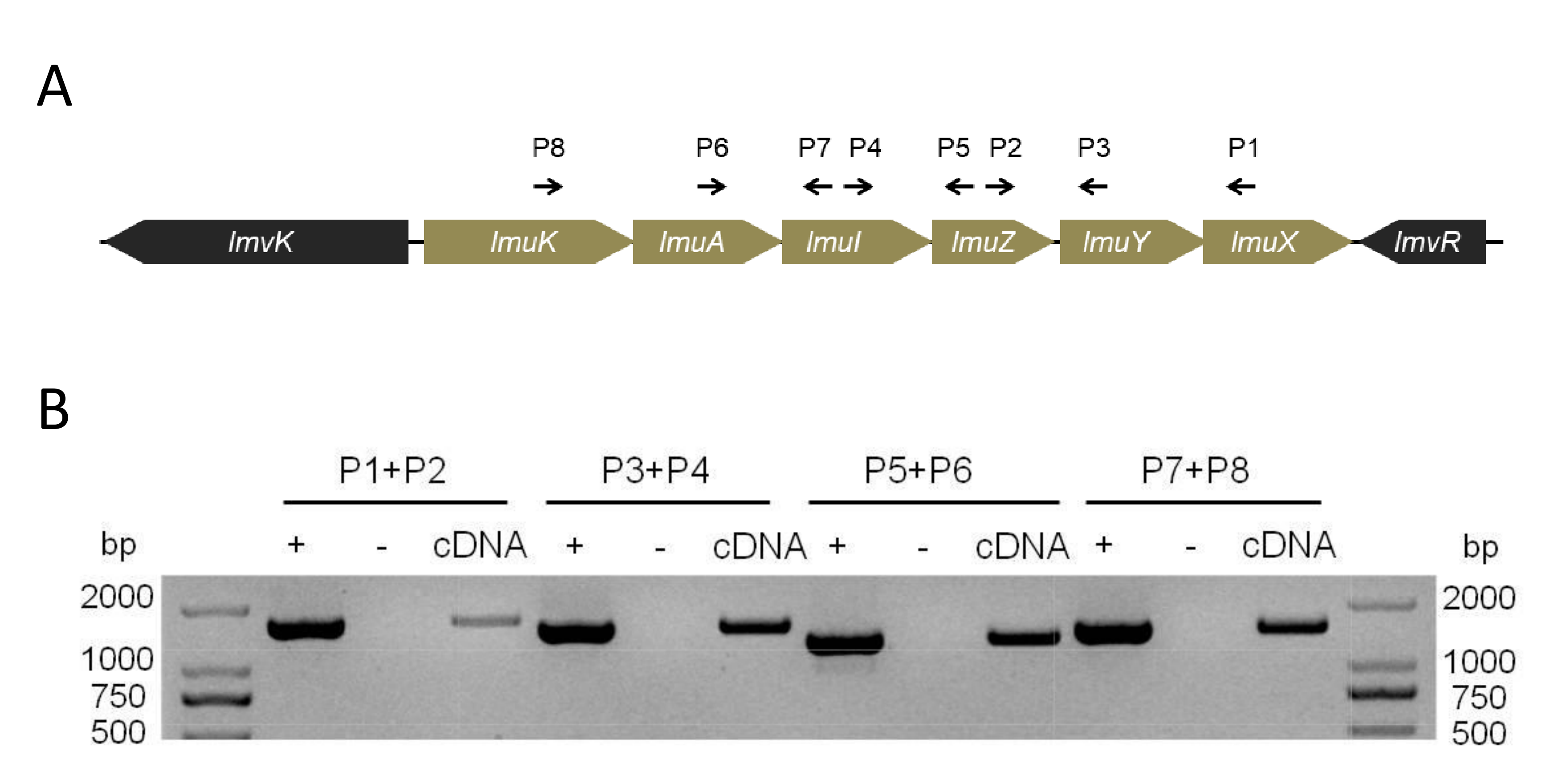

Supplement: FIG S2 [file mbio.03152-22-s0007.tif]

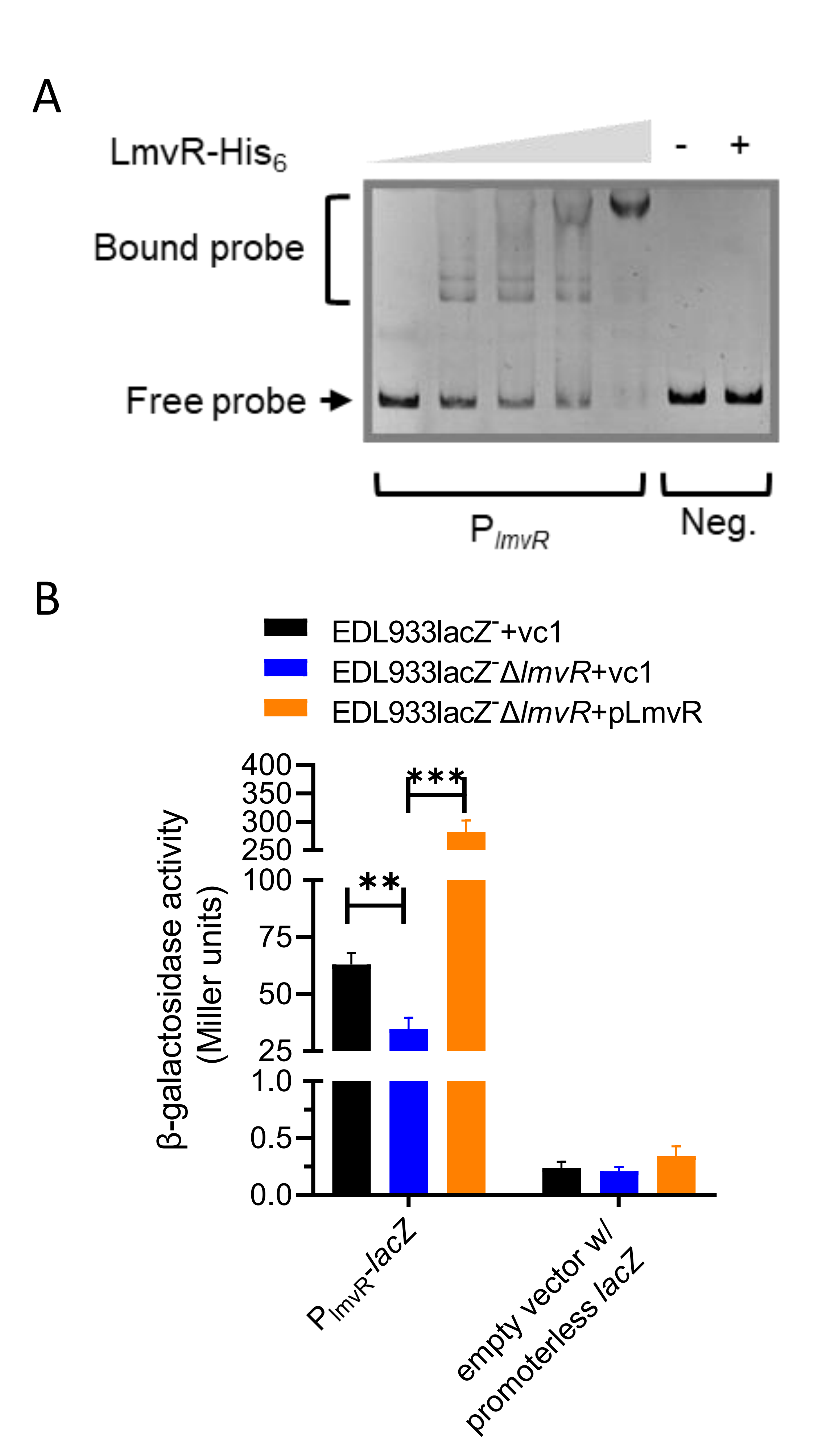

Supplement: FIG S3 [file mbio.03152-22-s0008.tif]

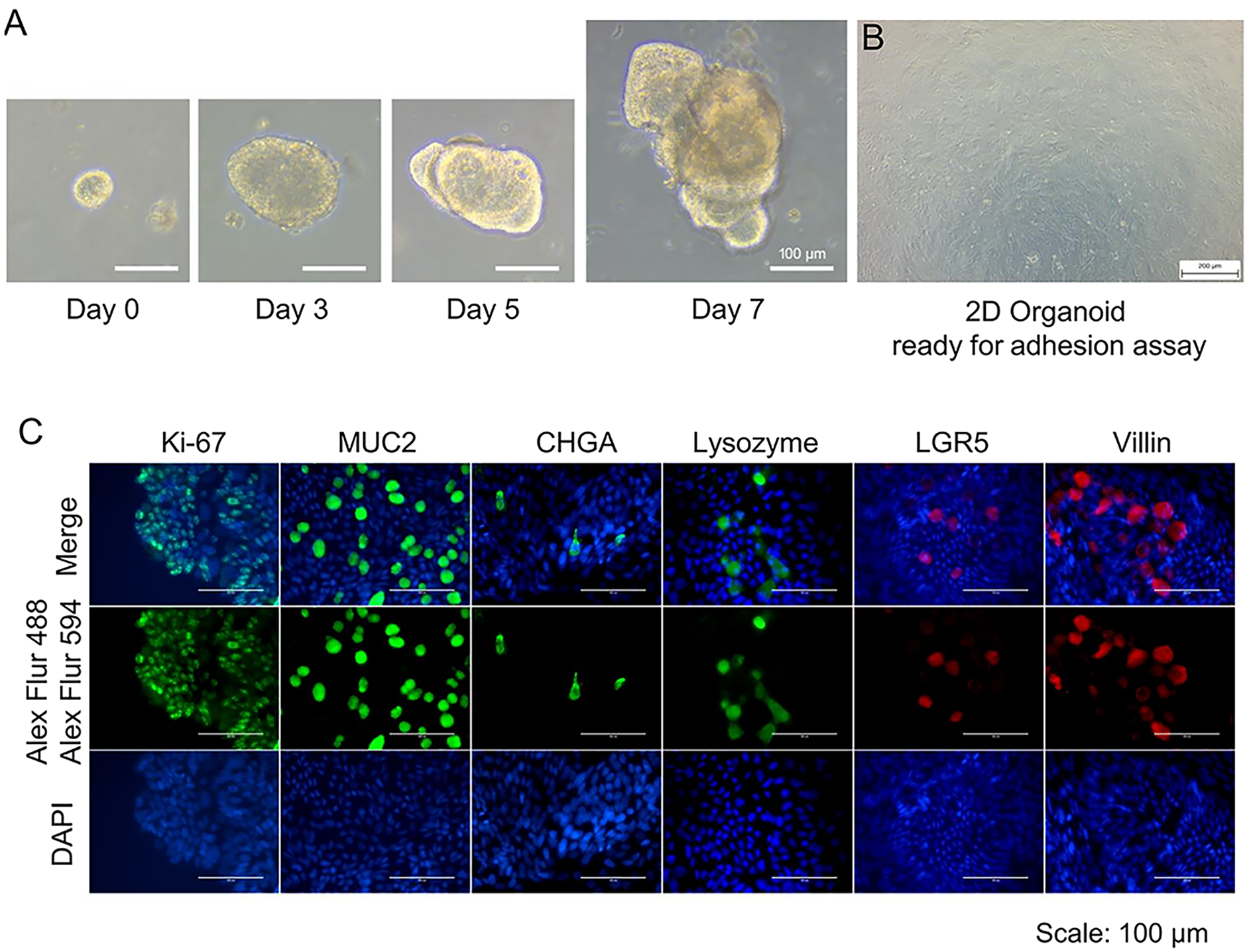

Supplement: FIG S4 [file mbio.03152-22-s0009.tif]

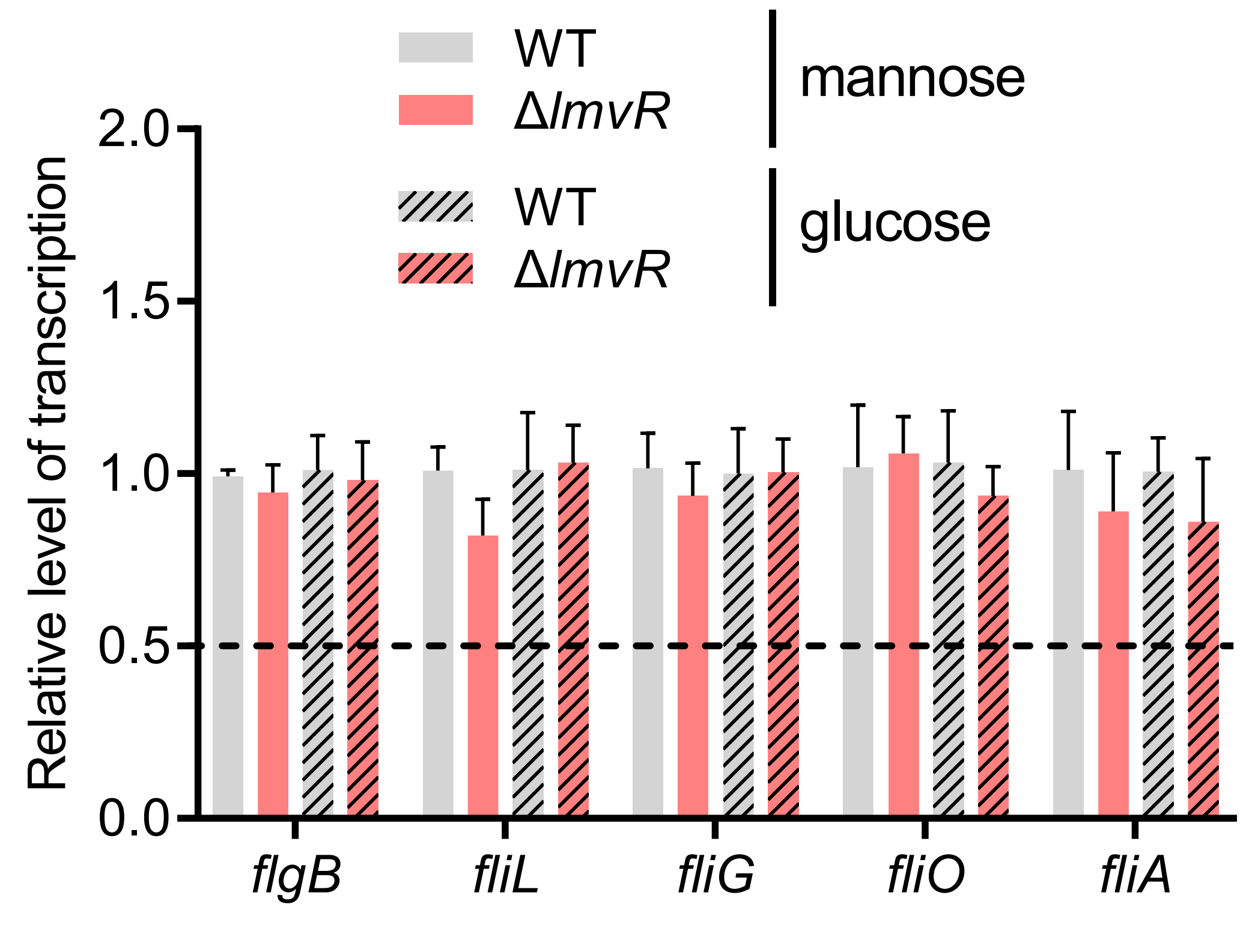

Supplement: FIG S5 [file mbio.03152-22-s0010.tif]
