# Supplementary material for: Genomic Island-Encoded Histidine Kinase and Response Regulator Coordinate Mannose Utilization with Virulence in Enterohemorrhagic Escherichia coli
Source: mBio. 2023 Feb 14;14(2):e03152-22. doi: 10.1128/mbio.03152-22 (PMC10128022; doi:10.1128/mbio.03152-22)
Supplement: TABLE S4 [file mbio.03152-22-s0004.docx]

| **Table S4. ChIP-seq analysis of RR LmvR during bacterial growth in DMEM and LB.** | | | | | | | | | | | | |
| --- | --- | --- | --- | --- | --- | --- | --- | --- | --- | --- | --- | --- |
| **In DMEM** | | | | | | | | | | | | |
| chr | start | end | length | location of peak summit | pileup reads | p value (-log10) | fold_enrichment | q value (-log10) | name | GeneFeature | GeneInside | Gene |
| NZ_CP008957.1 | 4474301 | 4481148 | 6848 | 4479181 | 2226 | 744.07623 | 4.73164 | 740.3902 | IP_DMC_vs_In_DMC_peak_393 | EDL933_RS23375:Promoter;EDL933_RS23370:Promoter;EDL933_RS23360:Promoter | EDL933_RS23355:NZ_CP008957.1:4476010-4477039:-:-685 | EDL933_RS23355(chuS) |
| NZ_CP008957.1 | 3734035 | 3735257 | 1223 | 3734400 | 2002 | 630.57062 | 4.46895 | 627.00806 | IP_DMC_vs_In_DMC_peak_309 | EDL933_RS19465:Promoter;EDL933_RS19495:Promoter;EDL933_RS19475:Promoter;EDL933_RS19470:Promoter;EDL933_RS19490:Promoter | EDL933_RS19475:NZ_CP008957.1:3733809-3734139:-:-506 | EDL933_RS19475(EDL933_RS19475) |
| NZ_CP008957.1 | 4148028 | 4148748 | 721 | 4148393 | 1773 | 522.64716 | 4.20504 | 519.21252 | IP_DMC_vs_In_DMC_peak_351 | EDL933_RS21600:exon;EDL933_RS21605:Promoter;EDL933_RS21595:Promoter;EDL933_RS21590:Promoter | EDL933_RS21600:NZ_CP008957.1:4148337-4148414:-:26 | EDL933_RS21600(EDL933_RS21600) |
| NZ_CP008957.1 | 1641589 | 1642008 | 420 | 1641810 | 1507 | 436.39792 | 4.13544 | 433.03638 | IP_DMC_vs_In_DMC_peak_159 | EDL933_RS08375:Promoter;EDL933_RS08390:Promoter;EDL933_RS30185:Promoter | EDL933_RS30185:NZ_CP008957.1:1642006-1642369:+:-208 | EDL933_RS30185(EDL933_RS30185) |
| NZ_CP008957.1 | 4554890 | 4556105 | 1216 | 4555198 | 1979 | 539.30579 | 3.93191 | 535.85474 | IP_DMC_vs_In_DMC_peak_405 | EDL933_RS23705:Promoter;EDL933_RS23695:Promoter | EDL933_RS23705:NZ_CP008957.1:4555246-4555459:+:251 | EDL933_RS23705(cspA) |
| NZ_CP008957.1 | 4387588 | 4388112 | 525 | 4387857 | 1611 | 382.01358 | 3.5146 | 378.7023 | IP_DMC_vs_In_DMC_peak_381 | EDL933_RS22890:Promoter;EDL933_RS22885:Promoter;EDL933_RS22870:Promoter;EDL933_RS22875:Promoter | EDL933_RS22875:NZ_CP008957.1:4386648-4387686:-:-163 | EDL933_RS22875(EDL933_RS22875) |
| NZ_CP008957.1 | 4993195 | 4994124 | 930 | 4993534 | 1514 | 335.33325 | 3.33976 | 332.06903 | IP_DMC_vs_In_DMC_peak_474 | EDL933_RS25905:Promoter;EDL933_RS25900:CDS;EDL933_RS25895:Promoter;EDL933_RS25910:Promoter;EDL933_RS25890:Promoter | EDL933_RS25900:NZ_CP008957.1:4993405-4993906:+:254 | EDL933_RS25900(cpxP) |
| NZ_CP008957.1 | 3880110 | 3881198 | 1089 | 3880812 | 1302 | 251.91565 | 3.0384 | 248.78528 | IP_DMC_vs_In_DMC_peak_326 | EDL933_RS20190:Promoter;EDL933_RS29275:exon;EDL933_RS20175:Promoter;EDL933_RS20180:Promoter | EDL933_RS29275:NZ_CP008957.1:3880737-3880921:+:-83 | EDL933_RS29275(ssrS) |
| NZ_CP008957.1 | 301596 | 302089 | 494 | 301860 | 1234 | 224.27539 | 2.9167 | 221.20703 | IP_DMC_vs_In_DMC_peak_39 | EDL933_RS01450:Promoter;EDL933_RS01445:Promoter;EDL933_RS01425:Promoter;EDL933_RS01420:Promoter;EDL933_RS01430:Promoter;EDL933_RS01440:Promoter | EDL933_RS01430:NZ_CP008957.1:301422-301812:-:-30 | EDL933_RS01430(EDL933_RS01430) |
| NZ_CP008957.1 | 1315225 | 1317411 | 2187 | 1316506 | 1070 | 194.41241 | 2.91295 | 191.48329 | IP_DMC_vs_In_DMC_peak_135 | EDL933_RS06540:Promoter;EDL933_RS29850:Promoter;EDL933_RS29845:Promoter;EDL933_RS06535:Promoter;EDL933_RS06510:Promoter;EDL933_RS06515:Promoter;EDL933_RS06525:Promoter;EDL933_RS06520:Promoter | EDL933_RS06525:NZ_CP008957.1:1316041-1316347:-:29 | EDL933_RS06525(EDL933_RS06525) |
| NZ_CP008957.1 | 3432001 | 3432730 | 730 | 3432301 | 1061 | 192.73663 | 2.91236 | 189.81656 | IP_DMC_vs_In_DMC_peak_265 | EDL933_RS34045:CDS;EDL933_RS17960:Promoter;EDL933_RS17970:Promoter;EDL933_RS17950:Promoter;EDL933_RS17965:Promoter | EDL933_RS17960:NZ_CP008957.1:3432322-3432514:+:43 | EDL933_RS17960(EDL933_RS17960) |
| NZ_CP008957.1 | 5063450 | 5065048 | 1599 | 5063757 | 1355 | 235.60168 | 2.83901 | 232.51151 | IP_DMC_vs_In_DMC_peak_480 | EDL933_RS26235:Promoter;EDL933_RS26230:exon;EDL933_RS26240:Promoter | EDL933_RS26230:NZ_CP008957.1:5063552-5065094:+:696 | EDL933_RS26230(EDL933_RS26230) |
| NZ_CP008957.1 | 3562286 | 3563046 | 761 | 3562544 | 1157 | 200.88303 | 2.8333 | 197.91583 | IP_DMC_vs_In_DMC_peak_285 | EDL933_RS18575:Promoter;EDL933_RS18560:Promoter;EDL933_RS18565:Promoter;EDL933_RS29255:exon | EDL933_RS29255:NZ_CP008957.1:3562490-3562853:+:175 | EDL933_RS29255(ssrA) |
| NZ_CP008957.1 | 5233457 | 5235756 | 2300 | 5233697 | 1401 | 239.76926 | 2.81222 | 236.66978 | IP_DMC_vs_In_DMC_peak_498 | EDL933_RS27015:Promoter;EDL933_RS27020:Promoter | EDL933_RS27015:NZ_CP008957.1:5233816-5235319:+:790 | EDL933_RS27015(proP) |
| NZ_CP008957.1 | 3543730 | 3544797 | 1068 | 3544168 | 1168 | 200.00864 | 2.80964 | 197.04712 | IP_DMC_vs_In_DMC_peak_279 | EDL933_RS18450:Promoter;EDL933_RS18470:Promoter;EDL933_RS18465:CDS;EDL933_RS33705:Promoter | EDL933_RS18465:NZ_CP008957.1:3544161-3544503:+:102 | EDL933_RS18465(raiA) |
| NZ_CP008957.1 | 5104490 | 5106078 | 1589 | 5104821 | 1304 | 221.48874 | 2.79802 | 218.42824 | IP_DMC_vs_In_DMC_peak_488 | EDL933_RS26435:Promoter;EDL933_RS26440:Promoter;EDL933_RS26430:exon;EDL933_RS26425:Promoter | EDL933_RS26430:NZ_CP008957.1:5104578-5106120:+:705 | EDL933_RS26430(EDL933_RS26430) |
| NZ_CP008957.1 | 4151996 | 4152879 | 884 | 4152611 | 1261 | 209.5531 | 2.76083 | 206.53755 | IP_DMC_vs_In_DMC_peak_352 | EDL933_RS21620:CDS;EDL933_RS21615:Promoter;EDL933_RS21610:Promoter | EDL933_RS21615:NZ_CP008957.1:4152196-4152283:-:-154 | EDL933_RS21615(EDL933_RS21615) |
| NZ_CP008957.1 | 4919187 | 4920766 | 1580 | 4919666 | 1295 | 205.47661 | 2.68725 | 202.48094 | IP_DMC_vs_In_DMC_peak_466 | EDL933_RS25540:Promoter;EDL933_RS25545:Promoter;EDL933_RS25535:Promoter;EDL933_RS25530:exon | EDL933_RS25530:NZ_CP008957.1:4919279-4920821:+:697 | EDL933_RS25530(EDL933_RS25530) |
| NZ_CP008957.1 | 226998 | 228612 | 1615 | 227488 | 1321 | 209.54994 | 2.68723 | 206.53447 | IP_DMC_vs_In_DMC_peak_29 | EDL933_RS01055:Promoter;EDL933_RS01050:Promoter;EDL933_RS01045:Promoter;EDL933_RS01030:Promoter;EDL933_RS01040:exon | EDL933_RS01040:NZ_CP008957.1:227102-228644:+:702 | EDL933_RS01040(EDL933_RS01040) |
| NZ_CP008957.1 | 4494204 | 4494968 | 765 | 4494666 | 1168 | 184.97678 | 2.68234 | 182.10835 | IP_DMC_vs_In_DMC_peak_398 | EDL933_RS23440:Promoter | EDL933_RS23440:NZ_CP008957.1:4493648-4494377:-:-208 | EDL933_RS23440(gadW) |
| NZ_CP008957.1 | 4863220 | 4863644 | 425 | 4863484 | 1088 | 172.28307 | 2.68064 | 169.48882 | IP_DMC_vs_In_DMC_peak_460 | EDL933_RS25265:Promoter;EDL933_RS25270:Promoter;EDL933_RS25250:Promoter;EDL933_RS25245:Promoter;EDL933_RS25260:Promoter | EDL933_RS25240:NZ_CP008957.1:4863405-4863482:+:26 | EDL933_RS25240(EDL933_RS25240) |
| NZ_CP008957.1 | 3536661 | 3538280 | 1620 | 3537799 | 1306 | 204.25043 | 2.66513 | 201.26285 | IP_DMC_vs_In_DMC_peak_277 | EDL933_RS18435:exon;EDL933_RS18430:Promoter;EDL933_RS18425:Promoter | EDL933_RS18435:NZ_CP008957.1:3536623-3538165:-:695 | EDL933_RS18435(EDL933_RS18435) |
| NZ_CP008957.1 | 4846427 | 4847631 | 1205 | 4847285 | 1386 | 213.96571 | 2.64648 | 210.93637 | IP_DMC_vs_In_DMC_peak_459 | EDL933_RS25165:Promoter;EDL933_RS25150:Promoter;EDL933_RS25175:Promoter;EDL933_RS25160:CDS | EDL933_RS25160:NZ_CP008957.1:4847261-4847363:+:-232 | EDL933_RS25160(rhoL) |
| NZ_CP008957.1 | 4822767 | 4824379 | 1613 | 4823082 | 1334 | 205.54362 | 2.64293 | 202.54781 | IP_DMC_vs_In_DMC_peak_454 | EDL933_RS25045:exon;EDL933_RS25040:Promoter;EDL933_RS25055:Promoter;EDL933_RS25050:Promoter;EDL933_RS25035:Promoter | EDL933_RS25045:NZ_CP008957.1:4822875-4824417:+:697 | EDL933_RS25045(EDL933_RS25045) |
| NZ_CP008957.1 | 4137893 | 4139110 | 1218 | 4138687 | 1234 | 189.28551 | 2.63481 | 186.38766 | IP_DMC_vs_In_DMC_peak_349 | EDL933_RS21555:Promoter;EDL933_RS32785:Promoter;EDL933_RS21560:CDS | EDL933_RS32785:NZ_CP008957.1:4137976-4138057:-:-444 | EDL933_RS32785(yrbN) |
| NZ_CP008957.1 | 1420491 | 1423195 | 2705 | 1421831 | 1014 | 153.85999 | 2.6147 | 151.18301 | IP_DMC_vs_In_DMC_peak_147 | EDL933_RS07165:Promoter;EDL933_RS07160:Promoter;EDL933_RS07155:Promoter | EDL933_RS07155:NZ_CP008957.1:1419243-1421667:-:-175 | EDL933_RS07155(pgaA) |
| NZ_CP008957.1 | 976645 | 977316 | 672 | 976964 | 1078 | 160.50542 | 2.58851 | 157.7905 | IP_DMC_vs_In_DMC_peak_107 | EDL933_RS04690:Promoter;EDL933_RS04675:Promoter;EDL933_RS04685:Promoter;EDL933_RS04680:CDS | EDL933_RS04680:NZ_CP008957.1:976945-977074:-:94 | EDL933_RS04680(mntS) |
| NZ_CP008957.1 | 1857298 | 1857652 | 355 | 1857482 | 941 | 139.89046 | 2.58328 | 137.2988 | IP_DMC_vs_In_DMC_peak_170 | EDL933_RS33370:Promoter;EDL933_RS09675:Promoter;EDL933_RS09680:Promoter;EDL933_RS09685:Promoter | EDL933_RS33370:NZ_CP008957.1:1857680-1857878:+:-205 | EDL933_RS33370(EDL933_RS33370) |
| NZ_CP008957.1 | 1387860 | 1388348 | 489 | 1388118 | 1213 | 179.16187 | 2.57935 | 176.32698 | IP_DMC_vs_In_DMC_peak_142 | EDL933_RS07010:CDS | EDL933_RS07020:NZ_CP008957.1:1391478-1391823:-:3719 | EDL933_RS07020(EDL933_RS07020) |
| NZ_CP008957.1 | 4933297 | 4933758 | 462 | 4933515 | 1237 | 179.23077 | 2.55265 | 176.39552 | IP_DMC_vs_In_DMC_peak_468 | EDL933_RS25605:Promoter | EDL933_RS25600:NZ_CP008957.1:4933711-4934344:-:817 | EDL933_RS25600(EDL933_RS25600) |
| NZ_CP008957.1 | 2708387 | 2709013 | 627 | 2708597 | 928 | 133.18369 | 2.53353 | 130.64938 | IP_DMC_vs_In_DMC_peak_199 | EDL933_RS33550:Promoter;EDL933_RS14255:exon;EDL933_RS33555:Promoter;EDL933_RS14250:Promoter | EDL933_RS14260:NZ_CP008957.1:2708647-2708721:-:21 | EDL933_RS14260(EDL933_RS14260) |
| NZ_CP008957.1 | 5511707 | 5513712 | 2006 | 5512814 | 1161 | 162.70644 | 2.50598 | 159.97932 | IP_DMC_vs_In_DMC_peak_526 | EDL933_RS28330:Promoter;EDL933_RS28310:CDS;EDL933_RS28315:Promoter | EDL933_RS28310:NZ_CP008957.1:5512106-5512895:-:186 | EDL933_RS28310(fhuF) |
| NZ_CP008957.1 | 4867476 | 4868064 | 589 | 4867745 | 1019 | 132.65642 | 2.40893 | 130.12679 | IP_DMC_vs_In_DMC_peak_461 | EDL933_RS25275:Promoter | EDL933_RS25275:NZ_CP008957.1:4865617-4867273:-:-496 | EDL933_RS25275(aslA) |
| NZ_CP008957.1 | 3612087 | 3617241 | 5155 | 3612364 | 1004 | 130.47852 | 2.40652 | 127.96788 | IP_DMC_vs_In_DMC_peak_301 | EDL933_RS18860:Promoter;EDL933_RS18845:Promoter;EDL933_RS18855:Promoter;EDL933_RS18865:Promoter | EDL933_RS18870:NZ_CP008957.1:3615272-3616232:+:-608 | EDL933_RS18870(nrdF) |
| NZ_CP008957.1 | 3436872 | 3437464 | 593 | 3437217 | 970 | 123.66089 | 2.38253 | 121.20857 | IP_DMC_vs_In_DMC_peak_266 | EDL933_RS17985:Promoter;EDL933_RS17980:Promoter;EDL933_RS17975:Promoter | EDL933_RS17980:NZ_CP008957.1:3435636-3437103:-:-64 | EDL933_RS17980(guaB) |
| NZ_CP008957.1 | 88036 | 88831 | 796 | 88306 | 949 | 120.38582 | 2.37616 | 117.96182 | IP_DMC_vs_In_DMC_peak_11 | EDL933_RS00420:Promoter;EDL933_RS00400:Promoter;EDL933_RS00410:CDS;EDL933_RS00415:Promoter;EDL933_RS00405:Promoter | EDL933_RS00410:NZ_CP008957.1:88229-88316:-:-117 | EDL933_RS00410(leuL) |
| NZ_CP008957.1 | 5071861 | 5072619 | 759 | 5072341 | 1061 | 134.27583 | 2.37561 | 131.73087 | IP_DMC_vs_In_DMC_peak_482 | EDL933_RS26290:Promoter;EDL933_RS26280:exon;EDL933_RS26260:Promoter;EDL933_RS26285:Promoter;EDL933_RS26295:Promoter | EDL933_RS26280:NZ_CP008957.1:5072315-5072390:+:-75 | EDL933_RS26280(EDL933_RS26280) |
| NZ_CP008957.1 | 4485375 | 4488963 | 3589 | 4487651 | 1049 | 132.53883 | 2.37344 | 130.01038 | IP_DMC_vs_In_DMC_peak_397 | EDL933_RS23430:Promoter;EDL933_RS23410:Promoter;EDL933_RS23405:Promoter;EDL933_RS23420:Promoter;EDL933_RS23400:Promoter | EDL933_RS23415:NZ_CP008957.1:4486753-4487326:+:415 | EDL933_RS23415(hdeD) |
| NZ_CP008957.1 | 5215014 | 5215919 | 906 | 5215275 | 1031 | 130.0657 | 2.37129 | 127.55835 | IP_DMC_vs_In_DMC_peak_497 | EDL933_RS26905:Promoter;EDL933_RS26910:Promoter;EDL933_RS26915:Promoter | EDL933_RS26915:NZ_CP008957.1:5215318-5217589:+:148 | EDL933_RS26915(EDL933_RS26915) |
| NZ_CP008957.1 | 1960868 | 1961279 | 412 | 1961143 | 861 | 108.29508 | 2.36389 | 105.96835 | IP_DMC_vs_In_DMC_peak_175 | EDL933_RS10360:Promoter;EDL933_RS10350:Promoter | EDL933_RS10350:NZ_CP008957.1:1961192-1962644:+:-119 | EDL933_RS10350(uxaB) |
| NZ_CP008957.1 | 3674619 | 3676503 | 1885 | 3676140 | 889 | 111.14284 | 2.35752 | 108.79568 | IP_DMC_vs_In_DMC_peak_305 | EDL933_RS19195:Promoter;EDL933_RS19200:CDS | EDL933_RS19195:NZ_CP008957.1:3674707-3675700:-:139 | EDL933_RS19195(rpoS) |
| NZ_CP008957.1 | 4250493 | 4251500 | 1008 | 4251027 | 1286 | 158.20932 | 2.34588 | 155.50696 | IP_DMC_vs_In_DMC_peak_366 | EDL933_RS22120:exon;EDL933_RS22110:Promoter;EDL933_RS22115:Promoter;EDL933_RS22125:Promoter;EDL933_RS22105:Promoter | EDL933_RS22120:NZ_CP008957.1:4249860-4251402:-:406 | EDL933_RS22120(EDL933_RS22120) |
| NZ_CP008957.1 | 4274078 | 4277675 | 3598 | 4277370 | 1111 | 134.42427 | 2.32439 | 131.87798 | IP_DMC_vs_In_DMC_peak_370 | EDL933_RS22350:Promoter;EDL933_RS22330:Promoter;EDL933_RS22335:Promoter;EDL933_RS22355:CDS | EDL933_RS22335:NZ_CP008957.1:4275597-4275909:-:33 | EDL933_RS22335(rpsJ) |
| NZ_CP008957.1 | 5073790 | 5078103 | 4314 | 5076299 | 1078 | 130.34619 | 2.3231 | 127.83668 | IP_DMC_vs_In_DMC_peak_483 | EDL933_RS26315:Promoter;EDL933_RS26325:Promoter;EDL933_RS26320:Promoter | EDL933_RS26310:NZ_CP008957.1:5075521-5076226:+:425 | EDL933_RS26310(rplA) |
| NZ_CP008957.1 | 3498251 | 3498771 | 521 | 3498569 | 897 | 108.09826 | 2.31561 | 105.77319 | IP_DMC_vs_In_DMC_peak_273 | EDL933_RS18240:Promoter;EDL933_RS18235:Promoter | EDL933_RS18240:NZ_CP008957.1:3496927-3498355:-:-155 | EDL933_RS18240(qseE) |
| NZ_CP008958.1 | 2386 | 3638 | 1253 | 2919 | 3384 | 386.36777 | 2.27338 | 383.05127 | IP_DMC_vs_In_DMC_peak_530 | EDL933_RS28585:Promoter;EDL933_RS28595:Promoter;EDL933_RS28590:Promoter | EDL933_RS28590:NZ_CP008958.1:3039-3897:+:-27 | EDL933_RS28590(repA) |
| NZ_CP008957.1 | 2188593 | 2189445 | 853 | 2189223 | 810 | 89.81431 | 2.22403 | 87.57875 | IP_DMC_vs_In_DMC_peak_177 | EDL933_RS11480:Promoter;EDL933_RS11470:Promoter;EDL933_RS11465:CDS | EDL933_RS11465:NZ_CP008957.1:2188709-2189462:+:309 | EDL933_RS11465(fnr) |
| NZ_CP008958.1 | 3886 | 4971 | 1086 | 4095 | 2904 | 313.80505 | 2.21544 | 310.57523 | IP_DMC_vs_In_DMC_peak_531 | EDL933_RS28600:Promoter;EDL933_RS28595:Promoter;EDL933_RS28605:Promoter | EDL933_RS28595:NZ_CP008958.1:4809-5094:+:-381 | EDL933_RS28595(EDL933_RS28595) |
| NZ_CP008958.1 | 47831 | 48695 | 865 | 48438 | 2982 | 317.47681 | 2.20084 | 314.2392 | IP_DMC_vs_In_DMC_peak_551 | intergenic | EDL933_RS28810:NZ_CP008958.1:47524-48265:+:738 | EDL933_RS28810(EDL933_RS28810) |
| NZ_CP008957.1 | 24955 | 25884 | 930 | 25535 | 864 | 93.45517 | 2.20042 | 91.20509 | IP_DMC_vs_In_DMC_peak_8 | EDL933_RS00120:Promoter;EDL933_RS00140:Promoter;EDL933_RS00125:Promoter;EDL933_RS00135:Promoter;EDL933_RS00130:Promoter | EDL933_RS00125:NZ_CP008957.1:25220-25484:-:65 | EDL933_RS00125(rpsT) |
| NZ_CP008957.1 | 2762856 | 2764292 | 1437 | 2764080 | 796 | 84.98045 | 2.18564 | 82.76459 | IP_DMC_vs_In_DMC_peak_202 | EDL933_RS30800:CDS | EDL933_RS30800:NZ_CP008957.1:2764013-2765027:+:-439 | EDL933_RS30800(tccP) |
| NZ_CP008957.1 | 4262168 | 4272952 | 10785 | 4270805 | 905 | 95.15701 | 2.17358 | 92.89951 | IP_DMC_vs_In_DMC_peak_368 | EDL933_RS22265:Promoter;EDL933_RS22260:Promoter;EDL933_RS22275:Promoter;EDL933_RS22280:Promoter;EDL933_RS22270:Promoter | EDL933_RS22240:NZ_CP008957.1:4267181-4267361:-:-198 | EDL933_RS22240(rpmD) |
| NZ_CP008957.1 | 4065432 | 4065948 | 517 | 4065683 | 912 | 91.64208 | 2.1309 | 89.39931 | IP_DMC_vs_In_DMC_peak_342 | EDL933_RS21175:Promoter;EDL933_RS21170:Promoter | EDL933_RS21170:NZ_CP008957.1:4065838-4066804:+:-148 | EDL933_RS21170(alx) |
| NZ_CP008957.1 | 1261025 | 1262021 | 997 | 1261885 | 776 | 78.26001 | 2.13079 | 76.06622 | IP_DMC_vs_In_DMC_peak_129 | EDL933_RS32105:Promoter;EDL933_RS06200:Promoter;EDL933_RS06185:Promoter;EDL933_RS06180:Promoter;EDL933_RS06195:Promoter | EDL933_RS06180:NZ_CP008957.1:1260641-1261415:-:-107 | EDL933_RS06180(EDL933_RS06180) |
| NZ_CP008957.1 | 4831184 | 4831659 | 476 | 4831345 | 970 | 95.34279 | 2.11197 | 93.08466 | IP_DMC_vs_In_DMC_peak_456 | EDL933_RS25095:Promoter;EDL933_RS25090:Promoter;EDL933_RS25085:Promoter;EDL933_RS25100:Promoter;EDL933_RS33875:Promoter | EDL933_RS25090:NZ_CP008957.1:4831390-4831489:+:31 | EDL933_RS25090(ilvL) |
| NZ_CP008958.1 | 49563 | 50003 | 441 | 49774 | 2825 | 271.29129 | 2.1041 | 268.13556 | IP_DMC_vs_In_DMC_peak_552 | EDL933_RS28815:Promoter | EDL933_RS28815:NZ_CP008958.1:48549-49527:-:-255 | EDL933_RS28815(EDL933_RS28815) |
| NZ_CP008957.1 | 5301494 | 5303850 | 2357 | 5301763 | 928 | 90.45399 | 2.10361 | 88.21606 | IP_DMC_vs_In_DMC_peak_504 | EDL933_RS27340:Promoter;EDL933_RS27350:Promoter;EDL933_RS27335:CDS;EDL933_RS27345:Promoter | EDL933_RS27345:NZ_CP008957.1:5302878-5303187:+:-206 | EDL933_RS27345(hfq) |
| NZ_CP008957.1 | 110891 | 112174 | 1284 | 111158 | 947 | 90.84211 | 2.08983 | 88.60262 | IP_DMC_vs_In_DMC_peak_14 | EDL933_RS00520:Promoter;EDL933_RS00525:Promoter;EDL933_RS00515:Promoter | EDL933_RS00515:NZ_CP008957.1:111162-112080:+:370 | EDL933_RS00515(lpxC) |
| NZ_CP008957.1 | 738215 | 738846 | 632 | 738455 | 762 | 73.35751 | 2.08843 | 71.1807 | IP_DMC_vs_In_DMC_peak_84 | EDL933_RS03405:Promoter;EDL933_RS03385:Promoter;EDL933_RS03395:CDS;EDL933_RS03410:Promoter | EDL933_RS03395:NZ_CP008957.1:738424-738634:+:106 | EDL933_RS03395(cspE) |
| NZ_CP008957.1 | 2619226 | 2619696 | 471 | 2619448 | 758 | 72.4019 | 2.08143 | 70.22824 | IP_DMC_vs_In_DMC_peak_192 | EDL933_RS13765:Promoter;EDL933_RS13740:Promoter | EDL933_RS13740:NZ_CP008957.1:2618605-2619262:-:-198 | EDL933_RS13740(pphA) |
| NZ_CP008957.1 | 3331440 | 3332086 | 647 | 3331798 | 754 | 71.12502 | 2.07046 | 68.95539 | IP_DMC_vs_In_DMC_peak_256 | EDL933_RS17460:Promoter;EDL933_RS17455:Promoter;EDL933_RS17445:Promoter | EDL933_RS17450:NZ_CP008957.1:3331828-3331904:-:141 | EDL933_RS17450(EDL933_RS17450) |
| NZ_CP008957.1 | 3132940 | 3134712 | 1773 | 3134031 | 750 | 69.8573 | 2.05949 | 67.69212 | IP_DMC_vs_In_DMC_peak_235 | EDL933_RS16575:CDS;EDL933_RS16570:Promoter;EDL933_RS16590:Promoter | EDL933_RS16575:NZ_CP008957.1:3132951-3134055:-:229 | EDL933_RS16575(ompC) |
| NZ_CP008957.1 | 3326031 | 3328053 | 2023 | 3327776 | 757 | 69.5715 | 2.04839 | 67.40734 | IP_DMC_vs_In_DMC_peak_255 | EDL933_RS17440:Promoter;EDL933_RS17435:CDS | EDL933_RS17435:NZ_CP008957.1:3326605-3327844:-:802 | EDL933_RS17435(EDL933_RS17435) |
| NZ_CP008957.1 | 3336234 | 3336805 | 572 | 3336451 | 744 | 67.97302 | 2.04304 | 65.81516 | IP_DMC_vs_In_DMC_peak_257 | EDL933_RS17490:Promoter;EDL933_RS17495:Promoter;EDL933_RS17515:Promoter;EDL933_RS17485:Promoter;EDL933_RS17475:Promoter;EDL933_RS17500:Promoter | EDL933_RS17485:NZ_CP008957.1:3336470-3336546:+:49 | EDL933_RS17485(EDL933_RS17485) |
| NZ_CP008957.1 | 3279772 | 3280152 | 381 | 3279973 | 743 | 67.661 | 2.0403 | 65.50443 | IP_DMC_vs_In_DMC_peak_246 | EDL933_RS17220:Promoter;EDL933_RS17205:Promoter;EDL933_RS17215:exon | EDL933_RS17215:NZ_CP008957.1:3279912-3279987:+:49 | EDL933_RS17215(EDL933_RS17215) |
| NZ_CP008957.1 | 1214198 | 1214538 | 341 | 1214352 | 742 | 67.34956 | 2.03755 | 65.19428 | IP_DMC_vs_In_DMC_peak_124 | EDL933_RS05895:Promoter;EDL933_RS05910:Promoter;EDL933_RS05900:Promoter;EDL933_RS05915:Promoter;EDL933_RS05905:Promoter;EDL933_RS05920:Promoter | EDL933_RS05905:NZ_CP008957.1:1213740-1214316:-:-51 | EDL933_RS05905(ssuE) |
| NZ_CP008957.1 | 973021 | 973418 | 398 | 973217 | 759 | 68.63198 | 2.03493 | 66.47151 | IP_DMC_vs_In_DMC_peak_105 | EDL933_RS04670:Promoter;EDL933_RS04660:Promoter;EDL933_RS33100:Promoter;EDL933_RS04650:Promoter | EDL933_RS04660:NZ_CP008957.1:972484-972988:-:-231 | EDL933_RS04660(dps) |
| NZ_CP008957.1 | 121 | 465 | 345 | 219 | 737 | 65.80111 | 2.02384 | 63.65227 | IP_DMC_vs_In_DMC_peak_1 | EDL933_RS00010:Promoter;EDL933_RS00005:CDS | EDL933_RS00010:NZ_CP008957.1:353-2816:+:-60 | EDL933_RS00010(thrA) |
| NZ_CP008957.1 | 4071695 | 4072280 | 586 | 4072064 | 847 | 75.09807 | 2.0212 | 72.91504 | IP_DMC_vs_In_DMC_peak_343 | EDL933_RS21200:Promoter;EDL933_RS21185:Promoter;EDL933_RS21195:Promoter;EDL933_RS21190:Promoter | EDL933_RS21190:NZ_CP008957.1:4070587-4072000:-:13 | EDL933_RS21190(uxaC) |
| NZ_CP008957.1 | 1247621 | 1248436 | 816 | 1248193 | 811 | 71.98594 | 2.0212 | 69.81384 | IP_DMC_vs_In_DMC_peak_127 | EDL933_RS06080:CDS;EDL933_RS06075:Promoter;EDL933_RS06065:Promoter | EDL933_RS06080:NZ_CP008957.1:1247561-1248221:-:193 | EDL933_RS06080(yccA) |
| NZ_CP008957.1 | 3629455 | 3631038 | 1584 | 3630115 | 738 | 64.06513 | 2.0013 | 61.92458 | IP_DMC_vs_In_DMC_peak_304 | EDL933_RS18950:Promoter;EDL933_RS18955:Promoter;EDL933_RS18930:Promoter;EDL933_RS18960:Promoter;EDL933_RS18925:Promoter;EDL933_RS18965:exon;EDL933_RS18935:Promoter;EDL933_RS18945:Promoter | EDL933_RS18965:NZ_CP008957.1:3630101-3630194:-:-52 | EDL933_RS18965(EDL933_RS18965) |
| NZ_CP008957.1 | 135739 | 136613 | 875 | 136008 | 814 | 69.83234 | 1.99413 | 67.6675 | IP_DMC_vs_In_DMC_peak_17 | EDL933_RS00620:Promoter;EDL933_RS00625:CDS | EDL933_RS00625:NZ_CP008957.1:135987-138585:+:188 | EDL933_RS00625(acnB) |
| NZ_CP008957.1 | 3463940 | 3469999 | 6060 | 3469575 | 729 | 60.32635 | 1.96401 | 58.20113 | IP_DMC_vs_In_DMC_peak_271 | EDL933_RS18110:Promoter;EDL933_RS18105:Promoter;EDL933_RS18125:Promoter;EDL933_RS18115:CDS | EDL933_RS18095:NZ_CP008957.1:3466497-3467013:-:44 | EDL933_RS18095(hscB) |
| NZ_CP008957.1 | 4037709 | 4038429 | 721 | 4037981 | 833 | 68.06024 | 1.9573 | 65.90206 | IP_DMC_vs_In_DMC_peak_340 | EDL933_RS21055:Promoter;EDL933_RS21050:CDS;EDL933_RS21045:Promoter | EDL933_RS21050:NZ_CP008957.1:4037938-4038154:+:130 | EDL933_RS21050(rpsU) |
| NZ_CP008957.1 | 2850084 | 2850622 | 539 | 2850387 | 765 | 61.87441 | 1.948 | 59.74232 | IP_DMC_vs_In_DMC_peak_207 | EDL933_RS15115:Promoter;EDL933_RS15110:Promoter;EDL933_RS15120:Promoter;EDL933_RS30885:CDS;EDL933_RS15125:Promoter | EDL933_RS30885:NZ_CP008957.1:2850368-2850419:+:-15 | EDL933_RS30885(hisL) |
| NZ_CP008957.1 | 4422385 | 4422809 | 425 | 4422578 | 819 | 65.72841 | 1.9437 | 63.58034 | IP_DMC_vs_In_DMC_peak_387 | EDL933_RS23085:Promoter;EDL933_RS23075:Promoter;EDL933_RS23090:Promoter;EDL933_RS23080:Promoter;EDL933_RS23070:CDS | EDL933_RS23070:NZ_CP008957.1:4422443-4423265:+:153 | EDL933_RS23070(EDL933_RS23070) |
| NZ_CP008957.1 | 4245917 | 4246460 | 544 | 4246024 | 13551 | 1024.16943 | 1.92169 | 1020.27252 | IP_DMC_vs_In_DMC_peak_362 | intergenic | EDL933_RS22090:NZ_CP008957.1:4246056-4246172:-:-16 | EDL933_RS22090(rrf) |
| NZ_CP008957.1 | 4281553 | 4282388 | 836 | 4282134 | 749 | 58.4458 | 1.92151 | 56.32945 | IP_DMC_vs_In_DMC_peak_374 | EDL933_RS22375:Promoter;EDL933_RS22365:Promoter;EDL933_RS22370:Promoter | EDL933_RS22375:NZ_CP008957.1:4281710-4282085:-:115 | EDL933_RS22375(rpsL) |
| NZ_CP008957.1 | 4642724 | 4643505 | 782 | 4643307 | 858 | 65.75845 | 1.91158 | 63.61021 | IP_DMC_vs_In_DMC_peak_415 | EDL933_RS24095:Promoter;EDL933_RS24075:Promoter;EDL933_RS24080:Promoter;EDL933_RS24085:Promoter | EDL933_RS24085:NZ_CP008957.1:4642920-4643157:-:43 | EDL933_RS24085(rpmB) |
| NZ_CP008957.1 | 1012298 | 1012598 | 301 | 1012453 | 727 | 55.83788 | 1.90966 | 53.73385 | IP_DMC_vs_In_DMC_peak_110 | EDL933_RS04835:Promoter;EDL933_RS04845:Promoter;EDL933_RS04840:Promoter | EDL933_RS04845:NZ_CP008957.1:1012480-1013017:+:-32 | EDL933_RS04845(rcdA) |
| NZ_CP008957.1 | 15308 | 17697 | 2390 | 16787 | 825 | 63.05394 | 1.90887 | 60.91735 | IP_DMC_vs_In_DMC_peak_3 | EDL933_RS00080:Promoter;EDL933_RS00085:CDS;EDL933_RS00090:Promoter | EDL933_RS00085:NZ_CP008957.1:16156-17323:+:346 | EDL933_RS00085(nhaA) |
| NZ_CP008957.1 | 545735 | 547099 | 1365 | 546750 | 752 | 57.51398 | 1.90743 | 55.40179 | IP_DMC_vs_In_DMC_peak_66 | EDL933_RS02645:CDS;EDL933_RS02630:Promoter;EDL933_RS02635:Promoter;EDL933_RS02640:Promoter | EDL933_RS02640:NZ_CP008957.1:546154-546373:-:-43 | EDL933_RS02640(hha) |
| NZ_CP008957.1 | 3310441 | 3311003 | 563 | 3310703 | 734 | 54.30014 | 1.88401 | 52.20388 | IP_DMC_vs_In_DMC_peak_253 | EDL933_RS17345:Promoter;EDL933_RS17360:Promoter;EDL933_RS17365:Promoter;EDL933_RS32655:Promoter | EDL933_RS17365:NZ_CP008957.1:3310782-3311703:+:-60 | EDL933_RS17365(lpxP) |
| NZ_CP008957.1 | 693738 | 697568 | 3831 | 694049 | 686 | 50.86118 | 1.88398 | 48.78523 | IP_DMC_vs_In_DMC_peak_77 | EDL933_RS03190:Promoter;EDL933_RS03200:Promoter;EDL933_RS03195:Promoter;EDL933_RS03205:Promoter | EDL933_RS03205:NZ_CP008957.1:695590-699472:+:62 | EDL933_RS03205(entF) |
| NZ_CP008957.1 | 2451384 | 2451863 | 480 | 2451640 | 685 | 50.58421 | 1.88124 | 48.50996 | IP_DMC_vs_In_DMC_peak_186 | EDL933_RS12865:CDS | EDL933_RS12865:NZ_CP008957.1:2451593-2451830:+:30 | EDL933_RS12865(lpp) |
| NZ_CP008957.1 | 776287 | 777208 | 922 | 776923 | 684 | 50.30787 | 1.8785 | 48.23536 | IP_DMC_vs_In_DMC_peak_87 | EDL933_RS03595:Promoter;EDL933_RS03615:exon;EDL933_RS03600:Promoter;EDL933_RS03610:Promoter;EDL933_RS03605:Promoter;EDL933_RS03590:Promoter | EDL933_RS03605:NZ_CP008957.1:776694-776769:-:22 | EDL933_RS03605(EDL933_RS03605) |
| NZ_CP008957.1 | 3824774 | 3825160 | 387 | 3824959 | 737 | 53.78585 | 1.87495 | 51.6922 | IP_DMC_vs_In_DMC_peak_323 | EDL933_RS19955:exon;EDL933_RS19940:Promoter;EDL933_RS19965:Promoter;EDL933_RS19945:Promoter | EDL933_RS19955:NZ_CP008957.1:3824939-3825013:-:46 | EDL933_RS19955(EDL933_RS19955) |
| NZ_CP008957.1 | 4934509 | 4934940 | 432 | 4934680 | 799 | 56.88745 | 1.86031 | 54.77826 | IP_DMC_vs_In_DMC_peak_469 | EDL933_RS25605:Promoter;EDL933_RS25600:Promoter;EDL933_RS25610:Promoter | EDL933_RS25605:NZ_CP008957.1:4934925-4935432:+:-201 | EDL933_RS25605(yihI) |
| NZ_CP008957.1 | 1394492 | 1395051 | 560 | 1394794 | 762 | 54.00055 | 1.85629 | 51.9057 | IP_DMC_vs_In_DMC_peak_144 | EDL933_RS07040:Promoter;EDL933_RS07035:Promoter;EDL933_RS07045:Promoter;EDL933_RS29915:Promoter | EDL933_RS07045:NZ_CP008957.1:1393948-1394578:-:-193 | EDL933_RS07045(EDL933_RS07045) |
| NZ_CP008957.1 | 4757259 | 4758695 | 1437 | 4758258 | 778 | 54.82071 | 1.85302 | 52.72168 | IP_DMC_vs_In_DMC_peak_436 | EDL933_RS24735:Promoter;EDL933_RS24745:Promoter;EDL933_RS24740:CDS;EDL933_RS24755:Promoter;EDL933_RS24750:Promoter | EDL933_RS33865:NZ_CP008957.1:4757882-4757963:+:94 | EDL933_RS33865(EDL933_RS33865) |
| NZ_CP008957.1 | 2493263 | 2495224 | 1962 | 2494157 | 672 | 47.04117 | 1.84559 | 44.98894 | IP_DMC_vs_In_DMC_peak_187 | EDL933_RS33520:Promoter;EDL933_RS13075:CDS;EDL933_RS13070:Promoter;EDL933_RS13060:Promoter | EDL933_RS13075:NZ_CP008957.1:2493975-2494173:-:-70 | EDL933_RS13075(rpmI) |
| NZ_CP008957.1 | 126392 | 133728 | 7337 | 126802 | 763 | 52.545 | 1.83795 | 50.45867 | IP_DMC_vs_In_DMC_peak_16 | EDL933_RS00600:CDS;EDL933_RS00595:Promoter;EDL933_RS00605:Promoter | EDL933_RS00610:NZ_CP008957.1:130184-132077:+:-124 | EDL933_RS00610(aceF) |
| NZ_CP008957.1 | 1144340 | 1144620 | 281 | 1144443 | 665 | 45.17815 | 1.82639 | 43.13932 | IP_DMC_vs_In_DMC_peak_115 | EDL933_RS05640:Promoter | EDL933_RS05640:NZ_CP008957.1:1144292-1144380:-:-99 | EDL933_RS05640(EDL933_RS05640) |
| NZ_CP008957.1 | 4514209 | 4514511 | 303 | 4514363 | 747 | 50.49156 | 1.8258 | 48.41805 | IP_DMC_vs_In_DMC_peak_400 | EDL933_RS23515:CDS;EDL933_RS23510:Promoter | EDL933_RS23515:NZ_CP008957.1:4513391-4515380:-:1020 | EDL933_RS23515(hmsP) |
| NZ_CP008957.1 | 3092903 | 3093323 | 421 | 3093111 | 692 | 45.33644 | 1.80501 | 43.2967 | IP_DMC_vs_In_DMC_peak_229 | EDL933_RS16380:Promoter;EDL933_RS16375:Promoter | EDL933_RS16375:NZ_CP008957.1:3093227-3093794:+:-114 | EDL933_RS16375(mepS) |
| NZ_CP008957.1 | 240333 | 241347 | 1015 | 240537 | 676 | 44.12039 | 1.8022 | 42.09072 | IP_DMC_vs_In_DMC_peak_32 | EDL933_RS01115:exon;EDL933_RS01105:Promoter;EDL933_RS01120:Promoter | EDL933_RS01120:NZ_CP008957.1:240886-241687:+:-46 | EDL933_RS01120(EDL933_RS01120) |
| NZ_CP008957.1 | 1199893 | 1200740 | 848 | 1200106 | 655 | 42.57185 | 1.79897 | 40.55337 | IP_DMC_vs_In_DMC_peak_122 | EDL933_RS05850:Promoter;EDL933_RS05845:CDS | EDL933_RS05845:NZ_CP008957.1:1200048-1200597:+:268 | EDL933_RS05845(EDL933_RS05845) |
| NZ_CP008957.1 | 1711727 | 1712038 | 312 | 1711882 | 652 | 41.80273 | 1.79074 | 39.78988 | IP_DMC_vs_In_DMC_peak_163 | EDL933_RS33315:Promoter;EDL933_RS08865:Promoter;EDL933_RS08870:Promoter | EDL933_RS08865:NZ_CP008957.1:1712165-1712471:+:-283 | EDL933_RS08865(EDL933_RS08865) |
| NZ_CP008958.1 | 15768 | 16482 | 715 | 15990 | 2543 | 157.47543 | 1.78905 | 154.77698 | IP_DMC_vs_In_DMC_peak_538 | EDL933_RS28665:Promoter;EDL933_RS32940:Promoter | EDL933_RS32940:NZ_CP008958.1:15399-15881:-:-243 | EDL933_RS32940(EDL933_RS32940) |
| NZ_CP008957.1 | 5208491 | 5209087 | 597 | 5208781 | 45586 | 2689.90942 | 1.7691 | 2685.24463 | IP_DMC_vs_In_DMC_peak_496 | EDL933_RS26875:Promoter;EDL933_RS26880:CDS | EDL933_RS26880:NZ_CP008957.1:5208438-5209146:+:350 | EDL933_RS26880(EDL933_RS26880) |
| NZ_CP008957.1 | 2871230 | 2875901 | 4672 | 2875637 | 644 | 39.7809 | 1.7688 | 37.78342 | IP_DMC_vs_In_DMC_peak_212 | EDL933_RS15225:Promoter;EDL933_RS15230:Promoter;EDL933_RS15235:Promoter | EDL933_RS15225:NZ_CP008957.1:2872903-2873650:-:85 | EDL933_RS15225(EDL933_RS15225) |
| NZ_CP008958.1 | 66348 | 66840 | 493 | 66597 | 2449 | 146.38036 | 1.76869 | 143.74286 | IP_DMC_vs_In_DMC_peak_557 | EDL933_RS28940:Promoter;EDL933_RS34250:Promoter;EDL933_RS28945:Promoter;EDL933_RS28935:Promoter | EDL933_RS28935:NZ_CP008958.1:66607-66838:+:-13 | EDL933_RS28935(EDL933_RS28935) |
| NZ_CP008957.1 | 4249947 | 4250275 | 329 | 4250142 | 1198 | 71.13028 | 1.75774 | 68.96063 | IP_DMC_vs_In_DMC_peak_365 | EDL933_RS22120:exon;EDL933_RS22110:Promoter;EDL933_RS22115:Promoter;EDL933_RS22125:Promoter;EDL933_RS22105:Promoter | EDL933_RS22115:NZ_CP008957.1:4249715-4249792:-:-318 | EDL933_RS22115(EDL933_RS22115) |
| NZ_CP008957.1 | 4162606 | 4163891 | 1286 | 4163437 | 696 | 41.84352 | 1.75525 | 39.83037 | IP_DMC_vs_In_DMC_peak_355 | EDL933_RS21685:Promoter;EDL933_RS21680:Promoter;EDL933_RS21675:Promoter;EDL933_RS21660:Promoter;EDL933_RS21670:Promoter;EDL933_RS21665:Promoter | EDL933_RS21670:NZ_CP008957.1:4162840-4163098:-:-150 | EDL933_RS21670(rpmA) |
| NZ_CP008957.1 | 829552 | 832090 | 2539 | 831136 | 642 | 38.66068 | 1.75435 | 36.67155 | IP_DMC_vs_In_DMC_peak_92 | EDL933_RS03865:Promoter;EDL933_RS03860:Promoter;EDL933_RS03870:Promoter;EDL933_RS03850:Promoter;EDL933_RS33070:CDS;EDL933_RS03845:Promoter | EDL933_RS03850:NZ_CP008957.1:829606-830890:-:69 | EDL933_RS03850(gltA) |
| NZ_CP008958.1 | 73736 | 74152 | 417 | 73942 | 21966 | 1262.84619 | 1.75434 | 1258.79907 | IP_DMC_vs_In_DMC_peak_558 | EDL933_RS28995:Promoter;EDL933_RS28990:Promoter | EDL933_RS28990:NZ_CP008958.1:73948-74107:+:-4 | EDL933_RS28990(EDL933_RS28990) |
| NZ_CP008957.1 | 1178549 | 1180016 | 1468 | 1178820 | 637 | 38.04688 | 1.74961 | 36.06289 | IP_DMC_vs_In_DMC_peak_120 | EDL933_RS05775:Promoter;EDL933_RS05765:CDS;EDL933_RS05770:Promoter | EDL933_RS05770:NZ_CP008957.1:1178969-1180643:+:313 | EDL933_RS05770(rpsA) |
| NZ_CP008957.1 | 1854246 | 1854470 | 225 | 1854396 | 634 | 37.31384 | 1.74138 | 35.33622 | IP_DMC_vs_In_DMC_peak_169 | EDL933_RS09655:Promoter;EDL933_RS09640:Promoter;EDL933_RS32300:Promoter;EDL933_RS09645:Promoter | EDL933_RS09640:NZ_CP008957.1:1854498-1854654:+:-140 | EDL933_RS09640(hokD) |
| NZ_CP008957.1 | 573035 | 574708 | 1674 | 574385 | 680 | 39.74884 | 1.73925 | 37.75188 | IP_DMC_vs_In_DMC_peak_70 | EDL933_RS02745:Promoter;EDL933_RS02750:Promoter | EDL933_RS02750:NZ_CP008957.1:573075-573870:-:-1 | EDL933_RS02750(EDL933_RS02750) |
| NZ_CP008957.1 | 4872109 | 4872770 | 662 | 4872442 | 777 | 44.06944 | 1.72582 | 42.04012 | IP_DMC_vs_In_DMC_peak_462 | EDL933_RS25295:Promoter;EDL933_RS25300:CDS;EDL933_RS25290:Promoter | EDL933_RS25300:NZ_CP008957.1:4872436-4874983:+:3 | EDL933_RS25300(cyaA) |
| NZ_CP008957.1 | 4407351 | 4408069 | 719 | 4407746 | 776 | 43.79412 | 1.72317 | 41.7668 | IP_DMC_vs_In_DMC_peak_386 | EDL933_RS22980:Promoter;EDL933_RS22985:Promoter | EDL933_RS22985:NZ_CP008957.1:4406799-4407654:-:-55 | EDL933_RS22985(rpoH) |
| NZ_CP008957.1 | 5307131 | 5307967 | 837 | 5307394 | 693 | 38.01931 | 1.7061 | 36.03584 | IP_DMC_vs_In_DMC_peak_507 | EDL933_RS27380:Promoter;EDL933_RS27375:Promoter;EDL933_RS27370:CDS | EDL933_RS27370:NZ_CP008957.1:5307278-5308577:+:270 | EDL933_RS27370(purA) |
| NZ_CP008957.1 | 4018052 | 4018526 | 475 | 4018247 | 675 | 36.92329 | 1.70402 | 34.94954 | IP_DMC_vs_In_DMC_peak_338 | EDL933_RS20945:Promoter;EDL933_RS20950:Promoter;EDL933_RS34015:Promoter;EDL933_RS20965:Promoter;EDL933_RS20960:Promoter | EDL933_RS20945:NZ_CP008957.1:4017533-4018187:-:-101 | EDL933_RS20945(ribB) |
| NZ_CP008957.1 | 3109269 | 3109659 | 391 | 3109472 | 619 | 33.74084 | 1.70025 | 31.79541 | IP_DMC_vs_In_DMC_peak_233 | EDL933_RS16445:exon | EDL933_RS16445:NZ_CP008957.1:3109459-3109536:+:4 | EDL933_RS16445(EDL933_RS16445) |
| NZ_CP008957.1 | 5526078 | 5526466 | 389 | 5526271 | 691 | 36.72436 | 1.68993 | 34.75226 | IP_DMC_vs_In_DMC_peak_527 | EDL933_RS28400:Promoter;EDL933_RS28405:Promoter | EDL933_RS28400:NZ_CP008957.1:5526316-5527540:+:-44 | EDL933_RS28400(deoB) |
| NZ_CP008957.1 | 4325250 | 4326495 | 1246 | 4325979 | 683 | 36.24003 | 1.68886 | 34.27205 | IP_DMC_vs_In_DMC_peak_379 | EDL933_RS22620:Promoter;EDL933_RS22625:Promoter | EDL933_RS22620:NZ_CP008957.1:4325409-4325970:-:98 | EDL933_RS22620(nudE) |
| NZ_CP008957.1 | 979794 | 980156 | 363 | 979980 | 627 | 32.10129 | 1.66938 | 30.17239 | IP_DMC_vs_In_DMC_peak_108 | EDL933_RS04700:Promoter;EDL933_RS04695:Promoter | EDL933_RS04700:NZ_CP008957.1:980038-981631:+:-63 | EDL933_RS04700(EDL933_RS04700) |
| NZ_CP008957.1 | 4246674 | 4248737 | 2064 | 4247030 | 1124 | 54.58826 | 1.65497 | 52.49041 | IP_DMC_vs_In_DMC_peak_363 | EDL933_RS22100:Promoter;EDL933_RS22095:Promoter;EDL933_RS22105:exon;EDL933_RS22090:Promoter | EDL933_RS22100:NZ_CP008957.1:4246301-4246417:-:-1288 | EDL933_RS22100(rrf) |
| NZ_CP008957.1 | 3012664 | 3014410 | 1747 | 3014185 | 602 | 29.88061 | 1.65363 | 27.97332 | IP_DMC_vs_In_DMC_peak_221 | EDL933_RS15870:Promoter;EDL933_RS15875:Promoter;EDL933_RS15880:Promoter | EDL933_RS15875:NZ_CP008957.1:3012808-3013078:-:-458 | EDL933_RS15875(EDL933_RS15875) |
| NZ_CP008957.1 | 5176915 | 5177332 | 418 | 5177110 | 692 | 33.98412 | 1.65176 | 32.03608 | IP_DMC_vs_In_DMC_peak_495 | EDL933_RS26755:Promoter;EDL933_RS26740:Promoter;EDL933_RS26750:Promoter | EDL933_RS26750:NZ_CP008957.1:5176772-5177054:-:-69 | EDL933_RS26750(EDL933_RS26750) |
| NZ_CP008957.1 | 4699419 | 4704037 | 4619 | 4701066 | 681 | 33.3297 | 1.64982 | 31.38825 | IP_DMC_vs_In_DMC_peak_427 | EDL933_RS24415:Promoter | EDL933_RS24405:NZ_CP008957.1:4701390-4701804:-:76 | EDL933_RS24405(grlA) |
| NZ_CP008957.1 | 1378831 | 1379346 | 516 | 1379041 | 783 | 37.83987 | 1.64678 | 35.85769 | IP_DMC_vs_In_DMC_peak_141 | EDL933_RS06980:Promoter;EDL933_RS06985:Promoter;EDL933_RS06995:Promoter;EDL933_RS06990:Promoter;EDL933_RS07000:Promoter | EDL933_RS06980:NZ_CP008957.1:1379142-1379283:+:-54 | EDL933_RS06980(EDL933_RS06980) |
| NZ_CP008957.1 | 1617080 | 1617435 | 356 | 1617288 | 598 | 29.00208 | 1.64266 | 27.10444 | IP_DMC_vs_In_DMC_peak_158 | EDL933_RS08200:Promoter;EDL933_RS08190:Promoter | EDL933_RS08200:NZ_CP008957.1:1616277-1617240:-:-17 | EDL933_RS08200(ldtC) |
| NZ_CP008957.1 | 5294714 | 5294982 | 269 | 5294893 | 630 | 30.22492 | 1.63889 | 28.3142 | IP_DMC_vs_In_DMC_peak_503 | EDL933_RS27290:Promoter;EDL933_RS27300:Promoter;EDL933_RS27310:Promoter;EDL933_RS33890:Promoter;EDL933_RS27320:Promoter;EDL933_RS27285:Promoter;EDL933_RS27305:Promoter | EDL933_RS27300:NZ_CP008957.1:5294950-5295026:+:-102 | EDL933_RS27300(EDL933_RS27300) |
| NZ_CP008957.1 | 974259 | 974679 | 421 | 974453 | 616 | 29.44974 | 1.63677 | 27.54704 | IP_DMC_vs_In_DMC_peak_106 | EDL933_RS04670:Promoter;EDL933_RS33100:CDS;EDL933_RS04660:Promoter;EDL933_RS04665:Promoter | EDL933_RS04670:NZ_CP008957.1:974526-975042:+:-57 | EDL933_RS04670(ompX) |
| NZ_CP008957.1 | 5395734 | 5396151 | 418 | 5395952 | 711 | 32.67683 | 1.62219 | 30.74185 | IP_DMC_vs_In_DMC_peak_514 | EDL933_RS27810:exon;EDL933_RS27805:Promoter;EDL933_RS27800:Promoter;EDL933_RS27815:Promoter | EDL933_RS27810:NZ_CP008957.1:5395882-5395967:+:60 | EDL933_RS27810(EDL933_RS27810) |
| NZ_CP008957.1 | 4021920 | 4022547 | 628 | 4022022 | 615 | 28.42519 | 1.62154 | 26.53391 | IP_DMC_vs_In_DMC_peak_339 | EDL933_RS31285:Promoter | EDL933_RS31285:NZ_CP008957.1:4022295-4022355:+:-62 | EDL933_RS31285(ibsE) |
| NZ_CP008957.1 | 3978531 | 3980137 | 1607 | 3979918 | 660 | 30.10363 | 1.61728 | 28.19407 | IP_DMC_vs_In_DMC_peak_335 | EDL933_RS20760:Promoter;EDL933_RS20745:CDS;EDL933_RS20740:Promoter;EDL933_RS20755:Promoter | EDL933_RS20740:NZ_CP008957.1:3978800-3979226:-:-107 | EDL933_RS20740(exbD) |
| NZ_CP008957.1 | 4403754 | 4404022 | 269 | 4403905 | 665 | 29.2383 | 1.60158 | 27.33784 | IP_DMC_vs_In_DMC_peak_384 | EDL933_RS22960:Promoter;EDL933_RS22955:Promoter;EDL933_RS22965:Promoter | EDL933_RS22965:NZ_CP008957.1:4404095-4404479:+:-207 | EDL933_RS22965(panM) |
| NZ_CP008957.1 | 3164786 | 3165118 | 333 | 3164940 | 583 | 25.81053 | 1.60152 | 23.94845 | IP_DMC_vs_In_DMC_peak_239 | EDL933_RS16675:Promoter;EDL933_RS16665:Promoter;EDL933_RS16680:Promoter;EDL933_RS16670:Promoter | EDL933_RS16675:NZ_CP008957.1:3164989-3165868:+:-37 | EDL933_RS16675(EDL933_RS16675) |
| NZ_CP008957.1 | 1354265 | 1355131 | 867 | 1354380 | 612 | 27.01961 | 1.60149 | 25.14366 | IP_DMC_vs_In_DMC_peak_137 | EDL933_RS06830:Promoter;EDL933_RS06815:Promoter;EDL933_RS06835:Promoter;EDL933_RS06820:Promoter;EDL933_RS06825:Promoter | EDL933_RS06820:NZ_CP008957.1:1354687-1354764:+:10 | EDL933_RS06820(EDL933_RS06820) |
| NZ_CP008957.1 | 4158743 | 4159157 | 415 | 4158943 | 637 | 27.85197 | 1.59828 | 25.96717 | IP_DMC_vs_In_DMC_peak_354 | EDL933_RS21650:Promoter;EDL933_RS21640:Promoter;EDL933_RS21655:Promoter;EDL933_RS21635:Promoter | EDL933_RS21650:NZ_CP008957.1:4158363-4158840:-:-109 | EDL933_RS21650(greA) |
| NZ_CP008957.1 | 4183890 | 4185199 | 1310 | 4184608 | 668 | 29.08256 | 1.59751 | 27.18403 | IP_DMC_vs_In_DMC_peak_356 | EDL933_RS21795:Promoter;EDL933_RS21805:Promoter;EDL933_RS21800:Promoter | EDL933_RS21805:NZ_CP008957.1:4184703-4189164:+:-159 | EDL933_RS21805(gltB) |
| NZ_CP008957.1 | 2917098 | 2917672 | 575 | 2917517 | 623 | 26.9432 | 1.59316 | 25.06808 | IP_DMC_vs_In_DMC_peak_215 | EDL933_RS15405:Promoter;EDL933_RS30905:Promoter;EDL933_RS15420:Promoter;EDL933_RS33635:Promoter;EDL933_RS15410:Promoter;EDL933_RS15415:Promoter;EDL933_RS33630:Promoter | EDL933_RS33635:NZ_CP008957.1:2917420-2917477:-:92 | EDL933_RS33635(EDL933_RS33635) |
| NZ_CP008957.1 | 1298054 | 1298423 | 370 | 1298248 | 609 | 26.19935 | 1.59043 | 24.33286 | IP_DMC_vs_In_DMC_peak_134 | EDL933_RS06450:Promoter;EDL933_RS06445:Promoter;EDL933_RS06425:Promoter;EDL933_RS06440:exon | EDL933_RS06440:NZ_CP008957.1:1298217-1298305:-:67 | EDL933_RS06440(EDL933_RS06440) |
| NZ_CP008957.1 | 4695484 | 4699202 | 3719 | 4697094 | 664 | 28.1245 | 1.58591 | 26.23664 | IP_DMC_vs_In_DMC_peak_426 | EDL933_RS24385:Promoter;EDL933_RS24375:Promoter;EDL933_RS24370:Promoter;EDL933_RS24365:Promoter;EDL933_RS24390:Promoter;EDL933_RS24395:Promoter;EDL933_RS24380:Promoter | EDL933_RS24375:NZ_CP008957.1:4697203-4697503:+:139 | EDL933_RS24375(espZ) |
| NZ_CP008957.1 | 542382 | 542733 | 352 | 542574 | 619 | 26.0775 | 1.58213 | 24.21221 | IP_DMC_vs_In_DMC_peak_65 | EDL933_RS02620:Promoter;EDL933_RS29160:exon;EDL933_RS02605:Promoter;EDL933_RS02615:Promoter | EDL933_RS29160:NZ_CP008957.1:542520-542617:+:37 | EDL933_RS29160(ffs) |
| NZ_CP008957.1 | 3344623 | 3345874 | 1252 | 3344861 | 595 | 24.95819 | 1.57944 | 23.10732 | IP_DMC_vs_In_DMC_peak_259 | EDL933_RS17545:Promoter;EDL933_RS17550:Promoter | EDL933_RS17550:NZ_CP008957.1:3345347-3347075:+:-99 | EDL933_RS17550(ptsI) |
| NZ_CP008957.1 | 376141 | 377282 | 1142 | 376879 | 602 | 25.04628 | 1.57638 | 23.19407 | IP_DMC_vs_In_DMC_peak_53 | EDL933_RS01850:Promoter;EDL933_RS01840:Promoter;EDL933_RS01835:Promoter;EDL933_RS01845:CDS | EDL933_RS01845:NZ_CP008957.1:376410-376998:-:287 | EDL933_RS01845(betI) |
| NZ_CP008957.1 | 171579 | 172254 | 676 | 171748 | 609 | 25.17746 | 1.57408 | 23.32372 | IP_DMC_vs_In_DMC_peak_22 | EDL933_RS00790:Promoter | EDL933_RS00790:NZ_CP008957.1:171806-174050:+:110 | EDL933_RS00790(fhuA) |
| NZ_CP008957.1 | 4200247 | 4200832 | 586 | 4200439 | 648 | 26.62678 | 1.57302 | 24.75538 | IP_DMC_vs_In_DMC_peak_357 | EDL933_RS21860:Promoter;EDL933_RS21855:Promoter | EDL933_RS21860:NZ_CP008957.1:4199644-4200283:-:-256 | EDL933_RS21860(sspA) |
| NZ_CP008957.1 | 3069943 | 3070287 | 345 | 3070150 | 572 | 23.57505 | 1.57136 | 21.74286 | IP_DMC_vs_In_DMC_peak_226 | EDL933_RS16265:Promoter | EDL933_RS16265:NZ_CP008957.1:3068089-3070069:-:-45 | EDL933_RS16265(cirA) |
| NZ_CP008957.1 | 3226258 | 3226751 | 494 | 3226515 | 591 | 24.25627 | 1.57045 | 22.41497 | IP_DMC_vs_In_DMC_peak_244 | EDL933_RS16935:Promoter;EDL933_RS16960:Promoter;EDL933_RS16950:Promoter;EDL933_RS16965:Promoter;EDL933_RS16940:Promoter;EDL933_RS16945:Promoter | EDL933_RS16960:NZ_CP008957.1:3226645-3227848:+:-141 | EDL933_RS16960(ackA) |
| NZ_CP008957.1 | 4249067 | 4249404 | 338 | 4249272 | 1068 | 42.58161 | 1.56994 | 40.56308 | IP_DMC_vs_In_DMC_peak_364 | EDL933_RS22105:exon | EDL933_RS22105:NZ_CP008957.1:4246511-4249414:-:179 | EDL933_RS22105(EDL933_RS22105) |
| NZ_CP008957.1 | 3125539 | 3125802 | 264 | 3125682 | 570 | 23.17829 | 1.56587 | 21.35168 | IP_DMC_vs_In_DMC_peak_234 | EDL933_RS16535:Promoter;EDL933_RS16545:Promoter;EDL933_RS16525:Promoter;EDL933_RS16530:Promoter | EDL933_RS16545:NZ_CP008957.1:3125775-3126264:+:-105 | EDL933_RS16545(eco) |
| NZ_CP008957.1 | 4155128 | 4158437 | 3310 | 4158220 | 637 | 25.46319 | 1.56168 | 23.60576 | IP_DMC_vs_In_DMC_peak_353 | EDL933_RS21640:Promoter;EDL933_RS21655:Promoter;EDL933_RS21635:Promoter | EDL933_RS21635:NZ_CP008957.1:4155125-4157060:-:278 | EDL933_RS21635(ftsH) |
| NZ_CP008957.1 | 3313216 | 3313869 | 654 | 3313418 | 585 | 23.45768 | 1.56095 | 21.62738 | IP_DMC_vs_In_DMC_peak_254 | EDL933_RS17375:Promoter;EDL933_RS17370:CDS | EDL933_RS17370:NZ_CP008957.1:3312194-3313433:-:-109 | EDL933_RS17370(alaC) |
| NZ_CP008957.1 | 3552783 | 3553509 | 727 | 3553243 | 566 | 21.89859 | 1.54626 | 20.0914 | IP_DMC_vs_In_DMC_peak_282 | EDL933_RS18520:Promoter;EDL933_RS18530:Promoter;EDL933_RS18515:Promoter;EDL933_RS18505:Promoter;EDL933_RS18510:Promoter | EDL933_RS18520:NZ_CP008957.1:3552945-3553194:-:48 | EDL933_RS18520(rpsP) |
| NZ_CP008957.1 | 703276 | 703783 | 508 | 703568 | 603 | 23.07799 | 1.54362 | 21.25315 | IP_DMC_vs_In_DMC_peak_80 | EDL933_RS03220:Promoter;EDL933_RS03215:Promoter;EDL933_RS03230:Promoter;EDL933_RS03225:CDS | EDL933_RS03225:NZ_CP008957.1:702618-703623:-:94 | EDL933_RS03225(fepD) |
| NZ_CP008957.1 | 1598097 | 1598990 | 894 | 1598421 | 561 | 21.43022 | 1.54119 | 19.62999 | IP_DMC_vs_In_DMC_peak_157 | EDL933_RS08115:Promoter;EDL933_RS08110:Promoter;EDL933_RS08105:Promoter | EDL933_RS08105:NZ_CP008957.1:1598444-1598681:+:99 | EDL933_RS08105(acpP) |
| NZ_CP008957.1 | 3575282 | 3575533 | 252 | 3575350 | 559 | 21.05012 | 1.53571 | 19.25568 | IP_DMC_vs_In_DMC_peak_291 | EDL933_RS18655:Promoter;EDL933_RS33725:Promoter;EDL933_RS18665:Promoter | EDL933_RS18665:NZ_CP008957.1:3574428-3575172:-:-235 | EDL933_RS18665(EDL933_RS18665) |
| NZ_CP008957.1 | 5308408 | 5308894 | 487 | 5308625 | 683 | 25.29506 | 1.53404 | 23.43965 | IP_DMC_vs_In_DMC_peak_508 | EDL933_RS27380:Promoter;EDL933_RS27375:Promoter | EDL933_RS27375:NZ_CP008957.1:5308781-5309207:+:-130 | EDL933_RS27375(nsrR) |
| NZ_CP008957.1 | 5279844 | 5280202 | 359 | 5280028 | 657 | 23.86381 | 1.52609 | 22.02807 | IP_DMC_vs_In_DMC_peak_502 | EDL933_RS27235:CDS;EDL933_RS27215:Promoter | EDL933_RS27235:NZ_CP008957.1:5280021-5280339:+:1 | EDL933_RS27235(sugE) |
| NZ_CP008957.1 | 3089245 | 3089540 | 296 | 3089394 | 554 | 20.11329 | 1.52199 | 18.33305 | IP_DMC_vs_In_DMC_peak_228 | EDL933_RS16350:Promoter;EDL933_RS16365:Promoter;EDL933_RS16360:Promoter | EDL933_RS16360:NZ_CP008957.1:3089492-3090959:+:-100 | EDL933_RS16360(EDL933_RS16360) |
| NZ_CP008957.1 | 1593389 | 1594724 | 1336 | 1593637 | 554 | 20.11329 | 1.52199 | 18.33305 | IP_DMC_vs_In_DMC_peak_156 | EDL933_RS08085:Promoter;EDL933_RS08090:Promoter;EDL933_RS08070:Promoter;EDL933_RS08080:Promoter;EDL933_RS08075:CDS | EDL933_RS08080:NZ_CP008957.1:1594196-1594370:+:-140 | EDL933_RS08080(rpmF) |
| NZ_CP008957.1 | 2435305 | 2435623 | 319 | 2435466 | 553 | 19.92824 | 1.51925 | 18.15082 | IP_DMC_vs_In_DMC_peak_184 | EDL933_RS12785:Promoter;EDL933_RS12775:Promoter | EDL933_RS12785:NZ_CP008957.1:2435584-2436733:+:-120 | EDL933_RS12785(cfa) |
| NZ_CP008957.1 | 2823631 | 2823989 | 359 | 2823817 | 560 | 19.99592 | 1.51625 | 18.21756 | IP_DMC_vs_In_DMC_peak_205 | EDL933_RS29235:Promoter;EDL933_RS14960:exon;EDL933_RS14945:Promoter | EDL933_RS14960:NZ_CP008957.1:2823783-2823859:+:26 | EDL933_RS14960(EDL933_RS14960) |
| NZ_CP008957.1 | 1141396 | 1141872 | 477 | 1141554 | 664 | 23.37872 | 1.51511 | 21.54935 | IP_DMC_vs_In_DMC_peak_114 | EDL933_RS05635:Promoter;EDL933_RS05625:Promoter;EDL933_RS05620:Promoter;EDL933_RS05630:Promoter;EDL933_RS05610:CDS | EDL933_RS05610:NZ_CP008957.1:1141087-1142301:-:667 | EDL933_RS05610(EDL933_RS05610) |
| NZ_CP008957.1 | 517626 | 518029 | 404 | 517860 | 566 | 19.61249 | 1.50586 | 17.83996 | IP_DMC_vs_In_DMC_peak_60 | EDL933_RS02500:Promoter;EDL933_RS02495:Promoter | EDL933_RS02500:NZ_CP008957.1:516715-517663:-:-164 | EDL933_RS02500(cyoA) |
| NZ_CP008957.1 | 3882158 | 3883784 | 1627 | 3883595 | 616 | 21.02166 | 1.50251 | 19.22802 | IP_DMC_vs_In_DMC_peak_327 | EDL933_RS20200:Promoter;EDL933_RS20210:Promoter;EDL933_RS33760:Promoter;EDL933_RS31185:Promoter | EDL933_RS20200:NZ_CP008957.1:3882325-3883558:-:587 | EDL933_RS20200(serA) |
| NZ_CP008957.1 | 1236396 | 1237110 | 715 | 1236874 | 548 | 18.77418 | 1.5011 | 17.01477 | IP_DMC_vs_In_DMC_peak_126 | EDL933_RS06020:Promoter;EDL933_RS06005:Promoter;EDL933_RS05995:Promoter | EDL933_RS06005:NZ_CP008957.1:1235815-1236856:-:103 | EDL933_RS06005(ompA) |
| NZ_CP008957.1 | 4201124 | 4201826 | 703 | 4201601 | 650 | 21.97298 | 1.50042 | 20.16481 | IP_DMC_vs_In_DMC_peak_358 | EDL933_RS21860:Promoter;EDL933_RS21865:Promoter;EDL933_RS21880:Promoter;EDL933_RS21870:Promoter;EDL933_RS21855:Promoter | EDL933_RS21870:NZ_CP008957.1:4201085-4201514:-:39 | EDL933_RS21870(rplM) |
| NZ_CP008957.1 | 3786177 | 3786560 | 384 | 3786362 | 607 | 20.26799 | 1.49468 | 18.48571 | IP_DMC_vs_In_DMC_peak_310 | EDL933_RS19715:Promoter;EDL933_RS19710:Promoter | EDL933_RS19715:NZ_CP008957.1:3786625-3787657:+:-257 | EDL933_RS19715(galR) |
| NZ_CP008957.1 | 4689170 | 4690679 | 1510 | 4690486 | 640 | 21.21339 | 1.49337 | 19.41671 | IP_DMC_vs_In_DMC_peak_423 | EDL933_RS24330:Promoter;EDL933_RS24325:Promoter;EDL933_RS24335:Promoter | EDL933_RS24330:NZ_CP008957.1:4689653-4690265:-:341 | EDL933_RS24330(map) |
| NZ_CP008957.1 | 3719509 | 3719901 | 393 | 3719718 | 584 | 19.3199 | 1.49064 | 17.55195 | IP_DMC_vs_In_DMC_peak_308 | EDL933_RS19410:Promoter;EDL933_RS19415:Promoter | EDL933_RS19415:NZ_CP008957.1:3718053-3719691:-:-13 | EDL933_RS19415(pyrG) |
| NZ_CP008957.1 | 180592 | 180949 | 358 | 180763 | 573 | 18.55806 | 1.48307 | 16.80207 | IP_DMC_vs_In_DMC_peak_24 | EDL933_RS00810:Promoter;EDL933_RS32990:CDS;EDL933_RS00820:Promoter | EDL933_RS32990:NZ_CP008957.1:180751-180817:+:19 | EDL933_RS32990(EDL933_RS32990) |
| NZ_CP008957.1 | 1902416 | 1902671 | 256 | 1902521 | 539 | 17.41926 | 1.48086 | 15.68137 | IP_DMC_vs_In_DMC_peak_174 | EDL933_RS09990:Promoter;EDL933_RS10000:Promoter;EDL933_RS09995:Promoter;EDL933_RS09985:CDS | EDL933_RS09985:NZ_CP008957.1:1902457-1902730:+:86 | EDL933_RS09985(EDL933_RS09985) |
| NZ_CP008957.1 | 4824730 | 4828262 | 3533 | 4827757 | 1812 | 53.87972 | 1.47313 | 51.78562 | IP_DMC_vs_In_DMC_peak_455 | EDL933_RS25070:Promoter;EDL933_RS25060:Promoter;EDL933_RS25065:Promoter;EDL933_RS25080:Promoter | EDL933_RS25060:NZ_CP008957.1:4827770-4827886:+:-1274 | EDL933_RS25060(rrf) |
| NZ_CP008957.1 | 5455059 | 5455334 | 276 | 5455205 | 611 | 18.90065 | 1.46955 | 17.13921 | IP_DMC_vs_In_DMC_peak_523 | EDL933_RS28030:Promoter;EDL933_RS28045:Promoter;EDL933_RS28040:Promoter | EDL933_RS28040:NZ_CP008957.1:5455341-5456526:+:-145 | EDL933_RS28040(uxuA) |
| NZ_CP008957.1 | 621033 | 621660 | 628 | 621304 | 534 | 16.56063 | 1.46715 | 14.83727 | IP_DMC_vs_In_DMC_peak_72 | intergenic | EDL933_RS32000:NZ_CP008957.1:620924-621185:+:422 | EDL933_RS32000(EDL933_RS32000) |
| NZ_CP008957.1 | 5015875 | 5016269 | 395 | 5016020 | 616 | 18.39201 | 1.4585 | 16.63888 | IP_DMC_vs_In_DMC_peak_476 | EDL933_RS26030:Promoter;EDL933_RS26025:Promoter;EDL933_RS26020:Promoter | EDL933_RS26025:NZ_CP008957.1:5016130-5016343:+:-58 | EDL933_RS26025(rpmE) |
| NZ_CP008957.1 | 4681455 | 4684512 | 3058 | 4684013 | 646 | 19.11296 | 1.45671 | 17.34806 | IP_DMC_vs_In_DMC_peak_422 | EDL933_RS24305:Promoter | EDL933_RS24310:NZ_CP008957.1:4682597-4683818:+:386 | EDL933_RS24310(escD) |
| NZ_CP008957.1 | 3875302 | 3875711 | 410 | 3875481 | 558 | 16.64943 | 1.45599 | 14.92481 | IP_DMC_vs_In_DMC_peak_325 | EDL933_RS20155:Promoter;EDL933_RS20150:Promoter;EDL933_RS33755:Promoter;EDL933_RS20160:Promoter | EDL933_RS20160:NZ_CP008957.1:3874326-3875421:-:-85 | EDL933_RS20160(gcvT) |
| NZ_CP008957.1 | 299831 | 300149 | 319 | 300006 | 576 | 17.09209 | 1.45504 | 15.35955 | IP_DMC_vs_In_DMC_peak_38 | EDL933_RS01415:exon | EDL933_RS01415:NZ_CP008957.1:299982-300058:+:7 | EDL933_RS01415(EDL933_RS01415) |
| NZ_CP008957.1 | 1157652 | 1158211 | 560 | 1157838 | 529 | 15.72196 | 1.45344 | 14.01428 | IP_DMC_vs_In_DMC_peak_118 | EDL933_RS05690:Promoter;EDL933_RS05695:Promoter | EDL933_RS05690:NZ_CP008957.1:1157871-1159164:+:60 | EDL933_RS05690(serS) |
| NZ_CP008957.1 | 1118532 | 1119287 | 756 | 1118670 | 625 | 18.24684 | 1.45187 | 16.49608 | IP_DMC_vs_In_DMC_peak_113 | EDL933_RS05465:Promoter;EDL933_RS05470:CDS;EDL933_RS05480:Promoter | EDL933_RS05470:NZ_CP008957.1:1118219-1119433:-:524 | EDL933_RS05470(EDL933_RS05470) |
| NZ_CP008957.1 | 4254217 | 4255239 | 1023 | 4254408 | 604 | 17.26302 | 1.44463 | 15.52768 | IP_DMC_vs_In_DMC_peak_367 | EDL933_RS22160:Promoter;EDL933_RS22130:Promoter;EDL933_RS22135:Promoter;EDL933_RS22145:CDS;EDL933_RS22140:Promoter | EDL933_RS22145:NZ_CP008957.1:4254058-4254601:-:-126 | EDL933_RS22145(EDL933_RS22145) |
| NZ_CP008957.1 | 3626488 | 3626789 | 302 | 3626648 | 534 | 15.36179 | 1.44365 | 13.66159 | IP_DMC_vs_In_DMC_peak_303 | EDL933_RS18920:Promoter | EDL933_RS18920:NZ_CP008957.1:3625996-3626512:-:-126 | EDL933_RS18920(luxS) |
| NZ_CP008957.1 | 1541552 | 1542317 | 766 | 1541700 | 602 | 16.86681 | 1.43857 | 15.1385 | IP_DMC_vs_In_DMC_peak_154 | EDL933_RS07775:Promoter;EDL933_RS07770:Promoter;EDL933_RS07780:Promoter;EDL933_RS07765:Promoter;EDL933_RS07755:CDS | EDL933_RS07755:NZ_CP008957.1:1541234-1542448:-:514 | EDL933_RS07755(EDL933_RS07755) |
| NZ_CP008957.1 | 229028 | 232176 | 3149 | 232061 | 1733 | 44.97107 | 1.43237 | 42.93396 | IP_DMC_vs_In_DMC_peak_30 | EDL933_RS01065:Promoter;EDL933_RS01070:Promoter;EDL933_RS01060:Promoter | EDL933_RS01060:NZ_CP008957.1:232087-232203:+:-1485 | EDL933_RS01060(rrf) |
| NZ_CP008957.1 | 1822770 | 1823262 | 493 | 1823089 | 520 | 14.26322 | 1.42876 | 12.58725 | IP_DMC_vs_In_DMC_peak_166 | EDL933_RS09460:CDS;EDL933_RS09465:Promoter | EDL933_RS09460:NZ_CP008957.1:1822739-1823153:-:137 | EDL933_RS09460(hns) |
| NZ_CP008957.1 | 4280513 | 4281318 | 806 | 4281035 | 558 | 15.20667 | 1.42862 | 13.5098 | IP_DMC_vs_In_DMC_peak_373 | EDL933_RS22365:CDS | EDL933_RS22365:NZ_CP008957.1:4278932-4281047:-:132 | EDL933_RS22365(fusA) |
| NZ_CP008957.1 | 2682286 | 2682634 | 349 | 2682443 | 519 | 14.10522 | 1.42601 | 12.43292 | IP_DMC_vs_In_DMC_peak_196 | EDL933_RS14075:Promoter;EDL933_RS14055:Promoter;EDL933_RS14050:Promoter;EDL933_RS14060:Promoter;EDL933_RS14080:Promoter | EDL933_RS14060:NZ_CP008957.1:2682538-2683042:+:-78 | EDL933_RS14060(EDL933_RS14060) |
| NZ_CP008957.1 | 1518671 | 1519433 | 763 | 1518824 | 630 | 16.79694 | 1.42508 | 15.06953 | IP_DMC_vs_In_DMC_peak_153 | EDL933_RS07625:CDS;EDL933_RS07635:Promoter;EDL933_RS07620:Promoter | EDL933_RS07625:NZ_CP008957.1:1518363-1519577:-:525 | EDL933_RS07625(EDL933_RS07625) |
| NZ_CP008957.1 | 236922 | 237479 | 558 | 237324 | 565 | 14.99132 | 1.42119 | 13.29915 | IP_DMC_vs_In_DMC_peak_31 | EDL933_RS01100:Promoter;EDL933_RS01090:CDS | EDL933_RS01090:NZ_CP008957.1:236147-237506:-:306 | EDL933_RS01090(mltD) |
| NZ_CP008957.1 | 2558693 | 2559040 | 348 | 2558902 | 517 | 13.79166 | 1.42053 | 12.12675 | IP_DMC_vs_In_DMC_peak_189 | EDL933_RS13420:CDS;EDL933_RS13425:Promoter;EDL933_RS13410:Promoter;EDL933_RS13405:Promoter;EDL933_RS13415:Promoter | EDL933_RS13420:NZ_CP008957.1:2558834-2559830:+:32 | EDL933_RS13420(gapA) |
| NZ_CP008957.1 | 5327486 | 5327858 | 373 | 5327605 | 617 | 16.15687 | 1.41948 | 14.44106 | IP_DMC_vs_In_DMC_peak_509 | EDL933_RS27480:Promoter;EDL933_RS27495:Promoter;EDL933_RS27485:Promoter;EDL933_RS27500:Promoter;EDL933_RS27490:Promoter | EDL933_RS27485:NZ_CP008957.1:5327709-5328105:+:-37 | EDL933_RS27485(rpsF) |
| NZ_CP008957.1 | 4299559 | 4300100 | 542 | 4299999 | 623 | 16.28522 | 1.41919 | 14.56698 | IP_DMC_vs_In_DMC_peak_377 | EDL933_RS22495:Promoter;EDL933_RS22500:Promoter;EDL933_RS22480:Promoter;EDL933_RS22475:Promoter;EDL933_RS22485:Promoter;EDL933_RS22490:Promoter | EDL933_RS22490:NZ_CP008957.1:4299257-4299830:-:1 | EDL933_RS22490(ppiA) |
| NZ_CP008957.1 | 755768 | 756036 | 269 | 755965 | 516 | 13.63611 | 1.41779 | 11.97485 | IP_DMC_vs_In_DMC_peak_86 | EDL933_RS03505:Promoter;EDL933_RS03490:Promoter;EDL933_RS03495:Promoter | EDL933_RS03490:NZ_CP008957.1:753335-755918:-:16 | EDL933_RS03490(leuS) |
| NZ_CP008957.1 | 2692881 | 2693271 | 391 | 2692990 | 526 | 13.69224 | 1.414 | 12.0298 | IP_DMC_vs_In_DMC_peak_197 | EDL933_RS34005:CDS;EDL933_RS14180:Promoter;EDL933_RS14175:Promoter | EDL933_RS34005:NZ_CP008957.1:2692877-2693495:+:198 | EDL933_RS34005(EDL933_RS34005) |
| NZ_CP008957.1 | 302329 | 302734 | 406 | 302510 | 567 | 14.51301 | 1.41113 | 12.83152 | IP_DMC_vs_In_DMC_peak_40 | EDL933_RS01435:CDS;EDL933_RS01420:Promoter;EDL933_RS01430:Promoter;EDL933_RS01450:Promoter;EDL933_RS01425:Promoter;EDL933_RS01445:Promoter;EDL933_RS01455:Promoter;EDL933_RS01440:Promoter | EDL933_RS01435:NZ_CP008957.1:301939-302653:-:122 | EDL933_RS01435(EDL933_RS01435) |
| NZ_CP008957.1 | 4469944 | 4470402 | 459 | 4470248 | 584 | 14.76813 | 1.40846 | 13.08085 | IP_DMC_vs_In_DMC_peak_392 | EDL933_RS23335:Promoter;EDL933_RS23330:Promoter;EDL933_RS23315:Promoter;EDL933_RS23325:Promoter | EDL933_RS23325:NZ_CP008957.1:4470467-4470821:+:-294 | EDL933_RS23325(arsR) |
| NZ_CP008957.1 | 310467 | 310771 | 305 | 310629 | 534 | 13.5564 | 1.40737 | 11.89732 | IP_DMC_vs_In_DMC_peak_42 | EDL933_RS01515:Promoter;EDL933_RS01495:Promoter;EDL933_RS01510:Promoter;EDL933_RS01505:Promoter | EDL933_RS01505:NZ_CP008957.1:310806-311988:+:-187 | EDL933_RS01505(EDL933_RS01505) |
| NZ_CP008957.1 | 3298541 | 3299105 | 565 | 3298861 | 531 | 13.39659 | 1.40551 | 11.74128 | IP_DMC_vs_In_DMC_peak_250 | EDL933_RS17315:Promoter;EDL933_RS17305:Promoter;EDL933_RS17310:Promoter;EDL933_RS17300:Promoter | EDL933_RS17310:NZ_CP008957.1:3298896-3299511:+:-73 | EDL933_RS17310(evgA) |
| NZ_CP008957.1 | 4840806 | 4841128 | 323 | 4840956 | 609 | 15.13603 | 1.40467 | 13.44056 | IP_DMC_vs_In_DMC_peak_457 | EDL933_RS25140:Promoter;EDL933_RS25130:Promoter | EDL933_RS25130:NZ_CP008957.1:4840561-4840843:-:-123 | EDL933_RS25130(ppiC) |
| NZ_CP008957.1 | 4806739 | 4807622 | 884 | 4807514 | 647 | 15.85458 | 1.40216 | 14.14471 | IP_DMC_vs_In_DMC_peak_453 | EDL933_RS24975:CDS;EDL933_RS24985:Promoter;EDL933_RS24970:Promoter | EDL933_RS24975:NZ_CP008957.1:4807079-4807523:-:343 | EDL933_RS24975(mioC) |
| NZ_CP008957.1 | 2496574 | 2496974 | 401 | 2496802 | 510 | 12.72019 | 1.40133 | 11.08066 | IP_DMC_vs_In_DMC_peak_188 | EDL933_RS13090:Promoter;EDL933_RS13080:Promoter;EDL933_RS13085:Promoter | EDL933_RS13085:NZ_CP008957.1:2494815-2496744:-:-29 | EDL933_RS13085(thrS) |
| NZ_CP008957.1 | 1068296 | 1069239 | 944 | 1068826 | 631 | 15.39223 | 1.40039 | 13.69103 | IP_DMC_vs_In_DMC_peak_112 | EDL933_RS05120:CDS;EDL933_RS34090:Promoter;EDL933_RS33115:Promoter | EDL933_RS33115:NZ_CP008957.1:1067924-1068167:-:-600 | EDL933_RS33115(EDL933_RS33115) |
| NZ_CP008957.1 | 2867944 | 2871004 | 3061 | 2868506 | 520 | 12.71481 | 1.39645 | 11.07597 | IP_DMC_vs_In_DMC_peak_211 | EDL933_RS15205:CDS;EDL933_RS15200:Promoter;EDL933_RS15195:Promoter;EDL933_RS15190:Promoter | EDL933_RS15205:NZ_CP008957.1:2868051-2869170:-:-303 | EDL933_RS15205(gmd) |
| NZ_CP008957.1 | 423248 | 424284 | 1037 | 423542 | 38230 | 831.94226 | 1.39612 | 828.17468 | IP_DMC_vs_In_DMC_peak_57 | EDL933_RS02035:CDS;EDL933_RS02030:Promoter | EDL933_RS02035:NZ_CP008957.1:423203-424286:-:520 | EDL933_RS02035(lacI) |
| NZ_CP008957.1 | 2440575 | 2440874 | 300 | 2440751 | 508 | 12.42152 | 1.39585 | 10.7894 | IP_DMC_vs_In_DMC_peak_185 | EDL933_RS12800:Promoter;EDL933_RS12815:Promoter;EDL933_RS12820:Promoter;EDL933_RS12810:exon | EDL933_RS12810:NZ_CP008957.1:2440687-2440764:+:37 | EDL933_RS12810(EDL933_RS12810) |
| NZ_CP008957.1 | 4484112 | 4485043 | 932 | 4484740 | 575 | 13.77234 | 1.3934 | 12.10821 | IP_DMC_vs_In_DMC_peak_396 | EDL933_RS23395:CDS | EDL933_RS23395:NZ_CP008957.1:4484191-4484962:+:386 | EDL933_RS23395(EDL933_RS23395) |
| NZ_CP008957.1 | 2654915 | 2655241 | 327 | 2655150 | 507 | 12.27344 | 1.39311 | 10.645 | IP_DMC_vs_In_DMC_peak_195 | EDL933_RS13935:CDS;EDL933_RS13925:Promoter;EDL933_RS13930:Promoter;EDL933_RS13945:Promoter | EDL933_RS13930:NZ_CP008957.1:2653423-2654524:-:-553 | EDL933_RS13930(EDL933_RS13930) |
| NZ_CP008957.1 | 2830405 | 2832268 | 1864 | 2830689 | 642 | 15.11448 | 1.39134 | 13.41941 | IP_DMC_vs_In_DMC_peak_206 | EDL933_RS15005:CDS;EDL933_RS14995:Promoter;EDL933_RS14990:Promoter;EDL933_RS15010:Promoter | EDL933_RS15010:NZ_CP008957.1:2830831-2832370:+:505 | EDL933_RS15010(EDL933_RS15010) |
| NZ_CP008957.1 | 93995 | 94448 | 454 | 94258 | 541 | 12.69605 | 1.38654 | 11.0576 | IP_DMC_vs_In_DMC_peak_12 | EDL933_RS00445:Promoter;EDL933_RS00440:CDS;EDL933_RS00450:Promoter;EDL933_RS00455:Promoter | EDL933_RS00440:NZ_CP008957.1:94239-94698:+:-18 | EDL933_RS00440(mraZ) |
| NZ_CP008957.1 | 2284326 | 2285267 | 942 | 2285088 | 503 | 11.68951 | 1.38214 | 10.07654 | IP_DMC_vs_In_DMC_peak_178 | EDL933_RS11965:Promoter;EDL933_RS11960:Promoter;EDL933_RS11950:Promoter;EDL933_RS11955:CDS | EDL933_RS11950:NZ_CP008957.1:2284127-2284718:-:-78 | EDL933_RS11950(espM1) |
| NZ_CP008957.1 | 4278878 | 4279462 | 585 | 4279218 | 529 | 12.21883 | 1.38185 | 10.59219 | IP_DMC_vs_In_DMC_peak_371 | EDL933_RS22365:CDS;EDL933_RS22350:Promoter;EDL933_RS22360:Promoter;EDL933_RS22355:Promoter | EDL933_RS22360:NZ_CP008957.1:4277677-4278862:-:-307 | EDL933_RS22360(tuf) |
| NZ_CP008957.1 | 5175918 | 5176181 | 264 | 5176074 | 568 | 12.96248 | 1.3805 | 11.31753 | IP_DMC_vs_In_DMC_peak_494 | EDL933_RS26745:Promoter;EDL933_RS26755:Promoter;EDL933_RS26740:Promoter | EDL933_RS26745:NZ_CP008957.1:5176137-5176674:+:-88 | EDL933_RS26745(ssb1) |
| NZ_CP008957.1 | 289792 | 290301 | 510 | 290180 | 510 | 11.72113 | 1.37962 | 10.10741 | IP_DMC_vs_In_DMC_peak_36 | EDL933_RS01375:Promoter;EDL933_RS01370:Promoter;EDL933_RS01365:Promoter;EDL933_RS01360:Promoter;EDL933_RS01355:CDS | EDL933_RS01360:NZ_CP008957.1:290187-290586:+:-141 | EDL933_RS01360(yafO) |
| NZ_CP008957.1 | 4775851 | 4778337 | 2487 | 4776473 | 578 | 13.06645 | 1.37849 | 11.41884 | IP_DMC_vs_In_DMC_peak_443 | EDL933_RS24825:Promoter;EDL933_RS24820:Promoter | EDL933_RS24825:NZ_CP008957.1:4776618-4777290:+:475 | EDL933_RS24825(EDL933_RS24825) |
| NZ_CP008957.1 | 931220 | 934635 | 3416 | 932679 | 510 | 11.65778 | 1.37823 | 10.04632 | IP_DMC_vs_In_DMC_peak_100 | EDL933_RS04470:Promoter;EDL933_RS04460:CDS;EDL933_RS04450:Promoter;EDL933_RS04465:Promoter | EDL933_RS04460:NZ_CP008957.1:932517-933672:+:410 | EDL933_RS04460(bioF) |
| NZ_CP008957.1 | 4074784 | 4075208 | 425 | 4075014 | 543 | 12.28728 | 1.37723 | 10.65863 | IP_DMC_vs_In_DMC_peak_345 | EDL933_RS21210:Promoter;EDL933_RS21215:Promoter;EDL933_RS21220:Promoter;EDL933_RS21205:Promoter;EDL933_RS21225:Promoter | EDL933_RS21205:NZ_CP008957.1:4075031-4075694:+:-35 | EDL933_RS21205(EDL933_RS21205) |
| NZ_CP008957.1 | 1888280 | 1889471 | 1192 | 1889205 | 656 | 14.55878 | 1.37666 | 12.87639 | IP_DMC_vs_In_DMC_peak_172 | EDL933_RS09870:Promoter;EDL933_RS09855:CDS | EDL933_RS09855:NZ_CP008957.1:1888173-1889712:-:837 | EDL933_RS09855(EDL933_RS09855) |
| NZ_CP008957.1 | 1265395 | 1265678 | 284 | 1265519 | 501 | 11.40259 | 1.37665 | 9.7978 | IP_DMC_vs_In_DMC_peak_131 | EDL933_RS06240:Promoter;EDL933_RS06225:Promoter;EDL933_RS06220:Promoter;EDL933_RS06215:Promoter;EDL933_RS06230:Promoter;EDL933_RS33975:Promoter | EDL933_RS06215:NZ_CP008957.1:1265673-1265870:+:-137 | EDL933_RS06215(EDL933_RS06215) |
| NZ_CP008957.1 | 3342098 | 3342694 | 597 | 3342547 | 540 | 12.15772 | 1.37581 | 10.53243 | IP_DMC_vs_In_DMC_peak_258 | EDL933_RS17530:Promoter;EDL933_RS17525:Promoter;EDL933_RS17540:Promoter;EDL933_RS17535:Promoter | EDL933_RS17530:NZ_CP008957.1:3341516-3342515:-:119 | EDL933_RS17530(zipA) |
| NZ_CP008957.1 | 346486 | 347634 | 1149 | 347089 | 640 | 14.14613 | 1.37511 | 12.4731 | IP_DMC_vs_In_DMC_peak_48 | EDL933_RS01690:Promoter;EDL933_RS01705:CDS | EDL933_RS01705:NZ_CP008957.1:346223-347762:+:836 | EDL933_RS01705(EDL933_RS01705) |
| NZ_CP008957.1 | 5054884 | 5055206 | 323 | 5055066 | 595 | 13.03243 | 1.37121 | 11.38546 | IP_DMC_vs_In_DMC_peak_479 | EDL933_RS26200:Promoter;EDL933_RS26190:Promoter;EDL933_RS26195:Promoter | EDL933_RS26190:NZ_CP008957.1:5053652-5055053:-:8 | EDL933_RS26190(sthA) |
| NZ_CP008957.1 | 3000663 | 3001723 | 1061 | 3001007 | 777 | 16.3145 | 1.36636 | 14.59582 | IP_DMC_vs_In_DMC_peak_220 | EDL933_RS15785:Promoter;EDL933_RS15800:CDS;EDL933_RS15790:Promoter | EDL933_RS15800:NZ_CP008957.1:3000574-3001788:+:618 | EDL933_RS15800(EDL933_RS15800) |
| NZ_CP008957.1 | 4495353 | 4495851 | 499 | 4495475 | 587 | 12.61482 | 1.36619 | 10.97806 | IP_DMC_vs_In_DMC_peak_399 | EDL933_RS23440:Promoter;EDL933_RS23445:CDS | EDL933_RS23445:NZ_CP008957.1:4494744-4495569:-:-32 | EDL933_RS23445(gadX) |
| NZ_CP008957.1 | 4597647 | 4598546 | 900 | 4598016 | 569 | 12.26275 | 1.36616 | 10.63521 | IP_DMC_vs_In_DMC_peak_408 | EDL933_RS23880:CDS | EDL933_RS23880:NZ_CP008957.1:4597916-4598252:+:180 | EDL933_RS23880(EDL933_RS23880) |
| NZ_CP008957.1 | 2877980 | 2878246 | 267 | 2878080 | 497 | 10.83891 | 1.36568 | 9.25058 | IP_DMC_vs_In_DMC_peak_213 | EDL933_RS15245:Promoter;EDL933_RS15240:Promoter | EDL933_RS15245:NZ_CP008957.1:2877036-2878032:-:-80 | EDL933_RS15245(EDL933_RS15245) |
| NZ_CP008957.1 | 2861682 | 2863600 | 1919 | 2861904 | 497 | 10.83891 | 1.36568 | 9.25058 | IP_DMC_vs_In_DMC_peak_208 | EDL933_RS15165:Promoter;EDL933_RS15170:Promoter;EDL933_RS15175:CDS | EDL933_RS15175:NZ_CP008957.1:2861839-2862505:-:-135 | EDL933_RS15175(perB) |
| NZ_CP008957.1 | 3020932 | 3021431 | 500 | 3021171 | 556 | 11.97326 | 1.36541 | 10.35322 | IP_DMC_vs_In_DMC_peak_222 | EDL933_RS15970:CDS;EDL933_RS15965:Promoter;EDL933_RS15960:Promoter;EDL933_RS15955:Promoter;EDL933_RS15980:Promoter;EDL933_RS15950:Promoter;EDL933_RS15975:Promoter | EDL933_RS15970:NZ_CP008957.1:3021068-3021716:+:113 | EDL933_RS15970(EDL933_RS15970) |
| NZ_CP008958.1 | 35643 | 36074 | 432 | 35885 | 1849 | 36.80141 | 1.36516 | 34.82874 | IP_DMC_vs_In_DMC_peak_546 | EDL933_RS28755:Promoter;EDL933_RS28750:CDS;EDL933_RS28770:Promoter;EDL933_RS28760:Promoter | EDL933_RS28755:NZ_CP008958.1:36086-36920:+:-228 | EDL933_RS28755(EDL933_RS28755) |
| NZ_CP008957.1 | 4921231 | 4924375 | 3145 | 4924268 | 2446 | 47.95794 | 1.36393 | 45.90023 | IP_DMC_vs_In_DMC_peak_467 | EDL933_RS25570:Promoter;EDL933_RS25565:Promoter;EDL933_RS25550:exon | EDL933_RS25550:NZ_CP008957.1:4924266-4924382:+:-1463 | EDL933_RS25550(rrf) |
| NZ_CP008957.1 | 3293630 | 3293985 | 356 | 3293765 | 536 | 11.46975 | 1.36295 | 9.86347 | IP_DMC_vs_In_DMC_peak_248 | EDL933_RS17290:Promoter;EDL933_RS17285:CDS;EDL933_RS17295:Promoter | EDL933_RS17285:NZ_CP008957.1:3292869-3293928:-:121 | EDL933_RS17285(EDL933_RS17285) |
| NZ_CP008957.1 | 2955651 | 2955972 | 322 | 2955837 | 496 | 10.70011 | 1.36294 | 9.11587 | IP_DMC_vs_In_DMC_peak_217 | EDL933_RS15590:CDS;EDL933_RS15580:Promoter;EDL933_RS15585:Promoter | EDL933_RS15585:NZ_CP008957.1:2954968-2955643:-:-168 | EDL933_RS15585(EDL933_RS15585) |
| NZ_CP008957.1 | 3564420 | 3565006 | 587 | 3564607 | 519 | 11.12962 | 1.36266 | 9.53284 | IP_DMC_vs_In_DMC_peak_287 | EDL933_RS18580:Promoter;EDL933_RS18585:Promoter;EDL933_RS29085:Promoter;EDL933_RS18590:Promoter | EDL933_RS18585:NZ_CP008957.1:3564644-3565295:+:68 | EDL933_RS18585(EDL933_RS18585) |
| NZ_CP008957.1 | 271030 | 271327 | 298 | 271181 | 523 | 11.18833 | 1.36226 | 9.59004 | IP_DMC_vs_In_DMC_peak_35 | EDL933_RS01265:Promoter;EDL933_RS01250:Promoter;EDL933_RS01245:CDS | EDL933_RS01250:NZ_CP008957.1:271361-271907:+:-183 | EDL933_RS01250(EDL933_RS01250) |
| NZ_CP008957.1 | 5020330 | 5021297 | 968 | 5020712 | 497 | 10.68228 | 1.36209 | 9.09913 | IP_DMC_vs_In_DMC_peak_477 | EDL933_RS26035:CDS | EDL933_RS26035:NZ_CP008957.1:5020688-5020925:+:125 | EDL933_RS26035(EDL933_RS26035) |
| NZ_CP008957.1 | 1390937 | 1391410 | 474 | 1391085 | 585 | 12.33866 | 1.36155 | 10.7089 | IP_DMC_vs_In_DMC_peak_143 | intergenic | EDL933_RS07020:NZ_CP008957.1:1391478-1391823:-:650 | EDL933_RS07020(EDL933_RS07020) |
| NZ_CP008957.1 | 3718289 | 3719004 | 716 | 3718674 | 542 | 11.48305 | 1.3608 | 9.87654 | IP_DMC_vs_In_DMC_peak_307 | EDL933_RS19410:Promoter;EDL933_RS19415:CDS | EDL933_RS19410:NZ_CP008957.1:3716667-3717966:-:-680 | EDL933_RS19410(eno) |
| NZ_CP008957.1 | 3811207 | 3812609 | 1403 | 3811719 | 535 | 11.33006 | 1.36037 | 9.72769 | IP_DMC_vs_In_DMC_peak_319 | EDL933_RS19880:CDS;EDL933_RS19870:Promoter | EDL933_RS19870:NZ_CP008957.1:3810460-3811582:-:-325 | EDL933_RS19870(EDL933_RS19870) |
| NZ_CP008957.1 | 1742592 | 1744694 | 2103 | 1743754 | 637 | 13.24461 | 1.36002 | 11.59302 | IP_DMC_vs_In_DMC_peak_164 | EDL933_RS09035:Promoter;EDL933_RS09055:CDS | EDL933_RS09055:NZ_CP008957.1:1743240-1744779:+:402 | EDL933_RS09055(EDL933_RS09055) |
| NZ_CP008957.1 | 3515756 | 3517480 | 1725 | 3517275 | 509 | 10.8154 | 1.35991 | 9.22832 | IP_DMC_vs_In_DMC_peak_275 | EDL933_RS18345:Promoter;EDL933_RS33700:CDS;EDL933_RS18325:Promoter;EDL933_RS18330:Promoter;EDL933_RS18335:CDS | EDL933_RS18330:NZ_CP008957.1:3516029-3516668:-:50 | EDL933_RS18330(rseA) |
| NZ_CP008957.1 | 345951 | 346203 | 253 | 346074 | 674 | 13.8366 | 1.35818 | 12.1708 | IP_DMC_vs_In_DMC_peak_47 | EDL933_RS01705:Promoter;EDL933_RS01700:CDS;EDL933_RS01685:Promoter;EDL933_RS01690:Promoter | EDL933_RS01705:NZ_CP008957.1:346223-347762:+:-146 | EDL933_RS01705(EDL933_RS01705) |
| NZ_CP008957.1 | 870987 | 871375 | 389 | 871212 | 524 | 11.00602 | 1.35785 | 9.41282 | IP_DMC_vs_In_DMC_peak_96 | EDL933_RS04085:CDS | EDL933_RS04085:NZ_CP008957.1:870485-871238:-:57 | EDL933_RS04085(gpmA) |
| NZ_CP008957.1 | 1232444 | 1232741 | 298 | 1232605 | 500 | 10.51467 | 1.35692 | 8.93645 | IP_DMC_vs_In_DMC_peak_125 | EDL933_RS05985:CDS | EDL933_RS05985:NZ_CP008957.1:1232517-1232685:+:75 | EDL933_RS05985(rmf) |
| NZ_CP008957.1 | 5106441 | 5109582 | 3142 | 5109474 | 2435 | 45.57421 | 1.35342 | 43.53255 | IP_DMC_vs_In_DMC_peak_489 | EDL933_RS26445:exon;EDL933_RS26455:Promoter | EDL933_RS26445:NZ_CP008957.1:5109473-5109589:+:-1462 | EDL933_RS26445(rrf) |
| NZ_CP008957.1 | 4073750 | 4073993 | 244 | 4073868 | 529 | 10.8664 | 1.35275 | 9.27739 | IP_DMC_vs_In_DMC_peak_344 | EDL933_RS21210:Promoter;EDL933_RS21200:Promoter;EDL933_RS21205:Promoter;EDL933_RS21190:Promoter | EDL933_RS21200:NZ_CP008957.1:4073910-4074687:+:-39 | EDL933_RS21200(exuR) |
| NZ_CP008957.1 | 4455106 | 4456309 | 1204 | 4455839 | 561 | 11.41721 | 1.352 | 9.81207 | IP_DMC_vs_In_DMC_peak_390 | EDL933_RS23260:Promoter;EDL933_RS23245:CDS;EDL933_RS23240:Promoter | EDL933_RS23245:NZ_CP008957.1:4454952-4456563:-:856 | EDL933_RS23245(EDL933_RS23245) |
| NZ_CP008957.1 | 5053385 | 5053686 | 302 | 5053489 | 559 | 11.36565 | 1.35169 | 9.76249 | IP_DMC_vs_In_DMC_peak_478 | EDL933_RS26185:CDS;EDL933_RS26195:Promoter | EDL933_RS26185:NZ_CP008957.1:5052752-5053670:+:783 | EDL933_RS26185(oxyR) |
| NZ_CP008957.1 | 5278648 | 5279026 | 379 | 5278814 | 544 | 11.09155 | 1.35168 | 9.49641 | IP_DMC_vs_In_DMC_peak_501 | EDL933_RS27225:Promoter;EDL933_RS27210:Promoter;EDL933_RS27230:Promoter;EDL933_RS27220:Promoter;EDL933_RS27235:Promoter;EDL933_RS27215:Promoter | EDL933_RS27220:NZ_CP008957.1:5278845-5279412:+:-8 | EDL933_RS27220(efp) |
| NZ_CP008957.1 | 4674733 | 4675961 | 1229 | 4675808 | 628 | 12.57897 | 1.35086 | 10.94304 | IP_DMC_vs_In_DMC_peak_418 | EDL933_RS33850:Promoter;EDL933_RS24260:CDS | EDL933_RS24260:NZ_CP008957.1:4674477-4676016:+:869 | EDL933_RS24260(EDL933_RS24260) |
| NZ_CP008957.1 | 5444326 | 5444844 | 519 | 5444617 | 550 | 11.13791 | 1.35035 | 9.54089 | IP_DMC_vs_In_DMC_peak_520 | EDL933_RS27985:Promoter;EDL933_RS27980:Promoter;EDL933_RS27965:Promoter | EDL933_RS27980:NZ_CP008957.1:5444748-5445351:+:-163 | EDL933_RS27980(fimB) |
| NZ_CP008957.1 | 3548807 | 3549102 | 296 | 3548956 | 498 | 10.15319 | 1.34941 | 8.58622 | IP_DMC_vs_In_DMC_peak_280 | EDL933_RS18475:Promoter;EDL933_RS18500:Promoter;EDL933_RS18490:CDS;EDL933_RS18480:Promoter;EDL933_RS18495:Promoter | EDL933_RS18490:NZ_CP008957.1:3548882-3549401:+:72 | EDL933_RS18490(EDL933_RS18490) |
| NZ_CP008957.1 | 2597919 | 2598181 | 263 | 2598034 | 491 | 10.01896 | 1.34923 | 8.45627 | IP_DMC_vs_In_DMC_peak_190 | EDL933_RS13625:Promoter;EDL933_RS13610:Promoter;EDL933_RS13620:Promoter | EDL933_RS13610:NZ_CP008957.1:2596319-2597876:-:-173 | EDL933_RS13610(yoaE) |
| NZ_CP008957.1 | 4456658 | 4457271 | 614 | 4456881 | 577 | 11.50186 | 1.34781 | 9.89491 | IP_DMC_vs_In_DMC_peak_391 | EDL933_RS23260:Promoter;EDL933_RS23245:Promoter | EDL933_RS23260:NZ_CP008957.1:4456946-4457999:+:18 | EDL933_RS23260(EDL933_RS23260) |
| NZ_CP008957.1 | 204827 | 205255 | 429 | 205103 | 508 | 10.2076 | 1.34651 | 8.63877 | IP_DMC_vs_In_DMC_peak_28 | EDL933_RS00930:Promoter;EDL933_RS00940:Promoter;EDL933_RS00935:Promoter;EDL933_RS00925:CDS | EDL933_RS00930:NZ_CP008957.1:205444-205900:+:-403 | EDL933_RS00930(fabZ) |
| NZ_CP008957.1 | 524623 | 525224 | 602 | 524885 | 490 | 9.88531 | 1.34649 | 8.32712 | IP_DMC_vs_In_DMC_peak_63 | EDL933_RS02540:Promoter | EDL933_RS02540:NZ_CP008957.1:524939-527294:+:-16 | EDL933_RS02540(lon) |
| NZ_CP008957.1 | 3138153 | 3138370 | 218 | 3138246 | 494 | 9.94978 | 1.34632 | 8.3898 | IP_DMC_vs_In_DMC_peak_237 | intergenic | EDL933_RS16595:NZ_CP008957.1:3137482-3138133:+:779 | EDL933_RS16595(rcsB) |
| NZ_CP008957.1 | 3542905 | 3543200 | 296 | 3543043 | 492 | 9.86388 | 1.34513 | 8.30706 | IP_DMC_vs_In_DMC_peak_278 | EDL933_RS18450:Promoter;EDL933_RS18470:Promoter;EDL933_RS33705:Promoter;EDL933_RS18465:Promoter;EDL933_RS18445:Promoter;EDL933_RS18440:Promoter;EDL933_RS18455:Promoter | EDL933_RS18450:NZ_CP008957.1:3542038-3543019:-:-33 | EDL933_RS18450(rluD) |
| NZ_CP008957.1 | 4537901 | 4538240 | 340 | 4538088 | 584 | 11.47254 | 1.3447 | 9.86622 | IP_DMC_vs_In_DMC_peak_404 | EDL933_RS23625:exon;EDL933_RS23615:Promoter | EDL933_RS23625:NZ_CP008957.1:4538062-4538139:-:69 | EDL933_RS23625(EDL933_RS23625) |
| NZ_CP008957.1 | 5304083 | 5306018 | 1936 | 5304815 | 545 | 10.77679 | 1.34456 | 9.19064 | IP_DMC_vs_In_DMC_peak_505 | EDL933_RS27360:Promoter;EDL933_RS27355:CDS | EDL933_RS27355:NZ_CP008957.1:5304629-5305889:+:421 | EDL933_RS27355(hflK) |
| NZ_CP008957.1 | 4677640 | 4678187 | 548 | 4677900 | 549 | 10.84109 | 1.34442 | 9.25268 | IP_DMC_vs_In_DMC_peak_420 | EDL933_RS24270:Promoter;EDL933_RS24275:CDS | EDL933_RS24275:NZ_CP008957.1:4677711-4677990:-:77 | EDL933_RS24275(escG) |
| NZ_CP008957.1 | 4141276 | 4142028 | 753 | 4141918 | 551 | 10.86698 | 1.34422 | 9.27796 | IP_DMC_vs_In_DMC_peak_350 | EDL933_RS21570:Promoter;EDL933_RS21565:Promoter | EDL933_RS21570:NZ_CP008957.1:4141538-4141808:-:156 | EDL933_RS21570(rpsO) |
| NZ_CP008957.1 | 3400618 | 3400851 | 234 | 3400758 | 509 | 9.96573 | 1.34053 | 8.40533 | IP_DMC_vs_In_DMC_peak_262 | EDL933_RS17810:Promoter;EDL933_RS17800:Promoter;EDL933_RS17805:Promoter | EDL933_RS17810:NZ_CP008957.1:3399890-3400604:-:-130 | EDL933_RS17810(purC) |
| NZ_CP008957.1 | 4745521 | 4746284 | 764 | 4746026 | 569 | 10.93874 | 1.33914 | 9.34789 | IP_DMC_vs_In_DMC_peak_435 | EDL933_RS24680:CDS;EDL933_RS24695:Promoter;EDL933_RS24685:Promoter;EDL933_RS24675:Promoter;EDL933_RS24665:Promoter | EDL933_RS24680:NZ_CP008957.1:4745616-4746030:-:128 | EDL933_RS24680(ibpA) |
| NZ_CP008957.1 | 1274814 | 1277079 | 2266 | 1275441 | 631 | 11.84764 | 1.3362 | 10.23035 | IP_DMC_vs_In_DMC_peak_133 | EDL933_RS06315:CDS;EDL933_RS06290:Promoter;EDL933_RS06325:Promoter;EDL933_RS29840:Promoter;EDL933_RS06320:Promoter | EDL933_RS06320:NZ_CP008957.1:1275581-1277120:+:365 | EDL933_RS06320(EDL933_RS06320) |
| NZ_CP008957.1 | 5400929 | 5401263 | 335 | 5401058 | 558 | 10.60218 | 1.33602 | 9.02099 | IP_DMC_vs_In_DMC_peak_516 | EDL933_RS27850:Promoter;EDL933_RS27830:Promoter;EDL933_RS27835:Promoter | EDL933_RS27835:NZ_CP008957.1:5400101-5400434:-:-661 | EDL933_RS27835(EDL933_RS27835) |
| NZ_CP008957.1 | 4778787 | 4780545 | 1759 | 4779213 | 544 | 10.29842 | 1.33457 | 8.72645 | IP_DMC_vs_In_DMC_peak_444 | EDL933_RS24840:CDS | EDL933_RS24840:NZ_CP008957.1:4778274-4780659:-:993 | EDL933_RS24840(EDL933_RS24840) |
| NZ_CP008957.1 | 791165 | 791469 | 305 | 791329 | 486 | 9.3134 | 1.3344 | 7.77483 | IP_DMC_vs_In_DMC_peak_89 | EDL933_RS03675:Promoter;EDL933_RS03690:Promoter;EDL933_RS03680:Promoter;EDL933_RS03705:Promoter | EDL933_RS03690:NZ_CP008957.1:790735-791266:-:-50 | EDL933_RS03690(fldA) |
| NZ_CP008957.1 | 3957073 | 3958109 | 1037 | 3957761 | 771 | 14.07502 | 1.33405 | 12.40394 | IP_DMC_vs_In_DMC_peak_333 | EDL933_RS20620:Promoter;EDL933_RS20600:CDS;EDL933_RS20615:Promoter | EDL933_RS20600:NZ_CP008957.1:3957000-3958214:-:623 | EDL933_RS20600(EDL933_RS20600) |
| NZ_CP008957.1 | 4404920 | 4405316 | 397 | 4405191 | 546 | 10.24952 | 1.33277 | 8.6796 | IP_DMC_vs_In_DMC_peak_385 | EDL933_RS22970:Promoter;EDL933_RS22960:Promoter;EDL933_RS22975:CDS | EDL933_RS22970:NZ_CP008957.1:4404475-4405003:-:-114 | EDL933_RS22970(EDL933_RS22970) |
| NZ_CP008957.1 | 1839008 | 1839438 | 431 | 1839199 | 485 | 9.22998 | 1.33277 | 7.69384 | IP_DMC_vs_In_DMC_peak_168 | EDL933_RS09530:CDS;EDL933_RS09525:Promoter;EDL933_RS09520:Promoter | EDL933_RS09530:NZ_CP008957.1:1839119-1839839:+:103 | EDL933_RS09530(tonB) |
| NZ_CP008957.1 | 522477 | 522957 | 481 | 522627 | 485 | 9.22998 | 1.33277 | 7.69384 | IP_DMC_vs_In_DMC_peak_61 | EDL933_RS02530:Promoter;EDL933_RS02535:Promoter | EDL933_RS02530:NZ_CP008957.1:522728-523352:+:-11 | EDL933_RS02530(clpP) |
| NZ_CP008957.1 | 3067722 | 3068180 | 459 | 3067880 | 496 | 9.32916 | 1.33074 | 7.79014 | IP_DMC_vs_In_DMC_peak_225 | EDL933_RS16250:Promoter;EDL933_RS16245:Promoter;EDL933_RS16260:CDS | EDL933_RS16260:NZ_CP008957.1:3067221-3068058:+:729 | EDL933_RS16260(yeiG) |
| NZ_CP008957.1 | 4627986 | 4628347 | 362 | 4628200 | 555 | 10.27665 | 1.33013 | 8.70595 | IP_DMC_vs_In_DMC_peak_409 | EDL933_RS24005:Promoter;EDL933_RS24010:Promoter;EDL933_RS24015:Promoter;EDL933_RS24000:Promoter | EDL933_RS24005:NZ_CP008957.1:4627282-4628140:-:-26 | EDL933_RS24005(yibB) |
| NZ_CP008957.1 | 3813062 | 3813439 | 378 | 3813234 | 522 | 9.72867 | 1.33003 | 8.17635 | IP_DMC_vs_In_DMC_peak_320 | EDL933_RS19890:CDS;EDL933_RS19870:Promoter;EDL933_RS19885:Promoter;EDL933_RS19880:Promoter | EDL933_RS19890:NZ_CP008957.1:3812630-3813296:-:46 | EDL933_RS19890(EDL933_RS19890) |
| NZ_CP008958.1 | 55495 | 59028 | 3534 | 57814 | 1779 | 30.0147 | 1.32863 | 28.10613 | IP_DMC_vs_In_DMC_peak_554 | EDL933_RS28880:Promoter | EDL933_RS28875:NZ_CP008958.1:56576-57548:+:685 | EDL933_RS28875(EDL933_RS28875) |
| NZ_CP008957.1 | 523214 | 523599 | 386 | 523461 | 493 | 9.17504 | 1.32822 | 7.64126 | IP_DMC_vs_In_DMC_peak_62 | EDL933_RS02535:Promoter;EDL933_RS02540:Promoter | EDL933_RS02535:NZ_CP008957.1:523477-524752:+:-71 | EDL933_RS02535(clpX) |
| NZ_CP008957.1 | 4690948 | 4692545 | 1598 | 4691643 | 586 | 10.65914 | 1.32751 | 9.07654 | IP_DMC_vs_In_DMC_peak_424 | EDL933_RS24330:Promoter;EDL933_RS24340:Promoter | EDL933_RS24340:NZ_CP008957.1:4691111-4691618:-:-128 | EDL933_RS24340(espH) |
| NZ_CP008957.1 | 379073 | 379884 | 812 | 379727 | 493 | 9.0836 | 1.326 | 7.55326 | IP_DMC_vs_In_DMC_peak_55 | EDL933_RS01855:Promoter | EDL933_RS01855:NZ_CP008957.1:379733-383717:+:-255 | EDL933_RS01855(ehaA) |
| NZ_CP008957.1 | 386265 | 386521 | 257 | 386395 | 543 | 9.83506 | 1.32474 | 8.27893 | IP_DMC_vs_In_DMC_peak_56 | EDL933_RS01880:Promoter;EDL933_RS01875:Promoter;EDL933_RS01865:Promoter;EDL933_RS01885:Promoter;EDL933_RS01870:CDS | EDL933_RS01870:NZ_CP008957.1:386011-386519:-:126 | EDL933_RS01870(EDL933_RS01870) |
| NZ_CP008957.1 | 12072 | 13575 | 1504 | 12161 | 539 | 9.77007 | 1.32472 | 8.21574 | IP_DMC_vs_In_DMC_peak_2 | EDL933_RS00065:Promoter;EDL933_RS00070:Promoter;EDL933_RS00060:Promoter;EDL933_RS00055:Promoter | EDL933_RS00070:NZ_CP008957.1:12179-14096:+:644 | EDL933_RS00070(dnaK) |
| NZ_CP008957.1 | 2367694 | 2368041 | 348 | 2367877 | 482 | 8.8472 | 1.32455 | 7.32501 | IP_DMC_vs_In_DMC_peak_181 | EDL933_RS12465:Promoter;EDL933_RS12470:Promoter;EDL933_RS12460:Promoter | EDL933_RS12465:NZ_CP008957.1:2367397-2367763:-:-104 | EDL933_RS12465(mdtJ) |
| NZ_CP008957.1 | 4799941 | 4800185 | 245 | 4800030 | 562 | 10.08617 | 1.32359 | 8.52166 | IP_DMC_vs_In_DMC_peak_448 | EDL933_RS24935:CDS;EDL933_RS24925:Promoter;EDL933_RS24930:Promoter | EDL933_RS24930:NZ_CP008957.1:4798469-4799333:-:-729 | EDL933_RS24930(atpG) |
| NZ_CP008957.1 | 3801126 | 3802002 | 877 | 3801643 | 496 | 8.99779 | 1.32274 | 7.47022 | IP_DMC_vs_In_DMC_peak_312 | EDL933_RS32710:Promoter;EDL933_RS19775:Promoter;EDL933_RS19805:Promoter;EDL933_RS19795:CDS | EDL933_RS19795:NZ_CP008957.1:3801534-3802026:+:29 | EDL933_RS19795(EDL933_RS19795) |
| NZ_CP008957.1 | 3958498 | 3960138 | 1641 | 3959363 | 606 | 10.66734 | 1.32119 | 9.08454 | IP_DMC_vs_In_DMC_peak_334 | EDL933_RS20620:CDS;EDL933_RS20630:Promoter;EDL933_RS20600:Promoter;EDL933_RS20640:Promoter | EDL933_RS20620:NZ_CP008957.1:3958801-3960340:+:516 | EDL933_RS20620(EDL933_RS20620) |
| NZ_CP008957.1 | 2979316 | 2980252 | 937 | 2979883 | 762 | 13.11225 | 1.32115 | 11.4638 | IP_DMC_vs_In_DMC_peak_219 | EDL933_RS30930:CDS;EDL933_RS15685:Promoter | EDL933_RS30930:NZ_CP008957.1:2979136-2980350:-:566 | EDL933_RS30930(EDL933_RS30930) |
| NZ_CP008957.1 | 3533174 | 3536294 | 3121 | 3533285 | 1807 | 28.92702 | 1.31812 | 27.03027 | IP_DMC_vs_In_DMC_peak_276 | EDL933_RS18415:Promoter;EDL933_RS18420:Promoter | EDL933_RS18420:NZ_CP008957.1:3533156-3533272:-:-1461 | EDL933_RS18420(rrf) |
| NZ_CP008957.1 | 3568718 | 3569533 | 816 | 3568868 | 758 | 12.83962 | 1.31776 | 11.19773 | IP_DMC_vs_In_DMC_peak_289 | EDL933_RS33715:CDS;EDL933_RS33710:Promoter;EDL933_RS18595:Promoter | EDL933_RS33715:NZ_CP008957.1:3568576-3569619:+:549 | EDL933_RS33715(EDL933_RS33715) |
| NZ_CP008957.1 | 3567339 | 3568444 | 1106 | 3567753 | 789 | 13.24863 | 1.31671 | 11.59695 | IP_DMC_vs_In_DMC_peak_288 | EDL933_RS33715:Promoter;EDL933_RS33710:Promoter;EDL933_RS18595:Promoter;EDL933_RS18610:CDS | EDL933_RS18610:NZ_CP008957.1:3567319-3568533:+:572 | EDL933_RS18610(EDL933_RS18610) |
| NZ_CP008957.1 | 2399395 | 2399981 | 587 | 2399837 | 479 | 8.47228 | 1.31632 | 6.96353 | IP_DMC_vs_In_DMC_peak_183 | EDL933_RS12615:Promoter;EDL933_RS12605:Promoter;EDL933_RS12585:Promoter;EDL933_RS12610:Promoter | EDL933_RS12605:NZ_CP008957.1:2399858-2400440:+:-170 | EDL933_RS12605(rsxA) |
| NZ_CP008957.1 | 885062 | 885402 | 341 | 885266 | 486 | 8.57723 | 1.31625 | 7.06532 | IP_DMC_vs_In_DMC_peak_97 | EDL933_RS04165:Promoter;EDL933_RS04155:Promoter;EDL933_RS04160:CDS;EDL933_RS04170:Promoter | EDL933_RS04165:NZ_CP008957.1:885530-886964:+:-298 | EDL933_RS04165(EDL933_RS04165) |
| NZ_CP008957.1 | 4293448 | 4293766 | 319 | 4293594 | 522 | 9.12451 | 1.31608 | 7.59224 | IP_DMC_vs_In_DMC_peak_376 | EDL933_RS22460:Promoter;EDL933_RS22465:Promoter;EDL933_RS22450:Promoter | EDL933_RS22460:NZ_CP008957.1:4293652-4294285:+:-45 | EDL933_RS22460(crp) |
| NZ_CP008957.1 | 5065408 | 5068551 | 3144 | 5068442 | 1913 | 30.15118 | 1.31556 | 28.24119 | IP_DMC_vs_In_DMC_peak_481 | EDL933_RS26255:Promoter;EDL933_RS26250:Promoter;EDL933_RS26245:Promoter | EDL933_RS26245:NZ_CP008957.1:5068447-5068563:+:-1468 | EDL933_RS26245(rrf) |
| NZ_CP008957.1 | 2286939 | 2287637 | 699 | 2287501 | 637 | 10.80292 | 1.31445 | 9.21613 | IP_DMC_vs_In_DMC_peak_179 | EDL933_RS11975:Promoter;EDL933_RS30570:Promoter;EDL933_RS32415:Promoter;EDL933_RS11970:CDS | EDL933_RS11970:NZ_CP008957.1:2287254-2287602:+:33 | EDL933_RS11970(tnpB) |
| NZ_CP008957.1 | 5086756 | 5086981 | 226 | 5086871 | 615 | 10.40236 | 1.31312 | 8.82768 | IP_DMC_vs_In_DMC_peak_486 | intergenic | EDL933_RS26335:NZ_CP008957.1:5086307-5086751:+:561 | EDL933_RS26335(EDL933_RS26335) |
| NZ_CP008957.1 | 4762559 | 4764328 | 1770 | 4763404 | 561 | 9.57594 | 1.31287 | 8.02864 | IP_DMC_vs_In_DMC_peak_438 | EDL933_RS24780:Promoter;EDL933_RS24775:Promoter;EDL933_RS24770:CDS | EDL933_RS24770:NZ_CP008957.1:4762198-4764368:-:925 | EDL933_RS24770(EDL933_RS24770) |
| NZ_CP008957.1 | 4767752 | 4767962 | 211 | 4767870 | 584 | 9.82797 | 1.31087 | 8.27201 | IP_DMC_vs_In_DMC_peak_439 | EDL933_RS24790:Promoter;EDL933_RS24795:Promoter | EDL933_RS24790:NZ_CP008957.1:4767947-4769123:+:-90 | EDL933_RS24790(mdtL) |
| NZ_CP008957.1 | 4941577 | 4941830 | 254 | 4941702 | 600 | 10.04094 | 1.31034 | 8.47761 | IP_DMC_vs_In_DMC_peak_470 | EDL933_RS25630:Promoter;EDL933_RS25625:Promoter;EDL933_RS25640:Promoter | EDL933_RS25640:NZ_CP008957.1:4941831-4943655:+:-128 | EDL933_RS25640(typA) |
| NZ_CP008957.1 | 17994 | 18453 | 460 | 18234 | 507 | 8.63923 | 1.30995 | 7.12474 | IP_DMC_vs_In_DMC_peak_4 | EDL933_RS00090:CDS | EDL933_RS00090:NZ_CP008957.1:17388-18288:+:835 | EDL933_RS00090(nhaR) |
| NZ_CP008958.1 | 45447 | 47302 | 1856 | 46327 | 1742 | 26.72353 | 1.30941 | 24.85089 | IP_DMC_vs_In_DMC_peak_550 | EDL933_RS28810:Promoter;EDL933_RS28790:CDS;EDL933_RS32955:Promoter;EDL933_RS34240:Promoter | EDL933_RS34240:NZ_CP008958.1:46550-47043:+:-176 | EDL933_RS34240(EDL933_RS34240) |
| NZ_CP008957.1 | 4896657 | 4896889 | 233 | 4896774 | 567 | 9.49389 | 1.30912 | 7.94891 | IP_DMC_vs_In_DMC_peak_465 | EDL933_RS25430:Promoter;EDL933_RS25425:Promoter | EDL933_RS25425:NZ_CP008957.1:4895798-4896752:-:-20 | EDL933_RS25425(metR) |
| NZ_CP008957.1 | 1414162 | 1414468 | 307 | 1414401 | 476 | 8.10527 | 1.30809 | 6.60957 | IP_DMC_vs_In_DMC_peak_145 | EDL933_RS07135:Promoter | EDL933_RS07135:NZ_CP008957.1:1414645-1415434:+:-330 | EDL933_RS07135(phoH) |
| NZ_CP008957.1 | 870253 | 870708 | 456 | 870392 | 487 | 8.26397 | 1.308 | 6.76291 | IP_DMC_vs_In_DMC_peak_95 | EDL933_RS04070:Promoter;EDL933_RS04065:Promoter | EDL933_RS04085:NZ_CP008957.1:870485-871238:-:758 | EDL933_RS04085(gpmA) |
| NZ_CP008957.1 | 502041 | 502621 | 581 | 502391 | 516 | 8.65207 | 1.30705 | 7.1372 | IP_DMC_vs_In_DMC_peak_59 | EDL933_RS02430:CDS | EDL933_RS02430:NZ_CP008957.1:501536-503108:-:777 | EDL933_RS02430(espY3) |
| NZ_CP008957.1 | 3596410 | 3597017 | 608 | 3596545 | 481 | 8.07976 | 1.30555 | 6.58544 | IP_DMC_vs_In_DMC_peak_298 | EDL933_RS18750:Promoter;EDL933_RS18760:Promoter;EDL933_RS34185:CDS | EDL933_RS34185:NZ_CP008957.1:3596203-3598456:+:510 | EDL933_RS34185(EDL933_RS34185) |
| NZ_CP008957.1 | 4802834 | 4803509 | 676 | 4803241 | 551 | 9.0602 | 1.3047 | 7.53053 | IP_DMC_vs_In_DMC_peak_452 | EDL933_RS24955:Promoter;EDL933_RS24950:Promoter;EDL933_RS24940:Promoter;EDL933_RS24960:CDS;EDL933_RS24945:Promoter | EDL933_RS24955:NZ_CP008957.1:4802303-4803119:-:-52 | EDL933_RS24955(atpB) |
| NZ_CP008957.1 | 3861881 | 3862243 | 363 | 3862113 | 523 | 8.63926 | 1.30432 | 7.12476 | IP_DMC_vs_In_DMC_peak_324 | EDL933_RS20095:Promoter;EDL933_RS20090:Promoter | EDL933_RS20095:NZ_CP008957.1:3860958-3862057:-:-4 | EDL933_RS20095(prfB) |
| NZ_CP008957.1 | 4042620 | 4042870 | 251 | 4042780 | 506 | 8.38451 | 1.3041 | 6.87919 | IP_DMC_vs_In_DMC_peak_341 | EDL933_RS21070:exon;EDL933_RS21080:Promoter;EDL933_RS21065:Promoter | EDL933_RS21070:NZ_CP008957.1:4042755-4042831:+:-10 | EDL933_RS21070(EDL933_RS21070) |
| NZ_CP008957.1 | 4885996 | 4887043 | 1048 | 4886111 | 536 | 8.81643 | 1.30408 | 7.29582 | IP_DMC_vs_In_DMC_peak_464 | EDL933_RS25360:Promoter;EDL933_RS25370:CDS;EDL933_RS25365:Promoter;EDL933_RS25375:Promoter | EDL933_RS25375:NZ_CP008957.1:4886880-4887180:+:-361 | EDL933_RS25375(EDL933_RS25375) |
| NZ_CP008957.1 | 1702296 | 1702538 | 243 | 1702420 | 474 | 7.865 | 1.30261 | 6.37801 | IP_DMC_vs_In_DMC_peak_162 | EDL933_RS08780:Promoter;EDL933_RS08790:Promoter;EDL933_RS08785:Promoter;EDL933_RS08775:Promoter | EDL933_RS08790:NZ_CP008957.1:1702423-1703674:+:-6 | EDL933_RS08790(icd) |
| NZ_CP008957.1 | 4674151 | 4674508 | 358 | 4674316 | 607 | 9.71003 | 1.30144 | 8.15824 | IP_DMC_vs_In_DMC_peak_417 | EDL933_RS24260:Promoter;EDL933_RS24255:CDS | EDL933_RS24260:NZ_CP008957.1:4674477-4676016:+:-148 | EDL933_RS24260(EDL933_RS24260) |
| NZ_CP008957.1 | 996937 | 997246 | 310 | 997015 | 482 | 7.93085 | 1.30135 | 6.44183 | IP_DMC_vs_In_DMC_peak_109 | EDL933_RS04770:Promoter | EDL933_RS04770:NZ_CP008957.1:997083-999432:+:8 | EDL933_RS04770(EDL933_RS04770) |
| NZ_CP008957.1 | 5129385 | 5129643 | 259 | 5129553 | 541 | 8.75813 | 1.30108 | 7.23941 | IP_DMC_vs_In_DMC_peak_491 | EDL933_RS26505:Promoter;EDL933_RS26515:Promoter;EDL933_RS26510:Promoter;EDL933_RS26520:CDS | EDL933_RS26515:NZ_CP008957.1:5128667-5129465:-:-48 | EDL933_RS26515(EDL933_RS26515) |
| NZ_CP008957.1 | 5398300 | 5399384 | 1085 | 5398463 | 749 | 11.65516 | 1.30039 | 10.04376 | IP_DMC_vs_In_DMC_peak_515 | EDL933_RS27825:CDS | EDL933_RS27825:NZ_CP008957.1:5398206-5399420:+:635 | EDL933_RS27825(EDL933_RS27825) |
| NZ_CP008957.1 | 790308 | 790628 | 321 | 790525 | 473 | 7.74619 | 1.29987 | 6.26368 | IP_DMC_vs_In_DMC_peak_88 | EDL933_RS03680:CDS;EDL933_RS03675:Promoter | EDL933_RS03675:NZ_CP008957.1:790000-790447:-:-20 | EDL933_RS03675(fur) |
| NZ_CP008957.1 | 3563626 | 3564171 | 546 | 3564054 | 506 | 8.20071 | 1.29958 | 6.70214 | IP_DMC_vs_In_DMC_peak_286 | EDL933_RS18585:Promoter;EDL933_RS18580:CDS;EDL933_RS29085:Promoter;EDL933_RS18590:Promoter | EDL933_RS29085:NZ_CP008957.1:3563485-3563638:-:-260 | EDL933_RS29085(EDL933_RS29085) |
| NZ_CP008957.1 | 3897747 | 3898028 | 282 | 3897838 | 519 | 8.37521 | 1.29937 | 6.87013 | IP_DMC_vs_In_DMC_peak_328 | EDL933_RS20265:Promoter;EDL933_RS20280:Promoter;EDL933_RS20275:Promoter;EDL933_RS20260:Promoter;EDL933_RS20270:CDS | EDL933_RS20265:NZ_CP008957.1:3896560-3897724:-:-163 | EDL933_RS20265(pgk) |
| NZ_CP008957.1 | 3949911 | 3950749 | 839 | 3950078 | 501 | 8.098 | 1.29878 | 6.60313 | IP_DMC_vs_In_DMC_peak_330 | EDL933_RS20575:Promoter;EDL933_RS20570:CDS;EDL933_RS33775:Promoter | EDL933_RS33775:NZ_CP008957.1:3950849-3950999:+:-519 | EDL933_RS33775(EDL933_RS33775) |
| NZ_CP008957.1 | 3445745 | 3446099 | 355 | 3445978 | 474 | 7.71203 | 1.2986 | 6.23131 | IP_DMC_vs_In_DMC_peak_268 | EDL933_RS18020:Promoter;EDL933_RS18015:Promoter | EDL933_RS18020:NZ_CP008957.1:3444862-3445876:-:-45 | EDL933_RS18020(rodZ) |
| NZ_CP008957.1 | 4710373 | 4711615 | 1243 | 4711532 | 531 | 8.45107 | 1.29718 | 6.94367 | IP_DMC_vs_In_DMC_peak_431 | EDL933_RS34215:Promoter;EDL933_RS24485:Promoter | EDL933_RS24470:NZ_CP008957.1:4710367-4711186:+:626 | EDL933_RS24470(EDL933_RS24470) |
| NZ_CP008957.1 | 94735 | 95053 | 319 | 94898 | 499 | 7.93723 | 1.29545 | 6.44802 | IP_DMC_vs_In_DMC_peak_13 | EDL933_RS00450:Promoter;EDL933_RS00445:CDS;EDL933_RS00455:Promoter | EDL933_RS00445:NZ_CP008957.1:94699-95641:+:194 | EDL933_RS00445(rsmH) |
| NZ_CP008957.1 | 4676578 | 4676841 | 264 | 4676660 | 554 | 8.68937 | 1.29534 | 7.17341 | IP_DMC_vs_In_DMC_peak_419 | intergenic | EDL933_RS33850:NZ_CP008957.1:4676333-4676649:+:376 | EDL933_RS33850(EDL933_RS33850) |
| NZ_CP008957.1 | 4707563 | 4708299 | 737 | 4707794 | 522 | 8.22274 | 1.29469 | 6.7236 | IP_DMC_vs_In_DMC_peak_429 | EDL933_RS24440:Promoter;EDL933_RS24465:Promoter;EDL933_RS24445:Promoter;EDL933_RS24455:Promoter;EDL933_RS24460:Promoter;EDL933_RS24450:Promoter | EDL933_RS24460:NZ_CP008957.1:4707409-4707781:-:-149 | EDL933_RS24460(ler) |
| NZ_CP008957.1 | 1591216 | 1591626 | 411 | 1591459 | 471 | 7.51124 | 1.29438 | 6.03756 | IP_DMC_vs_In_DMC_peak_155 | EDL933_RS08055:Promoter;EDL933_RS08065:Promoter | EDL933_RS08055:NZ_CP008957.1:1588022-1591208:-:-212 | EDL933_RS08055(rne) |
| NZ_CP008957.1 | 3607455 | 3607749 | 295 | 3607592 | 491 | 7.77292 | 1.29406 | 6.28957 | IP_DMC_vs_In_DMC_peak_300 | EDL933_RS18830:Promoter;EDL933_RS18820:CDS;EDL933_RS18810:Promoter;EDL933_RS18805:Promoter | EDL933_RS18820:NZ_CP008957.1:3607327-3607846:+:274 | EDL933_RS18820(ygaP) |
| NZ_CP008957.1 | 2287904 | 2289105 | 1202 | 2288150 | 602 | 9.23049 | 1.29288 | 7.69434 | IP_DMC_vs_In_DMC_peak_180 | EDL933_RS32415:Promoter;EDL933_RS11975:CDS | EDL933_RS11975:NZ_CP008957.1:2287651-2289190:+:853 | EDL933_RS11975(EDL933_RS11975) |
| NZ_CP008957.1 | 2186066 | 2187114 | 1049 | 2186449 | 762 | 11.31754 | 1.29171 | 9.71544 | IP_DMC_vs_In_DMC_peak_176 | EDL933_RS11460:Promoter;EDL933_RS11450:CDS;EDL933_RS30520:Promoter | EDL933_RS11450:NZ_CP008957.1:2185972-2187186:+:617 | EDL933_RS11450(EDL933_RS11450) |
| NZ_CP008957.1 | 3560429 | 3560728 | 300 | 3560502 | 472 | 7.33104 | 1.2892 | 5.86482 | IP_DMC_vs_In_DMC_peak_284 | EDL933_RS29255:Promoter;EDL933_RS18570:Promoter;EDL933_RS18555:Promoter | EDL933_RS18555:NZ_CP008957.1:3560502-3560844:+:76 | EDL933_RS18555(bamE) |
| NZ_CP008957.1 | 3583987 | 3585202 | 1216 | 3585028 | 479 | 7.41082 | 1.28884 | 5.94116 | IP_DMC_vs_In_DMC_peak_295 | EDL933_RS18715:Promoter;EDL933_RS18725:CDS;EDL933_RS18720:Promoter | EDL933_RS18720:NZ_CP008957.1:3583517-3584831:-:237 | EDL933_RS18720(EDL933_RS18720) |
| NZ_CP008957.1 | 551662 | 551887 | 226 | 551775 | 483 | 7.4406 | 1.28823 | 5.96997 | IP_DMC_vs_In_DMC_peak_67 | EDL933_RS02660:Promoter;EDL933_RS02655:Promoter;EDL933_RS02665:Promoter;EDL933_RS02650:Promoter | EDL933_RS02660:NZ_CP008957.1:551825-552473:+:-51 | EDL933_RS02660(acrR) |
| NZ_CP008957.1 | 4120975 | 4122039 | 1065 | 4121340 | 760 | 11.04087 | 1.28748 | 9.44686 | IP_DMC_vs_In_DMC_peak_348 | EDL933_RS21465:Promoter;EDL933_RS21460:CDS | EDL933_RS21460:NZ_CP008957.1:4120886-4122100:+:620 | EDL933_RS21460(EDL933_RS21460) |
| NZ_CP008957.1 | 4389735 | 4390277 | 543 | 4390110 | 512 | 7.76469 | 1.28675 | 6.28157 | IP_DMC_vs_In_DMC_peak_383 | intergenic | EDL933_RS22890:NZ_CP008957.1:4388718-4389630:+:1287 | EDL933_RS22890(EDL933_RS22890) |
| NZ_CP008957.1 | 2787983 | 2789194 | 1212 | 2788410 | 777 | 11.21798 | 1.28674 | 9.61907 | IP_DMC_vs_In_DMC_peak_203 | EDL933_RS14760:CDS;EDL933_RS14750:Promoter;EDL933_RS14745:Promoter;EDL933_RS14740:Promoter | EDL933_RS14760:NZ_CP008957.1:2787994-2789208:+:594 | EDL933_RS14760(EDL933_RS14760) |
| NZ_CP008957.1 | 620492 | 620769 | 278 | 620617 | 468 | 7.16548 | 1.28615 | 5.70571 | IP_DMC_vs_In_DMC_peak_71 | EDL933_RS02870:CDS;EDL933_RS32000:Promoter | EDL933_RS32000:NZ_CP008957.1:620924-621185:+:-294 | EDL933_RS32000(EDL933_RS32000) |
| NZ_CP008957.1 | 5306356 | 5306773 | 418 | 5306564 | 525 | 7.89425 | 1.28576 | 6.40634 | IP_DMC_vs_In_DMC_peak_506 | EDL933_RS27370:Promoter;EDL933_RS27365:Promoter;EDL933_RS27360:CDS | EDL933_RS27365:NZ_CP008957.1:5306977-5307175:+:-413 | EDL933_RS27365(EDL933_RS27365) |
| NZ_CP008957.1 | 4979230 | 4979855 | 626 | 4979770 | 526 | 7.88963 | 1.28533 | 6.40185 | IP_DMC_vs_In_DMC_peak_473 | EDL933_RS25825:CDS;EDL933_RS25820:Promoter;EDL933_RS25815:Promoter | EDL933_RS25825:NZ_CP008957.1:4979550-4980945:+:-8 | EDL933_RS25825(EDL933_RS25825) |
| NZ_CP008958.1 | 26236 | 26807 | 572 | 26624 | 1757 | 23.42556 | 1.28359 | 21.59567 | IP_DMC_vs_In_DMC_peak_544 | EDL933_RS28705:Promoter;EDL933_RS28700:CDS | EDL933_RS28705:NZ_CP008958.1:26673-28641:+:-152 | EDL933_RS28705(etpD) |
| NZ_CP008957.1 | 3105611 | 3105937 | 327 | 3105752 | 467 | 7.05201 | 1.28341 | 5.59682 | IP_DMC_vs_In_DMC_peak_231 | EDL933_RS16435:Promoter;EDL933_RS16440:Promoter;EDL933_RS16425:Promoter | EDL933_RS16425:NZ_CP008957.1:3105765-3106050:+:8 | EDL933_RS16425(rplY) |
| NZ_CP008957.1 | 1748648 | 1749732 | 1085 | 1748977 | 786 | 11.09218 | 1.28273 | 9.49701 | IP_DMC_vs_In_DMC_peak_165 | EDL933_RS09085:Promoter;EDL933_RS09065:Promoter;EDL933_RS09080:CDS | EDL933_RS09080:NZ_CP008957.1:1748573-1749787:+:616 | EDL933_RS09080(EDL933_RS09080) |
| NZ_CP008957.1 | 4526384 | 4526663 | 280 | 4526538 | 544 | 8.00408 | 1.28251 | 6.51215 | IP_DMC_vs_In_DMC_peak_401 | EDL933_RS23560:CDS;EDL933_RS23570:Promoter;EDL933_RS23555:Promoter;EDL933_RS23565:Promoter;EDL933_RS23545:Promoter;EDL933_RS23540:Promoter | EDL933_RS23560:NZ_CP008957.1:4526227-4527799:+:296 | EDL933_RS23560(bcsE) |
| NZ_CP008957.1 | 4635622 | 4635927 | 306 | 4635735 | 524 | 7.69406 | 1.28117 | 6.21389 | IP_DMC_vs_In_DMC_peak_413 | EDL933_RS24035:Promoter;EDL933_RS24040:Promoter;EDL933_RS24045:CDS;EDL933_RS24030:Promoter | EDL933_RS24040:NZ_CP008957.1:4634857-4635565:-:-209 | EDL933_RS24040(rfaY) |
| NZ_CP008957.1 | 3346658 | 3347306 | 649 | 3347075 | 468 | 6.97844 | 1.28104 | 5.52659 | IP_DMC_vs_In_DMC_peak_260 | EDL933_RS17565:Promoter;EDL933_RS17555:Promoter;EDL933_RS17550:CDS | EDL933_RS17555:NZ_CP008957.1:3347115-3347625:+:-133 | EDL933_RS17555(crr) |
| NZ_CP008957.1 | 1255493 | 1255751 | 259 | 1255620 | 466 | 6.93944 | 1.28067 | 5.48894 | IP_DMC_vs_In_DMC_peak_128 | EDL933_RS06160:Promoter;EDL933_RS06125:Promoter;EDL933_RS06140:CDS;EDL933_RS06130:Promoter;EDL933_RS06135:Promoter;EDL933_RS06120:Promoter;EDL933_RS06145:Promoter;EDL933_RS06150:Promoter | EDL933_RS06140:NZ_CP008957.1:1255292-1255694:-:72 | EDL933_RS06140(EDL933_RS06140) |
| NZ_CP008957.1 | 699444 | 699740 | 297 | 699533 | 466 | 6.93944 | 1.28067 | 5.48894 | IP_DMC_vs_In_DMC_peak_79 | EDL933_RS03210:Promoter | EDL933_RS03210:NZ_CP008957.1:699687-700821:+:-95 | EDL933_RS03210(fepE) |
| NZ_CP008957.1 | 5273490 | 5275742 | 2253 | 5273966 | 533 | 7.7548 | 1.27987 | 6.27202 | IP_DMC_vs_In_DMC_peak_500 | EDL933_RS27190:Promoter;EDL933_RS27195:Promoter;EDL933_RS27200:Promoter | EDL933_RS27200:NZ_CP008957.1:5274341-5275988:+:274 | EDL933_RS27200(groL) |
| NZ_CP008957.1 | 4800594 | 4800852 | 259 | 4800673 | 540 | 7.81776 | 1.27927 | 6.33294 | IP_DMC_vs_In_DMC_peak_449 | EDL933_RS24935:CDS;EDL933_RS24930:Promoter | EDL933_RS24935:NZ_CP008957.1:4799383-4800925:-:202 | EDL933_RS24935(atpA) |
| NZ_CP008957.1 | 3569954 | 3570364 | 411 | 3570220 | 619 | 8.76342 | 1.27838 | 7.24449 | IP_DMC_vs_In_DMC_peak_290 | EDL933_RS18630:CDS;EDL933_RS34175:Promoter | EDL933_RS34175:NZ_CP008957.1:3569662-3570070:-:-88 | EDL933_RS34175(EDL933_RS34175) |
| NZ_CP008957.1 | 146255 | 147103 | 849 | 146979 | 513 | 7.43676 | 1.2782 | 5.96626 | IP_DMC_vs_In_DMC_peak_20 | EDL933_RS00675:Promoter;EDL933_RS00655:Promoter;EDL933_RS00665:CDS;EDL933_RS00670:Promoter;EDL933_RS00680:Promoter | EDL933_RS00665:NZ_CP008957.1:146380-147043:-:364 | EDL933_RS00665(can) |
| NZ_CP008957.1 | 2398667 | 2398978 | 312 | 2398879 | 465 | 6.82777 | 1.27793 | 5.38192 | IP_DMC_vs_In_DMC_peak_182 | EDL933_RS12605:Promoter;EDL933_RS12600:Promoter;EDL933_RS12595:Promoter;EDL933_RS12585:Promoter;EDL933_RS12610:Promoter | EDL933_RS12590:NZ_CP008957.1:2398642-2398768:+:180 | EDL933_RS12590(blr) |
| NZ_CP008957.1 | 697949 | 699214 | 1266 | 698316 | 465 | 6.82777 | 1.27793 | 5.38192 | IP_DMC_vs_In_DMC_peak_78 | EDL933_RS03205:CDS;EDL933_RS03210:Promoter | EDL933_RS03210:NZ_CP008957.1:699687-700821:+:-1106 | EDL933_RS03210(fepE) |
| NZ_CP008957.1 | 5092534 | 5092871 | 338 | 5092651 | 571 | 8.11961 | 1.27731 | 6.62352 | IP_DMC_vs_In_DMC_peak_487 | EDL933_RS26365:Promoter;EDL933_RS26360:Promoter;EDL933_RS26375:Promoter;EDL933_RS26380:Promoter | EDL933_RS26365:NZ_CP008957.1:5090654-5092550:-:-152 | EDL933_RS26365(thiC) |
| NZ_CP008957.1 | 4774764 | 4774997 | 234 | 4774896 | 528 | 7.5612 | 1.27666 | 6.086 | IP_DMC_vs_In_DMC_peak_441 | EDL933_RS24825:Promoter;EDL933_RS24820:CDS;EDL933_RS24810:Promoter | EDL933_RS24820:NZ_CP008957.1:4773909-4776318:-:1438 | EDL933_RS24820(espY4) |
| NZ_CP008957.1 | 3810043 | 3810791 | 749 | 3810248 | 488 | 7.02905 | 1.27565 | 5.57549 | IP_DMC_vs_In_DMC_peak_318 | EDL933_RS19865:CDS;EDL933_RS34195:Promoter | EDL933_RS19865:NZ_CP008957.1:3810133-3810400:+:283 | EDL933_RS19865(EDL933_RS19865) |
| NZ_CP008957.1 | 4632233 | 4632627 | 395 | 4632457 | 570 | 8.01653 | 1.27523 | 6.5242 | IP_DMC_vs_In_DMC_peak_411 | EDL933_RS24025:CDS | EDL933_RS24025:NZ_CP008957.1:4631424-4632633:+:1005 | EDL933_RS24025(rfaL) |
| NZ_CP008957.1 | 2866183 | 2867539 | 1357 | 2866306 | 464 | 6.71699 | 1.27518 | 5.27608 | IP_DMC_vs_In_DMC_peak_210 | EDL933_RS15190:CDS;EDL933_RS15185:Promoter | EDL933_RS15195:NZ_CP008957.1:2866571-2867081:-:220 | EDL933_RS15195(EDL933_RS15195) |
| NZ_CP008957.1 | 2621621 | 2622006 | 386 | 2621769 | 464 | 6.71699 | 1.27518 | 5.27608 | IP_DMC_vs_In_DMC_peak_193 | EDL933_RS13775:Promoter;EDL933_RS13755:Promoter;EDL933_RS13760:Promoter;EDL933_RS13750:Promoter;EDL933_RS13770:CDS | EDL933_RS13770:NZ_CP008957.1:2621732-2622389:+:81 | EDL933_RS13770(EDL933_RS13770) |
| NZ_CP008957.1 | 152452 | 152781 | 330 | 152581 | 513 | 7.29153 | 1.27452 | 5.8267 | IP_DMC_vs_In_DMC_peak_21 | EDL933_RS00700:CDS;EDL933_RS00690:Promoter | EDL933_RS00700:NZ_CP008957.1:152535-153387:-:771 | EDL933_RS00700(panC) |
| NZ_CP008957.1 | 705909 | 706248 | 340 | 705975 | 463 | 6.60712 | 1.27244 | 5.17112 | IP_DMC_vs_In_DMC_peak_81 | EDL933_RS03240:Promoter;EDL933_RS03235:Promoter;EDL933_RS03245:Promoter | EDL933_RS03240:NZ_CP008957.1:706132-707308:+:-54 | EDL933_RS03240(entC) |
| NZ_CP008957.1 | 173916 | 174312 | 397 | 174023 | 487 | 6.8574 | 1.27138 | 5.41058 | IP_DMC_vs_In_DMC_peak_23 | EDL933_RS00805:Promoter;EDL933_RS00795:Promoter;EDL933_RS00790:CDS;EDL933_RS00800:Promoter | EDL933_RS00795:NZ_CP008957.1:174100-174898:+:13 | EDL933_RS00795(fhuC) |
| NZ_CP008957.1 | 3807107 | 3807345 | 239 | 3807260 | 525 | 7.26214 | 1.27013 | 5.79909 | IP_DMC_vs_In_DMC_peak_316 | EDL933_RS19830:Promoter;EDL933_RS19820:Promoter;EDL933_RS19840:CDS;EDL933_RS31165:Promoter;EDL933_RS34190:Promoter | EDL933_RS19840:NZ_CP008957.1:3806829-3807564:-:338 | EDL933_RS19840(EDL933_RS19840) |
| NZ_CP008957.1 | 4704563 | 4707324 | 2762 | 4705123 | 550 | 7.55078 | 1.26994 | 6.07592 | IP_DMC_vs_In_DMC_peak_428 | EDL933_RS24430:Promoter;EDL933_RS24425:Promoter;EDL933_RS24420:Promoter;EDL933_RS24435:CDS | EDL933_RS24440:NZ_CP008957.1:4705613-4706267:-:324 | EDL933_RS24440(espL) |
| NZ_CP008957.1 | 193066 | 193354 | 289 | 193212 | 505 | 6.99307 | 1.2693 | 5.5407 | IP_DMC_vs_In_DMC_peak_25 | EDL933_RS00880:Promoter;EDL933_RS00865:Promoter;EDL933_RS00870:Promoter;EDL933_RS00875:Promoter | EDL933_RS00875:NZ_CP008957.1:193360-194086:+:-150 | EDL933_RS00875(rpsB) |
| NZ_CP008958.1 | 20986 | 22008 | 1023 | 21223 | 1696 | 20.63821 | 1.26762 | 18.85036 | IP_DMC_vs_In_DMC_peak_541 | EDL933_RS28695:Promoter;EDL933_RS33935:Promoter;EDL933_RS28680:CDS | EDL933_RS28680:NZ_CP008958.1:21134-22133:+:362 | EDL933_RS28680(lpxM) |
| NZ_CP008957.1 | 1827289 | 1827579 | 291 | 1827461 | 461 | 6.39007 | 1.26696 | 4.96402 | IP_DMC_vs_In_DMC_peak_167 | EDL933_RS09470:Promoter;EDL933_RS09475:Promoter;EDL933_RS09480:Promoter | EDL933_RS09470:NZ_CP008957.1:1824674-1827350:-:-83 | EDL933_RS09470(adhE) |
| NZ_CP008957.1 | 1180377 | 1180930 | 554 | 1180520 | 461 | 6.39007 | 1.26696 | 4.96402 | IP_DMC_vs_In_DMC_peak_121 | EDL933_RS05775:Promoter;EDL933_RS05780:Promoter;EDL933_RS05770:CDS | EDL933_RS05775:NZ_CP008957.1:1180802-1181087:+:-149 | EDL933_RS05775(ihfB) |
| NZ_CP008957.1 | 4096023 | 4096288 | 266 | 4096214 | 482 | 6.61382 | 1.26637 | 5.1776 | IP_DMC_vs_In_DMC_peak_346 | EDL933_RS21325:CDS;EDL933_RS21310:Promoter | EDL933_RS21325:NZ_CP008957.1:4095670-4096858:+:485 | EDL933_RS21325(EDL933_RS21325) |
| NZ_CP008957.1 | 3597987 | 3598529 | 543 | 3598301 | 485 | 6.64141 | 1.26617 | 5.20413 | IP_DMC_vs_In_DMC_peak_299 | EDL933_RS18780:Promoter;EDL933_RS18775:Promoter;EDL933_RS34185:CDS | EDL933_RS18775:NZ_CP008957.1:3598792-3599770:+:-534 | EDL933_RS18775(csiD) |
| NZ_CP008957.1 | 3809299 | 3809698 | 400 | 3809562 | 482 | 6.58505 | 1.26558 | 5.15067 | IP_DMC_vs_In_DMC_peak_317 | EDL933_RS19840:Promoter;EDL933_RS34195:Promoter;EDL933_RS19865:Promoter;EDL933_RS19850:Promoter;EDL933_RS19860:Promoter;EDL933_RS19845:Promoter | EDL933_RS19860:NZ_CP008957.1:3809603-3810104:+:-105 | EDL933_RS19860(EDL933_RS19860) |
| NZ_CP008957.1 | 360851 | 361240 | 390 | 361146 | 485 | 6.59486 | 1.2649 | 5.16016 | IP_DMC_vs_In_DMC_peak_50 | EDL933_RS01770:Promoter;EDL933_RS01760:CDS;EDL933_RS01755:Promoter;EDL933_RS01745:Promoter | EDL933_RS01755:NZ_CP008957.1:360440-361034:-:-11 | EDL933_RS01755(rclC) |
| NZ_CP008957.1 | 4801819 | 4802097 | 279 | 4801958 | 529 | 7.06809 | 1.26408 | 5.61234 | IP_DMC_vs_In_DMC_peak_451 | EDL933_RS24940:Promoter;EDL933_RS24945:Promoter;EDL933_RS24935:Promoter | EDL933_RS24945:NZ_CP008957.1:4801485-4801956:-:-1 | EDL933_RS24945(atpF) |
| NZ_CP008957.1 | 5342466 | 5343000 | 535 | 5342833 | 520 | 6.96077 | 1.26396 | 5.50954 | IP_DMC_vs_In_DMC_peak_511 | EDL933_RS27570:CDS;EDL933_RS27560:Promoter;EDL933_RS27580:Promoter | EDL933_RS27570:NZ_CP008957.1:5342035-5342974:+:697 | EDL933_RS27570(EDL933_RS27570) |
| NZ_CP008958.1 | 22911 | 23286 | 376 | 23102 | 1743 | 20.53029 | 1.26255 | 18.74373 | IP_DMC_vs_In_DMC_peak_542 | EDL933_RS28695:CDS;EDL933_RS34065:Promoter | EDL933_RS28695:NZ_CP008958.1:23014-25711:+:84 | EDL933_RS28695(stcE) |
| NZ_CP008957.1 | 2821868 | 2822103 | 236 | 2822048 | 459 | 6.17663 | 1.26147 | 4.76057 | IP_DMC_vs_In_DMC_peak_204 | EDL933_RS14960:Promoter;EDL933_RS14945:Promoter | EDL933_RS14945:NZ_CP008957.1:2821959-2822035:-:50 | EDL933_RS14945(EDL933_RS14945) |
| NZ_CP008957.1 | 4010171 | 4010382 | 212 | 4010318 | 485 | 6.43505 | 1.26051 | 5.00725 | IP_DMC_vs_In_DMC_peak_337 | EDL933_RS20890:Promoter;EDL933_RS20895:CDS;EDL933_RS20885:Promoter;EDL933_RS20910:Promoter | EDL933_RS20895:NZ_CP008957.1:4009726-4010554:-:278 | EDL933_RS20895(cpdA) |
| NZ_CP008957.1 | 318479 | 318757 | 279 | 318643 | 480 | 6.36605 | 1.26015 | 4.94188 | IP_DMC_vs_In_DMC_peak_45 | EDL933_RS01580:Promoter;EDL933_RS01570:Promoter;EDL933_RS01565:CDS;EDL933_RS01575:Promoter;EDL933_RS01550:Promoter | EDL933_RS01565:NZ_CP008957.1:318446-319004:+:171 | EDL933_RS01565(EDL933_RS01565) |
| NZ_CP008957.1 | 4279917 | 4280280 | 364 | 4280101 | 481 | 6.34468 | 1.25925 | 4.92131 | IP_DMC_vs_In_DMC_peak_372 | EDL933_RS22365:CDS;EDL933_RS22360:Promoter | EDL933_RS22365:NZ_CP008957.1:4278932-4281047:-:949 | EDL933_RS22365(fusA) |
| NZ_CP008957.1 | 3581925 | 3582365 | 441 | 3582173 | 458 | 6.07128 | 1.25873 | 4.66034 | IP_DMC_vs_In_DMC_peak_293 | EDL933_RS18705:Promoter;EDL933_RS18700:Promoter;EDL933_RS18715:CDS;EDL933_RS18710:Promoter | EDL933_RS18710:NZ_CP008957.1:3581490-3581889:-:-255 | EDL933_RS18710(EDL933_RS18710) |
| NZ_CP008957.1 | 2947077 | 2947298 | 222 | 2947209 | 458 | 6.07128 | 1.25873 | 4.66034 | IP_DMC_vs_In_DMC_peak_216 | EDL933_RS15555:Promoter;EDL933_RS15540:Promoter;EDL933_RS15545:Promoter;EDL933_RS15530:Promoter;EDL933_RS15550:Promoter;EDL933_RS15535:Promoter | EDL933_RS15540:NZ_CP008957.1:2946352-2947141:-:-46 | EDL933_RS15540(thiM) |
| NZ_CP008957.1 | 4207230 | 4207598 | 369 | 4207475 | 508 | 6.61607 | 1.25849 | 5.17974 | IP_DMC_vs_In_DMC_peak_359 | EDL933_RS21895:Promoter;EDL933_RS21900:Promoter;EDL933_RS21905:Promoter | EDL933_RS21900:NZ_CP008957.1:4207566-4208037:+:-152 | EDL933_RS21900(argR) |
| NZ_CP008957.1 | 557878 | 558147 | 270 | 558056 | 467 | 6.15641 | 1.25831 | 4.74204 | IP_DMC_vs_In_DMC_peak_69 | EDL933_RS02695:Promoter;EDL933_RS02670:Promoter;EDL933_RS02690:CDS;EDL933_RS02675:Promoter | EDL933_RS02690:NZ_CP008957.1:557986-559918:+:26 | EDL933_RS02690(dnaX) |
| NZ_CP008957.1 | 691031 | 691891 | 861 | 691342 | 494 | 6.44874 | 1.25815 | 5.02042 | IP_DMC_vs_In_DMC_peak_75 | EDL933_RS03180:CDS;EDL933_RS03160:Promoter | EDL933_RS03180:NZ_CP008957.1:690892-691513:-:52 | EDL933_RS03180(entD) |
| NZ_CP008957.1 | 117677 | 118037 | 361 | 117852 | 495 | 6.44164 | 1.25766 | 5.01357 | IP_DMC_vs_In_DMC_peak_15 | EDL933_RS00540:Promoter;EDL933_RS00550:Promoter;EDL933_RS00545:Promoter;EDL933_RS00555:Promoter | EDL933_RS00555:NZ_CP008957.1:117933-118977:+:-76 | EDL933_RS00555(guaC) |
| NZ_CP008957.1 | 19707 | 20530 | 824 | 19993 | 494 | 6.41987 | 1.25736 | 4.99266 | IP_DMC_vs_In_DMC_peak_6 | EDL933_RS00105:CDS;EDL933_RS32985:Promoter | EDL933_RS32985:NZ_CP008957.1:18325-19286:-:-832 | EDL933_RS32985(EDL933_RS32985) |
| NZ_CP008957.1 | 321973 | 322670 | 698 | 322387 | 489 | 6.36111 | 1.25725 | 4.93711 | IP_DMC_vs_In_DMC_peak_46 | EDL933_RS01585:CDS | EDL933_RS01585:NZ_CP008957.1:322024-322981:-:660 | EDL933_RS01585(EDL933_RS01585) |
| NZ_CP008957.1 | 3202054 | 3202277 | 224 | 3202157 | 457 | 5.96683 | 1.25599 | 4.56132 | IP_DMC_vs_In_DMC_peak_241 | EDL933_RS16850:Promoter;EDL933_RS32635:CDS | EDL933_RS32635:NZ_CP008957.1:3201866-3202484:+:299 | EDL933_RS32635(EDL933_RS32635) |
| NZ_CP008957.1 | 3107346 | 3107628 | 283 | 3107537 | 457 | 5.96683 | 1.25599 | 4.56132 | IP_DMC_vs_In_DMC_peak_232 | EDL933_RS16440:Promoter;EDL933_RS16445:Promoter;EDL933_RS16430:Promoter;EDL933_RS16435:CDS | EDL933_RS16435:NZ_CP008957.1:3107377-3107605:+:109 | EDL933_RS16435(EDL933_RS16435) |
| NZ_CP008957.1 | 2638125 | 2638335 | 211 | 2638184 | 457 | 5.96683 | 1.25599 | 4.56132 | IP_DMC_vs_In_DMC_peak_194 | EDL933_RS13845:Promoter;EDL933_RS13860:Promoter;EDL933_RS13855:Promoter;EDL933_RS13850:CDS;EDL933_RS13840:Promoter | EDL933_RS13845:NZ_CP008957.1:2636605-2637928:-:-301 | EDL933_RS13845(mepM) |
| NZ_CP008957.1 | 5343678 | 5343927 | 250 | 5343768 | 518 | 6.56958 | 1.2544 | 5.13579 | IP_DMC_vs_In_DMC_peak_512 | EDL933_RS27575:Promoter;EDL933_RS27580:Promoter | EDL933_RS27580:NZ_CP008957.1:5343915-5344122:+:-113 | EDL933_RS27580(EDL933_RS27580) |
| NZ_CP008957.1 | 2711750 | 2712284 | 535 | 2711912 | 456 | 5.86329 | 1.25325 | 4.46322 | IP_DMC_vs_In_DMC_peak_201 | EDL933_RS33560:Promoter;EDL933_RS14275:Promoter;EDL933_RS14290:Promoter;EDL933_RS14280:CDS | EDL933_RS14280:NZ_CP008957.1:2711435-2712092:-:75 | EDL933_RS14280(uvrY) |
| NZ_CP008957.1 | 1468959 | 1469575 | 617 | 1469425 | 557 | 6.91234 | 1.25254 | 5.46365 | IP_DMC_vs_In_DMC_peak_151 | EDL933_RS07340:CDS;EDL933_RS34115:Promoter;EDL933_RS07360:Promoter;EDL933_RS33240:Promoter | EDL933_RS07340:NZ_CP008957.1:1468352-1469891:-:624 | EDL933_RS07340(EDL933_RS07340) |
| NZ_CP008957.1 | 3621330 | 3621617 | 288 | 3621433 | 466 | 5.92776 | 1.25201 | 4.52487 | IP_DMC_vs_In_DMC_peak_302 | EDL933_RS18905:Promoter;EDL933_RS18900:Promoter;EDL933_RS18910:Promoter;EDL933_RS18895:CDS | EDL933_RS18895:NZ_CP008957.1:3621395-3622133:+:78 | EDL933_RS18895(ygaZ) |
| NZ_CP008957.1 | 4680885 | 4681216 | 332 | 4680977 | 532 | 6.5789 | 1.25078 | 5.14474 | IP_DMC_vs_In_DMC_peak_421 | EDL933_RS24300:CDS;EDL933_RS24310:Promoter;EDL933_RS24295:Promoter;EDL933_RS24290:Promoter | EDL933_RS24300:NZ_CP008957.1:4680762-4681341:-:291 | EDL933_RS24300(espA) |
| NZ_CP008957.1 | 1441893 | 1442120 | 228 | 1442039 | 455 | 5.76066 | 1.2505 | 4.36612 | IP_DMC_vs_In_DMC_peak_150 | EDL933_RS07230:Promoter;EDL933_RS07225:CDS;EDL933_RS07245:Promoter;EDL933_RS07235:Promoter;EDL933_RS07240:Promoter | EDL933_RS07225:NZ_CP008957.1:1441896-1442265:+:110 | EDL933_RS07225(acpS) |
| NZ_CP008957.1 | 314984 | 315245 | 262 | 315117 | 455 | 5.76066 | 1.2505 | 4.36612 | IP_DMC_vs_In_DMC_peak_44 | EDL933_RS01535:CDS | EDL933_RS01535:NZ_CP008957.1:314701-315268:+:413 | EDL933_RS01535(EDL933_RS01535) |
| NZ_CP008957.1 | 3470602 | 3470926 | 325 | 3470694 | 523 | 6.45881 | 1.2501 | 5.03006 | IP_DMC_vs_In_DMC_peak_272 | EDL933_RS18130:Promoter;EDL933_RS18110:Promoter;EDL933_RS18120:CDS;EDL933_RS18115:Promoter;EDL933_RS18125:Promoter | EDL933_RS18120:NZ_CP008957.1:3469977-3470715:-:-48 | EDL933_RS18120(trmJ) |
| **In LB** | | | | | | | | | | | | |
| NZ_CP008957.1 | 4863185 | 4863676 | 492 | 4863484 | 3799 | 1224.30872 | 4.57776 | 1220.28748 | IP_LBN_vs_In_LBN_peak_488 | EDL933_RS25265:Promoter;EDL933_RS25270:Promoter;EDL933_RS25250:Promoter;EDL933_RS25245:Promoter;EDL933_RS25260:Promoter | EDL933_RS25240:NZ_CP008957.1:4863405-4863482:+:25 | EDL933_RS25240(EDL933_RS25240) |
| NZ_CP008957.1 | 2708300 | 2709096 | 797 | 2708686 | 2720 | 869.63098 | 4.53833 | 866.18079 | IP_LBN_vs_In_LBN_peak_231 | EDL933_RS33550:Promoter;EDL933_RS14260:exon;EDL933_RS33555:Promoter;EDL933_RS14250:Promoter;EDL933_RS14255:Promoter | EDL933_RS14260:NZ_CP008957.1:2708647-2708721:-:23 | EDL933_RS14260(EDL933_RS14260) |
| NZ_CP008957.1 | 5071777 | 5072692 | 916 | 5072268 | 3485 | 1103.86707 | 4.50268 | 1099.93469 | IP_LBN_vs_In_LBN_peak_515 | EDL933_RS26290:Promoter;EDL933_RS26260:Promoter;EDL933_RS26285:Promoter;EDL933_RS26295:Promoter;EDL933_RS26280:Promoter | EDL933_RS26280:NZ_CP008957.1:5072315-5072390:+:-81 | EDL933_RS26280(EDL933_RS26280) |
| NZ_CP008957.1 | 3629235 | 3631005 | 1771 | 3630118 | 2487 | 720.836 | 4.14971 | 717.94128 | IP_LBN_vs_In_LBN_peak_336 | EDL933_RS18950:Promoter;EDL933_RS18955:Promoter;EDL933_RS18930:Promoter;EDL933_RS18960:Promoter;EDL933_RS18925:Promoter;EDL933_RS18965:exon;EDL933_RS18935:Promoter;EDL933_RS18945:Promoter | EDL933_RS18960:NZ_CP008957.1:3630021-3630098:-:-21 | EDL933_RS18960(EDL933_RS18960) |
| NZ_CP008957.1 | 4151035 | 4152957 | 1923 | 4152555 | 3067 | 879.63757 | 4.11498 | 876.13531 | IP_LBN_vs_In_LBN_peak_391 | EDL933_RS21620:CDS;EDL933_RS21615:Promoter;EDL933_RS21610:Promoter | EDL933_RS21610:NZ_CP008957.1:4150112-4151738:-:-257 | EDL933_RS21610(EDL933_RS21610) |
| NZ_CP008957.1 | 4919154 | 4920807 | 1654 | 4919457 | 3239 | 921.74487 | 4.08793 | 918.1015 | IP_LBN_vs_In_LBN_peak_493 | EDL933_RS25540:Promoter;EDL933_RS25545:Promoter;EDL933_RS25535:Promoter;EDL933_RS25530:exon | EDL933_RS25530:NZ_CP008957.1:4919279-4920821:+:701 | EDL933_RS25530(EDL933_RS25530) |
| NZ_CP008957.1 | 228998 | 232547 | 3550 | 229224 | 2727 | 750.37787 | 3.96966 | 747.40753 | IP_LBN_vs_In_LBN_peak_16 | EDL933_RS01055:exon | EDL933_RS01060:NZ_CP008957.1:232087-232203:+:-1315 | EDL933_RS01060(rrf) |
| NZ_CP008957.1 | 5104044 | 5106116 | 2073 | 5104744 | 3175 | 859.17212 | 3.91641 | 855.79437 | IP_LBN_vs_In_LBN_peak_518 | EDL933_RS26435:Promoter;EDL933_RS26440:Promoter;EDL933_RS26430:exon;EDL933_RS26425:Promoter | EDL933_RS26430:NZ_CP008957.1:5104578-5106120:+:501 | EDL933_RS26430(EDL933_RS26430) |
| NZ_CP008957.1 | 4148018 | 4148802 | 785 | 4148379 | 2786 | 751.01318 | 3.90211 | 748.04102 | IP_LBN_vs_In_LBN_peak_390 | EDL933_RS21600:exon;EDL933_RS21605:Promoter;EDL933_RS21595:Promoter;EDL933_RS21590:Promoter | EDL933_RS21600:NZ_CP008957.1:4148337-4148414:-:4 | EDL933_RS21600(EDL933_RS21600) |
| NZ_CP008957.1 | 226563 | 228643 | 2081 | 227564 | 3263 | 871.04358 | 3.87268 | 867.58569 | IP_LBN_vs_In_LBN_peak_15 | EDL933_RS01055:Promoter;EDL933_RS01050:Promoter;EDL933_RS01045:Promoter;EDL933_RS01030:Promoter;EDL933_RS01040:exon | EDL933_RS01040:NZ_CP008957.1:227102-228644:+:500 | EDL933_RS01040(EDL933_RS01040) |
| NZ_CP008957.1 | 3536631 | 3538298 | 1668 | 3537696 | 3318 | 872.46698 | 3.82514 | 868.99902 | IP_LBN_vs_In_LBN_peak_319 | EDL933_RS18435:exon;EDL933_RS18430:Promoter;EDL933_RS18425:Promoter | EDL933_RS18435:NZ_CP008957.1:3536623-3538165:-:701 | EDL933_RS18435(EDL933_RS18435) |
| NZ_CP008957.1 | 776183 | 777282 | 1100 | 776919 | 2291 | 602.97729 | 3.8228 | 600.24585 | IP_LBN_vs_In_LBN_peak_101 | EDL933_RS03595:Promoter;EDL933_RS03615:exon;EDL933_RS03600:Promoter;EDL933_RS03610:Promoter;EDL933_RS03605:Promoter;EDL933_RS03590:Promoter | EDL933_RS03605:NZ_CP008957.1:776694-776769:-:37 | EDL933_RS03605(EDL933_RS03605) |
| NZ_CP008957.1 | 4822755 | 4824409 | 1655 | 4823382 | 3174 | 814.64679 | 3.75026 | 811.45294 | IP_LBN_vs_In_LBN_peak_484 | EDL933_RS25045:exon;EDL933_RS25040:Promoter;EDL933_RS25055:Promoter;EDL933_RS25050:Promoter;EDL933_RS25035:Promoter | EDL933_RS25045:NZ_CP008957.1:4822875-4824417:+:706 | EDL933_RS25045(EDL933_RS25045) |
| NZ_CP008957.1 | 4249879 | 4251889 | 2011 | 4250936 | 3141 | 787.51782 | 3.68074 | 784.4364 | IP_LBN_vs_In_LBN_peak_406 | EDL933_RS22120:exon;EDL933_RS22110:Promoter;EDL933_RS22115:Promoter;EDL933_RS22125:Promoter;EDL933_RS22105:Promoter | EDL933_RS22120:NZ_CP008957.1:4249860-4251402:-:518 | EDL933_RS22120(EDL933_RS22120) |
| NZ_CP008957.1 | 5063425 | 5065089 | 1665 | 5064014 | 3184 | 781.20203 | 3.61887 | 778.14136 | IP_LBN_vs_In_LBN_peak_512 | EDL933_RS26235:Promoter;EDL933_RS26230:exon;EDL933_RS26240:Promoter | EDL933_RS26230:NZ_CP008957.1:5063552-5065094:+:704 | EDL933_RS26230(EDL933_RS26230) |
| NZ_CP008957.1 | 4554873 | 4556206 | 1334 | 4555189 | 3084 | 743.2663 | 3.56879 | 740.31439 | IP_LBN_vs_In_LBN_peak_442 | EDL933_RS23705:Promoter;EDL933_RS23695:Promoter | EDL933_RS23710:NZ_CP008957.1:4555646-4555799:-:260 | EDL933_RS23710(hokA) |
| NZ_CP008957.1 | 3336110 | 3336955 | 846 | 3336440 | 2135 | 514.34576 | 3.56261 | 511.74347 | IP_LBN_vs_In_LBN_peak_301 | EDL933_RS17490:Promoter;EDL933_RS17495:Promoter;EDL933_RS17515:Promoter;EDL933_RS17485:Promoter;EDL933_RS17475:Promoter;EDL933_RS17500:Promoter | EDL933_RS17490:NZ_CP008957.1:3336592-3336668:+:-60 | EDL933_RS17490(EDL933_RS17490) |
| NZ_CP008957.1 | 3752521 | 3753139 | 619 | 3752808 | 2415 | 579.133 | 3.55195 | 576.43347 | IP_LBN_vs_In_LBN_peak_352 | EDL933_RS19570:Promoter;EDL933_RS19555:Promoter;EDL933_RS19550:Promoter;EDL933_RS19575:Promoter;EDL933_RS19560:Promoter | EDL933_RS19560:NZ_CP008957.1:3751675-3752593:-:-236 | EDL933_RS19560(gcvA) |
| NZ_CP008957.1 | 4824691 | 4828317 | 3627 | 4824939 | 2723 | 646.91467 | 3.5283 | 644.13177 | IP_LBN_vs_In_LBN_peak_485 | EDL933_RS25055:exon | EDL933_RS25060:NZ_CP008957.1:4827770-4827886:+:-1266 | EDL933_RS25060(rrf) |
| NZ_CP008957.1 | 4921197 | 4924395 | 3199 | 4921475 | 2701 | 634.30389 | 3.49735 | 631.53418 | IP_LBN_vs_In_LBN_peak_494 | EDL933_RS25545:exon | EDL933_RS25550:NZ_CP008957.1:4924266-4924382:+:-1470 | EDL933_RS25550(rrf) |
| NZ_CP008957.1 | 1297597 | 1298529 | 933 | 1298230 | 2687 | 612.86945 | 3.4218 | 610.12689 | IP_LBN_vs_In_LBN_peak_156 | EDL933_RS06450:Promoter;EDL933_RS06445:Promoter;EDL933_RS06425:Promoter;EDL933_RS06440:exon | EDL933_RS06440:NZ_CP008957.1:1298217-1298305:-:242 | EDL933_RS06440(EDL933_RS06440) |
| NZ_CP008957.1 | 3331243 | 3332149 | 907 | 3331845 | 1982 | 432.2236 | 3.30743 | 429.83286 | IP_LBN_vs_In_LBN_peak_300 | EDL933_RS17460:Promoter;EDL933_RS17455:Promoter;EDL933_RS17450:exon;EDL933_RS17445:Promoter | EDL933_RS17445:NZ_CP008957.1:3329431-3331621:-:-74 | EDL933_RS17445(EDL933_RS17445) |
| NZ_CP008957.1 | 3562260 | 3563055 | 796 | 3562562 | 2051 | 427.73877 | 3.20522 | 425.36002 | IP_LBN_vs_In_LBN_peak_327 | EDL933_RS18575:Promoter;EDL933_RS18560:Promoter;EDL933_RS18565:Promoter;EDL933_RS29255:exon | EDL933_RS29255:NZ_CP008957.1:3562490-3562853:+:167 | EDL933_RS29255(ssrA) |
| NZ_CP008957.1 | 5065364 | 5068750 | 3387 | 5066718 | 2770 | 567.50421 | 3.16988 | 564.81891 | IP_LBN_vs_In_LBN_peak_513 | EDL933_RS26250:Promoter;EDL933_RS26245:Promoter;EDL933_RS26240:exon | EDL933_RS26245:NZ_CP008957.1:5068447-5068563:+:-1390 | EDL933_RS26245(rrf) |
| NZ_CP008957.1 | 5106390 | 5110110 | 3721 | 5107794 | 2655 | 528.89996 | 3.10944 | 526.26141 | IP_LBN_vs_In_LBN_peak_519 | EDL933_RS26445:Promoter;EDL933_RS26440:exon | EDL933_RS26445:NZ_CP008957.1:5109473-5109589:+:-1223 | EDL933_RS26445(rrf) |
| NZ_CP008957.1 | 5214942 | 5215963 | 1022 | 5215274 | 2480 | 488.3877 | 3.08471 | 485.85269 | IP_LBN_vs_In_LBN_peak_533 | EDL933_RS26905:Promoter;EDL933_RS26910:Promoter;EDL933_RS26915:Promoter | EDL933_RS26915:NZ_CP008957.1:5215318-5217589:+:134 | EDL933_RS26915(EDL933_RS26915) |
| NZ_CP008957.1 | 4422344 | 4422848 | 505 | 4422581 | 2402 | 471.91895 | 3.07949 | 469.42944 | IP_LBN_vs_In_LBN_peak_423 | EDL933_RS23085:Promoter;EDL933_RS23075:Promoter;EDL933_RS23090:Promoter;EDL933_RS23080:Promoter;EDL933_RS23070:CDS | EDL933_RS23070:NZ_CP008957.1:4422443-4423265:+:152 | EDL933_RS23070(EDL933_RS23070) |
| NZ_CP008957.1 | 3533055 | 3536330 | 3276 | 3533938 | 2656 | 476.13663 | 2.90309 | 473.63541 | IP_LBN_vs_In_LBN_peak_318 | EDL933_RS18415:Promoter;EDL933_RS18420:Promoter;EDL933_RS18425:exon | EDL933_RS18420:NZ_CP008957.1:3533156-3533272:-:-1420 | EDL933_RS18420(rrf) |
| NZ_CP008957.1 | 240271 | 244121 | 3851 | 240572 | 1806 | 294.30069 | 2.73482 | 292.09433 | IP_LBN_vs_In_LBN_peak_17 | EDL933_RS01105:Promoter;EDL933_RS01120:Promoter | EDL933_RS01130:NZ_CP008957.1:242164-242614:-:418 | EDL933_RS01130(EDL933_RS01130) |
| NZ_CP008957.1 | 1641605 | 1641983 | 379 | 1641806 | 1634 | 265.21844 | 2.727 | 263.04684 | IP_LBN_vs_In_LBN_peak_198 | EDL933_RS08375:Promoter;EDL933_RS08390:Promoter;EDL933_RS30185:Promoter | EDL933_RS30185:NZ_CP008957.1:1642006-1642369:+:-212 | EDL933_RS30185(EDL933_RS30185) |
| NZ_CP008957.1 | 5395692 | 5396279 | 588 | 5395935 | 2037 | 322.08493 | 2.68867 | 319.85507 | IP_LBN_vs_In_LBN_peak_561 | EDL933_RS27810:exon;EDL933_RS27805:Promoter;EDL933_RS27800:Promoter;EDL933_RS27815:Promoter | EDL933_RS27815:NZ_CP008957.1:5396057-5398007:+:-72 | EDL933_RS27815(EDL933_RS27815) |
| NZ_CP008957.1 | 1167654 | 1171965 | 4312 | 1171371 | 1667 | 257.47891 | 2.65015 | 255.31715 | IP_LBN_vs_In_LBN_peak_139 | EDL933_RS33145:CDS;EDL933_RS05730:Promoter | EDL933_RS05730:NZ_CP008957.1:1168246-1170529:-:720 | EDL933_RS05730(pflB) |
| NZ_CP008957.1 | 4137809 | 4139106 | 1298 | 4138629 | 1884 | 281.43652 | 2.60243 | 279.24503 | IP_LBN_vs_In_LBN_peak_388 | EDL933_RS21555:Promoter;EDL933_RS32785:Promoter;EDL933_RS21560:CDS | EDL933_RS32785:NZ_CP008957.1:4137976-4138057:-:-400 | EDL933_RS32785(yrbN) |
| NZ_CP008957.1 | 1315450 | 1317580 | 2131 | 1316511 | 1958 | 284.31827 | 2.56253 | 282.12357 | IP_LBN_vs_In_LBN_peak_158 | EDL933_RS06540:Promoter;EDL933_RS29850:Promoter;EDL933_RS29845:Promoter;EDL933_RS06535:Promoter;EDL933_RS06510:Promoter;EDL933_RS06515:Promoter;EDL933_RS06525:Promoter;EDL933_RS06520:Promoter | EDL933_RS06525:NZ_CP008957.1:1316041-1316347:-:-167 | EDL933_RS06525(EDL933_RS06525) |
| NZ_CP008957.1 | 3880099 | 3881186 | 1088 | 3880807 | 1788 | 258.82343 | 2.55703 | 256.6597 | IP_LBN_vs_In_LBN_peak_361 | EDL933_RS20190:Promoter;EDL933_RS29275:exon;EDL933_RS20175:Promoter;EDL933_RS20180:Promoter | EDL933_RS29275:NZ_CP008957.1:3880737-3880921:+:-95 | EDL933_RS29275(ssrS) |
| NZ_CP008957.1 | 3522946 | 3523814 | 869 | 3523541 | 1667 | 233.67674 | 2.51213 | 231.54758 | IP_LBN_vs_In_LBN_peak_316 | EDL933_RS18360:Promoter;EDL933_RS18375:Promoter;EDL933_RS18370:Promoter | EDL933_RS18370:NZ_CP008957.1:3523153-3523537:-:157 | EDL933_RS18370(grcA) |
| NZ_CP008957.1 | 2440471 | 2440961 | 491 | 2440706 | 1502 | 209.95052 | 2.50684 | 207.85628 | IP_LBN_vs_In_LBN_peak_217 | EDL933_RS12800:Promoter;EDL933_RS12815:Promoter;EDL933_RS12820:Promoter;EDL933_RS12810:exon | EDL933_RS12810:NZ_CP008957.1:2440687-2440764:+:28 | EDL933_RS12810(EDL933_RS12810) |
| NZ_CP008957.1 | 2807392 | 2807911 | 520 | 2807623 | 1474 | 198.85912 | 2.46014 | 196.78154 | IP_LBN_vs_In_LBN_peak_238 | EDL933_RS14905:Promoter;EDL933_RS14920:Promoter;EDL933_RS14915:exon | EDL933_RS14915:NZ_CP008957.1:2807579-2807655:+:72 | EDL933_RS14915(EDL933_RS14915) |
| NZ_CP008958.1 | 48108 | 48717 | 610 | 48432 | 3286 | 439.67514 | 2.45808 | 437.26724 | IP_LBN_vs_In_LBN_peak_601 | intergenic | EDL933_RS28810:NZ_CP008958.1:47524-48265:+:888 | EDL933_RS28810(EDL933_RS28810) |
| NZ_CP008958.1 | 139 | 3328 | 3190 | 2920 | 3368 | 449.12695 | 2.45396 | 446.69662 | IP_LBN_vs_In_LBN_peak_588 | EDL933_RS28585:Promoter;EDL933_RS28595:Promoter;EDL933_RS28590:Promoter | EDL933_RS28570:NZ_CP008958.1:1657-1867:+:76 | EDL933_RS28570(hha) |
| NZ_CP008957.1 | 2823591 | 2824041 | 451 | 2823817 | 1432 | 182.65555 | 2.39009 | 180.60251 | IP_LBN_vs_In_LBN_peak_242 | EDL933_RS29235:Promoter;EDL933_RS14960:exon;EDL933_RS14945:Promoter | EDL933_RS14960:NZ_CP008957.1:2823783-2823859:+:32 | EDL933_RS14960(EDL933_RS14960) |
| NZ_CP008957.1 | 3734059 | 3734669 | 611 | 3734359 | 1531 | 193.94978 | 2.38285 | 191.87953 | IP_LBN_vs_In_LBN_peak_349 | EDL933_RS19465:Promoter;EDL933_RS19495:Promoter;EDL933_RS19475:Promoter;EDL933_RS19470:Promoter;EDL933_RS19490:Promoter | EDL933_RS19475:NZ_CP008957.1:3733809-3734139:-:-224 | EDL933_RS19475(EDL933_RS19475) |
| NZ_CP008957.1 | 3824238 | 3825245 | 1008 | 3824962 | 1654 | 208.88525 | 2.38021 | 206.79277 | IP_LBN_vs_In_LBN_peak_356 | EDL933_RS19955:exon;EDL933_RS19940:Promoter;EDL933_RS19965:Promoter;EDL933_RS19945:Promoter | EDL933_RS19945:NZ_CP008957.1:3823100-3824706:-:-35 | EDL933_RS19945(EDL933_RS19945) |
| NZ_CP008957.1 | 2865069 | 2875995 | 10927 | 2872446 | 1420 | 178.12331 | 2.37007 | 176.07744 | IP_LBN_vs_In_LBN_peak_247 | EDL933_RS15215:Promoter;EDL933_RS15220:CDS | EDL933_RS15210:NZ_CP008957.1:2869189-2870404:-:-127 | EDL933_RS15210(EDL933_RS15210) |
| NZ_CP008957.1 | 4275652 | 4276232 | 581 | 4275980 | 1829 | 227.21722 | 2.36207 | 225.09668 | IP_LBN_vs_In_LBN_peak_409 | EDL933_RS22345:Promoter;EDL933_RS22330:Promoter;EDL933_RS22325:Promoter;EDL933_RS22335:Promoter;EDL933_RS22320:Promoter;EDL933_RS22315:Promoter | EDL933_RS22335:NZ_CP008957.1:4275597-4275909:-:-32 | EDL933_RS22335(rpsJ) |
| NZ_CP008957.1 | 5208479 | 5209126 | 648 | 5208778 | 38148 | 4592.06641 | 2.33788 | 4587.44873 | IP_LBN_vs_In_LBN_peak_532 | EDL933_RS26875:Promoter;EDL933_RS26880:CDS | EDL933_RS26880:NZ_CP008957.1:5208438-5209146:+:364 | EDL933_RS26880(EDL933_RS26880) |
| NZ_CP008957.1 | 4694594 | 4709530 | 14937 | 4702563 | 2084 | 251.72745 | 2.33122 | 249.57399 | IP_LBN_vs_In_LBN_peak_462 | EDL933_RS24405:Promoter;EDL933_RS24410:Promoter;EDL933_RS24415:CDS | EDL933_RS24410:NZ_CP008957.1:4701858-4702230:-:168 | EDL933_RS24410(grlR) |
| NZ_CP008957.1 | 4071765 | 4072325 | 561 | 4072066 | 1620 | 194.49847 | 2.32147 | 192.4274 | IP_LBN_vs_In_LBN_peak_380 | EDL933_RS21200:Promoter;EDL933_RS21185:Promoter;EDL933_RS21195:Promoter;EDL933_RS21190:Promoter | EDL933_RS21190:NZ_CP008957.1:4070587-4072000:-:-44 | EDL933_RS21190(uxaC) |
| NZ_CP008957.1 | 2821412 | 2822183 | 772 | 2821959 | 1387 | 165.88733 | 2.31503 | 163.86438 | IP_LBN_vs_In_LBN_peak_241 | EDL933_RS14960:Promoter | EDL933_RS14945:NZ_CP008957.1:2821959-2822035:-:238 | EDL933_RS14945(EDL933_RS14945) |
| NZ_CP008958.1 | 78311 | 88076 | 9766 | 84862 | 3318 | 388.28647 | 2.29987 | 385.98444 | IP_LBN_vs_In_LBN_peak_606 | EDL933_RS29015:CDS | EDL933_RS29015:NZ_CP008958.1:78540-88050:+:4653 | EDL933_RS29015(toxB) |
| NZ_CP008957.1 | 301602 | 302094 | 493 | 301829 | 1453 | 169.93971 | 2.29104 | 167.90912 | IP_LBN_vs_In_LBN_peak_24 | EDL933_RS01450:Promoter;EDL933_RS01445:Promoter;EDL933_RS01425:Promoter;EDL933_RS01420:Promoter;EDL933_RS01430:Promoter;EDL933_RS01440:Promoter | EDL933_RS01430:NZ_CP008957.1:301422-301812:-:-35 | EDL933_RS01430(EDL933_RS01430) |
| NZ_CP008957.1 | 3436948 | 3437487 | 540 | 3437217 | 1370 | 159.71619 | 2.28668 | 157.70587 | IP_LBN_vs_In_LBN_peak_307 | EDL933_RS17985:Promoter;EDL933_RS17980:Promoter;EDL933_RS17975:Promoter | EDL933_RS17985:NZ_CP008957.1:3437264-3438635:+:-47 | EDL933_RS17985(xseA) |
| NZ_CP008957.1 | 3796244 | 3815690 | 19447 | 3813261 | 1551 | 178.30199 | 2.27331 | 176.25592 | IP_LBN_vs_In_LBN_peak_354 | EDL933_RS19890:CDS;EDL933_RS19870:Promoter;EDL933_RS19885:Promoter;EDL933_RS19880:Promoter | EDL933_RS34190:NZ_CP008957.1:3805582-3806014:-:47 | EDL933_RS34190(EDL933_RS34190) |
| NZ_CP008957.1 | 4037630 | 4038449 | 820 | 4037968 | 1607 | 181.75529 | 2.2565 | 179.70369 | IP_LBN_vs_In_LBN_peak_377 | EDL933_RS21055:Promoter;EDL933_RS21050:CDS;EDL933_RS21045:Promoter | EDL933_RS21050:NZ_CP008957.1:4037938-4038154:+:101 | EDL933_RS21050(rpsU) |
| NZ_CP008957.1 | 542288 | 542776 | 489 | 542526 | 1377 | 154.09727 | 2.24332 | 152.09998 | IP_LBN_vs_In_LBN_peak_65 | EDL933_RS02620:Promoter;EDL933_RS29160:exon;EDL933_RS02605:Promoter;EDL933_RS02615:Promoter | EDL933_RS29160:NZ_CP008957.1:542520-542617:+:11 | EDL933_RS29160(ffs) |
| NZ_CP008958.1 | 3878 | 4399 | 522 | 4095 | 2929 | 321.31281 | 2.23074 | 319.08359 | IP_LBN_vs_In_LBN_peak_589 | EDL933_RS28600:Promoter;EDL933_RS28595:Promoter;EDL933_RS28605:Promoter | EDL933_RS28595:NZ_CP008958.1:4809-5094:+:-671 | EDL933_RS28595(EDL933_RS28595) |
| NZ_CP008957.1 | 3092865 | 3094006 | 1142 | 3093155 | 1330 | 145.49426 | 2.21952 | 143.51932 | IP_LBN_vs_In_LBN_peak_273 | EDL933_RS16380:Promoter;EDL933_RS16375:Promoter | EDL933_RS16375:NZ_CP008957.1:3093227-3093794:+:208 | EDL933_RS16375(mepS) |
| NZ_CP008957.1 | 501311 | 503095 | 1785 | 502559 | 1350 | 146.32965 | 2.21046 | 144.35223 | IP_LBN_vs_In_LBN_peak_61 | EDL933_RS02430:CDS | EDL933_RS02430:NZ_CP008957.1:501536-503108:-:905 | EDL933_RS02430(espY3) |
| NZ_CP008957.1 | 4846538 | 4847648 | 1111 | 4847285 | 2136 | 229.80244 | 2.20846 | 227.67851 | IP_LBN_vs_In_LBN_peak_487 | EDL933_RS25165:Promoter;EDL933_RS25150:Promoter;EDL933_RS25175:Promoter;EDL933_RS25160:CDS | EDL933_RS25160:NZ_CP008957.1:4847261-4847363:+:-168 | EDL933_RS25160(rhoL) |
| NZ_CP008958.1 | 49549 | 49982 | 434 | 49768 | 2823 | 301.02121 | 2.20199 | 298.80804 | IP_LBN_vs_In_LBN_peak_602 | EDL933_RS28815:Promoter | EDL933_RS28815:NZ_CP008958.1:48549-49527:-:-238 | EDL933_RS28815(EDL933_RS28815) |
| NZ_CP008957.1 | 3109244 | 3109712 | 469 | 3109497 | 1316 | 140.72533 | 2.19661 | 138.76541 | IP_LBN_vs_In_LBN_peak_275 | EDL933_RS16445:exon | EDL933_RS16445:NZ_CP008957.1:3109459-3109536:+:18 | EDL933_RS16445(EDL933_RS16445) |
| NZ_CP008957.1 | 972981 | 973414 | 434 | 973213 | 1325 | 140.36996 | 2.18753 | 138.41132 | IP_LBN_vs_In_LBN_peak_120 | EDL933_RS04670:Promoter;EDL933_RS04660:Promoter;EDL933_RS33100:Promoter;EDL933_RS04650:Promoter | EDL933_RS04660:NZ_CP008957.1:972484-972988:-:-209 | EDL933_RS04660(dps) |
| NZ_CP008957.1 | 4245921 | 4249485 | 3565 | 4246027 | 9198 | 957.15497 | 2.18357 | 953.43634 | IP_LBN_vs_In_LBN_peak_405 | intergenic | EDL933_RS22100:NZ_CP008957.1:4246301-4246417:-:-1285 | EDL933_RS22100(rrf) |
| NZ_CP008957.1 | 5294717 | 5295522 | 806 | 5294899 | 1582 | 161.45615 | 2.1541 | 159.44215 | IP_LBN_vs_In_LBN_peak_548 | EDL933_RS27290:Promoter;EDL933_RS27300:Promoter;EDL933_RS27310:Promoter;EDL933_RS33890:Promoter;EDL933_RS27320:Promoter;EDL933_RS27285:Promoter;EDL933_RS27305:Promoter | EDL933_RS27310:NZ_CP008957.1:5295173-5295249:+:-54 | EDL933_RS27310(EDL933_RS27310) |
| NZ_CP008957.1 | 864567 | 865619 | 1053 | 865326 | 1290 | 131.92146 | 2.15325 | 129.99352 | IP_LBN_vs_In_LBN_peak_108 | EDL933_RS04060:Promoter;EDL933_RS04050:Promoter;EDL933_RS04055:Promoter | EDL933_RS04040:NZ_CP008957.1:865071-865147:+:21 | EDL933_RS04040(EDL933_RS04040) |
| NZ_CP008957.1 | 1434224 | 1436793 | 2570 | 1435762 | 1326 | 132.24092 | 2.13031 | 130.31161 | IP_LBN_vs_In_LBN_peak_185 | EDL933_RS07205:Promoter;EDL933_RS32150:Promoter;EDL933_RS07215:Promoter | EDL933_RS32150:NZ_CP008957.1:1434346-1434970:-:-538 | EDL933_RS32150(EDL933_RS32150) |
| NZ_CP008958.1 | 15708 | 18049 | 2342 | 15992 | 2891 | 283.57791 | 2.12322 | 281.38419 | IP_LBN_vs_In_LBN_peak_595 | EDL933_RS28665:Promoter;EDL933_RS32940:Promoter | EDL933_RS28665:NZ_CP008958.1:17322-18144:+:-444 | EDL933_RS28665(EDL933_RS28665) |
| NZ_CP008957.1 | 18366 | 25976 | 7611 | 25540 | 1493 | 146.46835 | 2.11696 | 144.49051 | IP_LBN_vs_In_LBN_peak_2 | EDL933_RS00120:Promoter;EDL933_RS00140:Promoter;EDL933_RS00125:Promoter;EDL933_RS00135:Promoter;EDL933_RS00130:Promoter | EDL933_RS00110:NZ_CP008957.1:21761-22445:-:274 | EDL933_RS00110(EDL933_RS00110) |
| NZ_CP008957.1 | 3164680 | 3167857 | 3178 | 3167453 | 1262 | 122.69425 | 2.10654 | 120.80662 | IP_LBN_vs_In_LBN_peak_280 | intergenic | EDL933_RS16680:NZ_CP008957.1:3166331-3167402:+:-63 | EDL933_RS16680(EDL933_RS16680) |
| NZ_CP008957.1 | 5073784 | 5077767 | 3984 | 5076253 | 1703 | 164.59445 | 2.10518 | 162.57385 | IP_LBN_vs_In_LBN_peak_516 | EDL933_RS26315:Promoter;EDL933_RS26325:Promoter;EDL933_RS26320:Promoter | EDL933_RS26310:NZ_CP008957.1:5075521-5076226:+:254 | EDL933_RS26310(rplA) |
| NZ_CP008957.1 | 3579386 | 3586525 | 7140 | 3584340 | 1421 | 136.71767 | 2.09893 | 134.77155 | IP_LBN_vs_In_LBN_peak_330 | EDL933_RS18720:CDS;EDL933_RS18715:Promoter | EDL933_RS18715:NZ_CP008957.1:3581889-3583521:-:566 | EDL933_RS18715(EDL933_RS18715) |
| NZ_CP008957.1 | 4162694 | 4163776 | 1083 | 4163421 | 1503 | 144.00067 | 2.09595 | 142.03021 | IP_LBN_vs_In_LBN_peak_396 | EDL933_RS21685:Promoter;EDL933_RS21680:Promoter;EDL933_RS21660:Promoter;EDL933_RS21670:Promoter;EDL933_RS21675:CDS;EDL933_RS21665:Promoter | EDL933_RS21670:NZ_CP008957.1:4162840-4163098:-:-136 | EDL933_RS21670(rpmA) |
| NZ_CP008957.1 | 738218 | 738814 | 597 | 738484 | 1252 | 119.4637 | 2.08987 | 117.59052 | IP_LBN_vs_In_LBN_peak_96 | EDL933_RS03405:Promoter;EDL933_RS03385:Promoter;EDL933_RS03395:CDS;EDL933_RS03410:Promoter | EDL933_RS03395:NZ_CP008957.1:738424-738634:+:91 | EDL933_RS03395(cspE) |
| NZ_CP008957.1 | 517588 | 518234 | 647 | 517868 | 1264 | 120.43637 | 2.08876 | 118.55849 | IP_LBN_vs_In_LBN_peak_62 | EDL933_RS02500:Promoter;EDL933_RS02495:Promoter | EDL933_RS02500:NZ_CP008957.1:516715-517663:-:-247 | EDL933_RS02500(cyoA) |
| NZ_CP008957.1 | 1420440 | 1428424 | 7985 | 1425894 | 1348 | 126.95228 | 2.07954 | 125.04554 | IP_LBN_vs_In_LBN_peak_184 | EDL933_RS07180:Promoter;EDL933_RS07170:CDS | EDL933_RS07165:NZ_CP008957.1:1423709-1425320:+:722 | EDL933_RS07165(EDL933_RS07165) |
| NZ_CP008957.1 | 321836 | 323699 | 1864 | 322314 | 1378 | 129.67181 | 2.07914 | 127.75324 | IP_LBN_vs_In_LBN_peak_30 | EDL933_RS01585:CDS | EDL933_RS01585:NZ_CP008957.1:322024-322981:-:214 | EDL933_RS01585(EDL933_RS01585) |
| NZ_CP008957.1 | 1260117 | 1262014 | 1898 | 1261881 | 1245 | 117.22286 | 2.07819 | 115.36115 | IP_LBN_vs_In_LBN_peak_152 | EDL933_RS32105:Promoter;EDL933_RS06200:Promoter;EDL933_RS06185:Promoter;EDL933_RS06180:Promoter;EDL933_RS06195:Promoter | EDL933_RS06180:NZ_CP008957.1:1260641-1261415:-:350 | EDL933_RS06180(EDL933_RS06180) |
| NZ_CP008957.1 | 299771 | 300285 | 515 | 300003 | 1329 | 123.37381 | 2.06699 | 121.48341 | IP_LBN_vs_In_LBN_peak_23 | EDL933_RS01415:exon | EDL933_RS01415:NZ_CP008957.1:299982-300058:+:45 | EDL933_RS01415(EDL933_RS01415) |
| NZ_CP008957.1 | 5512806 | 5513623 | 818 | 5513375 | 1498 | 138.1319 | 2.06287 | 136.18054 | IP_LBN_vs_In_LBN_peak_585 | EDL933_RS28330:Promoter;EDL933_RS28310:Promoter;EDL933_RS28320:exon;EDL933_RS28335:Promoter | EDL933_RS28315:NZ_CP008957.1:5513035-5513272:+:179 | EDL933_RS28315(EDL933_RS28315) |
| NZ_CP008957.1 | 4262243 | 4271374 | 9132 | 4270785 | 1519 | 137.9258 | 2.0501 | 135.9752 | IP_LBN_vs_In_LBN_peak_408 | EDL933_RS22280:CDS;EDL933_RS22265:Promoter;EDL933_RS22260:Promoter;EDL933_RS22275:Promoter;EDL933_RS22270:Promoter | EDL933_RS22230:NZ_CP008957.1:4265404-4266736:-:-72 | EDL933_RS22230(secY) |
| NZ_CP008957.1 | 4403622 | 4404114 | 493 | 4403913 | 1612 | 145.8233 | 2.0477 | 143.84729 | IP_LBN_vs_In_LBN_peak_420 | EDL933_RS22960:Promoter;EDL933_RS22955:Promoter;EDL933_RS22965:Promoter | EDL933_RS22960:NZ_CP008957.1:4402562-4403672:-:-195 | EDL933_RS22960(livK) |
| NZ_CP008957.1 | 4484666 | 4488699 | 4034 | 4487711 | 1659 | 149.43306 | 2.04448 | 147.44707 | IP_LBN_vs_In_LBN_peak_431 | EDL933_RS23430:Promoter;EDL933_RS23410:Promoter;EDL933_RS23405:Promoter;EDL933_RS23420:Promoter | EDL933_RS23415:NZ_CP008957.1:4486753-4487326:+:-71 | EDL933_RS23415(hdeD) |
| NZ_CP008957.1 | 4933317 | 4933747 | 431 | 4933500 | 1707 | 153.27831 | 2.04222 | 151.28285 | IP_LBN_vs_In_LBN_peak_496 | EDL933_RS25605:Promoter | EDL933_RS25600:NZ_CP008957.1:4933711-4934344:-:812 | EDL933_RS25600(EDL933_RS25600) |
| NZ_CP008957.1 | 5152620 | 5154194 | 1575 | 5153627 | 1596 | 142.7773 | 2.03839 | 140.81078 | IP_LBN_vs_In_LBN_peak_525 | EDL933_RS26645:Promoter;EDL933_RS26650:Promoter;EDL933_RS26640:CDS | EDL933_RS26640:NZ_CP008957.1:5152751-5154332:+:655 | EDL933_RS26640(EDL933_RS26640) |
| NZ_CP008957.1 | 3279754 | 3280161 | 408 | 3279973 | 1218 | 108.74007 | 2.03316 | 106.92429 | IP_LBN_vs_In_LBN_peak_289 | EDL933_RS17220:Promoter;EDL933_RS17205:Promoter;EDL933_RS17215:exon | EDL933_RS17215:NZ_CP008957.1:3279912-3279987:+:45 | EDL933_RS17215(EDL933_RS17215) |
| NZ_CP008957.1 | 1387861 | 1388459 | 599 | 1388123 | 4739 | 416.29837 | 2.03168 | 413.94522 | IP_LBN_vs_In_LBN_peak_176 | EDL933_RS07010:CDS | EDL933_RS07020:NZ_CP008957.1:1391478-1391823:-:3663 | EDL933_RS07020(EDL933_RS07020) |
| NZ_CP008957.1 | 4596245 | 4598601 | 2357 | 4597944 | 1791 | 158.31087 | 2.02988 | 156.30399 | IP_LBN_vs_In_LBN_peak_449 | EDL933_RS23880:CDS | EDL933_RS23880:NZ_CP008957.1:4597916-4598252:+:-493 | EDL933_RS23880(EDL933_RS23880) |
| NZ_CP008957.1 | 3948501 | 3953160 | 4660 | 3950053 | 1500 | 132.59921 | 2.02793 | 130.6685 | IP_LBN_vs_In_LBN_peak_369 | EDL933_RS20575:Promoter;EDL933_RS20570:CDS;EDL933_RS33775:Promoter | EDL933_RS33775:NZ_CP008957.1:3950849-3950999:+:-19 | EDL933_RS33775(EDL933_RS33775) |
| NZ_CP008957.1 | 4388342 | 4390373 | 2032 | 4389828 | 1683 | 147.80713 | 2.02399 | 145.82542 | IP_LBN_vs_In_LBN_peak_419 | intergenic | EDL933_RS22890:NZ_CP008957.1:4388718-4389630:+:639 | EDL933_RS22890(EDL933_RS22890) |
| NZ_CP008957.1 | 110897 | 112539 | 1643 | 111140 | 1508 | 132.40231 | 2.02249 | 130.4724 | IP_LBN_vs_In_LBN_peak_7 | EDL933_RS00520:Promoter;EDL933_RS00525:Promoter;EDL933_RS00515:Promoter | EDL933_RS00515:NZ_CP008957.1:111162-112080:+:555 | EDL933_RS00515(lpxC) |
| NZ_CP008957.1 | 4689077 | 4693600 | 4524 | 4690517 | 1742 | 152.18323 | 2.02012 | 150.19028 | IP_LBN_vs_In_LBN_peak_461 | EDL933_RS24330:Promoter;EDL933_RS24325:Promoter;EDL933_RS24335:Promoter | EDL933_RS24340:NZ_CP008957.1:4691111-4691618:-:280 | EDL933_RS24340(espH) |
| NZ_CP008957.1 | 126188 | 133750 | 7563 | 126664 | 1476 | 128.12875 | 2.01314 | 126.21706 | IP_LBN_vs_In_LBN_peak_9 | EDL933_RS00600:CDS;EDL933_RS00595:Promoter;EDL933_RS00605:Promoter | EDL933_RS00610:NZ_CP008957.1:130184-132077:+:-215 | EDL933_RS00610(aceF) |
| NZ_CP008957.1 | 3816839 | 3823727 | 6889 | 3819538 | 1318 | 114.26389 | 2.01062 | 112.41759 | IP_LBN_vs_In_LBN_peak_355 | EDL933_RS19920:Promoter;EDL933_RS19925:CDS | EDL933_RS19925:NZ_CP008957.1:3818909-3820055:-:-227 | EDL933_RS19925(EDL933_RS19925) |
| NZ_CP008957.1 | 4773866 | 4781196 | 7331 | 4776952 | 1897 | 162.68086 | 2.00628 | 160.66394 | IP_LBN_vs_In_LBN_peak_473 | EDL933_RS24825:CDS;EDL933_RS24820:Promoter | EDL933_RS24825:NZ_CP008957.1:4776618-4777290:+:912 | EDL933_RS24825(EDL933_RS24825) |
| NZ_CP008957.1 | 4867311 | 4868010 | 700 | 4867728 | 1704 | 146.09119 | 2.00496 | 144.11444 | IP_LBN_vs_In_LBN_peak_490 | EDL933_RS25275:Promoter | EDL933_RS25275:NZ_CP008957.1:4865617-4867273:-:-387 | EDL933_RS25275(aslA) |
| NZ_CP008957.1 | 5492517 | 5494418 | 1902 | 5493178 | 1410 | 120.94305 | 2.00309 | 119.06299 | IP_LBN_vs_In_LBN_peak_581 | EDL933_RS28210:Promoter;EDL933_RS28220:CDS | EDL933_RS28220:NZ_CP008957.1:5493017-5494376:+:450 | EDL933_RS28220(EDL933_RS28220) |
| NZ_CP008957.1 | 1033027 | 1035362 | 2336 | 1034986 | 1199 | 102.92563 | 2.00147 | 101.14589 | IP_LBN_vs_In_LBN_peak_130 | EDL933_RS04970:CDS;EDL933_RS04985:Promoter;EDL933_RS04990:Promoter | EDL933_RS04970:NZ_CP008957.1:1033491-1035265:+:703 | EDL933_RS04970(EDL933_RS04970) |
| NZ_CP008957.1 | 5408686 | 5412820 | 4135 | 5412050 | 1511 | 129.02684 | 2.00044 | 127.11109 | IP_LBN_vs_In_LBN_peak_566 | EDL933_RS27900:CDS;EDL933_RS27905:Promoter;EDL933_RS27895:Promoter;EDL933_RS27890:Promoter | EDL933_RS27895:NZ_CP008957.1:5410189-5410897:-:144 | EDL933_RS27895(EDL933_RS27895) |
| NZ_CP008958.1 | 41135 | 47540 | 6406 | 44984 | 2765 | 234.20779 | 1.99978 | 232.07809 | IP_LBN_vs_In_LBN_peak_600 | EDL933_RS34240:Promoter;EDL933_RS28790:Promoter;EDL933_RS28785:CDS | EDL933_RS28790:NZ_CP008958.1:44990-46430:+:-653 | EDL933_RS28790(ehxD) |
| NZ_CP008958.1 | 73738 | 74154 | 417 | 73927 | 16151 | 1356.97168 | 1.99969 | 1352.92285 | IP_LBN_vs_In_LBN_peak_605 | EDL933_RS28995:Promoter;EDL933_RS28990:Promoter | EDL933_RS28990:NZ_CP008958.1:73948-74107:+:-2 | EDL933_RS28990(EDL933_RS28990) |
| NZ_CP008957.1 | 5379666 | 5381762 | 2097 | 5380858 | 1513 | 127.7519 | 1.99169 | 125.84179 | IP_LBN_vs_In_LBN_peak_559 | EDL933_RS27760:Promoter;EDL933_RS27750:CDS | EDL933_RS27750:NZ_CP008957.1:5379840-5381631:+:873 | EDL933_RS27750(EDL933_RS27750) |
| NZ_CP008957.1 | 4680636 | 4684687 | 4052 | 4684159 | 1714 | 142.86903 | 1.98319 | 140.90216 | IP_LBN_vs_In_LBN_peak_459 | EDL933_RS24305:Promoter;EDL933_RS24315:CDS | EDL933_RS24310:NZ_CP008957.1:4682597-4683818:+:64 | EDL933_RS24310(escD) |
| NZ_CP008957.1 | 3089184 | 3089594 | 411 | 3089358 | 1188 | 99.6188 | 1.98312 | 97.85931 | IP_LBN_vs_In_LBN_peak_272 | EDL933_RS16350:Promoter;EDL933_RS16365:Promoter;EDL933_RS16360:Promoter | EDL933_RS16360:NZ_CP008957.1:3089492-3090959:+:-103 | EDL933_RS16360(EDL933_RS16360) |
| NZ_CP008957.1 | 2861606 | 2863761 | 2156 | 2863120 | 1179 | 96.94596 | 1.96811 | 95.20155 | IP_LBN_vs_In_LBN_peak_246 | EDL933_RS15170:Promoter;EDL933_RS15175:Promoter | EDL933_RS15175:NZ_CP008957.1:2861839-2862505:-:-178 | EDL933_RS15175(perB) |
| NZ_CP008957.1 | 4452896 | 4457229 | 4334 | 4454157 | 1585 | 129.31291 | 1.9661 | 127.39585 | IP_LBN_vs_In_LBN_peak_426 | EDL933_RS23240:CDS;EDL933_RS23235:Promoter | EDL933_RS23240:NZ_CP008957.1:4453068-4454691:-:-371 | EDL933_RS23240(EDL933_RS23240) |
| NZ_CP008957.1 | 954361 | 956968 | 2608 | 956532 | 1193 | 97.07377 | 1.96043 | 95.32871 | IP_LBN_vs_In_LBN_peak_118 | EDL933_RS04585:Promoter;EDL933_RS04580:CDS | EDL933_RS04580:NZ_CP008957.1:954984-956997:+:680 | EDL933_RS04580(espX2) |
| NZ_CP008957.1 | 4537828 | 4538325 | 498 | 4538111 | 1737 | 138.62791 | 1.9508 | 136.67503 | IP_LBN_vs_In_LBN_peak_439 | EDL933_RS23625:exon;EDL933_RS23615:Promoter | EDL933_RS23625:NZ_CP008957.1:4538062-4538139:-:63 | EDL933_RS23625(EDL933_RS23625) |
| NZ_CP008957.1 | 1176929 | 1177634 | 706 | 1177195 | 1183 | 94.77786 | 1.94883 | 93.04681 | IP_LBN_vs_In_LBN_peak_140 | EDL933_RS05765:Promoter;EDL933_RS05770:Promoter;EDL933_RS05760:Promoter | EDL933_RS05760:NZ_CP008957.1:1177238-1178003:+:43 | EDL933_RS05760(ycaL) |
| NZ_CP008957.1 | 4762157 | 4765198 | 3042 | 4763381 | 1761 | 139.60503 | 1.94605 | 137.64908 | IP_LBN_vs_In_LBN_peak_471 | EDL933_RS24780:Promoter;EDL933_RS24775:Promoter;EDL933_RS24770:CDS | EDL933_RS24770:NZ_CP008957.1:4762198-4764368:-:691 | EDL933_RS24770(EDL933_RS24770) |
| NZ_CP008957.1 | 5020191 | 5021249 | 1059 | 5020597 | 1689 | 133.17201 | 1.94167 | 131.23912 | IP_LBN_vs_In_LBN_peak_510 | EDL933_RS26035:Promoter;EDL933_RS26030:CDS | EDL933_RS26035:NZ_CP008957.1:5020688-5020925:+:31 | EDL933_RS26035(EDL933_RS26035) |
| NZ_CP008957.1 | 1178070 | 1180841 | 2772 | 1178823 | 1161 | 91.68968 | 1.93809 | 89.9788 | IP_LBN_vs_In_LBN_peak_141 | EDL933_RS05775:Promoter;EDL933_RS05765:CDS;EDL933_RS05770:Promoter | EDL933_RS05770:NZ_CP008957.1:1178969-1180643:+:486 | EDL933_RS05770(rpsA) |
| NZ_CP008957.1 | 1214196 | 1215518 | 1323 | 1214374 | 1225 | 96.61591 | 1.93791 | 94.87354 | IP_LBN_vs_In_LBN_peak_144 | EDL933_RS05895:Promoter;EDL933_RS05910:Promoter;EDL933_RS05900:Promoter;EDL933_RS05915:Promoter;EDL933_RS05905:Promoter;EDL933_RS05920:Promoter | EDL933_RS05910:NZ_CP008957.1:1214671-1215211:+:185 | EDL933_RS05910(EDL933_RS05910) |
| NZ_CP008957.1 | 4864011 | 4864289 | 279 | 4864095 | 1610 | 125.84382 | 1.93488 | 123.94237 | IP_LBN_vs_In_LBN_peak_489 | EDL933_RS25270:Promoter | EDL933_RS25270:NZ_CP008957.1:4864214-4865450:+:-64 | EDL933_RS25270(EDL933_RS25270) |
| NZ_CP008957.1 | 4631236 | 4637142 | 5907 | 4633444 | 1760 | 137.28989 | 1.93438 | 135.34161 | IP_LBN_vs_In_LBN_peak_455 | EDL933_RS24030:CDS | EDL933_RS24030:NZ_CP008957.1:4632668-4633811:-:-377 | EDL933_RS24030(EDL933_RS24030) |
| NZ_CP008957.1 | 1817432 | 1817977 | 546 | 1817663 | 1158 | 90.82532 | 1.93308 | 89.1203 | IP_LBN_vs_In_LBN_peak_203 | EDL933_RS29210:Promoter;EDL933_RS09445:Promoter;EDL933_RS09425:Promoter;EDL933_RS29205:Promoter;EDL933_RS29215:exon | EDL933_RS29215:NZ_CP008957.1:1817593-1817724:-:20 | EDL933_RS29215(EDL933_RS29215) |
| NZ_CP008957.1 | 1048685 | 1050166 | 1482 | 1049932 | 1155 | 89.96432 | 1.92808 | 88.26527 | IP_LBN_vs_In_LBN_peak_131 | EDL933_RS05045:Promoter;EDL933_RS05055:Promoter;EDL933_RS05050:Promoter | EDL933_RS05045:NZ_CP008957.1:1048973-1049930:-:505 | EDL933_RS05045(EDL933_RS05045) |
| NZ_CP008957.1 | 3563649 | 3566547 | 2899 | 3565784 | 1184 | 91.43688 | 1.92236 | 89.72771 | IP_LBN_vs_In_LBN_peak_328 | EDL933_RS18610:Promoter;EDL933_RS18580:Promoter;EDL933_RS18590:CDS | EDL933_RS18590:NZ_CP008957.1:3565373-3566432:+:-275 | EDL933_RS18590(espW) |
| NZ_CP008957.1 | 5371633 | 5372198 | 566 | 5371891 | 1479 | 113.52944 | 1.92102 | 111.68713 | IP_LBN_vs_In_LBN_peak_558 | EDL933_RS27700:Promoter;EDL933_RS27690:Promoter;EDL933_RS27695:Promoter | EDL933_RS33895:NZ_CP008957.1:5371834-5371888:+:81 | EDL933_RS33895(mgtL) |
| NZ_CP008957.1 | 2826031 | 2826424 | 394 | 2826238 | 1147 | 87.68483 | 1.91474 | 86.00259 | IP_LBN_vs_In_LBN_peak_243 | EDL933_RS14975:exon;EDL933_RS14965:Promoter;EDL933_RS14970:Promoter | EDL933_RS14975:NZ_CP008957.1:2826191-2826267:+:36 | EDL933_RS14975(EDL933_RS14975) |
| NZ_CP008957.1 | 1144347 | 1145141 | 795 | 1144451 | 1145 | 87.11872 | 1.9114 | 85.44054 | IP_LBN_vs_In_LBN_peak_134 | EDL933_RS05640:Promoter | EDL933_RS05645:NZ_CP008957.1:1144633-1144852:-:108 | EDL933_RS05645(infA) |
| NZ_CP008957.1 | 311713 | 315477 | 3765 | 314045 | 1265 | 96.04578 | 1.91135 | 94.30684 | IP_LBN_vs_In_LBN_peak_28 | EDL933_RS01535:Promoter;EDL933_RS01530:Promoter;EDL933_RS01525:CDS | EDL933_RS01525:NZ_CP008957.1:313448-314081:+:146 | EDL933_RS01525(EDL933_RS01525) |
| NZ_CP008957.1 | 2762674 | 2764280 | 1607 | 2763152 | 1144 | 86.83623 | 1.90973 | 85.16017 | IP_LBN_vs_In_LBN_peak_236 | EDL933_RS30800:Promoter;EDL933_RS14600:CDS | EDL933_RS14600:NZ_CP008957.1:2763035-2763689:+:441 | EDL933_RS14600(espJ) |
| NZ_CP008957.1 | 4092241 | 4097397 | 5157 | 4095807 | 1379 | 103.49621 | 1.90447 | 101.71278 | IP_LBN_vs_In_LBN_peak_383 | EDL933_RS21325:CDS;EDL933_RS21310:Promoter | EDL933_RS21320:NZ_CP008957.1:4095109-4095649:+:-290 | EDL933_RS21320(EDL933_RS21320) |
| NZ_CP008957.1 | 3592055 | 3598692 | 6638 | 3594083 | 1225 | 91.20577 | 1.8974 | 89.4981 | IP_LBN_vs_In_LBN_peak_332 | EDL933_RS34180:Promoter;EDL933_RS29265:Promoter;EDL933_RS31105:Promoter;EDL933_RS18750:CDS;EDL933_RS31115:Promoter | EDL933_RS18760:NZ_CP008957.1:3595567-3595643:-:270 | EDL933_RS18760(EDL933_RS18760) |
| NZ_CP008957.1 | 811802 | 813984 | 2183 | 813741 | 1134 | 84.03219 | 1.89305 | 82.37836 | IP_LBN_vs_In_LBN_peak_103 | EDL933_RS03785:Promoter;EDL933_RS03780:Promoter | EDL933_RS03775:NZ_CP008957.1:812822-813023:+:70 | EDL933_RS03775(EDL933_RS03775) |
| NZ_CP008957.1 | 667410 | 668232 | 823 | 667628 | 1134 | 84.03219 | 1.89305 | 82.37836 | IP_LBN_vs_In_LBN_peak_89 | EDL933_RS03085:CDS;EDL933_RS03080:Promoter;EDL933_RS03075:Promoter | EDL933_RS03085:NZ_CP008957.1:667393-667801:-:-19 | EDL933_RS03085(EDL933_RS03085) |
| NZ_CP008957.1 | 4725615 | 4728800 | 3186 | 4726456 | 1724 | 125.21769 | 1.88477 | 123.31911 | IP_LBN_vs_In_LBN_peak_467 | EDL933_RS24555:Promoter;EDL933_RS24550:Promoter;EDL933_RS24560:CDS | EDL933_RS24560:NZ_CP008957.1:4725643-4726747:-:-460 | EDL933_RS24560(EDL933_RS24560) |
| NZ_CP008957.1 | 4709993 | 4712058 | 2066 | 4710514 | 1606 | 115.53526 | 1.87764 | 113.68239 | IP_LBN_vs_In_LBN_peak_463 | EDL933_RS34215:Promoter;EDL933_RS24470:CDS;EDL933_RS24485:Promoter | EDL933_RS34205:NZ_CP008957.1:4711560-4711626:-:601 | EDL933_RS34205(EDL933_RS34205) |
| NZ_CP008957.1 | 5116371 | 5118882 | 2512 | 5117371 | 1470 | 105.80881 | 1.877 | 104.01035 | IP_LBN_vs_In_LBN_peak_520 | EDL933_RS26475:CDS | EDL933_RS26475:NZ_CP008957.1:5116327-5118514:-:888 | EDL933_RS26475(espL4) |
| NZ_CP008957.1 | 5183763 | 5185281 | 1519 | 5184644 | 1482 | 105.6457 | 1.87071 | 103.8483 | IP_LBN_vs_In_LBN_peak_531 | EDL933_RS26780:CDS | EDL933_RS26780:NZ_CP008957.1:5183793-5185086:-:564 | EDL933_RS26780(espX5) |
| NZ_CP008957.1 | 4472671 | 4476024 | 3354 | 4474393 | 1551 | 109.97989 | 1.86777 | 108.15723 | IP_LBN_vs_In_LBN_peak_430 | EDL933_RS23350:Promoter;EDL933_RS23345:Promoter | EDL933_RS23345:NZ_CP008957.1:4474697-4475264:+:-350 | EDL933_RS23345(slp) |
| NZ_CP008957.1 | 1323594 | 1324031 | 438 | 1323806 | 1362 | 96.42458 | 1.86518 | 94.68327 | IP_LBN_vs_In_LBN_peak_160 | EDL933_RS06570:Promoter;EDL933_RS06550:Promoter;EDL933_RS06560:Promoter;EDL933_RS06565:Promoter | EDL933_RS06560:NZ_CP008957.1:1323086-1323779:-:-33 | EDL933_RS06560(torR) |
| NZ_CP008957.1 | 3431859 | 3432994 | 1136 | 3432304 | 1125 | 79.64684 | 1.86256 | 78.02763 | IP_LBN_vs_In_LBN_peak_306 | EDL933_RS34045:CDS;EDL933_RS17960:Promoter;EDL933_RS17970:Promoter;EDL933_RS17950:Promoter;EDL933_RS17965:Promoter | EDL933_RS17960:NZ_CP008957.1:3432322-3432514:+:104 | EDL933_RS17960(EDL933_RS17960) |
| NZ_CP008957.1 | 3743681 | 3744839 | 1159 | 3744540 | 1236 | 87.18127 | 1.8615 | 85.50272 | IP_LBN_vs_In_LBN_peak_351 | EDL933_RS19515:Promoter;EDL933_RS19525:CDS;EDL933_RS19520:Promoter;EDL933_RS19530:Promoter | EDL933_RS19525:NZ_CP008957.1:3744260-3745577:+:0 | EDL933_RS19525(fucP) |
| NZ_CP008957.1 | 4493998 | 4495912 | 1915 | 4495522 | 1552 | 108.42099 | 1.85809 | 106.6073 | IP_LBN_vs_In_LBN_peak_432 | EDL933_RS23440:Promoter;EDL933_RS23445:CDS | EDL933_RS23440:NZ_CP008957.1:4493648-4494377:-:-577 | EDL933_RS23440(gadW) |
| NZ_CP008957.1 | 4757507 | 4758980 | 1474 | 4758259 | 1623 | 112.33896 | 1.85266 | 110.50296 | IP_LBN_vs_In_LBN_peak_470 | EDL933_RS24735:Promoter;EDL933_RS24745:Promoter;EDL933_RS24740:CDS;EDL933_RS24755:Promoter;EDL933_RS24750:Promoter | EDL933_RS24740:NZ_CP008957.1:4758192-4758333:+:51 | EDL933_RS24740(rpmH) |
| NZ_CP008957.1 | 3344571 | 3345129 | 559 | 3344781 | 1105 | 76.11731 | 1.84469 | 74.52576 | IP_LBN_vs_In_LBN_peak_303 | EDL933_RS17545:Promoter;EDL933_RS17550:Promoter | EDL933_RS17545:NZ_CP008957.1:3345045-3345303:+:-195 | EDL933_RS17545(ptsH) |
| NZ_CP008957.1 | 4279900 | 4282350 | 2451 | 4282135 | 1359 | 92.71942 | 1.84146 | 91.00201 | IP_LBN_vs_In_LBN_peak_411 | EDL933_RS22375:Promoter;EDL933_RS22365:Promoter;EDL933_RS22370:Promoter | EDL933_RS22365:NZ_CP008957.1:4278932-4281047:-:-77 | EDL933_RS22365(fusA) |
| NZ_CP008957.1 | 5161942 | 5163719 | 1778 | 5162582 | 1452 | 98.4561 | 1.8384 | 96.70318 | IP_LBN_vs_In_LBN_peak_527 | EDL933_RS26685:CDS;EDL933_RS26680:Promoter;EDL933_RS26690:Promoter | EDL933_RS26685:NZ_CP008957.1:5162370-5163327:+:460 | EDL933_RS26685(EDL933_RS26685) |
| NZ_CP008957.1 | 5383694 | 5385093 | 1400 | 5384143 | 1388 | 92.32843 | 1.82596 | 90.61346 | IP_LBN_vs_In_LBN_peak_560 | EDL933_RS27765:Promoter;EDL933_RS27770:CDS;EDL933_RS27755:Promoter | EDL933_RS27770:NZ_CP008957.1:5384011-5385208:+:382 | EDL933_RS27770(EDL933_RS27770) |
| NZ_CP008958.1 | 38720 | 40718 | 1999 | 39167 | 2479 | 163.18114 | 1.82509 | 161.16327 | IP_LBN_vs_In_LBN_peak_599 | EDL933_RS28775:Promoter;EDL933_RS28780:Promoter | EDL933_RS28780:NZ_CP008958.1:39820-42817:+:-101 | EDL933_RS28780(ehxA) |
| NZ_CP008957.1 | 1354202 | 1357054 | 2853 | 1354395 | 3171 | 207.54324 | 1.82323 | 205.45291 | IP_LBN_vs_In_LBN_peak_170 | EDL933_RS06830:Promoter;EDL933_RS06815:Promoter;EDL933_RS06835:Promoter;EDL933_RS06820:Promoter;EDL933_RS06825:Promoter | EDL933_RS06835:NZ_CP008957.1:1355915-1356185:+:-287 | EDL933_RS06835(EDL933_RS06835) |
| NZ_CP008957.1 | 620518 | 621812 | 1295 | 621322 | 1160 | 76.85913 | 1.82117 | 75.26204 | IP_LBN_vs_In_LBN_peak_77 | intergenic | EDL933_RS32000:NZ_CP008957.1:620924-621185:+:240 | EDL933_RS32000(EDL933_RS32000) |
| NZ_CP008957.1 | 4884476 | 4887341 | 2866 | 4885763 | 1618 | 106.18121 | 1.81944 | 104.38054 | IP_LBN_vs_In_LBN_peak_492 | EDL933_RS25360:Promoter;EDL933_RS25365:CDS;EDL933_RS25375:Promoter;EDL933_RS25370:Promoter | EDL933_RS25365:NZ_CP008957.1:4885072-4885921:-:13 | EDL933_RS25365(EDL933_RS25365) |
| NZ_CP008957.1 | 4627273 | 4628399 | 1127 | 4628092 | 1503 | 97.42519 | 1.81118 | 95.67818 | IP_LBN_vs_In_LBN_peak_454 | EDL933_RS24010:Promoter;EDL933_RS24015:Promoter;EDL933_RS24005:CDS;EDL933_RS24000:Promoter | EDL933_RS24005:NZ_CP008957.1:4627282-4628140:-:304 | EDL933_RS24005(yibB) |
| NZ_CP008957.1 | 2954489 | 2956640 | 2152 | 2955879 | 1083 | 70.33194 | 1.80799 | 68.78079 | IP_LBN_vs_In_LBN_peak_258 | EDL933_RS15590:CDS;EDL933_RS15580:Promoter;EDL933_RS15585:Promoter | EDL933_RS15585:NZ_CP008957.1:2954968-2955643:-:79 | EDL933_RS15585(EDL933_RS15585) |
| NZ_CP008957.1 | 4790682 | 4793015 | 2334 | 4791282 | 1619 | 103.95049 | 1.8063 | 102.16384 | IP_LBN_vs_In_LBN_peak_477 | EDL933_RS24895:Promoter;EDL933_RS24900:CDS;EDL933_RS24890:Promoter | EDL933_RS24905:NZ_CP008957.1:4791605-4792208:-:360 | EDL933_RS24905(lpfA2) |
| NZ_CP008957.1 | 5175802 | 5176270 | 469 | 5176080 | 1401 | 88.84862 | 1.79736 | 87.15755 | IP_LBN_vs_In_LBN_peak_529 | EDL933_RS26745:Promoter;EDL933_RS26755:Promoter;EDL933_RS26740:Promoter | EDL933_RS26745:NZ_CP008957.1:5176137-5176674:+:-101 | EDL933_RS26745(ssb1) |
| NZ_CP008957.1 | 5413906 | 5416084 | 2179 | 5415440 | 1415 | 89.38131 | 1.79515 | 87.68668 | IP_LBN_vs_In_LBN_peak_567 | EDL933_RS27905:CDS;EDL933_RS27910:Promoter | EDL933_RS27910:NZ_CP008957.1:5416126-5422441:+:-1131 | EDL933_RS27910(EDL933_RS27910) |
| NZ_CP008957.1 | 1406661 | 1407043 | 383 | 1406890 | 1126 | 71.44206 | 1.79474 | 69.88378 | IP_LBN_vs_In_LBN_peak_179 | EDL933_RS07090:Promoter;EDL933_RS07100:CDS | EDL933_RS07100:NZ_CP008957.1:1406551-1408060:+:300 | EDL933_RS07100(putP) |
| NZ_CP008957.1 | 5444156 | 5446849 | 2694 | 5444660 | 1335 | 83.59355 | 1.78933 | 81.94335 | IP_LBN_vs_In_LBN_peak_571 | EDL933_RS27985:Promoter;EDL933_RS27980:Promoter;EDL933_RS27965:Promoter | EDL933_RS27985:NZ_CP008957.1:5445828-5446425:+:-326 | EDL933_RS27985(fimE) |
| NZ_CP008957.1 | 4642731 | 4643560 | 830 | 4643252 | 1688 | 104.12517 | 1.78332 | 102.33754 | IP_LBN_vs_In_LBN_peak_456 | EDL933_RS24095:Promoter;EDL933_RS24075:Promoter;EDL933_RS24080:Promoter;EDL933_RS24085:Promoter | EDL933_RS24085:NZ_CP008957.1:4642920-4643157:-:12 | EDL933_RS24085(rpmB) |
| NZ_CP008957.1 | 852814 | 857098 | 4285 | 855500 | 1068 | 66.49786 | 1.78297 | 64.9724 | IP_LBN_vs_In_LBN_peak_107 | EDL933_RS03970:Promoter;EDL933_RS03960:Promoter;EDL933_RS03975:Promoter;EDL933_RS03965:Promoter | EDL933_RS03965:NZ_CP008957.1:853990-854935:-:-20 | EDL933_RS03965(EDL933_RS03965) |
| NZ_CP008957.1 | 3297970 | 3301654 | 3685 | 3298777 | 1067 | 66.24547 | 1.78131 | 64.72182 | IP_LBN_vs_In_LBN_peak_294 | EDL933_RS17315:Promoter;EDL933_RS17305:Promoter;EDL933_RS17310:Promoter;EDL933_RS17300:Promoter | EDL933_RS17315:NZ_CP008957.1:3299515-3303109:+:296 | EDL933_RS17315(evgS) |
| NZ_CP008957.1 | 5458905 | 5460596 | 1692 | 5460238 | 1297 | 79.69241 | 1.77806 | 78.07291 | IP_LBN_vs_In_LBN_peak_574 | EDL933_RS28075:Promoter;EDL933_RS33905:Promoter;EDL933_RS28060:Promoter | EDL933_RS28060:NZ_CP008957.1:5459195-5460026:-:276 | EDL933_RS28060(EDL933_RS28060) |
| NZ_CP008957.1 | 2451396 | 2451820 | 425 | 2451627 | 1065 | 65.74192 | 1.77797 | 64.22147 | IP_LBN_vs_In_LBN_peak_218 | EDL933_RS12865:CDS | EDL933_RS12865:NZ_CP008957.1:2451593-2451830:+:14 | EDL933_RS12865(lpp) |
| NZ_CP008957.1 | 3954391 | 3956908 | 2518 | 3955405 | 1301 | 78.72177 | 1.7694 | 77.10991 | IP_LBN_vs_In_LBN_peak_370 | EDL933_RS20595:Promoter;EDL933_RS20590:CDS | EDL933_RS20595:NZ_CP008957.1:3956133-3956961:+:-484 | EDL933_RS20595(EDL933_RS20595) |
| NZ_CP008957.1 | 1394452 | 1395155 | 704 | 1394806 | 3935 | 232.88173 | 1.766 | 230.75378 | IP_LBN_vs_In_LBN_peak_178 | EDL933_RS07040:Promoter;EDL933_RS07035:Promoter;EDL933_RS07045:Promoter;EDL933_RS29915:Promoter | EDL933_RS07045:NZ_CP008957.1:1393948-1394578:-:-225 | EDL933_RS07045(EDL933_RS07045) |
| NZ_CP008957.1 | 5472999 | 5475762 | 2764 | 5473728 | 1404 | 82.43068 | 1.75357 | 80.78989 | IP_LBN_vs_In_LBN_peak_576 | EDL933_RS28125:Promoter;EDL933_RS28135:CDS;EDL933_RS28130:Promoter | EDL933_RS28135:NZ_CP008957.1:5473148-5475344:-:964 | EDL933_RS28135(espX6) |
| NZ_CP008957.1 | 1664694 | 1671568 | 6875 | 1669030 | 1048 | 61.52756 | 1.74962 | 60.03372 | IP_LBN_vs_In_LBN_peak_199 | EDL933_RS08565:CDS | EDL933_RS08565:NZ_CP008957.1:1667679-1671081:+:451 | EDL933_RS08565(espN) |
| NZ_CP008957.1 | 46149 | 46920 | 772 | 46677 | 1214 | 70.53566 | 1.74605 | 68.98325 | IP_LBN_vs_In_LBN_peak_3 | EDL933_RS00225:Promoter;EDL933_RS00230:Promoter;EDL933_RS00235:Promoter;EDL933_RS00240:Promoter;EDL933_RS00220:Promoter | EDL933_RS00225:NZ_CP008957.1:44823-46338:-:-196 | EDL933_RS00225(caiT) |
| NZ_CP008957.1 | 270939 | 272503 | 1565 | 271796 | 1134 | 65.68573 | 1.74344 | 64.16579 | IP_LBN_vs_In_LBN_peak_19 | EDL933_RS01265:Promoter;EDL933_RS01250:CDS | EDL933_RS01250:NZ_CP008957.1:271361-271907:+:359 | EDL933_RS01250(EDL933_RS01250) |
| NZ_CP008957.1 | 1235791 | 1237531 | 1741 | 1236874 | 1171 | 67.64574 | 1.74243 | 66.11259 | IP_LBN_vs_In_LBN_peak_148 | EDL933_RS06020:Promoter;EDL933_RS06005:Promoter;EDL933_RS05995:Promoter | EDL933_RS06005:NZ_CP008957.1:1235815-1236856:-:195 | EDL933_RS06005(ompA) |
| NZ_CP008957.1 | 308781 | 309807 | 1027 | 309435 | 1131 | 65.30877 | 1.74172 | 63.79114 | IP_LBN_vs_In_LBN_peak_27 | EDL933_RS01485:Promoter;EDL933_RS01505:Promoter;EDL933_RS01490:CDS | EDL933_RS01490:NZ_CP008957.1:309100-309844:+:193 | EDL933_RS01490(EDL933_RS01490) |
| NZ_CP008957.1 | 4677596 | 4678428 | 833 | 4677924 | 1498 | 83.67583 | 1.72759 | 82.02507 | IP_LBN_vs_In_LBN_peak_458 | EDL933_RS24270:Promoter;EDL933_RS24275:CDS | EDL933_RS24275:NZ_CP008957.1:4677711-4677990:-:-21 | EDL933_RS24275(escG) |
| NZ_CP008957.1 | 3552787 | 3553543 | 757 | 3553244 | 1064 | 59.60254 | 1.72467 | 58.12146 | IP_LBN_vs_In_LBN_peak_324 | EDL933_RS18520:Promoter;EDL933_RS18530:Promoter;EDL933_RS18515:Promoter;EDL933_RS18505:Promoter;EDL933_RS18510:Promoter | EDL933_RS18520:NZ_CP008957.1:3552945-3553194:-:29 | EDL933_RS18520(rpsP) |
| NZ_CP008957.1 | 665585 | 666297 | 713 | 665826 | 1033 | 57.90785 | 1.7246 | 56.43846 | IP_LBN_vs_In_LBN_peak_88 | EDL933_RS03075:CDS;EDL933_RS03070:Promoter;EDL933_RS03065:Promoter | EDL933_RS03075:NZ_CP008957.1:665642-665852:-:-88 | EDL933_RS03075(EDL933_RS03075) |
| NZ_CP008957.1 | 5265183 | 5266135 | 953 | 5265983 | 1285 | 71.16986 | 1.72133 | 69.61316 | IP_LBN_vs_In_LBN_peak_541 | EDL933_RS27145:Promoter;EDL933_RS27155:Promoter | EDL933_RS27155:NZ_CP008957.1:5265867-5265943:-:284 | EDL933_RS27155(EDL933_RS27155) |
| NZ_CP008957.1 | 4200190 | 4201851 | 1662 | 4201598 | 1226 | 67.9312 | 1.72094 | 66.39612 | IP_LBN_vs_In_LBN_peak_401 | EDL933_RS21860:Promoter;EDL933_RS21865:Promoter;EDL933_RS21880:Promoter;EDL933_RS21870:Promoter;EDL933_RS21855:Promoter | EDL933_RS21865:NZ_CP008957.1:4200677-4201070:-:50 | EDL933_RS21865(rpsI) |
| NZ_CP008957.1 | 1608358 | 1608671 | 314 | 1608504 | 1029 | 56.95842 | 1.71793 | 55.49544 | IP_LBN_vs_In_LBN_peak_197 | EDL933_RS08150:Promoter;EDL933_RS08160:Promoter;EDL933_RS08155:Promoter;EDL933_RS08165:Promoter;EDL933_RS30165:CDS;EDL933_RS08145:Promoter | EDL933_RS30165:NZ_CP008957.1:1608413-1608602:-:88 | EDL933_RS30165(EDL933_RS30165) |
| NZ_CP008957.1 | 325236 | 326376 | 1141 | 325683 | 1123 | 61.35767 | 1.71249 | 59.86486 | IP_LBN_vs_In_LBN_peak_31 | EDL933_RS01610:Promoter;EDL933_RS01590:Promoter;EDL933_RS01600:CDS | EDL933_RS01600:NZ_CP008957.1:325543-325822:+:262 | EDL933_RS01600(EDL933_RS01600) |
| NZ_CP008957.1 | 5170273 | 5171431 | 1159 | 5171000 | 1376 | 73.34445 | 1.70255 | 71.77341 | IP_LBN_vs_In_LBN_peak_528 | EDL933_RS26735:Promoter;EDL933_RS26730:Promoter;EDL933_RS26725:Promoter;EDL933_RS26720:CDS | EDL933_RS26720:NZ_CP008957.1:5170665-5171025:-:173 | EDL933_RS26720(EDL933_RS26720) |
| NZ_CP008957.1 | 3757272 | 3757919 | 648 | 3757763 | 1058 | 56.33581 | 1.69853 | 54.87725 | IP_LBN_vs_In_LBN_peak_353 | EDL933_RS19580:Promoter;EDL933_RS19610:Promoter;EDL933_RS19585:Promoter | EDL933_RS19600:NZ_CP008957.1:3757632-3757709:+:-37 | EDL933_RS19600(EDL933_RS19600) |
| NZ_CP008957.1 | 1341986 | 1342663 | 678 | 1342218 | 3641 | 188.96947 | 1.69704 | 186.90668 | IP_LBN_vs_In_LBN_peak_163 | EDL933_RS06705:CDS;EDL933_RS33185:Promoter;EDL933_RS33180:Promoter;EDL933_RS06685:Promoter;EDL933_RS06700:Promoter;EDL933_RS06680:Promoter | EDL933_RS06705:NZ_CP008957.1:1342018-1342489:-:165 | EDL933_RS06705(EDL933_RS06705) |
| NZ_CP008957.1 | 400600 | 402266 | 1667 | 402003 | 1079 | 57.07973 | 1.69555 | 55.61602 | IP_LBN_vs_In_LBN_peak_49 | EDL933_RS01960:Promoter;EDL933_RS33025:Promoter | EDL933_RS01945:NZ_CP008957.1:400871-401687:+:561 | EDL933_RS01945(EDL933_RS01945) |
| NZ_CP008957.1 | 3226237 | 3226983 | 747 | 3226506 | 1017 | 53.62066 | 1.69299 | 52.18111 | IP_LBN_vs_In_LBN_peak_285 | EDL933_RS16935:Promoter;EDL933_RS16960:Promoter;EDL933_RS16950:Promoter;EDL933_RS16965:Promoter;EDL933_RS16940:Promoter;EDL933_RS16945:Promoter | EDL933_RS16960:NZ_CP008957.1:3226645-3227848:+:-35 | EDL933_RS16960(ackA) |
| NZ_CP008957.1 | 379143 | 379983 | 841 | 379613 | 1061 | 55.75727 | 1.692 | 54.30283 | IP_LBN_vs_In_LBN_peak_44 | EDL933_RS01855:Promoter | EDL933_RS01855:NZ_CP008957.1:379733-383717:+:-170 | EDL933_RS01855(ehaA) |
| NZ_CP008957.1 | 1012299 | 1012591 | 293 | 1012457 | 1013 | 53.2281 | 1.69124 | 51.79095 | IP_LBN_vs_In_LBN_peak_125 | EDL933_RS04835:Promoter;EDL933_RS04845:Promoter;EDL933_RS04840:Promoter | EDL933_RS04845:NZ_CP008957.1:1012480-1013017:+:-35 | EDL933_RS04845(rcdA) |
| NZ_CP008957.1 | 351775 | 352717 | 943 | 352162 | 1121 | 58.71939 | 1.69119 | 57.24426 | IP_LBN_vs_In_LBN_peak_35 | EDL933_RS01720:Promoter;EDL933_RS01740:Promoter | EDL933_RS01730:NZ_CP008957.1:352241-352598:-:352 | EDL933_RS01730(EDL933_RS01730) |
| NZ_CP008957.1 | 1343306 | 1345235 | 1930 | 1344191 | 4100 | 209.90892 | 1.69093 | 207.81477 | IP_LBN_vs_In_LBN_peak_164 | EDL933_RS06710:Promoter;EDL933_RS06715:CDS;EDL933_RS34105:Promoter;EDL933_RS06705:Promoter | EDL933_RS06715:NZ_CP008957.1:1343586-1344633:-:363 | EDL933_RS06715(EDL933_RS06715) |
| NZ_CP008957.1 | 353370 | 354457 | 1088 | 353768 | 1098 | 57.3581 | 1.68955 | 55.89254 | IP_LBN_vs_In_LBN_peak_36 | EDL933_RS01730:Promoter;EDL933_RS01740:Promoter;EDL933_RS01735:Promoter | EDL933_RS01740:NZ_CP008957.1:353931-358185:+:-18 | EDL933_RS01740(fdeC) |
| NZ_CP008957.1 | 717815 | 719176 | 1362 | 718159 | 1009 | 52.31249 | 1.68457 | 50.88157 | IP_LBN_vs_In_LBN_peak_93 | EDL933_RS03290:Promoter;EDL933_RS03285:Promoter;EDL933_RS03295:CDS | EDL933_RS03295:NZ_CP008957.1:717962-718865:-:370 | EDL933_RS03295(EDL933_RS03295) |
| NZ_CP008958.1 | 21078 | 23263 | 2186 | 23010 | 2308 | 116.78505 | 1.68186 | 114.92544 | IP_LBN_vs_In_LBN_peak_596 | EDL933_RS28695:Promoter;EDL933_RS34065:Promoter | EDL933_RS33935:NZ_CP008958.1:22185-22374:+:-15 | EDL933_RS33935(EDL933_RS33935) |
| NZ_CP008957.1 | 5329755 | 5330559 | 805 | 5330324 | 1135 | 58.18726 | 1.68081 | 56.71593 | IP_LBN_vs_In_LBN_peak_554 | EDL933_RS34225:CDS;EDL933_RS27505:Promoter;EDL933_RS27520:Promoter | EDL933_RS34225:NZ_CP008957.1:5330285-5330579:+:-128 | EDL933_RS34225(EDL933_RS34225) |
| NZ_CP008957.1 | 5327460 | 5329035 | 1576 | 5327608 | 1296 | 65.49249 | 1.6756 | 63.97363 | IP_LBN_vs_In_LBN_peak_553 | EDL933_RS27480:Promoter;EDL933_RS27495:Promoter;EDL933_RS27485:Promoter;EDL933_RS27500:Promoter;EDL933_RS27490:Promoter | EDL933_RS27490:NZ_CP008957.1:5328111-5328426:+:136 | EDL933_RS27490(priB) |
| NZ_CP008957.1 | 3012659 | 3014431 | 1773 | 3014168 | 1075 | 54.38827 | 1.67365 | 52.94339 | IP_LBN_vs_In_LBN_peak_261 | EDL933_RS15870:Promoter;EDL933_RS15875:Promoter;EDL933_RS15880:Promoter | EDL933_RS15875:NZ_CP008957.1:3012808-3013078:-:-466 | EDL933_RS15875(EDL933_RS15875) |
| NZ_CP008957.1 | 1409697 | 1410696 | 1000 | 1410012 | 1055 | 53.22953 | 1.67205 | 51.79237 | IP_LBN_vs_In_LBN_peak_180 | EDL933_RS07105:Promoter;EDL933_RS07115:Promoter;EDL933_RS07120:Promoter | EDL933_RS07115:NZ_CP008957.1:1410733-1411564:+:-537 | EDL933_RS07115(EDL933_RS07115) |
| NZ_CP008957.1 | 736968 | 737842 | 875 | 737329 | 999 | 50.05348 | 1.66789 | 48.63754 | IP_LBN_vs_In_LBN_peak_95 | EDL933_RS03395:Promoter;EDL933_RS03405:Promoter;EDL933_RS03385:Promoter;EDL933_RS03390:Promoter | EDL933_RS03390:NZ_CP008957.1:737689-738250:+:-284 | EDL933_RS03390(pagP) |
| NZ_CP008957.1 | 5251908 | 5252710 | 803 | 5252175 | 1335 | 66.11188 | 1.66633 | 64.58916 | IP_LBN_vs_In_LBN_peak_537 | EDL933_RS27080:Promoter;EDL933_RS27085:Promoter | EDL933_RS27085:NZ_CP008957.1:5250719-5252060:-:-248 | EDL933_RS27085(dcuB) |
| NZ_CP008957.1 | 3200062 | 3202671 | 2610 | 3201188 | 997 | 49.60684 | 1.66455 | 48.19402 | IP_LBN_vs_In_LBN_peak_282 | EDL933_RS32635:Promoter;EDL933_RS16840:CDS;EDL933_RS16850:Promoter | EDL933_RS32635:NZ_CP008957.1:3201866-3202484:+:-500 | EDL933_RS32635(EDL933_RS32635) |
| NZ_CP008957.1 | 5442624 | 5443653 | 1030 | 5443386 | 1201 | 59.04382 | 1.66156 | 57.5667 | IP_LBN_vs_In_LBN_peak_570 | EDL933_RS27960:Promoter;EDL933_RS27955:Promoter;EDL933_RS27980:Promoter;EDL933_RS27965:Promoter | EDL933_RS27965:NZ_CP008957.1:5442576-5443293:-:155 | EDL933_RS27965(nanC) |
| NZ_CP008957.1 | 4299843 | 4300160 | 318 | 4299999 | 1246 | 61.02678 | 1.66029 | 59.53625 | IP_LBN_vs_In_LBN_peak_413 | EDL933_RS22495:Promoter;EDL933_RS22500:Promoter;EDL933_RS22480:Promoter;EDL933_RS22475:Promoter;EDL933_RS22485:Promoter;EDL933_RS22490:Promoter | EDL933_RS22495:NZ_CP008957.1:4300100-4301282:+:-99 | EDL933_RS22495(tsgA) |
| NZ_CP008957.1 | 4962644 | 4964803 | 2160 | 4963799 | 1409 | 68.7731 | 1.66017 | 67.23243 | IP_LBN_vs_In_LBN_peak_502 | EDL933_RS25755:Promoter;EDL933_RS25740:Promoter;EDL933_RS25735:CDS | EDL933_RS25740:NZ_CP008957.1:4963945-4964860:+:-222 | EDL933_RS25740(EDL933_RS25740) |
| NZ_CP008958.1 | 66386 | 66805 | 420 | 66600 | 2203 | 106.10432 | 1.65824 | 104.30402 | IP_LBN_vs_In_LBN_peak_604 | EDL933_RS28940:Promoter;EDL933_RS34250:Promoter;EDL933_RS28945:Promoter;EDL933_RS28935:Promoter | EDL933_RS28935:NZ_CP008958.1:66607-66838:+:-12 | EDL933_RS28935(EDL933_RS28935) |
| NZ_CP008957.1 | 1593431 | 1594712 | 1282 | 1593761 | 993 | 48.71877 | 1.65788 | 47.31201 | IP_LBN_vs_In_LBN_peak_195 | EDL933_RS08085:Promoter;EDL933_RS08090:Promoter;EDL933_RS08070:Promoter;EDL933_RS08080:Promoter;EDL933_RS08075:CDS | EDL933_RS08080:NZ_CP008957.1:1594196-1594370:+:-125 | EDL933_RS08080(rpmF) |
| NZ_CP008957.1 | 3285445 | 3287362 | 1918 | 3286421 | 991 | 48.27734 | 1.65455 | 46.87354 | IP_LBN_vs_In_LBN_peak_291 | EDL933_RS17260:Promoter;EDL933_RS17250:CDS | EDL933_RS17250:NZ_CP008957.1:3285677-3287090:-:687 | EDL933_RS17250(EDL933_RS17250) |
| NZ_CP008957.1 | 545744 | 547322 | 1579 | 546424 | 1078 | 52.23288 | 1.6533 | 50.8027 | IP_LBN_vs_In_LBN_peak_66 | EDL933_RS02645:CDS;EDL933_RS02630:Promoter;EDL933_RS02625:Promoter;EDL933_RS02635:Promoter;EDL933_RS02640:Promoter | EDL933_RS02640:NZ_CP008957.1:546154-546373:-:-159 | EDL933_RS02640(hha) |
| NZ_CP008957.1 | 5341815 | 5343066 | 1252 | 5342681 | 1222 | 58.7125 | 1.65113 | 57.23742 | IP_LBN_vs_In_LBN_peak_555 | EDL933_RS27570:CDS;EDL933_RS27560:Promoter;EDL933_RS27580:Promoter | EDL933_RS27570:NZ_CP008957.1:5342035-5342974:+:405 | EDL933_RS27570(EDL933_RS27570) |
| NZ_CP008957.1 | 1264146 | 1265703 | 1558 | 1264855 | 1132 | 54.43247 | 1.65048 | 52.98731 | IP_LBN_vs_In_LBN_peak_153 | EDL933_RS06225:Promoter;EDL933_RS06210:CDS;EDL933_RS06220:Promoter;EDL933_RS06215:Promoter;EDL933_RS33975:Promoter | EDL933_RS06210:NZ_CP008957.1:1264568-1265387:+:356 | EDL933_RS06210(EDL933_RS06210) |
| NZ_CP008957.1 | 5262939 | 5264292 | 1354 | 5263766 | 1300 | 62.13094 | 1.64949 | 60.63327 | IP_LBN_vs_In_LBN_peak_540 | EDL933_RS27140:Promoter;EDL933_RS27145:CDS;EDL933_RS27135:Promoter | EDL933_RS27140:NZ_CP008957.1:5262012-5263347:-:-268 | EDL933_RS27140(cadB) |
| NZ_CP008957.1 | 4686584 | 4687733 | 1150 | 4686880 | 1440 | 67.72033 | 1.64335 | 66.18675 | IP_LBN_vs_In_LBN_peak_460 | EDL933_RS24315:CDS | EDL933_RS24320:NZ_CP008957.1:4686945-4687416:-:258 | EDL933_RS24320(cesT) |
| NZ_CP008957.1 | 3276288 | 3277910 | 1623 | 3277709 | 983 | 46.52906 | 1.6412 | 45.13733 | IP_LBN_vs_In_LBN_peak_288 | EDL933_RS17210:Promoter | EDL933_RS17200:NZ_CP008957.1:3276429-3277461:+:669 | EDL933_RS17200(EDL933_RS17200) |
| NZ_CP008957.1 | 897582 | 899070 | 1489 | 898688 | 983 | 46.52906 | 1.6412 | 45.13733 | IP_LBN_vs_In_LBN_peak_112 | EDL933_RS04255:Promoter;EDL933_RS29620:Promoter;EDL933_RS04265:Promoter;EDL933_RS04260:Promoter | EDL933_RS04255:NZ_CP008957.1:897660-898620:-:294 | EDL933_RS04255(EDL933_RS04255) |
| NZ_CP008957.1 | 1598062 | 1598986 | 925 | 1598415 | 977 | 45.23627 | 1.6312 | 43.85362 | IP_LBN_vs_In_LBN_peak_196 | EDL933_RS08115:Promoter;EDL933_RS08110:Promoter;EDL933_RS08105:Promoter | EDL933_RS08105:NZ_CP008957.1:1598444-1598681:+:79 | EDL933_RS08105(acpP) |
| NZ_CP008957.1 | 4965146 | 4965995 | 850 | 4965632 | 1360 | 61.83733 | 1.62768 | 60.34145 | IP_LBN_vs_In_LBN_peak_503 | EDL933_RS25755:CDS;EDL933_RS25760:Promoter | EDL933_RS25755:NZ_CP008957.1:4965553-4965772:+:17 | EDL933_RS25755(EDL933_RS25755) |
| NZ_CP008957.1 | 798288 | 799315 | 1028 | 798545 | 970 | 43.7481 | 1.61952 | 42.3755 | IP_LBN_vs_In_LBN_peak_102 | EDL933_RS03720:CDS | EDL933_RS33065:NZ_CP008957.1:798880-798985:-:184 | EDL933_RS33065(EDL933_RS33065) |
| NZ_CP008957.1 | 3342042 | 3342755 | 714 | 3342573 | 966 | 42.90748 | 1.61285 | 41.54063 | IP_LBN_vs_In_LBN_peak_302 | EDL933_RS17530:Promoter;EDL933_RS17525:Promoter;EDL933_RS17540:Promoter;EDL933_RS17535:Promoter | EDL933_RS17530:NZ_CP008957.1:3341516-3342515:-:117 | EDL933_RS17530(zipA) |
| NZ_CP008957.1 | 5177383 | 5178656 | 1274 | 5178159 | 1255 | 55.2135 | 1.61244 | 53.76291 | IP_LBN_vs_In_LBN_peak_530 | EDL933_RS26750:Promoter;EDL933_RS26755:CDS;EDL933_RS26765:Promoter | EDL933_RS26755:NZ_CP008957.1:5177482-5179069:+:537 | EDL933_RS26755(pdeC) |
| NZ_CP008957.1 | 1440031 | 1442354 | 2324 | 1442033 | 978 | 43.14061 | 1.61007 | 41.77221 | IP_LBN_vs_In_LBN_peak_186 | EDL933_RS07230:Promoter;EDL933_RS07225:CDS;EDL933_RS07245:Promoter;EDL933_RS07235:Promoter;EDL933_RS07240:Promoter | EDL933_RS07225:NZ_CP008957.1:1441896-1442265:+:-704 | EDL933_RS07225(acpS) |
| NZ_CP008957.1 | 4676522 | 4676895 | 374 | 4676724 | 1222 | 53.06864 | 1.60659 | 51.63272 | IP_LBN_vs_In_LBN_peak_457 | intergenic | EDL933_RS33850:NZ_CP008957.1:4676333-4676649:+:375 | EDL933_RS33850(EDL933_RS33850) |
| NZ_CP008957.1 | 263335 | 264147 | 813 | 263794 | 1085 | 47.14157 | 1.60519 | 45.74578 | IP_LBN_vs_In_LBN_peak_18 | EDL933_RS01230:Promoter;EDL933_RS01215:Promoter;EDL933_RS01210:Promoter;EDL933_RS01240:Promoter;EDL933_RS01220:Promoter | EDL933_RS01220:NZ_CP008957.1:262936-263437:-:-303 | EDL933_RS01220(tssB) |
| NZ_CP008957.1 | 3711228 | 3712268 | 1041 | 3711725 | 1054 | 45.77335 | 1.60457 | 44.387 | IP_LBN_vs_In_LBN_peak_345 | EDL933_RS19395:Promoter;EDL933_RS19390:Promoter | EDL933_RS19385:NZ_CP008957.1:3712180-3712852:-:1104 | EDL933_RS19385(queE) |
| NZ_CP008957.1 | 3269029 | 3269642 | 614 | 3269269 | 959 | 41.4536 | 1.60117 | 40.09653 | IP_LBN_vs_In_LBN_peak_287 | EDL933_RS17170:Promoter;EDL933_RS17165:Promoter | EDL933_RS17170:NZ_CP008957.1:3268537-3269101:-:-234 | EDL933_RS17170(EDL933_RS17170) |
| NZ_CP008957.1 | 371989 | 373078 | 1090 | 372299 | 1045 | 44.48964 | 1.59613 | 43.11224 | IP_LBN_vs_In_LBN_peak_43 | EDL933_RS01815:Promoter;EDL933_RS01810:Promoter;EDL933_RS33980:Promoter;EDL933_RS01825:Promoter | EDL933_RS33980:NZ_CP008957.1:372407-372542:+:126 | EDL933_RS33980(EDL933_RS33980) |
| NZ_CP008957.1 | 2951350 | 2952640 | 1291 | 2952114 | 955 | 40.63269 | 1.5945 | 39.2816 | IP_LBN_vs_In_LBN_peak_257 | EDL933_RS15575:CDS | EDL933_RS15575:NZ_CP008957.1:2951427-2952462:-:467 | EDL933_RS15575(EDL933_RS15575) |
| NZ_CP008957.1 | 4042590 | 4042981 | 392 | 4042750 | 1115 | 47.17076 | 1.59444 | 45.77481 | IP_LBN_vs_In_LBN_peak_378 | EDL933_RS21080:Promoter;EDL933_RS21070:Promoter;EDL933_RS21065:Promoter | EDL933_RS21070:NZ_CP008957.1:4042755-4042831:+:30 | EDL933_RS21070(EDL933_RS21070) |
| NZ_CP008957.1 | 4101636 | 4102373 | 738 | 4102105 | 1140 | 47.98301 | 1.59264 | 46.58141 | IP_LBN_vs_In_LBN_peak_384 | EDL933_RS21350:Promoter;EDL933_RS21345:Promoter;EDL933_RS21355:Promoter;EDL933_RS21340:Promoter | EDL933_RS21350:NZ_CP008957.1:4102143-4103715:+:-139 | EDL933_RS21350(garD) |
| NZ_CP008957.1 | 4605515 | 4606925 | 1411 | 4606417 | 1321 | 55.16665 | 1.59131 | 53.7163 | IP_LBN_vs_In_LBN_peak_451 | EDL933_RS23920:CDS;EDL933_RS23925:Promoter;EDL933_RS23910:Promoter | EDL933_RS23920:NZ_CP008957.1:4606020-4606689:+:199 | EDL933_RS23920(EDL933_RS23920) |
| NZ_CP008957.1 | 3945224 | 3946718 | 1495 | 3946492 | 1070 | 44.81902 | 1.58977 | 43.43926 | IP_LBN_vs_In_LBN_peak_368 | EDL933_RS20530:Promoter;EDL933_RS32760:Promoter;EDL933_RS20535:Promoter | EDL933_RS20535:NZ_CP008957.1:3945454-3946003:-:32 | EDL933_RS20535(EDL933_RS20535) |
| NZ_CP008957.1 | 387753 | 388207 | 455 | 387911 | 1001 | 41.83892 | 1.58791 | 40.47948 | IP_LBN_vs_In_LBN_peak_47 | EDL933_RS01880:CDS;EDL933_RS01865:Promoter;EDL933_RS01890:Promoter;EDL933_RS01885:Promoter;EDL933_RS01870:Promoter | EDL933_RS01885:NZ_CP008957.1:388274-389822:+:-294 | EDL933_RS01885(fdrA) |
| NZ_CP008957.1 | 5486383 | 5487455 | 1073 | 5486952 | 1152 | 47.91282 | 1.5879 | 46.51163 | IP_LBN_vs_In_LBN_peak_579 | EDL933_RS28200:CDS;EDL933_RS28195:Promoter | EDL933_RS28195:NZ_CP008957.1:5486162-5486504:-:-414 | EDL933_RS28195(symE) |
| NZ_CP008957.1 | 3719469 | 3719920 | 452 | 3719751 | 1000 | 41.58137 | 1.58578 | 40.22361 | IP_LBN_vs_In_LBN_peak_348 | EDL933_RS19410:Promoter;EDL933_RS19415:Promoter | EDL933_RS19415:NZ_CP008957.1:3718053-3719691:-:-3 | EDL933_RS19415(pyrG) |
| NZ_CP008957.1 | 367319 | 368236 | 918 | 367929 | 1043 | 42.83059 | 1.58135 | 41.46444 | IP_LBN_vs_In_LBN_peak_40 | EDL933_RS34075:CDS | EDL933_RS34075:NZ_CP008957.1:367410-368079:-:302 | EDL933_RS34075(EDL933_RS34075) |
| NZ_CP008958.1 | 26151 | 26896 | 746 | 26581 | 2042 | 81.64575 | 1.57817 | 80.01107 | IP_LBN_vs_In_LBN_peak_598 | EDL933_RS28705:Promoter;EDL933_RS28700:CDS | EDL933_RS28705:NZ_CP008958.1:26673-28641:+:-150 | EDL933_RS28705(etpD) |
| NZ_CP008957.1 | 4580596 | 4581398 | 803 | 4580785 | 1278 | 51.70196 | 1.57809 | 50.2752 | IP_LBN_vs_In_LBN_peak_446 | EDL933_RS23820:Promoter;EDL933_RS23815:CDS | EDL933_RS23820:NZ_CP008957.1:4580948-4581923:+:48 | EDL933_RS23820(EDL933_RS23820) |
| NZ_CP008957.1 | 5526084 | 5526483 | 400 | 5526268 | 1172 | 47.38306 | 1.57672 | 45.98565 | IP_LBN_vs_In_LBN_peak_586 | EDL933_RS28400:Promoter;EDL933_RS28405:Promoter | EDL933_RS28400:NZ_CP008957.1:5526316-5527540:+:-33 | EDL933_RS28400(deoB) |
| NZ_CP008957.1 | 974230 | 974860 | 631 | 974445 | 942 | 38.01473 | 1.57282 | 36.68277 | IP_LBN_vs_In_LBN_peak_121 | EDL933_RS04670:Promoter;EDL933_RS33100:CDS;EDL933_RS04660:Promoter;EDL933_RS04665:Promoter | EDL933_RS04670:NZ_CP008957.1:974526-975042:+:18 | EDL933_RS04670(ompX) |
| NZ_CP008957.1 | 965807 | 966098 | 292 | 965949 | 942 | 38.01473 | 1.57282 | 36.68277 | IP_LBN_vs_In_LBN_peak_119 | EDL933_RS04615:Promoter;EDL933_RS04625:Promoter | EDL933_RS04615:NZ_CP008957.1:963470-965753:-:-199 | EDL933_RS04615(EDL933_RS04615) |
| NZ_CP008957.1 | 675199 | 675813 | 615 | 675538 | 942 | 38.01473 | 1.57282 | 36.68277 | IP_LBN_vs_In_LBN_peak_90 | EDL933_RS03100:Promoter | EDL933_RS03100:NZ_CP008957.1:673264-675166:-:-339 | EDL933_RS03100(EDL933_RS03100) |
| NZ_CP008957.1 | 4235274 | 4236636 | 1363 | 4236111 | 1181 | 47.05982 | 1.57109 | 45.66448 | IP_LBN_vs_In_LBN_peak_403 | EDL933_RS22045:Promoter;EDL933_RS33805:Promoter;EDL933_RS22050:Promoter | EDL933_RS22045:NZ_CP008957.1:4235388-4236051:-:96 | EDL933_RS22045(acrS) |
| NZ_CP008957.1 | 5307166 | 5307694 | 529 | 5307335 | 1182 | 47.09435 | 1.57106 | 45.69881 | IP_LBN_vs_In_LBN_peak_549 | EDL933_RS27380:Promoter;EDL933_RS27375:Promoter;EDL933_RS27370:CDS | EDL933_RS27370:NZ_CP008957.1:5307278-5308577:+:151 | EDL933_RS27370(purA) |
| NZ_CP008957.1 | 317419 | 318272 | 854 | 317604 | 1038 | 41.42671 | 1.56992 | 40.07012 | IP_LBN_vs_In_LBN_peak_29 | EDL933_RS01570:Promoter;EDL933_RS01575:Promoter;EDL933_RS01550:Promoter;EDL933_RS01540:Promoter;EDL933_RS01560:Promoter;EDL933_RS01580:Promoter;EDL933_RS01565:Promoter;EDL933_RS01545:Promoter | EDL933_RS01560:NZ_CP008957.1:318189-318450:+:-344 | EDL933_RS01560(EDL933_RS01560) |
| NZ_CP008957.1 | 2892340 | 2893012 | 673 | 2892640 | 940 | 37.6188 | 1.56948 | 36.28962 | IP_LBN_vs_In_LBN_peak_249 | EDL933_RS15305:Promoter;EDL933_RS15310:CDS;EDL933_RS15300:Promoter;EDL933_RS15295:Promoter | EDL933_RS15305:NZ_CP008957.1:2892022-2892571:-:-104 | EDL933_RS15305(wcaF) |
| NZ_CP008957.1 | 2806304 | 2806663 | 360 | 2806514 | 940 | 37.6188 | 1.56948 | 36.28962 | IP_LBN_vs_In_LBN_peak_237 | EDL933_RS14915:Promoter;EDL933_RS14910:Promoter;EDL933_RS14920:Promoter;EDL933_RS14905:exon | EDL933_RS14905:NZ_CP008957.1:2806498-2806588:-:105 | EDL933_RS14905(EDL933_RS14905) |
| NZ_CP008957.1 | 3133763 | 3134675 | 913 | 3134395 | 939 | 37.42153 | 1.56782 | 36.09365 | IP_LBN_vs_In_LBN_peak_276 | EDL933_RS16575:Promoter;EDL933_RS16570:Promoter;EDL933_RS16590:Promoter | EDL933_RS16575:NZ_CP008957.1:3132951-3134055:-:-163 | EDL933_RS16575(ompC) |
| NZ_CP008957.1 | 924434 | 925548 | 1115 | 925269 | 939 | 37.42153 | 1.56782 | 36.09365 | IP_LBN_vs_In_LBN_peak_113 | EDL933_RS04430:Promoter;EDL933_RS29640:Promoter;EDL933_RS04425:Promoter;EDL933_RS04420:CDS | EDL933_RS04425:NZ_CP008957.1:925405-926425:+:-414 | EDL933_RS04425(nleC) |
| NZ_CP008957.1 | 275388 | 277461 | 2074 | 275782 | 1066 | 42.1003 | 1.56619 | 40.73886 | IP_LBN_vs_In_LBN_peak_20 | EDL933_RS01280:CDS;EDL933_RS01285:Promoter | EDL933_RS01285:NZ_CP008957.1:276197-277370:+:227 | EDL933_RS01285(EDL933_RS01285) |
| NZ_CP008957.1 | 4017976 | 4018538 | 563 | 4018337 | 1052 | 40.69702 | 1.558 | 39.34558 | IP_LBN_vs_In_LBN_peak_374 | EDL933_RS20945:Promoter;EDL933_RS20950:Promoter;EDL933_RS34015:Promoter;EDL933_RS20965:Promoter;EDL933_RS20960:Promoter | EDL933_RS20945:NZ_CP008957.1:4017533-4018187:-:-69 | EDL933_RS20945(ribB) |
| NZ_CP008957.1 | 4158743 | 4159107 | 365 | 4158940 | 1148 | 43.79893 | 1.55392 | 42.42612 | IP_LBN_vs_In_LBN_peak_395 | EDL933_RS21650:Promoter;EDL933_RS21640:Promoter;EDL933_RS21655:Promoter;EDL933_RS21635:Promoter | EDL933_RS21650:NZ_CP008957.1:4158363-4158840:-:-84 | EDL933_RS21650(greA) |
| NZ_CP008957.1 | 3706951 | 3707603 | 653 | 3707317 | 1016 | 38.75688 | 1.55214 | 37.41978 | IP_LBN_vs_In_LBN_peak_343 | EDL933_RS19370:Promoter;EDL933_RS19360:Promoter;EDL933_RS19365:CDS;EDL933_RS19355:Promoter | EDL933_RS19360:NZ_CP008957.1:3705706-3707161:-:-115 | EDL933_RS19360(EDL933_RS19360) |
| NZ_CP008957.1 | 760376 | 760839 | 464 | 760656 | 927 | 35.0901 | 1.5478 | 33.7785 | IP_LBN_vs_In_LBN_peak_100 | EDL933_RS33055:Promoter;EDL933_RS03515:Promoter;EDL933_RS03520:CDS | EDL933_RS03520:NZ_CP008957.1:760644-761352:+:-37 | EDL933_RS03520(EDL933_RS03520) |
| NZ_CP008957.1 | 996895 | 998304 | 1410 | 997144 | 926 | 34.89883 | 1.54613 | 33.58858 | IP_LBN_vs_In_LBN_peak_124 | EDL933_RS04770:CDS | EDL933_RS04770:NZ_CP008957.1:997083-999432:+:516 | EDL933_RS04770(EDL933_RS04770) |
| NZ_CP008957.1 | 3939407 | 3939832 | 426 | 3939548 | 1023 | 38.32359 | 1.54542 | 36.98961 | IP_LBN_vs_In_LBN_peak_367 | EDL933_RS20505:Promoter;EDL933_RS20500:Promoter;EDL933_RS20490:Promoter | EDL933_RS20500:NZ_CP008957.1:3939585-3939661:+:34 | EDL933_RS20500(EDL933_RS20500) |
| NZ_CP008957.1 | 343242 | 344239 | 998 | 343940 | 1042 | 38.98868 | 1.54524 | 37.64997 | IP_LBN_vs_In_LBN_peak_32 | EDL933_RS01670:Promoter;EDL933_RS01700:Promoter;EDL933_RS01665:Promoter;EDL933_RS01675:Promoter;EDL933_RS01680:Promoter;EDL933_RS01695:Promoter | EDL933_RS01675:NZ_CP008957.1:343154-343697:-:-43 | EDL933_RS01675(ecpR) |
| NZ_CP008957.1 | 5366958 | 5370679 | 3722 | 5369482 | 1168 | 43.07804 | 1.54147 | 41.71033 | IP_LBN_vs_In_LBN_peak_557 | EDL933_RS27685:Promoter;EDL933_RS27690:CDS | EDL933_RS27685:NZ_CP008957.1:5367457-5369113:-:295 | EDL933_RS27685(treC) |
| NZ_CP008957.1 | 2947451 | 2948905 | 1455 | 2948514 | 922 | 34.13837 | 1.53946 | 32.83348 | IP_LBN_vs_In_LBN_peak_256 | EDL933_RS15555:Promoter;EDL933_RS15540:Promoter;EDL933_RS15550:Promoter;EDL933_RS15565:Promoter | EDL933_RS15550:NZ_CP008957.1:2948764-2949112:+:-586 | EDL933_RS15550(EDL933_RS15550) |
| NZ_CP008957.1 | 4407381 | 4408074 | 694 | 4407834 | 1223 | 44.48793 | 1.537 | 43.11054 | IP_LBN_vs_In_LBN_peak_421 | EDL933_RS22980:Promoter;EDL933_RS22985:Promoter | EDL933_RS22985:NZ_CP008957.1:4406799-4407654:-:-73 | EDL933_RS22985(rpoH) |
| NZ_CP008957.1 | 4018853 | 4020209 | 1357 | 4019980 | 1113 | 40.44002 | 1.53533 | 39.09034 | IP_LBN_vs_In_LBN_peak_375 | EDL933_RS20945:Promoter;EDL933_RS33800:Promoter;EDL933_RS20965:Promoter;EDL933_RS20955:Promoter | EDL933_RS20960:NZ_CP008957.1:4019363-4019978:+:167 | EDL933_RS20960(EDL933_RS20960) |
| NZ_CP008957.1 | 2752503 | 2753132 | 630 | 2752688 | 919 | 33.57292 | 1.53446 | 32.27182 | IP_LBN_vs_In_LBN_peak_233 | EDL933_RS33570:Promoter;EDL933_RS14530:Promoter;EDL933_RS14525:Promoter;EDL933_RS14535:Promoter | EDL933_RS33570:NZ_CP008957.1:2753058-2754245:+:-241 | EDL933_RS33570(ompC) |
| NZ_CP008957.1 | 3281247 | 3281967 | 721 | 3281541 | 919 | 33.57292 | 1.53446 | 32.27182 | IP_LBN_vs_In_LBN_peak_290 | EDL933_RS17225:CDS | EDL933_RS17225:NZ_CP008957.1:3281480-3282617:-:1010 | EDL933_RS17225(EDL933_RS17225) |
| NZ_CP008957.1 | 650034 | 651455 | 1422 | 650246 | 919 | 33.57292 | 1.53446 | 32.27182 | IP_LBN_vs_In_LBN_peak_83 | EDL933_RS03020:Promoter;EDL933_RS03010:Promoter;EDL933_RS03005:Promoter;EDL933_RS03015:Promoter;EDL933_RS03000:Promoter;EDL933_RS03025:Promoter | EDL933_RS03020:NZ_CP008957.1:651116-651809:+:-372 | EDL933_RS03020(fimC) |
| NZ_CP008957.1 | 5545277 | 5546471 | 1195 | 5546062 | 1048 | 38.0571 | 1.53428 | 36.72494 | IP_LBN_vs_In_LBN_peak_587 | EDL933_RS28505:Promoter;EDL933_RS28500:Promoter;EDL933_RS28495:Promoter | EDL933_RS28495:NZ_CP008957.1:5545260-5545977:-:103 | EDL933_RS28495(arcA) |
| NZ_CP008957.1 | 1333486 | 1333803 | 318 | 1333654 | 2539 | 89.82091 | 1.5338 | 88.12292 | IP_LBN_vs_In_LBN_peak_162 | EDL933_RS06610:CDS;EDL933_RS06605:Promoter;EDL933_RS06600:Promoter | EDL933_RS06605:NZ_CP008957.1:1332936-1333515:-:-129 | EDL933_RS06605(wrbA) |
| NZ_CP008957.1 | 2493360 | 2495165 | 1806 | 2494980 | 918 | 33.38538 | 1.53279 | 32.08559 | IP_LBN_vs_In_LBN_peak_219 | EDL933_RS13080:Promoter;EDL933_RS33520:Promoter;EDL933_RS13085:CDS;EDL933_RS13075:Promoter;EDL933_RS13070:Promoter;EDL933_RS13060:Promoter | EDL933_RS13075:NZ_CP008957.1:2493975-2494173:-:-89 | EDL933_RS13075(rpmI) |
| NZ_CP008957.1 | 450780 | 451483 | 704 | 451163 | 988 | 35.65161 | 1.5311 | 34.33649 | IP_LBN_vs_In_LBN_peak_52 | EDL933_RS02160:Promoter;EDL933_RS02165:Promoter | EDL933_RS02165:NZ_CP008957.1:451254-454197:+:-123 | EDL933_RS02165(ehaB) |
| NZ_CP008957.1 | 4979032 | 4980655 | 1624 | 4979375 | 1284 | 45.84094 | 1.53081 | 44.4542 | IP_LBN_vs_In_LBN_peak_506 | EDL933_RS25825:Promoter;EDL933_RS25820:Promoter;EDL933_RS25815:Promoter | EDL933_RS25825:NZ_CP008957.1:4979550-4980945:+:293 | EDL933_RS25825(EDL933_RS25825) |
| NZ_CP008957.1 | 360754 | 361641 | 888 | 361231 | 973 | 34.81718 | 1.52784 | 33.50772 | IP_LBN_vs_In_LBN_peak_37 | EDL933_RS01770:Promoter;EDL933_RS01760:CDS;EDL933_RS01755:Promoter | EDL933_RS01760:NZ_CP008957.1:361045-361282:-:85 | EDL933_RS01760(EDL933_RS01760) |
| NZ_CP008957.1 | 3551288 | 3552447 | 1160 | 3552162 | 919 | 32.7083 | 1.525 | 31.41342 | IP_LBN_vs_In_LBN_peak_323 | EDL933_RS18510:CDS;EDL933_RS18505:Promoter | EDL933_RS18505:NZ_CP008957.1:3551191-3551539:-:-328 | EDL933_RS18505(rplS) |
| NZ_CP008957.1 | 4514215 | 4514508 | 294 | 4514369 | 1205 | 42.31583 | 1.52412 | 40.953 | IP_LBN_vs_In_LBN_peak_437 | EDL933_RS23515:CDS;EDL933_RS23510:Promoter | EDL933_RS23515:NZ_CP008957.1:4513391-4515380:-:1019 | EDL933_RS23515(hmsP) |
| NZ_CP008957.1 | 5283186 | 5285673 | 2488 | 5285480 | 1171 | 40.70891 | 1.52018 | 39.3574 | IP_LBN_vs_In_LBN_peak_545 | EDL933_RS27260:Promoter;EDL933_RS27275:Promoter;EDL933_RS27270:Promoter;EDL933_RS27265:Promoter | EDL933_RS27260:NZ_CP008957.1:5282928-5283663:-:-766 | EDL933_RS27260(frdB) |
| NZ_CP008957.1 | 1707347 | 1709251 | 1905 | 1707631 | 910 | 31.90193 | 1.51945 | 30.61284 | IP_LBN_vs_In_LBN_peak_200 | EDL933_RS33305:CDS;EDL933_RS08830:Promoter | EDL933_RS08830:NZ_CP008957.1:1707725-1709252:+:573 | EDL933_RS08830(EDL933_RS08830) |
| NZ_CP008957.1 | 3707936 | 3708629 | 694 | 3708334 | 973 | 33.90891 | 1.51841 | 32.60585 | IP_LBN_vs_In_LBN_peak_344 | EDL933_RS19375:Promoter;EDL933_RS19370:Promoter;EDL933_RS19360:Promoter;EDL933_RS19365:Promoter | EDL933_RS19370:NZ_CP008957.1:3708334-3709612:+:-52 | EDL933_RS19370(EDL933_RS19370) |
| NZ_CP008957.1 | 3543780 | 3544684 | 905 | 3544033 | 907 | 31.35342 | 1.51444 | 30.06835 | IP_LBN_vs_In_LBN_peak_321 | EDL933_RS18450:Promoter;EDL933_RS18470:Promoter;EDL933_RS33705:Promoter;EDL933_RS18465:Promoter;EDL933_RS18445:Promoter | EDL933_RS18465:NZ_CP008957.1:3544161-3544503:+:70 | EDL933_RS18465(raiA) |
| NZ_CP008957.1 | 4545594 | 4546064 | 471 | 4545895 | 1209 | 41.29056 | 1.51438 | 39.93471 | IP_LBN_vs_In_LBN_peak_441 | EDL933_RS23645:Promoter;EDL933_RS23655:Promoter;EDL933_RS23650:Promoter | EDL933_RS23655:NZ_CP008957.1:4545134-4545659:-:-169 | EDL933_RS23655(lpfA1) |
| NZ_CP008957.1 | 1960985 | 1961228 | 244 | 1961150 | 906 | 31.17153 | 1.51278 | 29.88771 | IP_LBN_vs_In_LBN_peak_205 | EDL933_RS10360:Promoter;EDL933_RS10350:Promoter | EDL933_RS10350:NZ_CP008957.1:1961192-1962644:+:-86 | EDL933_RS10350(uxaB) |
| NZ_CP008957.1 | 2900845 | 2901626 | 782 | 2901344 | 905 | 30.99012 | 1.51111 | 29.70772 | IP_LBN_vs_In_LBN_peak_251 | EDL933_RS15350:Promoter;EDL933_RS15345:Promoter;EDL933_RS15340:Promoter;EDL933_RS15355:Promoter;EDL933_RS34165:CDS | EDL933_RS34165:NZ_CP008957.1:2901234-2901384:+:1 | EDL933_RS34165(EDL933_RS34165) |
| NZ_CP008957.1 | 3308280 | 3308908 | 629 | 3308683 | 903 | 30.62871 | 1.50777 | 29.34898 | IP_LBN_vs_In_LBN_peak_298 | EDL933_RS17350:Promoter;EDL933_RS17340:Promoter;EDL933_RS17335:Promoter | EDL933_RS17340:NZ_CP008957.1:3307141-3308392:-:-201 | EDL933_RS17340(frc) |
| NZ_CP008957.1 | 2972306 | 2972724 | 419 | 2972455 | 903 | 30.62871 | 1.50777 | 29.34898 | IP_LBN_vs_In_LBN_peak_260 | EDL933_RS15660:Promoter;EDL933_RS15655:Promoter | EDL933_RS15655:NZ_CP008957.1:2972588-2973677:+:-73 | EDL933_RS15655(EDL933_RS15655) |
| NZ_CP008957.1 | 88312 | 89018 | 707 | 88721 | 1023 | 34.46704 | 1.5074 | 33.16003 | IP_LBN_vs_In_LBN_peak_4 | EDL933_RS00420:Promoter;EDL933_RS00410:Promoter;EDL933_RS00415:Promoter;EDL933_RS00405:Promoter | EDL933_RS00415:NZ_CP008957.1:88973-89918:+:-308 | EDL933_RS00415(leuO) |
| NZ_CP008957.1 | 5269773 | 5272003 | 2231 | 5271729 | 1122 | 37.27019 | 1.50386 | 35.94343 | IP_LBN_vs_In_LBN_peak_542 | EDL933_RS27185:Promoter;EDL933_RS27180:Promoter;EDL933_RS27175:Promoter | EDL933_RS27180:NZ_CP008957.1:5270207-5271644:-:756 | EDL933_RS27180(aspA) |
| NZ_CP008957.1 | 3616195 | 3617924 | 1730 | 3616533 | 946 | 31.60524 | 1.50332 | 30.31849 | IP_LBN_vs_In_LBN_peak_334 | EDL933_RS18880:Promoter;EDL933_RS18875:Promoter | EDL933_RS18875:NZ_CP008957.1:3616587-3617790:+:472 | EDL933_RS18875(proV) |
| NZ_CP008957.1 | 2674477 | 2674825 | 349 | 2674635 | 900 | 30.09019 | 1.50277 | 28.81443 | IP_LBN_vs_In_LBN_peak_229 | EDL933_RS14020:Promoter;EDL933_RS14010:Promoter;EDL933_RS14030:Promoter;EDL933_RS14015:Promoter;EDL933_RS14025:Promoter | EDL933_RS14025:NZ_CP008957.1:2674236-2674587:-:-63 | EDL933_RS14025(flhD) |
| NZ_CP008957.1 | 3607395 | 3609069 | 1675 | 3608436 | 937 | 31.1896 | 1.50192 | 29.90571 | IP_LBN_vs_In_LBN_peak_333 | EDL933_RS18830:Promoter;EDL933_RS18840:Promoter;EDL933_RS18810:Promoter;EDL933_RS18805:Promoter;EDL933_RS18825:Promoter | EDL933_RS18825:NZ_CP008957.1:3607898-3608303:-:71 | EDL933_RS18825(stpA) |
| NZ_CP008957.1 | 5007154 | 5008127 | 974 | 5007840 | 1241 | 40.19145 | 1.49664 | 38.84392 | IP_LBN_vs_In_LBN_peak_508 | EDL933_RS25985:CDS;EDL933_RS32875:Promoter | EDL933_RS25985:NZ_CP008957.1:5007228-5007903:-:263 | EDL933_RS25985(EDL933_RS25985) |
| NZ_CP008957.1 | 5143771 | 5144690 | 920 | 5144471 | 1153 | 36.96826 | 1.49236 | 35.64375 | IP_LBN_vs_In_LBN_peak_524 | EDL933_RS26600:Promoter | EDL933_RS26600:NZ_CP008957.1:5142919-5144395:-:165 | EDL933_RS26600(xylE) |
| NZ_CP008957.1 | 4116157 | 4116697 | 541 | 4116392 | 1107 | 35.46221 | 1.49152 | 34.14837 | IP_LBN_vs_In_LBN_peak_386 | EDL933_RS21440:Promoter;EDL933_RS21435:Promoter;EDL933_RS21445:Promoter | EDL933_RS21435:NZ_CP008957.1:4116592-4117177:+:-165 | EDL933_RS21435(EDL933_RS21435) |
| NZ_CP008957.1 | 2621375 | 2621697 | 323 | 2621528 | 893 | 28.85036 | 1.49109 | 27.58388 | IP_LBN_vs_In_LBN_peak_227 | EDL933_RS13765:CDS;EDL933_RS13775:Promoter;EDL933_RS13755:Promoter;EDL933_RS13760:Promoter;EDL933_RS13750:Promoter;EDL933_RS13770:Promoter | EDL933_RS13765:NZ_CP008957.1:2621400-2621631:+:135 | EDL933_RS13765(holE) |
| NZ_CP008957.1 | 4623576 | 4624044 | 469 | 4623855 | 1295 | 40.68492 | 1.48715 | 39.33354 | IP_LBN_vs_In_LBN_peak_452 | EDL933_RS23990:CDS | EDL933_RS23990:NZ_CP008957.1:4623521-4624538:-:728 | EDL933_RS23990(waaH) |
| NZ_CP008957.1 | 4017105 | 4017533 | 429 | 4017359 | 999 | 31.68001 | 1.48671 | 30.39285 | IP_LBN_vs_In_LBN_peak_373 | EDL933_RS20950:Promoter;EDL933_RS20930:Promoter;EDL933_RS34015:Promoter | EDL933_RS34015:NZ_CP008957.1:4017101-4017272:-:-46 | EDL933_RS34015(EDL933_RS34015) |
| NZ_CP008957.1 | 2566398 | 2567019 | 622 | 2566700 | 890 | 28.32622 | 1.48609 | 27.06365 | IP_LBN_vs_In_LBN_peak_224 | EDL933_RS13450:CDS;EDL933_RS13455:Promoter | EDL933_RS13450:NZ_CP008957.1:2566451-2567927:+:257 | EDL933_RS13450(EDL933_RS13450) |
| NZ_CP008957.1 | 3157561 | 3159177 | 1617 | 3158006 | 907 | 28.83414 | 1.48602 | 27.56808 | IP_LBN_vs_In_LBN_peak_278 | EDL933_RS16640:CDS;EDL933_RS16650:Promoter | EDL933_RS16640:NZ_CP008957.1:3154518-3158271:-:-97 | EDL933_RS16640(yfaL) |
| NZ_CP008957.1 | 2498302 | 2499184 | 883 | 2499045 | 889 | 28.15247 | 1.48442 | 26.89128 | IP_LBN_vs_In_LBN_peak_221 | EDL933_RS13095:Promoter;EDL933_RS13105:Promoter;EDL933_RS13090:CDS | EDL933_RS13095:NZ_CP008957.1:2499339-2499447:+:-596 | EDL933_RS13095(EDL933_RS13095) |
| NZ_CP008957.1 | 927719 | 928347 | 629 | 927953 | 888 | 27.9792 | 1.48275 | 26.71935 | IP_LBN_vs_In_LBN_peak_115 | EDL933_RS33085:Promoter;EDL933_RS04440:Promoter;EDL933_RS04430:CDS | EDL933_RS04440:NZ_CP008957.1:928364-929063:+:-331 | EDL933_RS04440(nleD) |
| NZ_CP008957.1 | 5240385 | 5241599 | 1215 | 5240668 | 1132 | 34.96647 | 1.47996 | 33.65586 | IP_LBN_vs_In_LBN_peak_536 | EDL933_RS27045:CDS;EDL933_RS27035:Promoter;EDL933_RS27040:Promoter | EDL933_RS27045:NZ_CP008957.1:5240482-5241244:-:252 | EDL933_RS27045(adiY) |
| NZ_CP008957.1 | 2283620 | 2285087 | 1468 | 2284723 | 884 | 27.29098 | 1.47608 | 26.03643 | IP_LBN_vs_In_LBN_peak_213 | EDL933_RS11955:Promoter;EDL933_RS11960:Promoter;EDL933_RS11950:Promoter | EDL933_RS11950:NZ_CP008957.1:2284127-2284718:-:365 | EDL933_RS11950(espM1) |
| NZ_CP008957.1 | 655919 | 656310 | 392 | 656121 | 883 | 27.12014 | 1.47441 | 25.86693 | IP_LBN_vs_In_LBN_peak_86 | EDL933_RS03045:Promoter;EDL933_RS03040:CDS | EDL933_RS03040:NZ_CP008957.1:655997-656630:-:516 | EDL933_RS03040(fimZ) |
| NZ_CP008957.1 | 3577391 | 3578057 | 667 | 3577750 | 880 | 26.61053 | 1.46941 | 25.3613 | IP_LBN_vs_In_LBN_peak_329 | EDL933_RS18670:Promoter;EDL933_RS18685:CDS;EDL933_RS18680:Promoter;EDL933_RS18695:Promoter;EDL933_RS18690:Promoter;EDL933_RS18675:Promoter | EDL933_RS18685:NZ_CP008957.1:3577292-3577859:-:135 | EDL933_RS18685(EDL933_RS18685) |
| NZ_CP008957.1 | 3840836 | 3841814 | 979 | 3841612 | 941 | 28.29311 | 1.4687 | 27.0311 | IP_LBN_vs_In_LBN_peak_359 | EDL933_RS20010:Promoter;EDL933_RS20025:Promoter;EDL933_RS20015:Promoter | EDL933_RS20020:NZ_CP008957.1:3840936-3841515:+:388 | EDL933_RS20020(mocA) |
| NZ_CP008957.1 | 3498356 | 3498700 | 345 | 3498490 | 877 | 26.10532 | 1.46441 | 24.85997 | IP_LBN_vs_In_LBN_peak_312 | EDL933_RS18240:Promoter;EDL933_RS18235:Promoter | EDL933_RS18240:NZ_CP008957.1:3496927-3498355:-:-172 | EDL933_RS18240(qseE) |
| NZ_CP008957.1 | 4582855 | 4583654 | 800 | 4583361 | 1256 | 36.63276 | 1.46327 | 35.31069 | IP_LBN_vs_In_LBN_peak_447 | EDL933_RS23825:Promoter;EDL933_RS23830:CDS | EDL933_RS23830:NZ_CP008957.1:4583061-4583385:-:131 | EDL933_RS23830(EDL933_RS23830) |
| NZ_CP008957.1 | 1329794 | 1331159 | 1366 | 1330272 | 1458 | 42.20731 | 1.4627 | 40.84538 | IP_LBN_vs_In_LBN_peak_161 | EDL933_RS06580:Promoter;EDL933_RS06590:CDS;EDL933_RS06595:Promoter;EDL933_RS06585:Promoter | EDL933_RS06590:NZ_CP008957.1:1329861-1331119:+:615 | EDL933_RS06590(EDL933_RS06590) |
| NZ_CP008957.1 | 4927469 | 4928362 | 894 | 4927651 | 1244 | 36.05843 | 1.46125 | 34.7401 | IP_LBN_vs_In_LBN_peak_495 | EDL933_RS25580:Promoter | EDL933_RS25580:NZ_CP008957.1:4927776-4929207:+:139 | EDL933_RS25580(EDL933_RS25580) |
| NZ_CP008957.1 | 4718457 | 4718934 | 478 | 4718724 | 1289 | 37.27853 | 1.46099 | 35.95173 | IP_LBN_vs_In_LBN_peak_466 | EDL933_RS24535:CDS;EDL933_RS24530:Promoter;EDL933_RS24525:Promoter | EDL933_RS24535:NZ_CP008957.1:4718719-4720486:+:-24 | EDL933_RS24535(adeD) |
| NZ_CP008957.1 | 5131148 | 5131560 | 413 | 5131342 | 1152 | 33.18223 | 1.45834 | 31.88415 | IP_LBN_vs_In_LBN_peak_522 | EDL933_RS26530:Promoter;EDL933_RS26520:Promoter;EDL933_RS26540:Promoter;EDL933_RS26535:CDS;EDL933_RS26515:Promoter;EDL933_RS26525:Promoter | EDL933_RS26530:NZ_CP008957.1:5130441-5131248:-:-105 | EDL933_RS26530(EDL933_RS26530) |
| NZ_CP008957.1 | 4800603 | 4803989 | 3387 | 4803280 | 1260 | 35.90559 | 1.45623 | 34.58839 | IP_LBN_vs_In_LBN_peak_481 | EDL933_RS24955:Promoter;EDL933_RS24950:Promoter;EDL933_RS24940:Promoter;EDL933_RS24960:CDS;EDL933_RS24945:Promoter | EDL933_RS24950:NZ_CP008957.1:4802017-4802257:-:-38 | EDL933_RS24950(atpE) |
| NZ_CP008957.1 | 4293323 | 4293726 | 404 | 4293511 | 1086 | 30.88182 | 1.45364 | 29.60037 | IP_LBN_vs_In_LBN_peak_412 | EDL933_RS22460:Promoter;EDL933_RS22465:Promoter;EDL933_RS22450:Promoter | EDL933_RS22460:NZ_CP008957.1:4293652-4294285:+:-128 | EDL933_RS22460(crp) |
| NZ_CP008957.1 | 4504192 | 4504469 | 278 | 4504325 | 1181 | 33.36901 | 1.45285 | 32.06964 | IP_LBN_vs_In_LBN_peak_436 | EDL933_RS23480:Promoter | EDL933_RS23480:NZ_CP008957.1:4504547-4505870:+:-217 | EDL933_RS23480(EDL933_RS23480) |
| NZ_CP008957.1 | 841633 | 842401 | 769 | 841790 | 869 | 24.77967 | 1.45106 | 23.54472 | IP_LBN_vs_In_LBN_peak_106 | EDL933_RS03905:Promoter | EDL933_RS03905:NZ_CP008957.1:841956-842862:+:60 | EDL933_RS03905(EDL933_RS03905) |
| NZ_CP008957.1 | 161255 | 161683 | 429 | 161425 | 958 | 26.9565 | 1.44864 | 25.70457 | IP_LBN_vs_In_LBN_peak_11 | EDL933_RS00735:Promoter;EDL933_RS00740:Promoter;EDL933_RS00730:Promoter | EDL933_RS00740:NZ_CP008957.1:160695-161292:-:-176 | EDL933_RS00740(EDL933_RS00740) |
| NZ_CP008957.1 | 4831211 | 4831613 | 403 | 4831349 | 1257 | 34.74902 | 1.44714 | 33.43987 | IP_LBN_vs_In_LBN_peak_486 | EDL933_RS25095:Promoter;EDL933_RS25090:Promoter;EDL933_RS25085:Promoter;EDL933_RS25100:Promoter;EDL933_RS33875:Promoter | EDL933_RS25090:NZ_CP008957.1:4831390-4831489:+:21 | EDL933_RS25090(ilvL) |
| NZ_CP008957.1 | 979771 | 980143 | 373 | 980010 | 866 | 24.29068 | 1.44606 | 23.05949 | IP_LBN_vs_In_LBN_peak_123 | EDL933_RS04700:Promoter;EDL933_RS04695:Promoter | EDL933_RS04700:NZ_CP008957.1:980038-981631:+:-81 | EDL933_RS04700(EDL933_RS04700) |
| NZ_CP008957.1 | 3219807 | 3220666 | 860 | 3220175 | 865 | 24.12867 | 1.44439 | 22.89882 | IP_LBN_vs_In_LBN_peak_284 | EDL933_RS16930:Promoter;EDL933_RS16925:Promoter;EDL933_RS16915:Promoter;EDL933_RS16920:Promoter | EDL933_RS16920:NZ_CP008957.1:3218878-3219817:-:-419 | EDL933_RS16920(lrhA) |
| NZ_CP008957.1 | 2859529 | 2860355 | 827 | 2860091 | 865 | 24.12867 | 1.44439 | 22.89882 | IP_LBN_vs_In_LBN_peak_245 | EDL933_RS15165:Promoter;EDL933_RS15160:Promoter | EDL933_RS15165:NZ_CP008957.1:2858817-2859984:-:42 | EDL933_RS15165(ugd) |
| NZ_CP008957.1 | 883838 | 884483 | 646 | 884131 | 864 | 23.96717 | 1.44272 | 22.73859 | IP_LBN_vs_In_LBN_peak_109 | EDL933_RS04165:Promoter;EDL933_RS04155:CDS;EDL933_RS04160:Promoter | EDL933_RS04155:NZ_CP008957.1:883265-884219:-:59 | EDL933_RS04155(EDL933_RS04155) |
| NZ_CP008957.1 | 1378619 | 1379276 | 658 | 1379033 | 3374 | 89.052 | 1.44193 | 87.35966 | IP_LBN_vs_In_LBN_peak_175 | EDL933_RS06980:Promoter;EDL933_RS06985:Promoter;EDL933_RS06995:Promoter;EDL933_RS06990:Promoter;EDL933_RS07000:Promoter | EDL933_RS06980:NZ_CP008957.1:1379142-1379283:+:-195 | EDL933_RS06980(EDL933_RS06980) |
| NZ_CP008957.1 | 4141210 | 4142048 | 839 | 4141848 | 1063 | 28.96393 | 1.44067 | 27.69677 | IP_LBN_vs_In_LBN_peak_389 | EDL933_RS21570:Promoter;EDL933_RS21565:Promoter | EDL933_RS21570:NZ_CP008957.1:4141538-4141808:-:179 | EDL933_RS21570(rpsO) |
| NZ_CP008957.1 | 4089542 | 4090893 | 1352 | 4089729 | 1008 | 27.42308 | 1.43945 | 26.16783 | IP_LBN_vs_In_LBN_peak_382 | EDL933_RS21290:Promoter;EDL933_RS21295:CDS | EDL933_RS21290:NZ_CP008957.1:4087379-4089674:-:-543 | EDL933_RS21290(tdcE) |
| NZ_CP008957.1 | 4117360 | 4117854 | 495 | 4117572 | 1037 | 28.15154 | 1.43924 | 26.89075 | IP_LBN_vs_In_LBN_peak_387 | EDL933_RS21440:CDS;EDL933_RS21445:Promoter | EDL933_RS21440:NZ_CP008957.1:4117256-4117952:+:350 | EDL933_RS21440(EDL933_RS21440) |
| NZ_CP008957.1 | 3470470 | 3471063 | 594 | 3470856 | 873 | 23.81735 | 1.43801 | 22.59003 | IP_LBN_vs_In_LBN_peak_310 | EDL933_RS18135:Promoter;EDL933_RS18130:Promoter;EDL933_RS18110:Promoter;EDL933_RS18120:Promoter;EDL933_RS18115:Promoter;EDL933_RS18125:CDS | EDL933_RS18120:NZ_CP008957.1:3469977-3470715:-:-51 | EDL933_RS18120(trmJ) |
| NZ_CP008957.1 | 3912062 | 3913065 | 1004 | 3912676 | 975 | 26.26043 | 1.43604 | 25.01423 | IP_LBN_vs_In_LBN_peak_364 | EDL933_RS20345:CDS;EDL933_RS20340:Promoter | EDL933_RS20345:NZ_CP008957.1:3912169-3912901:-:338 | EDL933_RS20345(EDL933_RS20345) |
| NZ_CP008957.1 | 3515558 | 3517399 | 1842 | 3516439 | 861 | 22.91608 | 1.43061 | 21.69633 | IP_LBN_vs_In_LBN_peak_315 | EDL933_RS18345:Promoter;EDL933_RS18325:Promoter;EDL933_RS18320:Promoter;EDL933_RS18330:CDS | EDL933_RS18330:NZ_CP008957.1:3516029-3516668:-:190 | EDL933_RS18330(rseA) |
| NZ_CP008957.1 | 1455541 | 1456644 | 1104 | 1455751 | 855 | 22.53599 | 1.42771 | 21.31927 | IP_LBN_vs_In_LBN_peak_189 | EDL933_RS07295:CDS;EDL933_RS07300:Promoter | EDL933_RS07295:NZ_CP008957.1:1455703-1456690:+:389 | EDL933_RS07295(EDL933_RS07295) |
| NZ_CP008957.1 | 638542 | 638993 | 452 | 638784 | 872 | 22.91612 | 1.4272 | 21.69637 | IP_LBN_vs_In_LBN_peak_82 | EDL933_RS02945:Promoter;EDL933_RS02950:Promoter;EDL933_RS02960:Promoter;EDL933_RS02955:Promoter | EDL933_RS02955:NZ_CP008957.1:638878-640546:+:-111 | EDL933_RS02955(EDL933_RS02955) |
| NZ_CP008957.1 | 302369 | 302856 | 488 | 302609 | 938 | 24.52796 | 1.42697 | 23.29507 | IP_LBN_vs_In_LBN_peak_25 | EDL933_RS01435:CDS;EDL933_RS01420:Promoter;EDL933_RS01430:Promoter;EDL933_RS01450:Promoter;EDL933_RS01425:Promoter;EDL933_RS01445:Promoter;EDL933_RS01455:Promoter;EDL933_RS01440:Promoter | EDL933_RS01435:NZ_CP008957.1:301939-302653:-:41 | EDL933_RS01435(EDL933_RS01435) |
| NZ_CP008957.1 | 349506 | 350129 | 624 | 349841 | 949 | 24.69674 | 1.4258 | 23.46264 | IP_LBN_vs_In_LBN_peak_34 | EDL933_RS01710:Promoter;EDL933_RS01715:Promoter;EDL933_RS01725:Promoter | EDL933_RS01715:NZ_CP008957.1:348573-349716:-:-101 | EDL933_RS01715(EDL933_RS01715) |
| NZ_CP008957.1 | 5510151 | 5510996 | 846 | 5510400 | 1073 | 27.3649 | 1.42196 | 26.10993 | IP_LBN_vs_In_LBN_peak_584 | EDL933_RS28305:Promoter;EDL933_RS28285:Promoter;EDL933_RS28300:Promoter;EDL933_RS28290:Promoter;EDL933_RS28295:Promoter | EDL933_RS28300:NZ_CP008957.1:5510708-5511434:+:-135 | EDL933_RS28300(EDL933_RS28300) |
| NZ_CP008957.1 | 386256 | 387033 | 778 | 386795 | 890 | 22.76473 | 1.41993 | 21.54623 | IP_LBN_vs_In_LBN_peak_46 | EDL933_RS01880:Promoter;EDL933_RS01865:Promoter;EDL933_RS01885:Promoter;EDL933_RS01870:Promoter;EDL933_RS01875:CDS | EDL933_RS01870:NZ_CP008957.1:386011-386519:-:-125 | EDL933_RS01870(EDL933_RS01870) |
| NZ_CP008957.1 | 1549838 | 1551219 | 1382 | 1550758 | 848 | 21.45091 | 1.41604 | 20.24326 | IP_LBN_vs_In_LBN_peak_191 | EDL933_RS07835:Promoter;EDL933_RS07840:Promoter;EDL933_RS07845:Promoter;EDL933_RS07825:Promoter;EDL933_RS07855:Promoter;EDL933_RS07850:Promoter;EDL933_RS07815:Promoter;EDL933_RS07820:Promoter | EDL933_RS07835:NZ_CP008957.1:1550824-1551280:+:-296 | EDL933_RS07835(csgB) |
| NZ_CP008957.1 | 5312667 | 5313251 | 585 | 5312848 | 1084 | 27.02088 | 1.41581 | 25.76865 | IP_LBN_vs_In_LBN_peak_551 | EDL933_RS27405:Promoter;EDL933_RS27390:CDS;EDL933_RS27400:Promoter;EDL933_RS27395:Promoter | EDL933_RS27395:NZ_CP008957.1:5313145-5313844:+:-186 | EDL933_RS27395(EDL933_RS27395) |
| NZ_CP008957.1 | 486497 | 487743 | 1247 | 487093 | 877 | 22.08322 | 1.41535 | 20.87047 | IP_LBN_vs_In_LBN_peak_59 | EDL933_RS02335:CDS;EDL933_RS02345:Promoter | EDL933_RS02335:NZ_CP008957.1:486500-487946:+:619 | EDL933_RS02335(EDL933_RS02335) |
| NZ_CP008957.1 | 5276890 | 5277797 | 908 | 5277593 | 1068 | 26.53558 | 1.41471 | 25.28717 | IP_LBN_vs_In_LBN_peak_543 | EDL933_RS27225:Promoter;EDL933_RS27210:Promoter;EDL933_RS27220:Promoter | EDL933_RS27210:NZ_CP008957.1:5276671-5277541:-:198 | EDL933_RS27210(EDL933_RS27210) |
| NZ_CP008957.1 | 3712776 | 3713734 | 959 | 3712986 | 999 | 24.74802 | 1.41294 | 23.51362 | IP_LBN_vs_In_LBN_peak_346 | EDL933_RS19400:Promoter;EDL933_RS19395:Promoter;EDL933_RS19390:Promoter;EDL933_RS19385:Promoter | EDL933_RS19390:NZ_CP008957.1:3713006-3713627:+:248 | EDL933_RS19390(EDL933_RS19390) |
| NZ_CP008957.1 | 192988 | 193878 | 891 | 193191 | 947 | 23.51359 | 1.41274 | 22.28879 | IP_LBN_vs_In_LBN_peak_12 | EDL933_RS00880:Promoter;EDL933_RS00865:Promoter;EDL933_RS00870:Promoter;EDL933_RS00875:Promoter | EDL933_RS00875:NZ_CP008957.1:193360-194086:+:72 | EDL933_RS00875(rpsB) |
| NZ_CP008957.1 | 3548683 | 3549415 | 733 | 3548872 | 846 | 21.14543 | 1.4127 | 19.94029 | IP_LBN_vs_In_LBN_peak_322 | EDL933_RS18475:Promoter;EDL933_RS18500:Promoter;EDL933_RS18490:Promoter;EDL933_RS18480:Promoter;EDL933_RS18495:Promoter | EDL933_RS18490:NZ_CP008957.1:3548882-3549401:+:166 | EDL933_RS18490(EDL933_RS18490) |
| NZ_CP008957.1 | 4952524 | 4952942 | 419 | 4952730 | 1195 | 29.30653 | 1.41269 | 28.03678 | IP_LBN_vs_In_LBN_peak_500 | EDL933_RS25675:Promoter | EDL933_RS25675:NZ_CP008957.1:4950663-4952700:-:-32 | EDL933_RS25675(yihQ) |
| NZ_CP008957.1 | 2603721 | 2604521 | 801 | 2603889 | 844 | 20.84198 | 1.40937 | 19.63948 | IP_LBN_vs_In_LBN_peak_226 | EDL933_RS13680:Promoter;EDL933_RS13660:Promoter;EDL933_RS13650:Promoter;EDL933_RS13655:Promoter | EDL933_RS13660:NZ_CP008957.1:2603739-2603883:-:-237 | EDL933_RS13660(EDL933_RS13660) |
| NZ_CP008957.1 | 1946918 | 1947233 | 316 | 1947043 | 842 | 20.54055 | 1.40603 | 19.3406 | IP_LBN_vs_In_LBN_peak_204 | EDL933_RS10275:Promoter;EDL933_RS10250:Promoter | EDL933_RS10265:NZ_CP008957.1:1947149-1947245:-:170 | EDL933_RS10265(mgtS) |
| NZ_CP008957.1 | 1025025 | 1025526 | 502 | 1025290 | 842 | 20.54055 | 1.40603 | 19.3406 | IP_LBN_vs_In_LBN_peak_127 | EDL933_RS04920:CDS;EDL933_RS04930:Promoter;EDL933_RS04925:Promoter | EDL933_RS04920:NZ_CP008957.1:1024982-1026326:+:293 | EDL933_RS04920(EDL933_RS04920) |
| NZ_CP008957.1 | 4945477 | 4945939 | 463 | 4945760 | 1207 | 28.68595 | 1.40445 | 27.42068 | IP_LBN_vs_In_LBN_peak_498 | EDL933_RS25655:CDS | EDL933_RS25655:NZ_CP008957.1:4945671-4946937:+:36 | EDL933_RS25655(EDL933_RS25655) |
| NZ_CP008957.1 | 2820495 | 2820910 | 416 | 2820706 | 841 | 20.3906 | 1.40436 | 19.1919 | IP_LBN_vs_In_LBN_peak_240 | EDL933_RS14940:Promoter | EDL933_RS14940:NZ_CP008957.1:2820793-2821510:+:-91 | EDL933_RS14940(EDL933_RS14940) |
| NZ_CP008957.1 | 363776 | 364321 | 546 | 364069 | 904 | 21.79992 | 1.40413 | 20.58953 | IP_LBN_vs_In_LBN_peak_39 | EDL933_RS01765:Promoter;EDL933_RS01775:Promoter;EDL933_RS01780:Promoter | EDL933_RS01775:NZ_CP008957.1:364322-365042:+:-274 | EDL933_RS01775(EDL933_RS01775) |
| NZ_CP008957.1 | 4996194 | 4996435 | 242 | 4996295 | 1155 | 27.3522 | 1.40293 | 26.09731 | IP_LBN_vs_In_LBN_peak_507 | EDL933_RS25920:Promoter;EDL933_RS25925:Promoter | EDL933_RS25920:NZ_CP008957.1:4996418-4997408:+:-104 | EDL933_RS25920(sbp) |
| NZ_CP008957.1 | 3974147 | 3974652 | 506 | 3974310 | 953 | 22.7946 | 1.4028 | 21.57593 | IP_LBN_vs_In_LBN_peak_372 | EDL933_RS20705:Promoter;EDL933_RS20725:Promoter;EDL933_RS20700:Promoter | EDL933_RS20705:NZ_CP008957.1:3973122-3974241:-:-158 | EDL933_RS20705(hybO) |
| NZ_CP008957.1 | 2893264 | 2894035 | 772 | 2893803 | 840 | 20.24116 | 1.4027 | 19.0438 | IP_LBN_vs_In_LBN_peak_250 | EDL933_RS15305:Promoter;EDL933_RS15315:CDS;EDL933_RS15300:Promoter;EDL933_RS15310:Promoter | EDL933_RS15310:NZ_CP008957.1:2892586-2893333:-:-316 | EDL933_RS15310(wcaE) |
| NZ_CP008957.1 | 755808 | 756202 | 395 | 755979 | 839 | 20.09223 | 1.40103 | 18.89617 | IP_LBN_vs_In_LBN_peak_98 | EDL933_RS03505:Promoter;EDL933_RS03490:Promoter;EDL933_RS03495:Promoter | EDL933_RS03490:NZ_CP008957.1:753335-755918:-:-86 | EDL933_RS03490(leuS) |
| NZ_CP008957.1 | 579536 | 580037 | 502 | 579736 | 839 | 20.09223 | 1.40103 | 18.89617 | IP_LBN_vs_In_LBN_peak_72 | EDL933_RS02780:Promoter;EDL933_RS02775:Promoter | EDL933_RS02775:NZ_CP008957.1:579894-581250:+:-108 | EDL933_RS02775(EDL933_RS02775) |
| NZ_CP008957.1 | 5438971 | 5439582 | 612 | 5439309 | 1091 | 25.69387 | 1.4007 | 24.45197 | IP_LBN_vs_In_LBN_peak_568 | intergenic | EDL933_RS32920:NZ_CP008957.1:5439587-5440283:-:1007 | EDL933_RS32920(EDL933_RS32920) |
| NZ_CP008957.1 | 2506803 | 2507404 | 602 | 2507016 | 838 | 19.9438 | 1.39936 | 18.74903 | IP_LBN_vs_In_LBN_peak_222 | EDL933_RS33525:CDS;EDL933_RS13150:Promoter | EDL933_RS33525:NZ_CP008957.1:2506550-2507306:-:203 | EDL933_RS33525(EDL933_RS33525) |
| NZ_CP008957.1 | 742291 | 742994 | 704 | 742786 | 838 | 19.9438 | 1.39936 | 18.74903 | IP_LBN_vs_In_LBN_peak_97 | EDL933_RS03420:Promoter;EDL933_RS03415:Promoter;EDL933_RS03430:CDS | EDL933_RS03420:NZ_CP008957.1:741557-742511:-:-131 | EDL933_RS03420(EDL933_RS03420) |
| NZ_CP008957.1 | 581039 | 581389 | 351 | 581228 | 885 | 20.98275 | 1.39928 | 19.77945 | IP_LBN_vs_In_LBN_peak_73 | EDL933_RS02780:Promoter;EDL933_RS02775:CDS | EDL933_RS02780:NZ_CP008957.1:581352-585738:+:-138 | EDL933_RS02780(EDL933_RS02780) |
| NZ_CP008957.1 | 2971727 | 2972017 | 291 | 2971853 | 837 | 19.79589 | 1.39769 | 18.60244 | IP_LBN_vs_In_LBN_peak_259 | EDL933_RS15660:Promoter;EDL933_RS15655:Promoter | EDL933_RS15655:NZ_CP008957.1:2972588-2973677:+:-716 | EDL933_RS15655(EDL933_RS15655) |
| NZ_CP008957.1 | 1141441 | 1141789 | 349 | 1141533 | 837 | 19.79589 | 1.39769 | 18.60244 | IP_LBN_vs_In_LBN_peak_133 | EDL933_RS05635:Promoter;EDL933_RS05625:Promoter;EDL933_RS05620:Promoter;EDL933_RS05630:Promoter;EDL933_RS05610:CDS | EDL933_RS05610:NZ_CP008957.1:1141087-1142301:-:686 | EDL933_RS05610(EDL933_RS05610) |
| NZ_CP008957.1 | 3021849 | 3022577 | 729 | 3022016 | 991 | 23.16591 | 1.39737 | 21.94441 | IP_LBN_vs_In_LBN_peak_263 | EDL933_RS15985:Promoter;EDL933_RS15965:Promoter;EDL933_RS15960:Promoter;EDL933_RS15955:Promoter;EDL933_RS15980:Promoter;EDL933_RS15975:CDS | EDL933_RS15980:NZ_CP008957.1:3022126-3022678:+:86 | EDL933_RS15980(EDL933_RS15980) |
| NZ_CP008957.1 | 2160750 | 2161375 | 626 | 2161021 | 836 | 19.64849 | 1.39602 | 18.45634 | IP_LBN_vs_In_LBN_peak_211 | EDL933_RS11270:CDS | EDL933_RS11270:NZ_CP008957.1:2160254-2161406:-:344 | EDL933_RS11270(EDL933_RS11270) |
| NZ_CP008957.1 | 2158239 | 2160401 | 2163 | 2160208 | 836 | 19.64849 | 1.39602 | 18.45634 | IP_LBN_vs_In_LBN_peak_210 | EDL933_RS11265:CDS | EDL933_RS11265:NZ_CP008957.1:2159297-2160287:+:22 | EDL933_RS11265(EDL933_RS11265) |
| NZ_CP008957.1 | 3626422 | 3626784 | 363 | 3626585 | 845 | 19.79869 | 1.3954 | 18.60521 | IP_LBN_vs_In_LBN_peak_335 | EDL933_RS18920:Promoter | EDL933_RS18920:NZ_CP008957.1:3625996-3626512:-:-90 | EDL933_RS18920(luxS) |
| NZ_CP008957.1 | 423252 | 424288 | 1037 | 423527 | 25390 | 546.39203 | 1.39307 | 543.72797 | IP_LBN_vs_In_LBN_peak_50 | EDL933_RS02035:CDS;EDL933_RS02030:Promoter | EDL933_RS02035:NZ_CP008957.1:423203-424286:-:516 | EDL933_RS02035(lacI) |
| NZ_CP008957.1 | 4442552 | 4443244 | 693 | 4442950 | 1131 | 25.72446 | 1.39219 | 24.48237 | IP_LBN_vs_In_LBN_peak_425 | EDL933_RS23200:Promoter;EDL933_RS23195:Promoter | EDL933_RS23195:NZ_CP008957.1:4443015-4444374:+:-117 | EDL933_RS23195(EDL933_RS23195) |
| NZ_CP008957.1 | 2917065 | 2917622 | 558 | 2917454 | 832 | 19.06402 | 1.38935 | 17.87729 | IP_LBN_vs_In_LBN_peak_252 | EDL933_RS15405:Promoter;EDL933_RS30905:Promoter;EDL933_RS15420:Promoter;EDL933_RS15410:Promoter;EDL933_RS15415:Promoter;EDL933_RS33635:CDS;EDL933_RS33630:Promoter | EDL933_RS33635:NZ_CP008957.1:2917420-2917477:-:134 | EDL933_RS33635(EDL933_RS33635) |
| NZ_CP008957.1 | 4624294 | 4624783 | 490 | 4624492 | 1155 | 25.91142 | 1.38897 | 24.66797 | IP_LBN_vs_In_LBN_peak_453 | EDL933_RS23990:CDS | EDL933_RS23990:NZ_CP008957.1:4623521-4624538:-:0 | EDL933_RS23990(waaH) |
| NZ_CP008957.1 | 3293678 | 3293977 | 300 | 3293804 | 830 | 18.77487 | 1.38602 | 17.59091 | IP_LBN_vs_In_LBN_peak_292 | EDL933_RS17290:Promoter;EDL933_RS17285:CDS;EDL933_RS17295:Promoter | EDL933_RS17285:NZ_CP008957.1:3292869-3293928:-:101 | EDL933_RS17285(EDL933_RS17285) |
| NZ_CP008957.1 | 460778 | 461237 | 460 | 460996 | 856 | 19.27604 | 1.3854 | 18.08742 | IP_LBN_vs_In_LBN_peak_54 | EDL933_RS02220:Promoter;EDL933_RS02210:Promoter;EDL933_RS02225:Promoter;EDL933_RS02195:Promoter | EDL933_RS02220:NZ_CP008957.1:461164-461425:+:-157 | EDL933_RS02220(iraP) |
| NZ_CP008957.1 | 5278621 | 5278969 | 349 | 5278787 | 1040 | 23.11204 | 1.38513 | 21.89082 | IP_LBN_vs_In_LBN_peak_544 | EDL933_RS27225:Promoter;EDL933_RS27215:CDS;EDL933_RS27210:Promoter;EDL933_RS27230:Promoter;EDL933_RS27220:Promoter;EDL933_RS27235:Promoter | EDL933_RS27215:NZ_CP008957.1:5277775-5278804:-:9 | EDL933_RS27215(epmB) |
| NZ_CP008957.1 | 5137627 | 5138066 | 440 | 5137872 | 1101 | 24.2126 | 1.38332 | 22.98229 | IP_LBN_vs_In_LBN_peak_523 | EDL933_RS26560:Promoter;EDL933_RS26565:Promoter;EDL933_RS26570:Promoter;EDL933_RS26575:Promoter | EDL933_RS26560:NZ_CP008957.1:5138046-5138289:+:-200 | EDL933_RS26560(EDL933_RS26560) |
| NZ_CP008957.1 | 757570 | 758337 | 768 | 757856 | 828 | 18.48778 | 1.38268 | 17.30668 | IP_LBN_vs_In_LBN_peak_99 | EDL933_RS03505:CDS;EDL933_RS03490:Promoter;EDL933_RS03510:Promoter;EDL933_RS29545:Promoter | EDL933_RS03505:NZ_CP008957.1:757847-758555:+:106 | EDL933_RS03505(EDL933_RS03505) |
| NZ_CP008957.1 | 481208 | 481545 | 338 | 481327 | 838 | 18.61722 | 1.38162 | 17.43523 | IP_LBN_vs_In_LBN_peak_58 | EDL933_RS02325:Promoter;EDL933_RS02320:Promoter | EDL933_RS02320:NZ_CP008957.1:481571-482891:+:-195 | EDL933_RS02320(brnQ) |
| NZ_CP008957.1 | 732744 | 733492 | 749 | 733359 | 827 | 18.34501 | 1.38101 | 17.16535 | IP_LBN_vs_In_LBN_peak_94 | EDL933_RS03380:Promoter;EDL933_RS03370:Promoter;EDL933_RS03360:Promoter;EDL933_RS03375:Promoter;EDL933_RS03365:Promoter | EDL933_RS03370:NZ_CP008957.1:731929-732988:-:-129 | EDL933_RS03370(citC) |
| NZ_CP008957.1 | 3020746 | 3021477 | 732 | 3021250 | 1052 | 22.82774 | 1.37936 | 21.6089 | IP_LBN_vs_In_LBN_peak_262 | EDL933_RS15985:Promoter;EDL933_RS15955:Promoter;EDL933_RS15965:Promoter;EDL933_RS15970:CDS;EDL933_RS15960:Promoter;EDL933_RS15980:Promoter;EDL933_RS15950:Promoter;EDL933_RS15975:Promoter | EDL933_RS15970:NZ_CP008957.1:3021068-3021716:+:43 | EDL933_RS15970(EDL933_RS15970) |
| NZ_CP008957.1 | 2699771 | 2700321 | 551 | 2700106 | 826 | 18.20275 | 1.37934 | 17.02452 | IP_LBN_vs_In_LBN_peak_230 | EDL933_RS14210:CDS;EDL933_RS14195:Promoter | EDL933_RS14210:NZ_CP008957.1:2699706-2700279:-:233 | EDL933_RS14210(EDL933_RS14210) |
| NZ_CP008957.1 | 1567853 | 1568252 | 400 | 1568041 | 825 | 18.06102 | 1.37768 | 16.88423 | IP_LBN_vs_In_LBN_peak_193 | EDL933_RS07925:Promoter;EDL933_RS07935:Promoter;EDL933_RS07930:Promoter | EDL933_RS07935:NZ_CP008957.1:1567576-1567831:-:-221 | EDL933_RS07935(bssS) |
| NZ_CP008957.1 | 5314419 | 5314941 | 523 | 5314747 | 1057 | 22.75232 | 1.37744 | 21.53389 | IP_LBN_vs_In_LBN_peak_552 | EDL933_RS27410:Promoter;EDL933_RS27405:CDS;EDL933_RS27415:Promoter | EDL933_RS27405:NZ_CP008957.1:5314571-5314970:+:108 | EDL933_RS27405(EDL933_RS27405) |
| NZ_CP008957.1 | 3023844 | 3024088 | 245 | 3023989 | 848 | 18.49956 | 1.37728 | 17.31838 | IP_LBN_vs_In_LBN_peak_264 | EDL933_RS15990:CDS;EDL933_RS16010:Promoter;EDL933_RS33690:Promoter;EDL933_RS33685:Promoter;EDL933_RS15995:Promoter;EDL933_RS16015:Promoter | EDL933_RS15990:NZ_CP008957.1:3023479-3024061:-:95 | EDL933_RS15990(EDL933_RS15990) |
| NZ_CP008957.1 | 4767744 | 4768008 | 265 | 4767854 | 1196 | 25.3261 | 1.37513 | 24.08715 | IP_LBN_vs_In_LBN_peak_472 | EDL933_RS24790:Promoter;EDL933_RS24795:Promoter | EDL933_RS24790:NZ_CP008957.1:4767947-4769123:+:-71 | EDL933_RS24790(mdtL) |
| NZ_CP008957.1 | 1228767 | 1229119 | 353 | 1228927 | 861 | 18.34099 | 1.37169 | 17.16181 | IP_LBN_vs_In_LBN_peak_147 | EDL933_RS05975:Promoter;EDL933_RS05970:CDS | EDL933_RS05970:NZ_CP008957.1:1228803-1230057:+:139 | EDL933_RS05970(pqiA) |
| NZ_CP008957.1 | 520674 | 521354 | 681 | 520978 | 821 | 17.49926 | 1.37101 | 16.32818 | IP_LBN_vs_In_LBN_peak_63 | EDL933_RS02530:Promoter;EDL933_RS02525:Promoter;EDL933_RS02505:Promoter;EDL933_RS02510:Promoter | EDL933_RS02525:NZ_CP008957.1:521185-522484:+:-171 | EDL933_RS02525(tig) |
| NZ_CP008957.1 | 3056918 | 3057417 | 500 | 3057117 | 859 | 18.16845 | 1.36991 | 16.99086 | IP_LBN_vs_In_LBN_peak_267 | EDL933_RS16205:CDS;EDL933_RS16215:Promoter;EDL933_RS16210:Promoter | EDL933_RS16210:NZ_CP008957.1:3057345-3058584:+:-178 | EDL933_RS16210(preT) |
| NZ_CP008957.1 | 3906930 | 3907931 | 1002 | 3907724 | 975 | 20.4229 | 1.36962 | 19.22403 | IP_LBN_vs_In_LBN_peak_363 | EDL933_RS20325:Promoter;EDL933_RS20320:Promoter | EDL933_RS20325:NZ_CP008957.1:3907123-3907567:-:137 | EDL933_RS20325(cmtB) |
| NZ_CP008957.1 | 3183112 | 3183486 | 375 | 3183274 | 820 | 17.36012 | 1.36934 | 16.19042 | IP_LBN_vs_In_LBN_peak_281 | EDL933_RS16750:Promoter;EDL933_RS16755:Promoter;EDL933_RS16740:Promoter;EDL933_RS16760:Promoter | EDL933_RS16755:NZ_CP008957.1:3183446-3184586:+:-147 | EDL933_RS16755(arnB) |
| NZ_CP008957.1 | 4497202 | 4498085 | 884 | 4497899 | 1098 | 22.67653 | 1.36797 | 21.45898 | IP_LBN_vs_In_LBN_peak_433 | EDL933_RS23460:Promoter;EDL933_RS23455:CDS;EDL933_RS23450:Promoter | EDL933_RS23450:NZ_CP008957.1:4495939-4497340:-:-303 | EDL933_RS23450(EDL933_RS23450) |
| NZ_CP008957.1 | 5497884 | 5498748 | 865 | 5498069 | 974 | 20.19692 | 1.36718 | 19.00021 | IP_LBN_vs_In_LBN_peak_582 | EDL933_RS28250:Promoter;EDL933_RS28235:Promoter | EDL933_RS28245:NZ_CP008957.1:5498107-5498650:-:334 | EDL933_RS28245(EDL933_RS28245) |
| NZ_CP008957.1 | 1415607 | 1416375 | 769 | 1415807 | 852 | 17.75575 | 1.3662 | 16.58241 | IP_LBN_vs_In_LBN_peak_181 | EDL933_RS07140:CDS | EDL933_RS07140:NZ_CP008957.1:1415483-1415897:-:-93 | EDL933_RS07140(pgaD) |
| NZ_CP008957.1 | 496371 | 496720 | 350 | 496540 | 869 | 18.05865 | 1.36586 | 16.88227 | IP_LBN_vs_In_LBN_peak_60 | EDL933_RS02405:Promoter;EDL933_RS02400:Promoter;EDL933_RS02390:Promoter | EDL933_RS02390:NZ_CP008957.1:495383-496268:-:-277 | EDL933_RS02390(tsx) |
| NZ_CP008957.1 | 5487757 | 5488196 | 440 | 5487961 | 976 | 20.12285 | 1.36584 | 18.92659 | IP_LBN_vs_In_LBN_peak_580 | EDL933_RS28200:CDS;EDL933_RS28195:Promoter | EDL933_RS28200:NZ_CP008957.1:5486724-5488479:-:503 | EDL933_RS28200(EDL933_RS28200) |
| NZ_CP008957.1 | 347903 | 348255 | 353 | 348110 | 900 | 18.23952 | 1.36048 | 17.06105 | IP_LBN_vs_In_LBN_peak_33 | EDL933_RS01710:Promoter | EDL933_RS01710:NZ_CP008957.1:347999-348100:-:21 | EDL933_RS01710(EDL933_RS01710) |
| NZ_CP008957.1 | 3875341 | 3875785 | 445 | 3875509 | 918 | 18.43412 | 1.35865 | 17.25387 | IP_LBN_vs_In_LBN_peak_360 | EDL933_RS20155:Promoter;EDL933_RS20150:Promoter;EDL933_RS33755:Promoter;EDL933_RS20160:Promoter | EDL933_RS20160:NZ_CP008957.1:3874326-3875421:-:-141 | EDL933_RS20160(gcvT) |
| NZ_CP008957.1 | 2294867 | 2295180 | 314 | 2295005 | 812 | 16.26581 | 1.35599 | 15.10729 | IP_LBN_vs_In_LBN_peak_216 | intergenic | EDL933_RS30580:NZ_CP008957.1:2295193-2295768:-:745 | EDL933_RS30580(EDL933_RS30580) |
| NZ_CP008957.1 | 1150377 | 1150987 | 611 | 1150757 | 811 | 16.13138 | 1.35433 | 14.97422 | IP_LBN_vs_In_LBN_peak_135 | EDL933_RS05675:Promoter;EDL933_RS05660:Promoter;EDL933_RS05670:Promoter;EDL933_RS05665:Promoter | EDL933_RS05665:NZ_CP008957.1:1149493-1150459:-:-222 | EDL933_RS05665(trxB) |
| NZ_CP008957.1 | 1118559 | 1118987 | 429 | 1118686 | 811 | 16.13138 | 1.35433 | 14.97422 | IP_LBN_vs_In_LBN_peak_132 | EDL933_RS05465:Promoter;EDL933_RS05470:CDS;EDL933_RS05480:Promoter | EDL933_RS05470:NZ_CP008957.1:1118219-1119433:-:660 | EDL933_RS05470(EDL933_RS05470) |
| NZ_CP008957.1 | 5159043 | 5159399 | 357 | 5159189 | 1061 | 20.56685 | 1.35271 | 19.36676 | IP_LBN_vs_In_LBN_peak_526 | EDL933_RS26665:CDS;EDL933_RS26655:Promoter;EDL933_RS26670:Promoter | EDL933_RS26665:NZ_CP008957.1:5159163-5159772:+:57 | EDL933_RS26665(lexA) |
| NZ_CP008957.1 | 573093 | 574704 | 1612 | 574482 | 918 | 17.92524 | 1.35217 | 16.74983 | IP_LBN_vs_In_LBN_peak_71 | EDL933_RS02745:Promoter;EDL933_RS02750:Promoter | EDL933_RS02750:NZ_CP008957.1:573075-573870:-:-28 | EDL933_RS02750(EDL933_RS02750) |
| NZ_CP008957.1 | 468187 | 469176 | 990 | 468561 | 839 | 16.44805 | 1.35157 | 15.28782 | IP_LBN_vs_In_LBN_peak_56 | EDL933_RS31985:CDS | EDL933_RS31985:NZ_CP008957.1:468383-470663:+:298 | EDL933_RS31985(EDL933_RS31985) |
| NZ_CP008957.1 | 3929914 | 3930247 | 334 | 3930105 | 896 | 17.35415 | 1.34992 | 16.18492 | IP_LBN_vs_In_LBN_peak_366 | EDL933_RS20440:Promoter;EDL933_RS20445:Promoter | EDL933_RS20445:NZ_CP008957.1:3928901-3929948:-:-132 | EDL933_RS20445(ansB) |
| NZ_CP008957.1 | 3830872 | 3831349 | 478 | 3831190 | 865 | 16.58012 | 1.34696 | 15.41853 | IP_LBN_vs_In_LBN_peak_357 | EDL933_RS19985:Promoter;EDL933_RS19980:CDS | EDL933_RS19980:NZ_CP008957.1:3829964-3831743:-:633 | EDL933_RS19980(EDL933_RS19980) |
| NZ_CP008957.1 | 93713 | 94430 | 718 | 94285 | 968 | 18.36874 | 1.34652 | 17.18895 | IP_LBN_vs_In_LBN_peak_6 | EDL933_RS00445:Promoter;EDL933_RS00440:CDS;EDL933_RS00450:Promoter;EDL933_RS00455:Promoter | EDL933_RS00440:NZ_CP008957.1:94239-94698:+:-168 | EDL933_RS00440(mraZ) |
| NZ_CP008957.1 | 1222189 | 1223565 | 1377 | 1222708 | 852 | 16.25505 | 1.34565 | 15.097 | IP_LBN_vs_In_LBN_peak_145 | EDL933_RS05950:Promoter;EDL933_RS05960:Promoter | EDL933_RS05950:NZ_CP008957.1:1222754-1223297:+:122 | EDL933_RS05950(zapC) |
| NZ_CP008957.1 | 3915032 | 3915668 | 637 | 3915402 | 903 | 17.10083 | 1.34496 | 15.93384 | IP_LBN_vs_In_LBN_peak_365 | EDL933_RS20370:Promoter;EDL933_RS20355:Promoter;EDL933_RS34010:CDS;EDL933_RS20350:Promoter | EDL933_RS34010:NZ_CP008957.1:3915298-3915514:+:51 | EDL933_RS34010(EDL933_RS34010) |
| NZ_CP008957.1 | 3964237 | 3964684 | 448 | 3964521 | 937 | 17.68828 | 1.34484 | 16.51543 | IP_LBN_vs_In_LBN_peak_371 | EDL933_RS20655:Promoter | EDL933_RS20655:NZ_CP008957.1:3963115-3964351:-:-109 | EDL933_RS20655(pitB) |
| NZ_CP008957.1 | 1254743 | 1255838 | 1096 | 1255586 | 889 | 16.59573 | 1.3415 | 15.43403 | IP_LBN_vs_In_LBN_peak_151 | EDL933_RS06160:Promoter;EDL933_RS06125:Promoter;EDL933_RS06140:CDS;EDL933_RS06130:Promoter;EDL933_RS06135:Promoter;EDL933_RS06120:Promoter;EDL933_RS06145:Promoter;EDL933_RS06150:Promoter | EDL933_RS06135:NZ_CP008957.1:1255073-1255265:-:-25 | EDL933_RS06135(EDL933_RS06135) |
| NZ_CP008957.1 | 4785906 | 4786156 | 251 | 4786040 | 1172 | 21.44124 | 1.34116 | 20.23399 | IP_LBN_vs_In_LBN_peak_474 | EDL933_RS24870:Promoter;EDL933_RS24875:Promoter | EDL933_RS24875:NZ_CP008957.1:4784816-4785857:-:-173 | EDL933_RS24875(pstS) |
| NZ_CP008957.1 | 4717638 | 4718109 | 472 | 4717853 | 1135 | 20.80017 | 1.3411 | 19.59831 | IP_LBN_vs_In_LBN_peak_465 | EDL933_RS24525:Promoter;EDL933_RS24535:Promoter;EDL933_RS24530:CDS | EDL933_RS24530:NZ_CP008957.1:4717211-4718545:-:672 | EDL933_RS24530(adeQ) |
| NZ_CP008957.1 | 1541586 | 1541980 | 395 | 1541690 | 802 | 14.94528 | 1.33932 | 13.80006 | IP_LBN_vs_In_LBN_peak_190 | EDL933_RS07775:Promoter;EDL933_RS07770:Promoter;EDL933_RS07780:Promoter;EDL933_RS07765:Promoter;EDL933_RS07755:CDS | EDL933_RS07755:NZ_CP008957.1:1541234-1542448:-:665 | EDL933_RS07755(EDL933_RS07755) |
| NZ_CP008957.1 | 616339 | 616641 | 303 | 616523 | 802 | 14.94528 | 1.33932 | 13.80006 | IP_LBN_vs_In_LBN_peak_76 | EDL933_RS02870:Promoter | EDL933_RS02870:NZ_CP008957.1:616747-620944:+:-257 | EDL933_RS02870(EDL933_RS02870) |
| NZ_CP008957.1 | 4786794 | 4787203 | 410 | 4786984 | 1178 | 21.30306 | 1.3387 | 20.09659 | IP_LBN_vs_In_LBN_peak_475 | EDL933_RS24875:Promoter;EDL933_RS24880:CDS | EDL933_RS24880:NZ_CP008957.1:4786068-4787151:-:153 | EDL933_RS24880(EDL933_RS24880) |
| NZ_CP008957.1 | 394697 | 395109 | 413 | 394923 | 869 | 16.03952 | 1.3386 | 14.88338 | IP_LBN_vs_In_LBN_peak_48 | EDL933_RS01925:Promoter;EDL933_RS01915:Promoter;EDL933_RS01920:Promoter | EDL933_RS01915:NZ_CP008957.1:395059-396043:+:-156 | EDL933_RS01915(EDL933_RS01915) |
| NZ_CP008957.1 | 3078184 | 3078664 | 481 | 3078382 | 800 | 14.68753 | 1.33598 | 13.54501 | IP_LBN_vs_In_LBN_peak_269 | EDL933_RS16300:Promoter;EDL933_RS16305:CDS;EDL933_RS16310:Promoter | EDL933_RS16305:NZ_CP008957.1:3077491-3078433:-:9 | EDL933_RS16305(rihB) |
| NZ_CP008957.1 | 2654417 | 2655171 | 755 | 2654624 | 800 | 14.68753 | 1.33598 | 13.54501 | IP_LBN_vs_In_LBN_peak_228 | EDL933_RS13925:Promoter;EDL933_RS13930:Promoter;EDL933_RS13945:Promoter | EDL933_RS13930:NZ_CP008957.1:2653423-2654524:-:-269 | EDL933_RS13930(EDL933_RS13930) |
| NZ_CP008957.1 | 5049011 | 5049298 | 288 | 5049140 | 1092 | 19.5438 | 1.33542 | 18.35271 | IP_LBN_vs_In_LBN_peak_511 | EDL933_RS26180:Promoter;EDL933_RS26175:Promoter;EDL933_RS26160:Promoter;EDL933_RS26165:Promoter;EDL933_RS26170:Promoter | EDL933_RS26165:NZ_CP008957.1:5047958-5049110:-:-44 | EDL933_RS26165(argE) |
| NZ_CP008957.1 | 1028947 | 1029191 | 245 | 1029072 | 799 | 14.55946 | 1.33431 | 13.41822 | IP_LBN_vs_In_LBN_peak_129 | EDL933_RS04935:Promoter | EDL933_RS04935:NZ_CP008957.1:1028211-1028943:-:-125 | EDL933_RS04935(artJ) |
| NZ_CP008957.1 | 368601 | 369743 | 1143 | 369545 | 931 | 16.75142 | 1.33417 | 15.58817 | IP_LBN_vs_In_LBN_peak_41 | EDL933_RS34075:Promoter;EDL933_RS01805:Promoter;EDL933_RS01800:Promoter | EDL933_RS01805:NZ_CP008957.1:368426-369521:-:349 | EDL933_RS01805(EDL933_RS01805) |
| NZ_CP008957.1 | 1591201 | 1591631 | 431 | 1591431 | 798 | 14.43192 | 1.33264 | 13.29202 | IP_LBN_vs_In_LBN_peak_194 | EDL933_RS08055:Promoter;EDL933_RS08065:Promoter | EDL933_RS08055:NZ_CP008957.1:1588022-1591208:-:-207 | EDL933_RS08055(rne) |
| NZ_CP008957.1 | 454223 | 454822 | 600 | 454528 | 819 | 14.76411 | 1.33241 | 13.62102 | IP_LBN_vs_In_LBN_peak_53 | EDL933_RS02170:CDS;EDL933_RS02185:Promoter | EDL933_RS02170:NZ_CP008957.1:454284-454908:+:238 | EDL933_RS02170(iprA) |
| NZ_CP008957.1 | 4525872 | 4526715 | 844 | 4526502 | 1100 | 19.36003 | 1.33193 | 18.17047 | IP_LBN_vs_In_LBN_peak_438 | EDL933_RS23560:CDS;EDL933_RS23570:Promoter;EDL933_RS23555:Promoter;EDL933_RS23565:Promoter;EDL933_RS23545:Promoter;EDL933_RS23540:Promoter | EDL933_RS23560:NZ_CP008957.1:4526227-4527799:+:66 | EDL933_RS23560(bcsE) |
| NZ_CP008957.1 | 5255524 | 5256496 | 973 | 5256055 | 1026 | 18.07225 | 1.3311 | 16.89538 | IP_LBN_vs_In_LBN_peak_538 | EDL933_RS27120:Promoter;EDL933_RS27115:CDS;EDL933_RS27100:Promoter | EDL933_RS27115:NZ_CP008957.1:5255899-5256196:+:110 | EDL933_RS27115(ghoS) |
| NZ_CP008957.1 | 3529686 | 3529932 | 247 | 3529781 | 855 | 14.93347 | 1.32639 | 13.78871 | IP_LBN_vs_In_LBN_peak_317 | EDL933_RS18405:Promoter;EDL933_RS18410:Promoter | EDL933_RS18405:NZ_CP008957.1:3529814-3531170:+:-5 | EDL933_RS18405(pssA) |
| NZ_CP008957.1 | 2850235 | 2850510 | 276 | 2850358 | 794 | 13.9271 | 1.32597 | 12.79254 | IP_LBN_vs_In_LBN_peak_244 | EDL933_RS15115:Promoter;EDL933_RS15110:Promoter;EDL933_RS15120:Promoter;EDL933_RS15125:Promoter;EDL933_RS30885:Promoter | EDL933_RS30885:NZ_CP008957.1:2850368-2850419:+:4 | EDL933_RS30885(hisL) |
| NZ_CP008957.1 | 2601444 | 2601930 | 487 | 2601615 | 794 | 13.9271 | 1.32597 | 12.79254 | IP_LBN_vs_In_LBN_peak_225 | EDL933_RS13645:Promoter | EDL933_RS13645:NZ_CP008957.1:2601979-2602546:+:-292 | EDL933_RS13645(mntP) |
| NZ_CP008957.1 | 656774 | 657095 | 322 | 656925 | 794 | 13.9271 | 1.32597 | 12.79254 | IP_LBN_vs_In_LBN_peak_87 | EDL933_RS03040:Promoter;EDL933_RS03045:exon | EDL933_RS03045:NZ_CP008957.1:656872-656949:+:62 | EDL933_RS03045(EDL933_RS03045) |
| NZ_CP008957.1 | 4570558 | 4571083 | 526 | 4570947 | 1094 | 18.22436 | 1.32036 | 17.046 | IP_LBN_vs_In_LBN_peak_444 | EDL933_RS23775:Promoter;EDL933_RS23780:Promoter | EDL933_RS23775:NZ_CP008957.1:4570058-4570883:-:63 | EDL933_RS23775(EDL933_RS23775) |
| NZ_CP008957.1 | 5129344 | 5129686 | 343 | 5129520 | 1084 | 17.99163 | 1.31948 | 16.8157 | IP_LBN_vs_In_LBN_peak_521 | EDL933_RS26505:Promoter;EDL933_RS26515:Promoter;EDL933_RS26510:Promoter | EDL933_RS26515:NZ_CP008957.1:5128667-5129465:-:-49 | EDL933_RS26515(EDL933_RS26515) |
| NZ_CP008957.1 | 3302981 | 3303396 | 416 | 3303241 | 808 | 13.63545 | 1.31817 | 12.50428 | IP_LBN_vs_In_LBN_peak_295 | EDL933_RS17320:CDS | EDL933_RS17320:NZ_CP008957.1:3303164-3304306:-:1118 | EDL933_RS17320(yfdE) |
| NZ_CP008957.1 | 4113041 | 4114044 | 1004 | 4113193 | 974 | 16.0419 | 1.31631 | 14.88574 | IP_LBN_vs_In_LBN_peak_385 | EDL933_RS21415:Promoter;EDL933_RS21410:Promoter;EDL933_RS21420:Promoter | EDL933_RS21410:NZ_CP008957.1:4113336-4113813:+:206 | EDL933_RS21410(agaB) |
| NZ_CP008957.1 | 4941504 | 4941883 | 380 | 4941697 | 1101 | 17.96848 | 1.31627 | 16.79272 | IP_LBN_vs_In_LBN_peak_497 | EDL933_RS25630:Promoter;EDL933_RS25625:Promoter;EDL933_RS25640:Promoter | EDL933_RS25640:NZ_CP008957.1:4941831-4943655:+:-138 | EDL933_RS25640(typA) |
| NZ_CP008957.1 | 1151335 | 1151881 | 547 | 1151528 | 788 | 13.18597 | 1.31597 | 12.05944 | IP_LBN_vs_In_LBN_peak_136 | EDL933_RS05675:Promoter;EDL933_RS05665:Promoter | EDL933_RS05675:NZ_CP008957.1:1151632-1155661:+:-24 | EDL933_RS05675(ftsK) |
| NZ_CP008957.1 | 4738255 | 4738914 | 660 | 4738739 | 1168 | 18.82141 | 1.31452 | 17.63709 | IP_LBN_vs_In_LBN_peak_469 | EDL933_RS24645:CDS;EDL933_RS24640:Promoter | EDL933_RS24645:NZ_CP008957.1:4738382-4739276:+:202 | EDL933_RS24645(EDL933_RS24645) |
| NZ_CP008957.1 | 1271978 | 1272282 | 305 | 1272131 | 787 | 13.06434 | 1.3143 | 11.93923 | IP_LBN_vs_In_LBN_peak_154 | EDL933_RS33165:Promoter;EDL933_RS06270:Promoter;EDL933_RS06280:Promoter;EDL933_RS06260:Promoter | EDL933_RS06270:NZ_CP008957.1:1271944-1272058:-:-71 | EDL933_RS06270(EDL933_RS06270) |
| NZ_CP008957.1 | 4254275 | 4254662 | 388 | 4254456 | 978 | 15.93597 | 1.3142 | 14.78101 | IP_LBN_vs_In_LBN_peak_407 | EDL933_RS22160:Promoter;EDL933_RS22130:Promoter;EDL933_RS22135:Promoter;EDL933_RS22145:CDS;EDL933_RS22140:Promoter | EDL933_RS22145:NZ_CP008957.1:4254058-4254601:-:133 | EDL933_RS22145(EDL933_RS22145) |
| NZ_CP008957.1 | 1014782 | 1015283 | 502 | 1015045 | 786 | 12.94324 | 1.31263 | 11.81952 | IP_LBN_vs_In_LBN_peak_126 | EDL933_RS04870:Promoter;EDL933_RS04865:Promoter;EDL933_RS04855:Promoter;EDL933_RS04875:Promoter;EDL933_RS04850:Promoter | EDL933_RS04855:NZ_CP008957.1:1015146-1015524:+:-114 | EDL933_RS04855(EDL933_RS04855) |
| NZ_CP008957.1 | 4419809 | 4420221 | 413 | 4419952 | 1021 | 16.38768 | 1.31182 | 15.22827 | IP_LBN_vs_In_LBN_peak_422 | EDL933_RS23045:Promoter;EDL933_RS23055:Promoter;EDL933_RS23065:Promoter;EDL933_RS23060:Promoter | EDL933_RS23055:NZ_CP008957.1:4420179-4420557:+:-164 | EDL933_RS23055(EDL933_RS23055) |
| NZ_CP008957.1 | 4378146 | 4378453 | 308 | 4378291 | 988 | 15.7818 | 1.31036 | 14.62837 | IP_LBN_vs_In_LBN_peak_418 | EDL933_RS22825:Promoter;EDL933_RS22815:Promoter;EDL933_RS22820:CDS | EDL933_RS22820:NZ_CP008957.1:4377201-4378305:-:6 | EDL933_RS22820(asd) |
| NZ_CP008957.1 | 5015875 | 5016397 | 523 | 5016023 | 1111 | 17.57284 | 1.31013 | 16.40125 | IP_LBN_vs_In_LBN_peak_509 | EDL933_RS26030:Promoter;EDL933_RS26025:Promoter;EDL933_RS26020:Promoter | EDL933_RS26025:NZ_CP008957.1:5016130-5016343:+:5 | EDL933_RS26025(rpmE) |
| NZ_CP008957.1 | 4501587 | 4502141 | 555 | 4501850 | 1067 | 16.82025 | 1.30889 | 15.65609 | IP_LBN_vs_In_LBN_peak_435 | EDL933_RS23475:Promoter;EDL933_RS23465:Promoter;EDL933_RS23470:Promoter | EDL933_RS23465:NZ_CP008957.1:4501052-4501655:-:-208 | EDL933_RS23465(EDL933_RS23465) |
| NZ_CP008957.1 | 362081 | 362801 | 721 | 362277 | 829 | 13.26587 | 1.30781 | 12.13869 | IP_LBN_vs_In_LBN_peak_38 | EDL933_RS01765:CDS;EDL933_RS01770:Promoter;EDL933_RS01755:Promoter;EDL933_RS01760:Promoter | EDL933_RS01765:NZ_CP008957.1:361390-362716:-:275 | EDL933_RS01765(EDL933_RS01765) |
| NZ_CP008957.1 | 557115 | 557386 | 272 | 557193 | 820 | 13.05934 | 1.30666 | 11.93462 | IP_LBN_vs_In_LBN_peak_69 | EDL933_RS02690:Promoter;EDL933_RS02670:Promoter;EDL933_RS02685:Promoter;EDL933_RS02675:Promoter | EDL933_RS02685:NZ_CP008957.1:557306-557858:+:-56 | EDL933_RS02685(apt) |
| NZ_CP008957.1 | 926608 | 927475 | 868 | 926843 | 782 | 12.46428 | 1.30596 | 11.34599 | IP_LBN_vs_In_LBN_peak_114 | EDL933_RS04430:Promoter;EDL933_RS29640:Promoter;EDL933_RS33085:Promoter;EDL933_RS04440:Promoter | EDL933_RS29640:NZ_CP008957.1:926921-927083:+:120 | EDL933_RS29640(EDL933_RS29640) |
| NZ_CP008957.1 | 4469986 | 4470400 | 415 | 4470247 | 1021 | 15.83979 | 1.3051 | 14.6859 | IP_LBN_vs_In_LBN_peak_429 | EDL933_RS23335:Promoter;EDL933_RS23330:Promoter;EDL933_RS23315:Promoter;EDL933_RS23325:Promoter | EDL933_RS23325:NZ_CP008957.1:4470467-4470821:+:-274 | EDL933_RS23325(arsR) |
| NZ_CP008958.1 | 11142 | 11562 | 421 | 11356 | 1792 | 26.60941 | 1.30344 | 25.36063 | IP_LBN_vs_In_LBN_peak_592 | EDL933_RS28645:CDS | EDL933_RS28645:NZ_CP008958.1:11241-15144:+:110 | EDL933_RS28645(espP) |
| NZ_CP008957.1 | 4564590 | 4564869 | 280 | 4564714 | 1056 | 16.20026 | 1.30341 | 15.04261 | IP_LBN_vs_In_LBN_peak_443 | EDL933_RS23760:Promoter;EDL933_RS23755:Promoter;EDL933_RS23745:Promoter;EDL933_RS23750:Promoter | EDL933_RS23750:NZ_CP008957.1:4563236-4564559:-:-170 | EDL933_RS23750(xylA) |
| NZ_CP008957.1 | 885049 | 885429 | 381 | 885201 | 798 | 12.50898 | 1.30303 | 11.39032 | IP_LBN_vs_In_LBN_peak_110 | EDL933_RS04165:Promoter;EDL933_RS04155:Promoter;EDL933_RS04160:CDS;EDL933_RS04170:Promoter | EDL933_RS04165:NZ_CP008957.1:885530-886964:+:-291 | EDL933_RS04165(EDL933_RS04165) |
| NZ_CP008957.1 | 3675304 | 3675627 | 324 | 3675451 | 924 | 14.29512 | 1.30299 | 13.15698 | IP_LBN_vs_In_LBN_peak_340 | EDL933_RS19195:CDS;EDL933_RS19185:Promoter | EDL933_RS19195:NZ_CP008957.1:3674707-3675700:-:235 | EDL933_RS19195(rpoS) |
| NZ_CP008957.1 | 4441649 | 4442142 | 494 | 4441826 | 1035 | 15.8025 | 1.30219 | 14.64892 | IP_LBN_vs_In_LBN_peak_424 | EDL933_RS23190:Promoter;EDL933_RS23185:Promoter;EDL933_RS23180:CDS;EDL933_RS23195:Promoter | EDL933_RS23185:NZ_CP008957.1:4442187-4442661:+:-292 | EDL933_RS23185(EDL933_RS23185) |
| NZ_CP008957.1 | 699468 | 699757 | 290 | 699637 | 777 | 11.87781 | 1.29762 | 10.76662 | IP_LBN_vs_In_LBN_peak_92 | EDL933_RS03210:Promoter | EDL933_RS03210:NZ_CP008957.1:699687-700821:+:-75 | EDL933_RS03210(fepE) |
| NZ_CP008957.1 | 478955 | 479215 | 261 | 479067 | 787 | 11.85 | 1.29494 | 10.73942 | IP_LBN_vs_In_LBN_peak_57 | EDL933_RS02305:Promoter;EDL933_RS02300:Promoter;EDL933_RS02315:Promoter;EDL933_RS02310:Promoter | EDL933_RS02310:NZ_CP008957.1:479122-479812:+:-37 | EDL933_RS02310(phoB) |
| NZ_CP008957.1 | 3309561 | 3309981 | 421 | 3309707 | 775 | 11.64704 | 1.29428 | 10.53874 | IP_LBN_vs_In_LBN_peak_299 | EDL933_RS17345:Promoter;EDL933_RS17365:Promoter;EDL933_RS17350:Promoter;EDL933_RS17340:Promoter | EDL933_RS17350:NZ_CP008957.1:3309835-3310111:+:-64 | EDL933_RS17350(EDL933_RS17350) |
| NZ_CP008957.1 | 5448234 | 5449111 | 878 | 5448516 | 962 | 14.09153 | 1.29316 | 12.95531 | IP_LBN_vs_In_LBN_peak_572 | EDL933_RS28005:CDS;EDL933_RS28010:Promoter | EDL933_RS28010:NZ_CP008957.1:5448870-5451507:+:-198 | EDL933_RS28010(fimD) |
| NZ_CP008957.1 | 5441256 | 5442244 | 989 | 5441849 | 940 | 13.7705 | 1.2928 | 12.63787 | IP_LBN_vs_In_LBN_peak_569 | EDL933_RS27955:Promoter;EDL933_RS32920:Promoter;EDL933_RS27960:CDS | EDL933_RS27955:NZ_CP008957.1:5440405-5441386:-:-363 | EDL933_RS27955(EDL933_RS27955) |
| NZ_CP008957.1 | 627883 | 628799 | 917 | 628635 | 780 | 11.49841 | 1.29073 | 10.39223 | IP_LBN_vs_In_LBN_peak_78 | EDL933_RS02915:Promoter;EDL933_RS02920:Promoter;EDL933_RS02910:CDS | EDL933_RS02910:NZ_CP008957.1:627993-628770:+:347 | EDL933_RS02910(hyi) |
| NZ_CP008957.1 | 630929 | 631319 | 391 | 631182 | 780 | 11.48839 | 1.29057 | 10.38229 | IP_LBN_vs_In_LBN_peak_80 | EDL933_RS02920:CDS;EDL933_RS02925:Promoter;EDL933_RS02930:Promoter | EDL933_RS02925:NZ_CP008957.1:631345-632707:+:-221 | EDL933_RS02925(allB) |
| NZ_CP008957.1 | 3694559 | 3694916 | 358 | 3694713 | 825 | 11.99269 | 1.28912 | 10.88041 | IP_LBN_vs_In_LBN_peak_342 | EDL933_RS19295:CDS | EDL933_RS19295:NZ_CP008957.1:3692015-3694715:-:-22 | EDL933_RS19295(EDL933_RS19295) |
| NZ_CP008957.1 | 371370 | 371651 | 282 | 371499 | 818 | 11.81078 | 1.28771 | 10.70058 | IP_LBN_vs_In_LBN_peak_42 | EDL933_RS01805:Promoter;EDL933_RS01815:Promoter;EDL933_RS01810:Promoter;EDL933_RS33980:Promoter | EDL933_RS01815:NZ_CP008957.1:370477-371272:-:-238 | EDL933_RS01815(hyxR) |
| NZ_CP008957.1 | 4032837 | 4033657 | 821 | 4032956 | 986 | 13.97679 | 1.28743 | 12.84184 | IP_LBN_vs_In_LBN_peak_376 | EDL933_RS21035:Promoter;EDL933_RS21025:CDS;EDL933_RS21015:Promoter;EDL933_RS21010:Promoter;EDL933_RS21030:Promoter | EDL933_RS21025:NZ_CP008957.1:4032480-4033413:-:166 | EDL933_RS21025(ttdR) |
| NZ_CP008957.1 | 4325931 | 4326203 | 273 | 4326070 | 984 | 13.82207 | 1.28573 | 12.68863 | IP_LBN_vs_In_LBN_peak_416 | EDL933_RS22620:Promoter;EDL933_RS22625:Promoter | EDL933_RS22620:NZ_CP008957.1:4325409-4325970:-:-96 | EDL933_RS22620(nudE) |
| NZ_CP008957.1 | 829199 | 829611 | 413 | 829423 | 769 | 10.96789 | 1.28428 | 9.86828 | IP_LBN_vs_In_LBN_peak_105 | EDL933_RS03840:Promoter;EDL933_RS03845:Promoter | EDL933_RS03845:NZ_CP008957.1:828653-829220:-:-184 | EDL933_RS03845(EDL933_RS03845) |
| NZ_CP008957.1 | 4183930 | 4184227 | 298 | 4184038 | 946 | 13.21761 | 1.28413 | 12.09081 | IP_LBN_vs_In_LBN_peak_399 | EDL933_RS21795:Promoter;EDL933_RS21805:Promoter;EDL933_RS21800:Promoter | EDL933_RS21800:NZ_CP008957.1:4183100-4184030:-:-48 | EDL933_RS21800(EDL933_RS21800) |
| NZ_CP008957.1 | 3670881 | 3671661 | 781 | 3671017 | 825 | 11.63435 | 1.28349 | 10.52652 | IP_LBN_vs_In_LBN_peak_339 | EDL933_RS19170:Promoter | EDL933_RS19170:NZ_CP008957.1:3671069-3671726:+:201 | EDL933_RS19170(pphB) |
| NZ_CP008957.1 | 5070489 | 5071065 | 577 | 5070728 | 1101 | 15.08017 | 1.28283 | 13.93359 | IP_LBN_vs_In_LBN_peak_514 | EDL933_RS26290:Promoter;EDL933_RS26285:Promoter;EDL933_RS26270:Promoter;EDL933_RS26275:Promoter;EDL933_RS26260:CDS;EDL933_RS26280:Promoter | EDL933_RS26260:NZ_CP008957.1:5070718-5071669:-:892 | EDL933_RS26260(coaA) |
| NZ_CP008957.1 | 3676026 | 3676365 | 340 | 3676208 | 817 | 11.46024 | 1.28234 | 10.35441 | IP_LBN_vs_In_LBN_peak_341 | EDL933_RS19195:Promoter;EDL933_RS19200:CDS | EDL933_RS19195:NZ_CP008957.1:3674707-3675700:-:-495 | EDL933_RS19195(rpoS) |
| NZ_CP008957.1 | 1418078 | 1418507 | 430 | 1418284 | 835 | 11.60006 | 1.28097 | 10.49254 | IP_LBN_vs_In_LBN_peak_183 | EDL933_RS07150:CDS;EDL933_RS07145:Promoter | EDL933_RS07150:NZ_CP008957.1:1417216-1419235:-:943 | EDL933_RS07150(pgaB) |
| NZ_CP008957.1 | 2932661 | 2933076 | 416 | 2932921 | 766 | 10.63576 | 1.27927 | 9.54046 | IP_LBN_vs_In_LBN_peak_254 | EDL933_RS15460:Promoter;EDL933_RS15470:CDS | EDL933_RS15470:NZ_CP008957.1:2932804-2933578:-:710 | EDL933_RS15470(EDL933_RS15470) |
| NZ_CP008957.1 | 2290563 | 2291119 | 557 | 2291033 | 766 | 10.63576 | 1.27927 | 9.54046 | IP_LBN_vs_In_LBN_peak_214 | EDL933_RS12000:Promoter;EDL933_RS11990:Promoter;EDL933_RS11995:CDS | EDL933_RS11990:NZ_CP008957.1:2290155-2290725:-:-115 | EDL933_RS11990(nleF) |
| NZ_CP008957.1 | 3554579 | 3554858 | 280 | 3554738 | 804 | 11.04672 | 1.27828 | 9.94631 | IP_LBN_vs_In_LBN_peak_325 | EDL933_RS18535:Promoter;EDL933_RS18520:Promoter;EDL933_RS18530:Promoter;EDL933_RS18515:Promoter;EDL933_RS18525:Promoter | EDL933_RS18525:NZ_CP008957.1:3553330-3554692:-:-26 | EDL933_RS18525(ffh) |
| NZ_CP008957.1 | 4587957 | 4588522 | 566 | 4588345 | 1059 | 14.16672 | 1.27806 | 13.02989 | IP_LBN_vs_In_LBN_peak_448 | EDL933_RS23855:CDS;EDL933_RS23845:Promoter;EDL933_RS23850:Promoter | EDL933_RS23850:NZ_CP008957.1:4587000-4588152:-:-87 | EDL933_RS23850(yiaY) |
| NZ_CP008957.1 | 4054601 | 4054904 | 304 | 4054774 | 881 | 11.98144 | 1.27806 | 10.86927 | IP_LBN_vs_In_LBN_peak_379 | EDL933_RS21125:Promoter;EDL933_RS21120:Promoter;EDL933_RS21115:CDS | EDL933_RS21120:NZ_CP008957.1:4054958-4056029:+:-206 | EDL933_RS21120(ygjJ) |
| NZ_CP008957.1 | 461510 | 461742 | 233 | 461636 | 815 | 11.15458 | 1.27783 | 10.05275 | IP_LBN_vs_In_LBN_peak_55 | EDL933_RS02235:Promoter;EDL933_RS02230:Promoter;EDL933_RS02210:Promoter;EDL933_RS02225:CDS | EDL933_RS02225:NZ_CP008957.1:461525-462941:+:100 | EDL933_RS02225(phoA) |
| NZ_CP008957.1 | 4457935 | 4458291 | 357 | 4458114 | 992 | 13.2845 | 1.27725 | 12.15717 | IP_LBN_vs_In_LBN_peak_427 | EDL933_RS23275:Promoter;EDL933_RS23245:Promoter | EDL933_RS23260:NZ_CP008957.1:4456946-4457999:+:1166 | EDL933_RS23260(EDL933_RS23260) |
| NZ_CP008957.1 | 551602 | 551937 | 336 | 551743 | 798 | 10.83716 | 1.27604 | 9.73953 | IP_LBN_vs_In_LBN_peak_67 | EDL933_RS02660:Promoter;EDL933_RS02655:Promoter;EDL933_RS02665:Promoter;EDL933_RS02650:Promoter | EDL933_RS02660:NZ_CP008957.1:551825-552473:+:-56 | EDL933_RS02660(acrR) |
| NZ_CP008957.1 | 3511225 | 3511536 | 312 | 3511385 | 764 | 10.41709 | 1.27594 | 9.32469 | IP_LBN_vs_In_LBN_peak_313 | EDL933_RS18300:Promoter;EDL933_RS18295:Promoter;EDL933_RS18305:Promoter | EDL933_RS18305:NZ_CP008957.1:3510657-3511338:-:-42 | EDL933_RS18305(rnc) |
| NZ_CP008957.1 | 3452597 | 3452902 | 306 | 3452764 | 764 | 10.41709 | 1.27594 | 9.32469 | IP_LBN_vs_In_LBN_peak_309 | EDL933_RS18050:Promoter | EDL933_RS18050:NZ_CP008957.1:3450330-3452712:-:-37 | EDL933_RS18050(EDL933_RS18050) |
| NZ_CP008957.1 | 3153321 | 3153656 | 336 | 3153463 | 764 | 10.41709 | 1.27594 | 9.32469 | IP_LBN_vs_In_LBN_peak_277 | EDL933_RS16635:Promoter;EDL933_RS16630:CDS | EDL933_RS16630:NZ_CP008957.1:3150881-3153509:-:21 | EDL933_RS16630(gyrA) |
| NZ_CP008957.1 | 3061062 | 3061531 | 470 | 3061296 | 764 | 10.41709 | 1.27594 | 9.32469 | IP_LBN_vs_In_LBN_peak_268 | EDL933_RS16225:CDS;EDL933_RS16220:Promoter | EDL933_RS16220:NZ_CP008957.1:3060055-3061066:-:-230 | EDL933_RS16220(mglC) |
| NZ_CP008957.1 | 4972972 | 4973305 | 334 | 4973192 | 1081 | 14.23622 | 1.2756 | 13.09855 | IP_LBN_vs_In_LBN_peak_504 | EDL933_RS25785:Promoter;EDL933_RS25800:Promoter | EDL933_RS25800:NZ_CP008957.1:4973263-4974319:+:-125 | EDL933_RS25800(EDL933_RS25800) |
| NZ_CP008957.1 | 4953425 | 4953919 | 495 | 4953685 | 1065 | 14.02886 | 1.27542 | 12.89348 | IP_LBN_vs_In_LBN_peak_501 | EDL933_RS25680:Promoter;EDL933_RS25675:Promoter | EDL933_RS25680:NZ_CP008957.1:4952807-4953683:-:11 | EDL933_RS25680(EDL933_RS25680) |
| NZ_CP008957.1 | 3542923 | 3543193 | 271 | 3543057 | 763 | 10.30859 | 1.27427 | 9.21766 | IP_LBN_vs_In_LBN_peak_320 | EDL933_RS18450:Promoter;EDL933_RS18470:Promoter;EDL933_RS33705:Promoter;EDL933_RS18465:Promoter;EDL933_RS18445:Promoter;EDL933_RS18440:Promoter;EDL933_RS18455:Promoter | EDL933_RS18450:NZ_CP008957.1:3542038-3543019:-:-38 | EDL933_RS18450(rluD) |
| NZ_CP008957.1 | 2756647 | 2756928 | 282 | 2756770 | 763 | 10.30859 | 1.27427 | 9.21766 | IP_LBN_vs_In_LBN_peak_235 | EDL933_RS14560:CDS;EDL933_RS14575:Promoter;EDL933_RS14570:Promoter | EDL933_RS14560:NZ_CP008957.1:2755795-2757154:-:367 | EDL933_RS14560(hprS) |
| NZ_CP008957.1 | 4170015 | 4170406 | 392 | 4170233 | 922 | 12.20222 | 1.27399 | 11.08733 | IP_LBN_vs_In_LBN_peak_397 | EDL933_RS21715:Promoter;EDL933_RS21730:Promoter;EDL933_RS21710:Promoter;EDL933_RS21725:Promoter;EDL933_RS21720:Promoter | EDL933_RS21725:NZ_CP008957.1:4170254-4171232:+:-44 | EDL933_RS21725(EDL933_RS21725) |
| NZ_CP008957.1 | 89723 | 90213 | 491 | 89974 | 910 | 12.02987 | 1.27357 | 10.9169 | IP_LBN_vs_In_LBN_peak_5 | EDL933_RS00420:Promoter;EDL933_RS00425:Promoter;EDL933_RS00410:Promoter;EDL933_RS00405:Promoter | EDL933_RS00420:NZ_CP008957.1:90235-91960:+:-267 | EDL933_RS00420(ilvI) |
| NZ_CP008957.1 | 4079965 | 4080646 | 682 | 4080121 | 917 | 12.10844 | 1.2735 | 10.99476 | IP_LBN_vs_In_LBN_peak_381 | EDL933_RS21260:Promoter;EDL933_RS21250:Promoter | EDL933_RS21250:NZ_CP008957.1:4080166-4080523:+:139 | EDL933_RS21250(EDL933_RS21250) |
| NZ_CP008958.1 | 12535 | 13492 | 958 | 13269 | 1718 | 21.59049 | 1.27315 | 20.38203 | IP_LBN_vs_In_LBN_peak_594 | EDL933_RS28645:CDS | EDL933_RS28645:NZ_CP008958.1:11241-15144:+:1772 | EDL933_RS28645(espP) |
| NZ_CP008957.1 | 629700 | 630162 | 463 | 629844 | 762 | 10.20065 | 1.2726 | 9.11117 | IP_LBN_vs_In_LBN_peak_79 | EDL933_RS02920:Promoter;EDL933_RS02925:Promoter | EDL933_RS02920:NZ_CP008957.1:629917-631309:+:13 | EDL933_RS02920(EDL933_RS02920) |
| NZ_CP008958.1 | 9145 | 9738 | 594 | 9540 | 1705 | 21.24905 | 1.27167 | 20.04329 | IP_LBN_vs_In_LBN_peak_591 | EDL933_RS28640:Promoter;EDL933_RS28630:CDS;EDL933_RS28635:Promoter;EDL933_RS28645:Promoter | EDL933_RS28635:NZ_CP008958.1:9626-10016:+:-185 | EDL933_RS28635(EDL933_RS28635) |
| NZ_CP008957.1 | 2877963 | 2878261 | 299 | 2878042 | 761 | 10.09326 | 1.27093 | 9.00531 | IP_LBN_vs_In_LBN_peak_248 | EDL933_RS15245:Promoter;EDL933_RS15240:Promoter | EDL933_RS15245:NZ_CP008957.1:2877036-2878032:-:-79 | EDL933_RS15245(EDL933_RS15245) |
| NZ_CP008957.1 | 4197429 | 4197775 | 347 | 4197575 | 965 | 12.42154 | 1.26989 | 11.30396 | IP_LBN_vs_In_LBN_peak_400 | EDL933_RS21850:Promoter;EDL933_RS21840:Promoter;EDL933_RS21845:Promoter | EDL933_RS21850:NZ_CP008957.1:4197742-4199107:+:-140 | EDL933_RS21850(dcuC) |
| NZ_CP008957.1 | 3307047 | 3307461 | 415 | 3307258 | 760 | 9.98642 | 1.26926 | 8.89996 | IP_LBN_vs_In_LBN_peak_297 | EDL933_RS17340:CDS;EDL933_RS17330:Promoter;EDL933_RS17335:Promoter | EDL933_RS17335:NZ_CP008957.1:3305393-3307088:-:-165 | EDL933_RS17335(oxc) |
| NZ_CP008957.1 | 939082 | 939474 | 393 | 939217 | 760 | 9.98642 | 1.26926 | 8.89996 | IP_LBN_vs_In_LBN_peak_116 | EDL933_RS04500:Promoter;EDL933_RS04495:Promoter;EDL933_RS04490:CDS;EDL933_RS04480:Promoter;EDL933_RS04505:Promoter | EDL933_RS04490:NZ_CP008957.1:939179-940169:+:98 | EDL933_RS04490(moaA) |
| NZ_CP008957.1 | 297028 | 297471 | 444 | 297257 | 828 | 10.776 | 1.26919 | 9.67898 | IP_LBN_vs_In_LBN_peak_22 | EDL933_RS01410:Promoter;EDL933_RS01405:Promoter;EDL933_RS01400:Promoter | EDL933_RS01400:NZ_CP008957.1:296156-297212:-:-37 | EDL933_RS01400(phoE) |
| NZ_CP008957.1 | 1203748 | 1204119 | 372 | 1203894 | 760 | 9.87744 | 1.26734 | 8.79284 | IP_LBN_vs_In_LBN_peak_142 | EDL933_RS05855:Promoter;EDL933_RS05865:Promoter | EDL933_RS05865:NZ_CP008957.1:1202696-1203785:-:-148 | EDL933_RS05865(ompF) |
| NZ_CP008957.1 | 4973632 | 4974020 | 389 | 4973797 | 1073 | 13.4502 | 1.26697 | 12.32082 | IP_LBN_vs_In_LBN_peak_505 | EDL933_RS25800:CDS;EDL933_RS25785:Promoter | EDL933_RS25800:NZ_CP008957.1:4973263-4974319:+:562 | EDL933_RS25800(EDL933_RS25800) |
| NZ_CP008957.1 | 4318821 | 4319144 | 324 | 4319006 | 959 | 12.11018 | 1.2665 | 10.99648 | IP_LBN_vs_In_LBN_peak_415 | EDL933_RS22580:Promoter;EDL933_RS22585:Promoter;EDL933_RS22575:Promoter | EDL933_RS22585:NZ_CP008957.1:4318365-4318887:-:-95 | EDL933_RS22585(aroK) |
| NZ_CP008957.1 | 1377762 | 1378282 | 521 | 1378015 | 3000 | 34.84949 | 1.26466 | 33.53988 | IP_LBN_vs_In_LBN_peak_174 | EDL933_RS06980:Promoter;EDL933_RS06985:Promoter;EDL933_RS06990:Promoter;EDL933_RS06965:Promoter;EDL933_RS06960:CDS | EDL933_RS06965:NZ_CP008957.1:1378175-1378910:+:-153 | EDL933_RS06965(EDL933_RS06965) |
| NZ_CP008957.1 | 2031251 | 2032137 | 887 | 2031866 | 757 | 9.66926 | 1.26426 | 8.58734 | IP_LBN_vs_In_LBN_peak_208 | EDL933_RS10635:Promoter;EDL933_RS10630:CDS | EDL933_RS10630:NZ_CP008957.1:2030923-2032183:+:770 | EDL933_RS10630(espR1) |
| NZ_CP008957.1 | 942794 | 943403 | 610 | 943109 | 757 | 9.66926 | 1.26426 | 8.58734 | IP_LBN_vs_In_LBN_peak_117 | EDL933_RS04520:CDS | EDL933_RS04520:NZ_CP008957.1:942930-943644:+:168 | EDL933_RS04520(EDL933_RS04520) |
| NZ_CP008957.1 | 3078901 | 3079226 | 326 | 3079094 | 756 | 9.56465 | 1.26259 | 8.48424 | IP_LBN_vs_In_LBN_peak_270 | EDL933_RS16300:Promoter;EDL933_RS16310:CDS;EDL933_RS16305:Promoter | EDL933_RS16310:NZ_CP008957.1:3078562-3079261:+:501 | EDL933_RS16310(yeiL) |
| NZ_CP008957.1 | 2931303 | 2931668 | 366 | 2931541 | 756 | 9.56465 | 1.26259 | 8.48424 | IP_LBN_vs_In_LBN_peak_253 | EDL933_RS15460:Promoter;EDL933_RS15465:Promoter | EDL933_RS15460:NZ_CP008957.1:2931100-2931418:-:-67 | EDL933_RS15460(EDL933_RS15460) |
| NZ_CP008957.1 | 678947 | 679390 | 444 | 679158 | 765 | 9.60024 | 1.26144 | 8.51945 | IP_LBN_vs_In_LBN_peak_91 | EDL933_RS03105:Promoter;EDL933_RS03110:Promoter;EDL933_RS03130:Promoter;EDL933_RS03125:Promoter;EDL933_RS03120:Promoter;EDL933_RS03115:CDS | EDL933_RS03120:NZ_CP008957.1:679443-679776:+:-275 | EDL933_RS03120(cusF) |
| NZ_CP008957.1 | 1026056 | 1026762 | 707 | 1026506 | 755 | 9.46061 | 1.26092 | 8.38171 | IP_LBN_vs_In_LBN_peak_128 | EDL933_RS04925:CDS;EDL933_RS04930:Promoter | EDL933_RS04925:NZ_CP008957.1:1026347-1026668:+:61 | EDL933_RS04925(EDL933_RS04925) |
| NZ_CP008957.1 | 4799896 | 4800217 | 322 | 4800018 | 1086 | 13.00202 | 1.25947 | 11.87778 | IP_LBN_vs_In_LBN_peak_480 | EDL933_RS24935:CDS;EDL933_RS24925:Promoter;EDL933_RS24930:Promoter | EDL933_RS24930:NZ_CP008957.1:4798469-4799333:-:-723 | EDL933_RS24930(atpG) |
| NZ_CP008957.1 | 2497265 | 2497739 | 475 | 2497572 | 754 | 9.35712 | 1.25926 | 8.27978 | IP_LBN_vs_In_LBN_peak_220 | EDL933_RS13095:Promoter;EDL933_RS13085:Promoter;EDL933_RS13090:CDS | EDL933_RS13090:NZ_CP008957.1:2497266-2499165:+:235 | EDL933_RS13090(espL1) |
| NZ_CP008957.1 | 1787072 | 1787382 | 311 | 1787258 | 754 | 9.35712 | 1.25926 | 8.27978 | IP_LBN_vs_In_LBN_peak_202 | EDL933_RS09270:Promoter | EDL933_RS09270:NZ_CP008957.1:1785316-1786945:-:-281 | EDL933_RS09270(EDL933_RS09270) |
| NZ_CP008957.1 | 610119 | 610414 | 296 | 610223 | 754 | 9.35712 | 1.25926 | 8.27978 | IP_LBN_vs_In_LBN_peak_75 | EDL933_RS02840:CDS;EDL933_RS02830:Promoter;EDL933_RS02825:Promoter | EDL933_RS02840:NZ_CP008957.1:610070-610850:+:196 | EDL933_RS02840(fetB) |
| NZ_CP008957.1 | 3421555 | 3421791 | 237 | 3421642 | 753 | 9.25419 | 1.25759 | 8.17849 | IP_LBN_vs_In_LBN_peak_305 | EDL933_RS17915:CDS | EDL933_RS17915:NZ_CP008957.1:3421108-3421855:-:182 | EDL933_RS17915(hda) |
| NZ_CP008957.1 | 1157723 | 1158288 | 566 | 1157859 | 753 | 9.25419 | 1.25759 | 8.17849 | IP_LBN_vs_In_LBN_peak_138 | EDL933_RS05690:Promoter;EDL933_RS05695:Promoter | EDL933_RS05690:NZ_CP008957.1:1157871-1159164:+:134 | EDL933_RS05690(serS) |
| NZ_CP008957.1 | 552374 | 552657 | 284 | 552543 | 753 | 9.25419 | 1.25759 | 8.17849 | IP_LBN_vs_In_LBN_peak_68 | EDL933_RS02655:Promoter;EDL933_RS02665:Promoter | EDL933_RS02665:NZ_CP008957.1:552600-555963:+:-85 | EDL933_RS02665(mscK) |
| NZ_CP008957.1 | 3105647 | 3106097 | 451 | 3105788 | 752 | 9.15183 | 1.25592 | 8.07776 | IP_LBN_vs_In_LBN_peak_274 | EDL933_RS16435:Promoter;EDL933_RS16440:Promoter;EDL933_RS16425:CDS | EDL933_RS16425:NZ_CP008957.1:3105765-3106050:+:106 | EDL933_RS16425(rplY) |
| NZ_CP008957.1 | 1205674 | 1205960 | 287 | 1205823 | 752 | 9.15183 | 1.25592 | 8.07776 | IP_LBN_vs_In_LBN_peak_143 | EDL933_RS05880:Promoter;EDL933_RS05870:Promoter | EDL933_RS05870:NZ_CP008957.1:1204387-1205788:-:-28 | EDL933_RS05870(asnS) |
| NZ_CP008958.1 | 57445 | 58142 | 698 | 57734 | 1629 | 18.37143 | 1.2549 | 17.19161 | IP_LBN_vs_In_LBN_peak_603 | EDL933_RS28880:Promoter | EDL933_RS28880:NZ_CP008958.1:58847-60572:+:-1054 | EDL933_RS28880(ltrA) |
| NZ_CP008957.1 | 3831877 | 3832236 | 360 | 3832107 | 846 | 10.05479 | 1.25421 | 8.96766 | IP_LBN_vs_In_LBN_peak_358 | EDL933_RS19985:Promoter;EDL933_RS19980:Promoter;EDL933_RS19990:Promoter | EDL933_RS19985:NZ_CP008957.1:3832221-3833409:+:-165 | EDL933_RS19985(ygeW) |
| NZ_CP008957.1 | 5403447 | 5404001 | 555 | 5403653 | 911 | 10.67898 | 1.25324 | 9.58325 | IP_LBN_vs_In_LBN_peak_564 | EDL933_RS27865:Promoter;EDL933_RS27855:CDS;EDL933_RS27845:Promoter | EDL933_RS27855:NZ_CP008957.1:5402865-5404182:-:458 | EDL933_RS27855(EDL933_RS27855) |
| NZ_CP008957.1 | 892725 | 893362 | 638 | 893300 | 750 | 8.94878 | 1.25259 | 7.87783 | IP_LBN_vs_In_LBN_peak_111 | EDL933_RS04185:Promoter;EDL933_RS29605:Promoter;EDL933_RS04190:Promoter | EDL933_RS04195:NZ_CP008957.1:892678-893281:+:365 | EDL933_RS04195(EDL933_RS04195) |
| NZ_CP008957.1 | 5402654 | 5403021 | 368 | 5402807 | 913 | 10.614 | 1.25193 | 9.51927 | IP_LBN_vs_In_LBN_peak_563 | EDL933_RS27845:Promoter | EDL933_RS27850:NZ_CP008957.1:5402097-5402667:+:740 | EDL933_RS27850(EDL933_RS27850) |
| NZ_CP008957.1 | 4806726 | 4807554 | 829 | 4807065 | 1094 | 12.49131 | 1.25189 | 11.37279 | IP_LBN_vs_In_LBN_peak_482 | EDL933_RS24985:Promoter;EDL933_RS24970:Promoter | EDL933_RS24975:NZ_CP008957.1:4807079-4807523:-:383 | EDL933_RS24975(mioC) |
| NZ_CP008957.1 | 3486588 | 3486908 | 321 | 3486788 | 749 | 8.8481 | 1.25092 | 7.77869 | IP_LBN_vs_In_LBN_peak_311 | EDL933_RS18195:Promoter;EDL933_RS18200:Promoter | EDL933_RS18200:NZ_CP008957.1:3485775-3486759:-:11 | EDL933_RS18200(EDL933_RS18200) |
