# Supplementary material for: Genomic Island-Encoded Histidine Kinase and Response Regulator Coordinate Mannose Utilization with Virulence in Enterohemorrhagic Escherichia coli
Source: mBio. 2023 Feb 14;14(2):e03152-22. doi: 10.1128/mbio.03152-22 (PMC10128022; doi:10.1128/mbio.03152-22)
Supplement: TABLE S5 [file mbio.03152-22-s0005.docx]

| **Table S5. Information on shared genes with LmvR ChIP peaks during bacterial growth in LB and DMEM media.** | | | | | | | | | | | | |
| --- | --- | --- | --- | --- | --- | --- | --- | --- | --- | --- | --- | --- |
| chr | start | end | length | location of peak summit | pileup reads | p value (-log10) | fold_enrichment | q value (-log10) | name | GeneFeature | GeneInside | Gene |
| NZ_CP008957.1 | 93995 | 94448 | 454 | 94258 | 541 | 12.69605 | 1.38654 | 11.0576 | IP_DMC_vs_In_DMC_peak_12 | EDL933_RS00445:Promoter;EDL933_RS00440:CDS;EDL933_RS00450:Promoter;EDL933_RS00455:Promoter | EDL933_RS00440:NZ_CP008957.1:94239-94698:+:-18 | EDL933_RS00440(mraZ) |
| NZ_CP008957.1 | 110891 | 112174 | 1284 | 111158 | 947 | 90.84211 | 2.08983 | 88.60262 | IP_DMC_vs_In_DMC_peak_14 | EDL933_RS00520:Promoter;EDL933_RS00525:Promoter;EDL933_RS00515:Promoter | EDL933_RS00515:NZ_CP008957.1:111162-112080:+:370 | EDL933_RS00515(lpxC) |
| NZ_CP008957.1 | 126392 | 133728 | 7337 | 126802 | 763 | 52.545 | 1.83795 | 50.45867 | IP_DMC_vs_In_DMC_peak_16 | EDL933_RS00600:CDS;EDL933_RS00595:Promoter;EDL933_RS00605:Promoter | EDL933_RS00610:NZ_CP008957.1:130184-132077:+:-124 | EDL933_RS00610(aceF) |
| NZ_CP008957.1 | 193066 | 193354 | 289 | 193212 | 505 | 6.99307 | 1.2693 | 5.5407 | IP_DMC_vs_In_DMC_peak_25 | EDL933_RS00880:Promoter;EDL933_RS00865:Promoter;EDL933_RS00870:Promoter;EDL933_RS00875:Promoter | EDL933_RS00875:NZ_CP008957.1:193360-194086:+:-150 | EDL933_RS00875(rpsB) |
| NZ_CP008957.1 | 226998 | 228612 | 1615 | 227488 | 1321 | 209.54994 | 2.68723 | 206.53447 | IP_DMC_vs_In_DMC_peak_29 | EDL933_RS01055:Promoter;EDL933_RS01050:Promoter;EDL933_RS01045:Promoter;EDL933_RS01030:Promoter;EDL933_RS01040:exon | EDL933_RS01040:NZ_CP008957.1:227102-228644:+:702 | EDL933_RS01040(EDL933_RS01040) |
| NZ_CP008957.1 | 229028 | 232176 | 3149 | 232061 | 1733 | 44.97107 | 1.43237 | 42.93396 | IP_DMC_vs_In_DMC_peak_30 | EDL933_RS01065:Promoter;EDL933_RS01070:Promoter;EDL933_RS01060:Promoter | EDL933_RS01060:NZ_CP008957.1:232087-232203:+:-1485 | EDL933_RS01060(rrf) |
| NZ_CP008957.1 | 271030 | 271327 | 298 | 271181 | 523 | 11.18833 | 1.36226 | 9.59004 | IP_DMC_vs_In_DMC_peak_35 | EDL933_RS01265:Promoter;EDL933_RS01250:Promoter;EDL933_RS01245:CDS | EDL933_RS01250:NZ_CP008957.1:271361-271907:+:-183 | EDL933_RS01250(EDL933_RS01250) |
| NZ_CP008957.1 | 299831 | 300149 | 319 | 300006 | 576 | 17.09209 | 1.45504 | 15.35955 | IP_DMC_vs_In_DMC_peak_38 | EDL933_RS01415:exon | EDL933_RS01415:NZ_CP008957.1:299982-300058:+:7 | EDL933_RS01415(EDL933_RS01415) |
| NZ_CP008957.1 | 301596 | 302089 | 494 | 301860 | 1234 | 224.27539 | 2.9167 | 221.20703 | IP_DMC_vs_In_DMC_peak_39 | EDL933_RS01450:Promoter;EDL933_RS01445:Promoter;EDL933_RS01425:Promoter;EDL933_RS01420:Promoter;EDL933_RS01430:Promoter;EDL933_RS01440:Promoter | EDL933_RS01430:NZ_CP008957.1:301422-301812:-:-30 | EDL933_RS01430(EDL933_RS01430) |
| NZ_CP008957.1 | 302329 | 302734 | 406 | 302510 | 567 | 14.51301 | 1.41113 | 12.83152 | IP_DMC_vs_In_DMC_peak_40 | EDL933_RS01435:CDS;EDL933_RS01420:Promoter;EDL933_RS01430:Promoter;EDL933_RS01450:Promoter;EDL933_RS01425:Promoter;EDL933_RS01445:Promoter;EDL933_RS01455:Promoter;EDL933_RS01440:Promoter | EDL933_RS01435:NZ_CP008957.1:301939-302653:-:122 | EDL933_RS01435(EDL933_RS01435) |
| NZ_CP008957.1 | 321973 | 322670 | 698 | 322387 | 489 | 6.36111 | 1.25725 | 4.93711 | IP_DMC_vs_In_DMC_peak_46 | EDL933_RS01585:CDS | EDL933_RS01585:NZ_CP008957.1:322024-322981:-:660 | EDL933_RS01585(EDL933_RS01585) |
| NZ_CP008957.1 | 379073 | 379884 | 812 | 379727 | 493 | 9.0836 | 1.326 | 7.55326 | IP_DMC_vs_In_DMC_peak_55 | EDL933_RS01855:Promoter | EDL933_RS01855:NZ_CP008957.1:379733-383717:+:-255 | EDL933_RS01855(ehaA) |
| NZ_CP008957.1 | 386265 | 386521 | 257 | 386395 | 543 | 9.83506 | 1.32474 | 8.27893 | IP_DMC_vs_In_DMC_peak_56 | EDL933_RS01880:Promoter;EDL933_RS01875:Promoter;EDL933_RS01865:Promoter;EDL933_RS01885:Promoter;EDL933_RS01870:CDS | EDL933_RS01870:NZ_CP008957.1:386011-386519:-:126 | EDL933_RS01870(EDL933_RS01870) |
| NZ_CP008957.1 | 423248 | 424284 | 1037 | 423542 | 38230 | 831.94226 | 1.39612 | 828.17468 | IP_DMC_vs_In_DMC_peak_57 | EDL933_RS02035:CDS;EDL933_RS02030:Promoter | EDL933_RS02035:NZ_CP008957.1:423203-424286:-:520 | EDL933_RS02035(lacI) |
| NZ_CP008957.1 | 502041 | 502621 | 581 | 502391 | 516 | 8.65207 | 1.30705 | 7.1372 | IP_DMC_vs_In_DMC_peak_59 | EDL933_RS02430:CDS | EDL933_RS02430:NZ_CP008957.1:501536-503108:-:777 | EDL933_RS02430(espY3) |
| NZ_CP008957.1 | 517626 | 518029 | 404 | 517860 | 566 | 19.61249 | 1.50586 | 17.83996 | IP_DMC_vs_In_DMC_peak_60 | EDL933_RS02500:Promoter;EDL933_RS02495:Promoter | EDL933_RS02500:NZ_CP008957.1:516715-517663:-:-164 | EDL933_RS02500(cyoA) |
| NZ_CP008957.1 | 545735 | 547099 | 1365 | 546750 | 752 | 57.51398 | 1.90743 | 55.40179 | IP_DMC_vs_In_DMC_peak_66 | EDL933_RS02645:CDS;EDL933_RS02630:Promoter;EDL933_RS02635:Promoter;EDL933_RS02640:Promoter | EDL933_RS02640:NZ_CP008957.1:546154-546373:-:-43 | EDL933_RS02640(hha) |
| NZ_CP008957.1 | 551662 | 551887 | 226 | 551775 | 483 | 7.4406 | 1.28823 | 5.96997 | IP_DMC_vs_In_DMC_peak_67 | EDL933_RS02660:Promoter;EDL933_RS02655:Promoter;EDL933_RS02665:Promoter;EDL933_RS02650:Promoter | EDL933_RS02660:NZ_CP008957.1:551825-552473:+:-51 | EDL933_RS02660(acrR) |
| NZ_CP008957.1 | 573035 | 574708 | 1674 | 574385 | 680 | 39.74884 | 1.73925 | 37.75188 | IP_DMC_vs_In_DMC_peak_70 | EDL933_RS02745:Promoter;EDL933_RS02750:Promoter | EDL933_RS02750:NZ_CP008957.1:573075-573870:-:-1 | EDL933_RS02750(EDL933_RS02750) |
| NZ_CP008957.1 | 699444 | 699740 | 297 | 699533 | 466 | 6.93944 | 1.28067 | 5.48894 | IP_DMC_vs_In_DMC_peak_79 | EDL933_RS03210:Promoter | EDL933_RS03210:NZ_CP008957.1:699687-700821:+:-95 | EDL933_RS03210(fepE) |
| NZ_CP008957.1 | 738215 | 738846 | 632 | 738455 | 762 | 73.35751 | 2.08843 | 71.1807 | IP_DMC_vs_In_DMC_peak_84 | EDL933_RS03405:Promoter;EDL933_RS03385:Promoter;EDL933_RS03395:CDS;EDL933_RS03410:Promoter | EDL933_RS03395:NZ_CP008957.1:738424-738634:+:106 | EDL933_RS03395(cspE) |
| NZ_CP008957.1 | 755768 | 756036 | 269 | 755965 | 516 | 13.63611 | 1.41779 | 11.97485 | IP_DMC_vs_In_DMC_peak_86 | EDL933_RS03505:Promoter;EDL933_RS03490:Promoter;EDL933_RS03495:Promoter | EDL933_RS03490:NZ_CP008957.1:753335-755918:-:16 | EDL933_RS03490(leuS) |
| NZ_CP008957.1 | 776287 | 777208 | 922 | 776923 | 684 | 50.30787 | 1.8785 | 48.23536 | IP_DMC_vs_In_DMC_peak_87 | EDL933_RS03595:Promoter;EDL933_RS03615:exon;EDL933_RS03600:Promoter;EDL933_RS03610:Promoter;EDL933_RS03605:Promoter;EDL933_RS03590:Promoter | EDL933_RS03605:NZ_CP008957.1:776694-776769:-:22 | EDL933_RS03605(EDL933_RS03605) |
| NZ_CP008957.1 | 885062 | 885402 | 341 | 885266 | 486 | 8.57723 | 1.31625 | 7.06532 | IP_DMC_vs_In_DMC_peak_97 | EDL933_RS04165:Promoter;EDL933_RS04155:Promoter;EDL933_RS04160:CDS;EDL933_RS04170:Promoter | EDL933_RS04165:NZ_CP008957.1:885530-886964:+:-298 | EDL933_RS04165(EDL933_RS04165) |
| NZ_CP008957.1 | 973021 | 973418 | 398 | 973217 | 759 | 68.63198 | 2.03493 | 66.47151 | IP_DMC_vs_In_DMC_peak_105 | EDL933_RS04670:Promoter;EDL933_RS04660:Promoter;EDL933_RS33100:Promoter;EDL933_RS04650:Promoter | EDL933_RS04660:NZ_CP008957.1:972484-972988:-:-231 | EDL933_RS04660(dps) |
| NZ_CP008957.1 | 974259 | 974679 | 421 | 974453 | 616 | 29.44974 | 1.63677 | 27.54704 | IP_DMC_vs_In_DMC_peak_106 | EDL933_RS04670:Promoter;EDL933_RS33100:CDS;EDL933_RS04660:Promoter;EDL933_RS04665:Promoter | EDL933_RS04670:NZ_CP008957.1:974526-975042:+:-57 | EDL933_RS04670(ompX) |
| NZ_CP008957.1 | 979794 | 980156 | 363 | 979980 | 627 | 32.10129 | 1.66938 | 30.17239 | IP_DMC_vs_In_DMC_peak_108 | EDL933_RS04700:Promoter;EDL933_RS04695:Promoter | EDL933_RS04700:NZ_CP008957.1:980038-981631:+:-63 | EDL933_RS04700(EDL933_RS04700) |
| NZ_CP008957.1 | 996937 | 997246 | 310 | 997015 | 482 | 7.93085 | 1.30135 | 6.44183 | IP_DMC_vs_In_DMC_peak_109 | EDL933_RS04770:Promoter | EDL933_RS04770:NZ_CP008957.1:997083-999432:+:8 | EDL933_RS04770(EDL933_RS04770) |
| NZ_CP008957.1 | 1012298 | 1012598 | 301 | 1012453 | 727 | 55.83788 | 1.90966 | 53.73385 | IP_DMC_vs_In_DMC_peak_110 | EDL933_RS04835:Promoter;EDL933_RS04845:Promoter;EDL933_RS04840:Promoter | EDL933_RS04845:NZ_CP008957.1:1012480-1013017:+:-32 | EDL933_RS04845(rcdA) |
| NZ_CP008957.1 | 1118532 | 1119287 | 756 | 1118670 | 625 | 18.24684 | 1.45187 | 16.49608 | IP_DMC_vs_In_DMC_peak_113 | EDL933_RS05465:Promoter;EDL933_RS05470:CDS;EDL933_RS05480:Promoter | EDL933_RS05470:NZ_CP008957.1:1118219-1119433:-:524 | EDL933_RS05470(EDL933_RS05470) |
| NZ_CP008957.1 | 1141396 | 1141872 | 477 | 1141554 | 664 | 23.37872 | 1.51511 | 21.54935 | IP_DMC_vs_In_DMC_peak_114 | EDL933_RS05635:Promoter;EDL933_RS05625:Promoter;EDL933_RS05620:Promoter;EDL933_RS05630:Promoter;EDL933_RS05610:CDS | EDL933_RS05610:NZ_CP008957.1:1141087-1142301:-:667 | EDL933_RS05610(EDL933_RS05610) |
| NZ_CP008957.1 | 1157652 | 1158211 | 560 | 1157838 | 529 | 15.72196 | 1.45344 | 14.01428 | IP_DMC_vs_In_DMC_peak_118 | EDL933_RS05690:Promoter;EDL933_RS05695:Promoter | EDL933_RS05690:NZ_CP008957.1:1157871-1159164:+:60 | EDL933_RS05690(serS) |
| NZ_CP008957.1 | 1178549 | 1180016 | 1468 | 1178820 | 637 | 38.04688 | 1.74961 | 36.06289 | IP_DMC_vs_In_DMC_peak_120 | EDL933_RS05775:Promoter;EDL933_RS05765:CDS;EDL933_RS05770:Promoter | EDL933_RS05770:NZ_CP008957.1:1178969-1180643:+:313 | EDL933_RS05770(rpsA) |
| NZ_CP008957.1 | 1236396 | 1237110 | 715 | 1236874 | 548 | 18.77418 | 1.5011 | 17.01477 | IP_DMC_vs_In_DMC_peak_126 | EDL933_RS06020:Promoter;EDL933_RS06005:Promoter;EDL933_RS05995:Promoter | EDL933_RS06005:NZ_CP008957.1:1235815-1236856:-:103 | EDL933_RS06005(ompA) |
| NZ_CP008957.1 | 1261025 | 1262021 | 997 | 1261885 | 776 | 78.26001 | 2.13079 | 76.06622 | IP_DMC_vs_In_DMC_peak_129 | EDL933_RS32105:Promoter;EDL933_RS06200:Promoter;EDL933_RS06185:Promoter;EDL933_RS06180:Promoter;EDL933_RS06195:Promoter | EDL933_RS06180:NZ_CP008957.1:1260641-1261415:-:-107 | EDL933_RS06180(EDL933_RS06180) |
| NZ_CP008957.1 | 1298054 | 1298423 | 370 | 1298248 | 609 | 26.19935 | 1.59043 | 24.33286 | IP_DMC_vs_In_DMC_peak_134 | EDL933_RS06450:Promoter;EDL933_RS06445:Promoter;EDL933_RS06425:Promoter;EDL933_RS06440:exon | EDL933_RS06440:NZ_CP008957.1:1298217-1298305:-:67 | EDL933_RS06440(EDL933_RS06440) |
| NZ_CP008957.1 | 1315225 | 1317411 | 2187 | 1316506 | 1070 | 194.41241 | 2.91295 | 191.48329 | IP_DMC_vs_In_DMC_peak_135 | EDL933_RS06540:Promoter;EDL933_RS29850:Promoter;EDL933_RS29845:Promoter;EDL933_RS06535:Promoter;EDL933_RS06510:Promoter;EDL933_RS06515:Promoter;EDL933_RS06525:Promoter;EDL933_RS06520:Promoter | EDL933_RS06525:NZ_CP008957.1:1316041-1316347:-:29 | EDL933_RS06525(EDL933_RS06525) |
| NZ_CP008957.1 | 1378831 | 1379346 | 516 | 1379041 | 783 | 37.83987 | 1.64678 | 35.85769 | IP_DMC_vs_In_DMC_peak_141 | EDL933_RS06980:Promoter;EDL933_RS06985:Promoter;EDL933_RS06995:Promoter;EDL933_RS06990:Promoter;EDL933_RS07000:Promoter | EDL933_RS06980:NZ_CP008957.1:1379142-1379283:+:-54 | EDL933_RS06980(EDL933_RS06980) |
| NZ_CP008957.1 | 1387860 | 1388348 | 489 | 1388118 | 1213 | 179.16187 | 2.57935 | 176.32698 | IP_DMC_vs_In_DMC_peak_142 | EDL933_RS07010:CDS | EDL933_RS07020:NZ_CP008957.1:1391478-1391823:-:3719 | EDL933_RS07020(EDL933_RS07020) |
| NZ_CP008957.1 | 1394492 | 1395051 | 560 | 1394794 | 762 | 54.00055 | 1.85629 | 51.9057 | IP_DMC_vs_In_DMC_peak_144 | EDL933_RS07040:Promoter;EDL933_RS07035:Promoter;EDL933_RS07045:Promoter;EDL933_RS29915:Promoter | EDL933_RS07045:NZ_CP008957.1:1393948-1394578:-:-193 | EDL933_RS07045(EDL933_RS07045) |
| NZ_CP008957.1 | 1441893 | 1442120 | 228 | 1442039 | 455 | 5.76066 | 1.2505 | 4.36612 | IP_DMC_vs_In_DMC_peak_150 | EDL933_RS07230:Promoter;EDL933_RS07225:CDS;EDL933_RS07245:Promoter;EDL933_RS07235:Promoter;EDL933_RS07240:Promoter | EDL933_RS07225:NZ_CP008957.1:1441896-1442265:+:110 | EDL933_RS07225(acpS) |
| NZ_CP008957.1 | 1541552 | 1542317 | 766 | 1541700 | 602 | 16.86681 | 1.43857 | 15.1385 | IP_DMC_vs_In_DMC_peak_154 | EDL933_RS07775:Promoter;EDL933_RS07770:Promoter;EDL933_RS07780:Promoter;EDL933_RS07765:Promoter;EDL933_RS07755:CDS | EDL933_RS07755:NZ_CP008957.1:1541234-1542448:-:514 | EDL933_RS07755(EDL933_RS07755) |
| NZ_CP008957.1 | 1591216 | 1591626 | 411 | 1591459 | 471 | 7.51124 | 1.29438 | 6.03756 | IP_DMC_vs_In_DMC_peak_155 | EDL933_RS08055:Promoter;EDL933_RS08065:Promoter | EDL933_RS08055:NZ_CP008957.1:1588022-1591208:-:-212 | EDL933_RS08055(rne) |
| NZ_CP008957.1 | 1593389 | 1594724 | 1336 | 1593637 | 554 | 20.11329 | 1.52199 | 18.33305 | IP_DMC_vs_In_DMC_peak_156 | EDL933_RS08085:Promoter;EDL933_RS08090:Promoter;EDL933_RS08070:Promoter;EDL933_RS08080:Promoter;EDL933_RS08075:CDS | EDL933_RS08080:NZ_CP008957.1:1594196-1594370:+:-140 | EDL933_RS08080(rpmF) |
| NZ_CP008957.1 | 1598097 | 1598990 | 894 | 1598421 | 561 | 21.43022 | 1.54119 | 19.62999 | IP_DMC_vs_In_DMC_peak_157 | EDL933_RS08115:Promoter;EDL933_RS08110:Promoter;EDL933_RS08105:Promoter | EDL933_RS08105:NZ_CP008957.1:1598444-1598681:+:99 | EDL933_RS08105(acpP) |
| NZ_CP008957.1 | 1960868 | 1961279 | 412 | 1961143 | 861 | 108.29508 | 2.36389 | 105.96835 | IP_DMC_vs_In_DMC_peak_175 | EDL933_RS10360:Promoter;EDL933_RS10350:Promoter | EDL933_RS10350:NZ_CP008957.1:1961192-1962644:+:-119 | EDL933_RS10350(uxaB) |
| NZ_CP008957.1 | 2284326 | 2285267 | 942 | 2285088 | 503 | 11.68951 | 1.38214 | 10.07654 | IP_DMC_vs_In_DMC_peak_178 | EDL933_RS11965:Promoter;EDL933_RS11960:Promoter;EDL933_RS11950:Promoter;EDL933_RS11955:CDS | EDL933_RS11950:NZ_CP008957.1:2284127-2284718:-:-78 | EDL933_RS11950(espM1) |
| NZ_CP008957.1 | 2440575 | 2440874 | 300 | 2440751 | 508 | 12.42152 | 1.39585 | 10.7894 | IP_DMC_vs_In_DMC_peak_185 | EDL933_RS12800:Promoter;EDL933_RS12815:Promoter;EDL933_RS12820:Promoter;EDL933_RS12810:exon | EDL933_RS12810:NZ_CP008957.1:2440687-2440764:+:37 | EDL933_RS12810(EDL933_RS12810) |
| NZ_CP008957.1 | 2451384 | 2451863 | 480 | 2451640 | 685 | 50.58421 | 1.88124 | 48.50996 | IP_DMC_vs_In_DMC_peak_186 | EDL933_RS12865:CDS | EDL933_RS12865:NZ_CP008957.1:2451593-2451830:+:30 | EDL933_RS12865(lpp) |
| NZ_CP008957.1 | 2493263 | 2495224 | 1962 | 2494157 | 672 | 47.04117 | 1.84559 | 44.98894 | IP_DMC_vs_In_DMC_peak_187 | EDL933_RS33520:Promoter;EDL933_RS13075:CDS;EDL933_RS13070:Promoter;EDL933_RS13060:Promoter | EDL933_RS13075:NZ_CP008957.1:2493975-2494173:-:-70 | EDL933_RS13075(rpmI) |
| NZ_CP008957.1 | 2654915 | 2655241 | 327 | 2655150 | 507 | 12.27344 | 1.39311 | 10.645 | IP_DMC_vs_In_DMC_peak_195 | EDL933_RS13935:CDS;EDL933_RS13925:Promoter;EDL933_RS13930:Promoter;EDL933_RS13945:Promoter | EDL933_RS13930:NZ_CP008957.1:2653423-2654524:-:-553 | EDL933_RS13930(EDL933_RS13930) |
| NZ_CP008957.1 | 2708387 | 2709013 | 627 | 2708597 | 928 | 133.18369 | 2.53353 | 130.64938 | IP_DMC_vs_In_DMC_peak_199 | EDL933_RS33550:Promoter;EDL933_RS14255:exon;EDL933_RS33555:Promoter;EDL933_RS14250:Promoter | EDL933_RS14260:NZ_CP008957.1:2708647-2708721:-:21 | EDL933_RS14260(EDL933_RS14260) |
| NZ_CP008957.1 | 2821868 | 2822103 | 236 | 2822048 | 459 | 6.17663 | 1.26147 | 4.76057 | IP_DMC_vs_In_DMC_peak_204 | EDL933_RS14960:Promoter;EDL933_RS14945:Promoter | EDL933_RS14945:NZ_CP008957.1:2821959-2822035:-:50 | EDL933_RS14945(EDL933_RS14945) |
| NZ_CP008957.1 | 2823631 | 2823989 | 359 | 2823817 | 560 | 19.99592 | 1.51625 | 18.21756 | IP_DMC_vs_In_DMC_peak_205 | EDL933_RS29235:Promoter;EDL933_RS14960:exon;EDL933_RS14945:Promoter | EDL933_RS14960:NZ_CP008957.1:2823783-2823859:+:26 | EDL933_RS14960(EDL933_RS14960) |
| NZ_CP008957.1 | 2861682 | 2863600 | 1919 | 2861904 | 497 | 10.83891 | 1.36568 | 9.25058 | IP_DMC_vs_In_DMC_peak_208 | EDL933_RS15165:Promoter;EDL933_RS15170:Promoter;EDL933_RS15175:CDS | EDL933_RS15175:NZ_CP008957.1:2861839-2862505:-:-135 | EDL933_RS15175(perB) |
| NZ_CP008957.1 | 2877980 | 2878246 | 267 | 2878080 | 497 | 10.83891 | 1.36568 | 9.25058 | IP_DMC_vs_In_DMC_peak_213 | EDL933_RS15245:Promoter;EDL933_RS15240:Promoter | EDL933_RS15245:NZ_CP008957.1:2877036-2878032:-:-80 | EDL933_RS15245(EDL933_RS15245) |
| NZ_CP008957.1 | 2955651 | 2955972 | 322 | 2955837 | 496 | 10.70011 | 1.36294 | 9.11587 | IP_DMC_vs_In_DMC_peak_217 | EDL933_RS15590:CDS;EDL933_RS15580:Promoter;EDL933_RS15585:Promoter | EDL933_RS15585:NZ_CP008957.1:2954968-2955643:-:-168 | EDL933_RS15585(EDL933_RS15585) |
| NZ_CP008957.1 | 3012664 | 3014410 | 1747 | 3014185 | 602 | 29.88061 | 1.65363 | 27.97332 | IP_DMC_vs_In_DMC_peak_221 | EDL933_RS15870:Promoter;EDL933_RS15875:Promoter;EDL933_RS15880:Promoter | EDL933_RS15875:NZ_CP008957.1:3012808-3013078:-:-458 | EDL933_RS15875(EDL933_RS15875) |
| NZ_CP008957.1 | 3020932 | 3021431 | 500 | 3021171 | 556 | 11.97326 | 1.36541 | 10.35322 | IP_DMC_vs_In_DMC_peak_222 | EDL933_RS15970:CDS;EDL933_RS15965:Promoter;EDL933_RS15960:Promoter;EDL933_RS15955:Promoter;EDL933_RS15980:Promoter;EDL933_RS15950:Promoter;EDL933_RS15975:Promoter | EDL933_RS15970:NZ_CP008957.1:3021068-3021716:+:113 | EDL933_RS15970(EDL933_RS15970) |
| NZ_CP008957.1 | 3089245 | 3089540 | 296 | 3089394 | 554 | 20.11329 | 1.52199 | 18.33305 | IP_DMC_vs_In_DMC_peak_228 | EDL933_RS16350:Promoter;EDL933_RS16365:Promoter;EDL933_RS16360:Promoter | EDL933_RS16360:NZ_CP008957.1:3089492-3090959:+:-100 | EDL933_RS16360(EDL933_RS16360) |
| NZ_CP008957.1 | 3092903 | 3093323 | 421 | 3093111 | 692 | 45.33644 | 1.80501 | 43.2967 | IP_DMC_vs_In_DMC_peak_229 | EDL933_RS16380:Promoter;EDL933_RS16375:Promoter | EDL933_RS16375:NZ_CP008957.1:3093227-3093794:+:-114 | EDL933_RS16375(mepS) |
| NZ_CP008957.1 | 3105611 | 3105937 | 327 | 3105752 | 467 | 7.05201 | 1.28341 | 5.59682 | IP_DMC_vs_In_DMC_peak_231 | EDL933_RS16435:Promoter;EDL933_RS16440:Promoter;EDL933_RS16425:Promoter | EDL933_RS16425:NZ_CP008957.1:3105765-3106050:+:8 | EDL933_RS16425(rplY) |
| NZ_CP008957.1 | 3109269 | 3109659 | 391 | 3109472 | 619 | 33.74084 | 1.70025 | 31.79541 | IP_DMC_vs_In_DMC_peak_233 | EDL933_RS16445:exon | EDL933_RS16445:NZ_CP008957.1:3109459-3109536:+:4 | EDL933_RS16445(EDL933_RS16445) |
| NZ_CP008957.1 | 3132940 | 3134712 | 1773 | 3134031 | 750 | 69.8573 | 2.05949 | 67.69212 | IP_DMC_vs_In_DMC_peak_235 | EDL933_RS16575:CDS;EDL933_RS16570:Promoter;EDL933_RS16590:Promoter | EDL933_RS16575:NZ_CP008957.1:3132951-3134055:-:229 | EDL933_RS16575(ompC) |
| NZ_CP008957.1 | 3226258 | 3226751 | 494 | 3226515 | 591 | 24.25627 | 1.57045 | 22.41497 | IP_DMC_vs_In_DMC_peak_244 | EDL933_RS16935:Promoter;EDL933_RS16960:Promoter;EDL933_RS16950:Promoter;EDL933_RS16965:Promoter;EDL933_RS16940:Promoter;EDL933_RS16945:Promoter | EDL933_RS16960:NZ_CP008957.1:3226645-3227848:+:-141 | EDL933_RS16960(ackA) |
| NZ_CP008957.1 | 3279772 | 3280152 | 381 | 3279973 | 743 | 67.661 | 2.0403 | 65.50443 | IP_DMC_vs_In_DMC_peak_246 | EDL933_RS17220:Promoter;EDL933_RS17205:Promoter;EDL933_RS17215:exon | EDL933_RS17215:NZ_CP008957.1:3279912-3279987:+:49 | EDL933_RS17215(EDL933_RS17215) |
| NZ_CP008957.1 | 3293630 | 3293985 | 356 | 3293765 | 536 | 11.46975 | 1.36295 | 9.86347 | IP_DMC_vs_In_DMC_peak_248 | EDL933_RS17290:Promoter;EDL933_RS17285:CDS;EDL933_RS17295:Promoter | EDL933_RS17285:NZ_CP008957.1:3292869-3293928:-:121 | EDL933_RS17285(EDL933_RS17285) |
| NZ_CP008957.1 | 3342098 | 3342694 | 597 | 3342547 | 540 | 12.15772 | 1.37581 | 10.53243 | IP_DMC_vs_In_DMC_peak_258 | EDL933_RS17530:Promoter;EDL933_RS17525:Promoter;EDL933_RS17540:Promoter;EDL933_RS17535:Promoter | EDL933_RS17530:NZ_CP008957.1:3341516-3342515:-:119 | EDL933_RS17530(zipA) |
| NZ_CP008957.1 | 3432001 | 3432730 | 730 | 3432301 | 1061 | 192.73663 | 2.91236 | 189.81656 | IP_DMC_vs_In_DMC_peak_265 | EDL933_RS34045:CDS;EDL933_RS17960:Promoter;EDL933_RS17970:Promoter;EDL933_RS17950:Promoter;EDL933_RS17965:Promoter | EDL933_RS17960:NZ_CP008957.1:3432322-3432514:+:43 | EDL933_RS17960(EDL933_RS17960) |
| NZ_CP008957.1 | 3470602 | 3470926 | 325 | 3470694 | 523 | 6.45881 | 1.2501 | 5.03006 | IP_DMC_vs_In_DMC_peak_272 | EDL933_RS18130:Promoter;EDL933_RS18110:Promoter;EDL933_RS18120:CDS;EDL933_RS18115:Promoter;EDL933_RS18125:Promoter | EDL933_RS18120:NZ_CP008957.1:3469977-3470715:-:-48 | EDL933_RS18120(trmJ) |
| NZ_CP008957.1 | 3498251 | 3498771 | 521 | 3498569 | 897 | 108.09826 | 2.31561 | 105.77319 | IP_DMC_vs_In_DMC_peak_273 | EDL933_RS18240:Promoter;EDL933_RS18235:Promoter | EDL933_RS18240:NZ_CP008957.1:3496927-3498355:-:-155 | EDL933_RS18240(qseE) |
| NZ_CP008957.1 | 3515756 | 3517480 | 1725 | 3517275 | 509 | 10.8154 | 1.35991 | 9.22832 | IP_DMC_vs_In_DMC_peak_275 | EDL933_RS18345:Promoter;EDL933_RS33700:CDS;EDL933_RS18325:Promoter;EDL933_RS18330:Promoter;EDL933_RS18335:CDS | EDL933_RS18330:NZ_CP008957.1:3516029-3516668:-:50 | EDL933_RS18330(rseA) |
| NZ_CP008957.1 | 3533174 | 3536294 | 3121 | 3533285 | 1807 | 28.92702 | 1.31812 | 27.03027 | IP_DMC_vs_In_DMC_peak_276 | EDL933_RS18415:Promoter;EDL933_RS18420:Promoter | EDL933_RS18420:NZ_CP008957.1:3533156-3533272:-:-1461 | EDL933_RS18420(rrf) |
| NZ_CP008957.1 | 3536661 | 3538280 | 1620 | 3537799 | 1306 | 204.25043 | 2.66513 | 201.26285 | IP_DMC_vs_In_DMC_peak_277 | EDL933_RS18435:exon;EDL933_RS18430:Promoter;EDL933_RS18425:Promoter | EDL933_RS18435:NZ_CP008957.1:3536623-3538165:-:695 | EDL933_RS18435(EDL933_RS18435) |
| NZ_CP008957.1 | 3542905 | 3543200 | 296 | 3543043 | 492 | 9.86388 | 1.34513 | 8.30706 | IP_DMC_vs_In_DMC_peak_278 | EDL933_RS18450:Promoter;EDL933_RS18470:Promoter;EDL933_RS33705:Promoter;EDL933_RS18465:Promoter;EDL933_RS18445:Promoter;EDL933_RS18440:Promoter;EDL933_RS18455:Promoter | EDL933_RS18450:NZ_CP008957.1:3542038-3543019:-:-33 | EDL933_RS18450(rluD) |
| NZ_CP008957.1 | 3543730 | 3544797 | 1068 | 3544168 | 1168 | 200.00864 | 2.80964 | 197.04712 | IP_DMC_vs_In_DMC_peak_279 | EDL933_RS18450:Promoter;EDL933_RS18470:Promoter;EDL933_RS18465:CDS;EDL933_RS33705:Promoter | EDL933_RS18465:NZ_CP008957.1:3544161-3544503:+:102 | EDL933_RS18465(raiA) |
| NZ_CP008957.1 | 3548807 | 3549102 | 296 | 3548956 | 498 | 10.15319 | 1.34941 | 8.58622 | IP_DMC_vs_In_DMC_peak_280 | EDL933_RS18475:Promoter;EDL933_RS18500:Promoter;EDL933_RS18490:CDS;EDL933_RS18480:Promoter;EDL933_RS18495:Promoter | EDL933_RS18490:NZ_CP008957.1:3548882-3549401:+:72 | EDL933_RS18490(EDL933_RS18490) |
| NZ_CP008957.1 | 3552783 | 3553509 | 727 | 3553243 | 566 | 21.89859 | 1.54626 | 20.0914 | IP_DMC_vs_In_DMC_peak_282 | EDL933_RS18520:Promoter;EDL933_RS18530:Promoter;EDL933_RS18515:Promoter;EDL933_RS18505:Promoter;EDL933_RS18510:Promoter | EDL933_RS18520:NZ_CP008957.1:3552945-3553194:-:48 | EDL933_RS18520(rpsP) |
| NZ_CP008957.1 | 3626488 | 3626789 | 302 | 3626648 | 534 | 15.36179 | 1.44365 | 13.66159 | IP_DMC_vs_In_DMC_peak_303 | EDL933_RS18920:Promoter | EDL933_RS18920:NZ_CP008957.1:3625996-3626512:-:-126 | EDL933_RS18920(luxS) |
| NZ_CP008957.1 | 3674619 | 3676503 | 1885 | 3676140 | 889 | 111.14284 | 2.35752 | 108.79568 | IP_DMC_vs_In_DMC_peak_305 | EDL933_RS19195:Promoter;EDL933_RS19200:CDS | EDL933_RS19195:NZ_CP008957.1:3674707-3675700:-:139 | EDL933_RS19195(rpoS) |
| NZ_CP008957.1 | 3719509 | 3719901 | 393 | 3719718 | 584 | 19.3199 | 1.49064 | 17.55195 | IP_DMC_vs_In_DMC_peak_308 | EDL933_RS19410:Promoter;EDL933_RS19415:Promoter | EDL933_RS19415:NZ_CP008957.1:3718053-3719691:-:-13 | EDL933_RS19415(pyrG) |
| NZ_CP008957.1 | 3734035 | 3735257 | 1223 | 3734400 | 2002 | 630.57062 | 4.46895 | 627.00806 | IP_DMC_vs_In_DMC_peak_309 | EDL933_RS19465:Promoter;EDL933_RS19495:Promoter;EDL933_RS19475:Promoter;EDL933_RS19470:Promoter;EDL933_RS19490:Promoter | EDL933_RS19475:NZ_CP008957.1:3733809-3734139:-:-506 | EDL933_RS19475(EDL933_RS19475) |
| NZ_CP008957.1 | 3875302 | 3875711 | 410 | 3875481 | 558 | 16.64943 | 1.45599 | 14.92481 | IP_DMC_vs_In_DMC_peak_325 | EDL933_RS20155:Promoter;EDL933_RS20150:Promoter;EDL933_RS33755:Promoter;EDL933_RS20160:Promoter | EDL933_RS20160:NZ_CP008957.1:3874326-3875421:-:-85 | EDL933_RS20160(gcvT) |
| NZ_CP008957.1 | 4018052 | 4018526 | 475 | 4018247 | 675 | 36.92329 | 1.70402 | 34.94954 | IP_DMC_vs_In_DMC_peak_338 | EDL933_RS20945:Promoter;EDL933_RS20950:Promoter;EDL933_RS34015:Promoter;EDL933_RS20965:Promoter;EDL933_RS20960:Promoter | EDL933_RS20945:NZ_CP008957.1:4017533-4018187:-:-101 | EDL933_RS20945(ribB) |
| NZ_CP008957.1 | 4037709 | 4038429 | 721 | 4037981 | 833 | 68.06024 | 1.9573 | 65.90206 | IP_DMC_vs_In_DMC_peak_340 | EDL933_RS21055:Promoter;EDL933_RS21050:CDS;EDL933_RS21045:Promoter | EDL933_RS21050:NZ_CP008957.1:4037938-4038154:+:130 | EDL933_RS21050(rpsU) |
| NZ_CP008957.1 | 4042620 | 4042870 | 251 | 4042780 | 506 | 8.38451 | 1.3041 | 6.87919 | IP_DMC_vs_In_DMC_peak_341 | EDL933_RS21070:exon;EDL933_RS21080:Promoter;EDL933_RS21065:Promoter | EDL933_RS21070:NZ_CP008957.1:4042755-4042831:+:-10 | EDL933_RS21070(EDL933_RS21070) |
| NZ_CP008957.1 | 4071695 | 4072280 | 586 | 4072064 | 847 | 75.09807 | 2.0212 | 72.91504 | IP_DMC_vs_In_DMC_peak_343 | EDL933_RS21200:Promoter;EDL933_RS21185:Promoter;EDL933_RS21195:Promoter;EDL933_RS21190:Promoter | EDL933_RS21190:NZ_CP008957.1:4070587-4072000:-:13 | EDL933_RS21190(uxaC) |
| NZ_CP008957.1 | 4141276 | 4142028 | 753 | 4141918 | 551 | 10.86698 | 1.34422 | 9.27796 | IP_DMC_vs_In_DMC_peak_350 | EDL933_RS21570:Promoter;EDL933_RS21565:Promoter | EDL933_RS21570:NZ_CP008957.1:4141538-4141808:-:156 | EDL933_RS21570(rpsO) |
| NZ_CP008957.1 | 4148028 | 4148748 | 721 | 4148393 | 1773 | 522.64716 | 4.20504 | 519.21252 | IP_DMC_vs_In_DMC_peak_351 | EDL933_RS21600:exon;EDL933_RS21605:Promoter;EDL933_RS21595:Promoter;EDL933_RS21590:Promoter | EDL933_RS21600:NZ_CP008957.1:4148337-4148414:-:26 | EDL933_RS21600(EDL933_RS21600) |
| NZ_CP008957.1 | 4158743 | 4159157 | 415 | 4158943 | 637 | 27.85197 | 1.59828 | 25.96717 | IP_DMC_vs_In_DMC_peak_354 | EDL933_RS21650:Promoter;EDL933_RS21640:Promoter;EDL933_RS21655:Promoter;EDL933_RS21635:Promoter | EDL933_RS21650:NZ_CP008957.1:4158363-4158840:-:-109 | EDL933_RS21650(greA) |
| NZ_CP008957.1 | 4162606 | 4163891 | 1286 | 4163437 | 696 | 41.84352 | 1.75525 | 39.83037 | IP_DMC_vs_In_DMC_peak_355 | EDL933_RS21685:Promoter;EDL933_RS21680:Promoter;EDL933_RS21675:Promoter;EDL933_RS21660:Promoter;EDL933_RS21670:Promoter;EDL933_RS21665:Promoter | EDL933_RS21670:NZ_CP008957.1:4162840-4163098:-:-150 | EDL933_RS21670(rpmA) |
| NZ_CP008957.1 | 4246674 | 4248737 | 2064 | 4247030 | 1124 | 54.58826 | 1.65497 | 52.49041 | IP_DMC_vs_In_DMC_peak_363 | EDL933_RS22100:Promoter;EDL933_RS22095:Promoter;EDL933_RS22105:exon;EDL933_RS22090:Promoter | EDL933_RS22100:NZ_CP008957.1:4246301-4246417:-:-1288 | EDL933_RS22100(rrf) |
| NZ_CP008957.1 | 4250493 | 4251500 | 1008 | 4251027 | 1286 | 158.20932 | 2.34588 | 155.50696 | IP_DMC_vs_In_DMC_peak_366 | EDL933_RS22120:exon;EDL933_RS22110:Promoter;EDL933_RS22115:Promoter;EDL933_RS22125:Promoter;EDL933_RS22105:Promoter | EDL933_RS22120:NZ_CP008957.1:4249860-4251402:-:406 | EDL933_RS22120(EDL933_RS22120) |
| NZ_CP008957.1 | 4254217 | 4255239 | 1023 | 4254408 | 604 | 17.26302 | 1.44463 | 15.52768 | IP_DMC_vs_In_DMC_peak_367 | EDL933_RS22160:Promoter;EDL933_RS22130:Promoter;EDL933_RS22135:Promoter;EDL933_RS22145:CDS;EDL933_RS22140:Promoter | EDL933_RS22145:NZ_CP008957.1:4254058-4254601:-:-126 | EDL933_RS22145(EDL933_RS22145) |
| NZ_CP008957.1 | 4274078 | 4277675 | 3598 | 4277370 | 1111 | 134.42427 | 2.32439 | 131.87798 | IP_DMC_vs_In_DMC_peak_370 | EDL933_RS22350:Promoter;EDL933_RS22330:Promoter;EDL933_RS22335:Promoter;EDL933_RS22355:CDS | EDL933_RS22335:NZ_CP008957.1:4275597-4275909:-:33 | EDL933_RS22335(rpsJ) |
| NZ_CP008957.1 | 4279917 | 4280280 | 364 | 4280101 | 481 | 6.34468 | 1.25925 | 4.92131 | IP_DMC_vs_In_DMC_peak_372 | EDL933_RS22365:CDS;EDL933_RS22360:Promoter | EDL933_RS22365:NZ_CP008957.1:4278932-4281047:-:949 | EDL933_RS22365(fusA) |
| NZ_CP008957.1 | 4293448 | 4293766 | 319 | 4293594 | 522 | 9.12451 | 1.31608 | 7.59224 | IP_DMC_vs_In_DMC_peak_376 | EDL933_RS22460:Promoter;EDL933_RS22465:Promoter;EDL933_RS22450:Promoter | EDL933_RS22460:NZ_CP008957.1:4293652-4294285:+:-45 | EDL933_RS22460(crp) |
| NZ_CP008957.1 | 4325250 | 4326495 | 1246 | 4325979 | 683 | 36.24003 | 1.68886 | 34.27205 | IP_DMC_vs_In_DMC_peak_379 | EDL933_RS22620:Promoter;EDL933_RS22625:Promoter | EDL933_RS22620:NZ_CP008957.1:4325409-4325970:-:98 | EDL933_RS22620(nudE) |
| NZ_CP008957.1 | 4389735 | 4390277 | 543 | 4390110 | 512 | 7.76469 | 1.28675 | 6.28157 | IP_DMC_vs_In_DMC_peak_383 | intergenic | EDL933_RS22890:NZ_CP008957.1:4388718-4389630:+:1287 | EDL933_RS22890(EDL933_RS22890) |
| NZ_CP008957.1 | 4407351 | 4408069 | 719 | 4407746 | 776 | 43.79412 | 1.72317 | 41.7668 | IP_DMC_vs_In_DMC_peak_386 | EDL933_RS22980:Promoter;EDL933_RS22985:Promoter | EDL933_RS22985:NZ_CP008957.1:4406799-4407654:-:-55 | EDL933_RS22985(rpoH) |
| NZ_CP008957.1 | 4422385 | 4422809 | 425 | 4422578 | 819 | 65.72841 | 1.9437 | 63.58034 | IP_DMC_vs_In_DMC_peak_387 | EDL933_RS23085:Promoter;EDL933_RS23075:Promoter;EDL933_RS23090:Promoter;EDL933_RS23080:Promoter;EDL933_RS23070:CDS | EDL933_RS23070:NZ_CP008957.1:4422443-4423265:+:153 | EDL933_RS23070(EDL933_RS23070) |
| NZ_CP008957.1 | 4456658 | 4457271 | 614 | 4456881 | 577 | 11.50186 | 1.34781 | 9.89491 | IP_DMC_vs_In_DMC_peak_391 | EDL933_RS23260:Promoter;EDL933_RS23245:Promoter | EDL933_RS23260:NZ_CP008957.1:4456946-4457999:+:18 | EDL933_RS23260(EDL933_RS23260) |
| NZ_CP008957.1 | 4469944 | 4470402 | 459 | 4470248 | 584 | 14.76813 | 1.40846 | 13.08085 | IP_DMC_vs_In_DMC_peak_392 | EDL933_RS23335:Promoter;EDL933_RS23330:Promoter;EDL933_RS23315:Promoter;EDL933_RS23325:Promoter | EDL933_RS23325:NZ_CP008957.1:4470467-4470821:+:-294 | EDL933_RS23325(arsR) |
| NZ_CP008957.1 | 4485375 | 4488963 | 3589 | 4487651 | 1049 | 132.53883 | 2.37344 | 130.01038 | IP_DMC_vs_In_DMC_peak_397 | EDL933_RS23430:Promoter;EDL933_RS23410:Promoter;EDL933_RS23405:Promoter;EDL933_RS23420:Promoter;EDL933_RS23400:Promoter | EDL933_RS23415:NZ_CP008957.1:4486753-4487326:+:415 | EDL933_RS23415(hdeD) |
| NZ_CP008957.1 | 4494204 | 4494968 | 765 | 4494666 | 1168 | 184.97678 | 2.68234 | 182.10835 | IP_DMC_vs_In_DMC_peak_398 | EDL933_RS23440:Promoter | EDL933_RS23440:NZ_CP008957.1:4493648-4494377:-:-208 | EDL933_RS23440(gadW) |
| NZ_CP008957.1 | 4514209 | 4514511 | 303 | 4514363 | 747 | 50.49156 | 1.8258 | 48.41805 | IP_DMC_vs_In_DMC_peak_400 | EDL933_RS23515:CDS;EDL933_RS23510:Promoter | EDL933_RS23515:NZ_CP008957.1:4513391-4515380:-:1020 | EDL933_RS23515(hmsP) |
| NZ_CP008957.1 | 4526384 | 4526663 | 280 | 4526538 | 544 | 8.00408 | 1.28251 | 6.51215 | IP_DMC_vs_In_DMC_peak_401 | EDL933_RS23560:CDS;EDL933_RS23570:Promoter;EDL933_RS23555:Promoter;EDL933_RS23565:Promoter;EDL933_RS23545:Promoter;EDL933_RS23540:Promoter | EDL933_RS23560:NZ_CP008957.1:4526227-4527799:+:296 | EDL933_RS23560(bcsE) |
| NZ_CP008957.1 | 4537901 | 4538240 | 340 | 4538088 | 584 | 11.47254 | 1.3447 | 9.86622 | IP_DMC_vs_In_DMC_peak_404 | EDL933_RS23625:exon;EDL933_RS23615:Promoter | EDL933_RS23625:NZ_CP008957.1:4538062-4538139:-:69 | EDL933_RS23625(EDL933_RS23625) |
| NZ_CP008957.1 | 4597647 | 4598546 | 900 | 4598016 | 569 | 12.26275 | 1.36616 | 10.63521 | IP_DMC_vs_In_DMC_peak_408 | EDL933_RS23880:CDS | EDL933_RS23880:NZ_CP008957.1:4597916-4598252:+:180 | EDL933_RS23880(EDL933_RS23880) |
| NZ_CP008957.1 | 4627986 | 4628347 | 362 | 4628200 | 555 | 10.27665 | 1.33013 | 8.70595 | IP_DMC_vs_In_DMC_peak_409 | EDL933_RS24005:Promoter;EDL933_RS24010:Promoter;EDL933_RS24015:Promoter;EDL933_RS24000:Promoter | EDL933_RS24005:NZ_CP008957.1:4627282-4628140:-:-26 | EDL933_RS24005(yibB) |
| NZ_CP008957.1 | 4642724 | 4643505 | 782 | 4643307 | 858 | 65.75845 | 1.91158 | 63.61021 | IP_DMC_vs_In_DMC_peak_415 | EDL933_RS24095:Promoter;EDL933_RS24075:Promoter;EDL933_RS24080:Promoter;EDL933_RS24085:Promoter | EDL933_RS24085:NZ_CP008957.1:4642920-4643157:-:43 | EDL933_RS24085(rpmB) |
| NZ_CP008957.1 | 4677640 | 4678187 | 548 | 4677900 | 549 | 10.84109 | 1.34442 | 9.25268 | IP_DMC_vs_In_DMC_peak_420 | EDL933_RS24270:Promoter;EDL933_RS24275:CDS | EDL933_RS24275:NZ_CP008957.1:4677711-4677990:-:77 | EDL933_RS24275(escG) |
| NZ_CP008957.1 | 4681455 | 4684512 | 3058 | 4684013 | 646 | 19.11296 | 1.45671 | 17.34806 | IP_DMC_vs_In_DMC_peak_422 | EDL933_RS24305:Promoter | EDL933_RS24310:NZ_CP008957.1:4682597-4683818:+:386 | EDL933_RS24310(escD) |
| NZ_CP008957.1 | 4690948 | 4692545 | 1598 | 4691643 | 586 | 10.65914 | 1.32751 | 9.07654 | IP_DMC_vs_In_DMC_peak_424 | EDL933_RS24330:Promoter;EDL933_RS24340:Promoter | EDL933_RS24340:NZ_CP008957.1:4691111-4691618:-:-128 | EDL933_RS24340(espH) |
| NZ_CP008957.1 | 4762559 | 4764328 | 1770 | 4763404 | 561 | 9.57594 | 1.31287 | 8.02864 | IP_DMC_vs_In_DMC_peak_438 | EDL933_RS24780:Promoter;EDL933_RS24775:Promoter;EDL933_RS24770:CDS | EDL933_RS24770:NZ_CP008957.1:4762198-4764368:-:925 | EDL933_RS24770(EDL933_RS24770) |
| NZ_CP008957.1 | 4767752 | 4767962 | 211 | 4767870 | 584 | 9.82797 | 1.31087 | 8.27201 | IP_DMC_vs_In_DMC_peak_439 | EDL933_RS24790:Promoter;EDL933_RS24795:Promoter | EDL933_RS24790:NZ_CP008957.1:4767947-4769123:+:-90 | EDL933_RS24790(mdtL) |
| NZ_CP008957.1 | 4775851 | 4778337 | 2487 | 4776473 | 578 | 13.06645 | 1.37849 | 11.41884 | IP_DMC_vs_In_DMC_peak_443 | EDL933_RS24825:Promoter;EDL933_RS24820:Promoter | EDL933_RS24825:NZ_CP008957.1:4776618-4777290:+:475 | EDL933_RS24825(EDL933_RS24825) |
| NZ_CP008957.1 | 4799941 | 4800185 | 245 | 4800030 | 562 | 10.08617 | 1.32359 | 8.52166 | IP_DMC_vs_In_DMC_peak_448 | EDL933_RS24935:CDS;EDL933_RS24925:Promoter;EDL933_RS24930:Promoter | EDL933_RS24930:NZ_CP008957.1:4798469-4799333:-:-729 | EDL933_RS24930(atpG) |
| NZ_CP008957.1 | 4806739 | 4807622 | 884 | 4807514 | 647 | 15.85458 | 1.40216 | 14.14471 | IP_DMC_vs_In_DMC_peak_453 | EDL933_RS24975:CDS;EDL933_RS24985:Promoter;EDL933_RS24970:Promoter | EDL933_RS24975:NZ_CP008957.1:4807079-4807523:-:343 | EDL933_RS24975(mioC) |
| NZ_CP008957.1 | 4822767 | 4824379 | 1613 | 4823082 | 1334 | 205.54362 | 2.64293 | 202.54781 | IP_DMC_vs_In_DMC_peak_454 | EDL933_RS25045:exon;EDL933_RS25040:Promoter;EDL933_RS25055:Promoter;EDL933_RS25050:Promoter;EDL933_RS25035:Promoter | EDL933_RS25045:NZ_CP008957.1:4822875-4824417:+:697 | EDL933_RS25045(EDL933_RS25045) |
| NZ_CP008957.1 | 4824730 | 4828262 | 3533 | 4827757 | 1812 | 53.87972 | 1.47313 | 51.78562 | IP_DMC_vs_In_DMC_peak_455 | EDL933_RS25070:Promoter;EDL933_RS25060:Promoter;EDL933_RS25065:Promoter;EDL933_RS25080:Promoter | EDL933_RS25060:NZ_CP008957.1:4827770-4827886:+:-1274 | EDL933_RS25060(rrf) |
| NZ_CP008957.1 | 4831184 | 4831659 | 476 | 4831345 | 970 | 95.34279 | 2.11197 | 93.08466 | IP_DMC_vs_In_DMC_peak_456 | EDL933_RS25095:Promoter;EDL933_RS25090:Promoter;EDL933_RS25085:Promoter;EDL933_RS25100:Promoter;EDL933_RS33875:Promoter | EDL933_RS25090:NZ_CP008957.1:4831390-4831489:+:31 | EDL933_RS25090(ilvL) |
| NZ_CP008957.1 | 4846427 | 4847631 | 1205 | 4847285 | 1386 | 213.96571 | 2.64648 | 210.93637 | IP_DMC_vs_In_DMC_peak_459 | EDL933_RS25165:Promoter;EDL933_RS25150:Promoter;EDL933_RS25175:Promoter;EDL933_RS25160:CDS | EDL933_RS25160:NZ_CP008957.1:4847261-4847363:+:-232 | EDL933_RS25160(rhoL) |
| NZ_CP008957.1 | 4863220 | 4863644 | 425 | 4863484 | 1088 | 172.28307 | 2.68064 | 169.48882 | IP_DMC_vs_In_DMC_peak_460 | EDL933_RS25265:Promoter;EDL933_RS25270:Promoter;EDL933_RS25250:Promoter;EDL933_RS25245:Promoter;EDL933_RS25260:Promoter | EDL933_RS25240:NZ_CP008957.1:4863405-4863482:+:26 | EDL933_RS25240(EDL933_RS25240) |
| NZ_CP008957.1 | 4867476 | 4868064 | 589 | 4867745 | 1019 | 132.65642 | 2.40893 | 130.12679 | IP_DMC_vs_In_DMC_peak_461 | EDL933_RS25275:Promoter | EDL933_RS25275:NZ_CP008957.1:4865617-4867273:-:-496 | EDL933_RS25275(aslA) |
| NZ_CP008957.1 | 4919187 | 4920766 | 1580 | 4919666 | 1295 | 205.47661 | 2.68725 | 202.48094 | IP_DMC_vs_In_DMC_peak_466 | EDL933_RS25540:Promoter;EDL933_RS25545:Promoter;EDL933_RS25535:Promoter;EDL933_RS25530:exon | EDL933_RS25530:NZ_CP008957.1:4919279-4920821:+:697 | EDL933_RS25530(EDL933_RS25530) |
| NZ_CP008957.1 | 4921231 | 4924375 | 3145 | 4924268 | 2446 | 47.95794 | 1.36393 | 45.90023 | IP_DMC_vs_In_DMC_peak_467 | EDL933_RS25570:Promoter;EDL933_RS25565:Promoter;EDL933_RS25550:exon | EDL933_RS25550:NZ_CP008957.1:4924266-4924382:+:-1463 | EDL933_RS25550(rrf) |
| NZ_CP008957.1 | 4933297 | 4933758 | 462 | 4933515 | 1237 | 179.23077 | 2.55265 | 176.39552 | IP_DMC_vs_In_DMC_peak_468 | EDL933_RS25605:Promoter | EDL933_RS25600:NZ_CP008957.1:4933711-4934344:-:817 | EDL933_RS25600(EDL933_RS25600) |
| NZ_CP008957.1 | 4941577 | 4941830 | 254 | 4941702 | 600 | 10.04094 | 1.31034 | 8.47761 | IP_DMC_vs_In_DMC_peak_470 | EDL933_RS25630:Promoter;EDL933_RS25625:Promoter;EDL933_RS25640:Promoter | EDL933_RS25640:NZ_CP008957.1:4941831-4943655:+:-128 | EDL933_RS25640(typA) |
| NZ_CP008957.1 | 4979230 | 4979855 | 626 | 4979770 | 526 | 7.88963 | 1.28533 | 6.40185 | IP_DMC_vs_In_DMC_peak_473 | EDL933_RS25825:CDS;EDL933_RS25820:Promoter;EDL933_RS25815:Promoter | EDL933_RS25825:NZ_CP008957.1:4979550-4980945:+:-8 | EDL933_RS25825(EDL933_RS25825) |
| NZ_CP008957.1 | 5015875 | 5016269 | 395 | 5016020 | 616 | 18.39201 | 1.4585 | 16.63888 | IP_DMC_vs_In_DMC_peak_476 | EDL933_RS26030:Promoter;EDL933_RS26025:Promoter;EDL933_RS26020:Promoter | EDL933_RS26025:NZ_CP008957.1:5016130-5016343:+:-58 | EDL933_RS26025(rpmE) |
| NZ_CP008957.1 | 5020330 | 5021297 | 968 | 5020712 | 497 | 10.68228 | 1.36209 | 9.09913 | IP_DMC_vs_In_DMC_peak_477 | EDL933_RS26035:CDS | EDL933_RS26035:NZ_CP008957.1:5020688-5020925:+:125 | EDL933_RS26035(EDL933_RS26035) |
| NZ_CP008957.1 | 5063450 | 5065048 | 1599 | 5063757 | 1355 | 235.60168 | 2.83901 | 232.51151 | IP_DMC_vs_In_DMC_peak_480 | EDL933_RS26235:Promoter;EDL933_RS26230:exon;EDL933_RS26240:Promoter | EDL933_RS26230:NZ_CP008957.1:5063552-5065094:+:696 | EDL933_RS26230(EDL933_RS26230) |
| NZ_CP008957.1 | 5065408 | 5068551 | 3144 | 5068442 | 1913 | 30.15118 | 1.31556 | 28.24119 | IP_DMC_vs_In_DMC_peak_481 | EDL933_RS26255:Promoter;EDL933_RS26250:Promoter;EDL933_RS26245:Promoter | EDL933_RS26245:NZ_CP008957.1:5068447-5068563:+:-1468 | EDL933_RS26245(rrf) |
| NZ_CP008957.1 | 5071861 | 5072619 | 759 | 5072341 | 1061 | 134.27583 | 2.37561 | 131.73087 | IP_DMC_vs_In_DMC_peak_482 | EDL933_RS26290:Promoter;EDL933_RS26280:exon;EDL933_RS26260:Promoter;EDL933_RS26285:Promoter;EDL933_RS26295:Promoter | EDL933_RS26280:NZ_CP008957.1:5072315-5072390:+:-75 | EDL933_RS26280(EDL933_RS26280) |
| NZ_CP008957.1 | 5073790 | 5078103 | 4314 | 5076299 | 1078 | 130.34619 | 2.3231 | 127.83668 | IP_DMC_vs_In_DMC_peak_483 | EDL933_RS26315:Promoter;EDL933_RS26325:Promoter;EDL933_RS26320:Promoter | EDL933_RS26310:NZ_CP008957.1:5075521-5076226:+:425 | EDL933_RS26310(rplA) |
| NZ_CP008957.1 | 5104490 | 5106078 | 1589 | 5104821 | 1304 | 221.48874 | 2.79802 | 218.42824 | IP_DMC_vs_In_DMC_peak_488 | EDL933_RS26435:Promoter;EDL933_RS26440:Promoter;EDL933_RS26430:exon;EDL933_RS26425:Promoter | EDL933_RS26430:NZ_CP008957.1:5104578-5106120:+:705 | EDL933_RS26430(EDL933_RS26430) |
| NZ_CP008957.1 | 5106441 | 5109582 | 3142 | 5109474 | 2435 | 45.57421 | 1.35342 | 43.53255 | IP_DMC_vs_In_DMC_peak_489 | EDL933_RS26445:exon;EDL933_RS26455:Promoter | EDL933_RS26445:NZ_CP008957.1:5109473-5109589:+:-1462 | EDL933_RS26445(rrf) |
| NZ_CP008957.1 | 5129385 | 5129643 | 259 | 5129553 | 541 | 8.75813 | 1.30108 | 7.23941 | IP_DMC_vs_In_DMC_peak_491 | EDL933_RS26505:Promoter;EDL933_RS26515:Promoter;EDL933_RS26510:Promoter;EDL933_RS26520:CDS | EDL933_RS26515:NZ_CP008957.1:5128667-5129465:-:-48 | EDL933_RS26515(EDL933_RS26515) |
| NZ_CP008957.1 | 5175918 | 5176181 | 264 | 5176074 | 568 | 12.96248 | 1.3805 | 11.31753 | IP_DMC_vs_In_DMC_peak_494 | EDL933_RS26745:Promoter;EDL933_RS26755:Promoter;EDL933_RS26740:Promoter | EDL933_RS26745:NZ_CP008957.1:5176137-5176674:+:-88 | EDL933_RS26745(ssb1) |
| NZ_CP008957.1 | 5208491 | 5209087 | 597 | 5208781 | 45586 | 2689.90942 | 1.7691 | 2685.24463 | IP_DMC_vs_In_DMC_peak_496 | EDL933_RS26875:Promoter;EDL933_RS26880:CDS | EDL933_RS26880:NZ_CP008957.1:5208438-5209146:+:350 | EDL933_RS26880(EDL933_RS26880) |
| NZ_CP008957.1 | 5215014 | 5215919 | 906 | 5215275 | 1031 | 130.0657 | 2.37129 | 127.55835 | IP_DMC_vs_In_DMC_peak_497 | EDL933_RS26905:Promoter;EDL933_RS26910:Promoter;EDL933_RS26915:Promoter | EDL933_RS26915:NZ_CP008957.1:5215318-5217589:+:148 | EDL933_RS26915(EDL933_RS26915) |
| NZ_CP008957.1 | 5307131 | 5307967 | 837 | 5307394 | 693 | 38.01931 | 1.7061 | 36.03584 | IP_DMC_vs_In_DMC_peak_507 | EDL933_RS27380:Promoter;EDL933_RS27375:Promoter;EDL933_RS27370:CDS | EDL933_RS27370:NZ_CP008957.1:5307278-5308577:+:270 | EDL933_RS27370(purA) |
| NZ_CP008957.1 | 5342466 | 5343000 | 535 | 5342833 | 520 | 6.96077 | 1.26396 | 5.50954 | IP_DMC_vs_In_DMC_peak_511 | EDL933_RS27570:CDS;EDL933_RS27560:Promoter;EDL933_RS27580:Promoter | EDL933_RS27570:NZ_CP008957.1:5342035-5342974:+:697 | EDL933_RS27570(EDL933_RS27570) |
| NZ_CP008957.1 | 5526078 | 5526466 | 389 | 5526271 | 691 | 36.72436 | 1.68993 | 34.75226 | IP_DMC_vs_In_DMC_peak_527 | EDL933_RS28400:Promoter;EDL933_RS28405:Promoter | EDL933_RS28400:NZ_CP008957.1:5526316-5527540:+:-44 | EDL933_RS28400(deoB) |
| NZ_CP008958.1 | 3886 | 4971 | 1086 | 4095 | 2904 | 313.80505 | 2.21544 | 310.57523 | IP_DMC_vs_In_DMC_peak_531 | EDL933_RS28600:Promoter;EDL933_RS28595:Promoter;EDL933_RS28605:Promoter | EDL933_RS28595:NZ_CP008958.1:4809-5094:+:-381 | EDL933_RS28595(EDL933_RS28595) |
| NZ_CP008958.1 | 26236 | 26807 | 572 | 26624 | 1757 | 23.42556 | 1.28359 | 21.59567 | IP_DMC_vs_In_DMC_peak_544 | EDL933_RS28705:Promoter;EDL933_RS28700:CDS | EDL933_RS28705:NZ_CP008958.1:26673-28641:+:-152 | EDL933_RS28705(etpD) |
| NZ_CP008958.1 | 47831 | 48695 | 865 | 48438 | 2982 | 317.47681 | 2.20084 | 314.2392 | IP_DMC_vs_In_DMC_peak_551 | intergenic | EDL933_RS28810:NZ_CP008958.1:47524-48265:+:738 | EDL933_RS28810(EDL933_RS28810) |
| NZ_CP008958.1 | 49563 | 50003 | 441 | 49774 | 2825 | 271.29129 | 2.1041 | 268.13556 | IP_DMC_vs_In_DMC_peak_552 | EDL933_RS28815:Promoter | EDL933_RS28815:NZ_CP008958.1:48549-49527:-:-255 | EDL933_RS28815(EDL933_RS28815) |
| NZ_CP008958.1 | 66348 | 66840 | 493 | 66597 | 2449 | 146.38036 | 1.76869 | 143.74286 | IP_DMC_vs_In_DMC_peak_557 | EDL933_RS28940:Promoter;EDL933_RS34250:Promoter;EDL933_RS28945:Promoter;EDL933_RS28935:Promoter | EDL933_RS28935:NZ_CP008958.1:66607-66838:+:-13 | EDL933_RS28935(EDL933_RS28935) |
| NZ_CP008958.1 | 73736 | 74152 | 417 | 73942 | 21966 | 1262.84619 | 1.75434 | 1258.79907 | IP_DMC_vs_In_DMC_peak_558 | EDL933_RS28995:Promoter;EDL933_RS28990:Promoter | EDL933_RS28990:NZ_CP008958.1:73948-74107:+:-4 | EDL933_RS28990(EDL933_RS28990) |
| NZ_CP008957.1 | 542382 | 542733 | 352 | 542574 | 619 | 26.0775 | 1.58213 | 24.21221 | IP_DMC_vs_In_DMC_peak_65 | EDL933_RS02620:Promoter;EDL933_RS29160:exon;EDL933_RS02605:Promoter;EDL933_RS02615:Promoter | EDL933_RS29160:NZ_CP008957.1:542520-542617:+:37 | EDL933_RS29160(ffs) |
| NZ_CP008957.1 | 3562286 | 3563046 | 761 | 3562544 | 1157 | 200.88303 | 2.8333 | 197.91583 | IP_DMC_vs_In_DMC_peak_285 | EDL933_RS18575:Promoter;EDL933_RS18560:Promoter;EDL933_RS18565:Promoter;EDL933_RS29255:exon | EDL933_RS29255:NZ_CP008957.1:3562490-3562853:+:175 | EDL933_RS29255(ssrA) |
| NZ_CP008957.1 | 3880110 | 3881198 | 1089 | 3880812 | 1302 | 251.91565 | 3.0384 | 248.78528 | IP_DMC_vs_In_DMC_peak_326 | EDL933_RS20190:Promoter;EDL933_RS29275:exon;EDL933_RS20175:Promoter;EDL933_RS20180:Promoter | EDL933_RS29275:NZ_CP008957.1:3880737-3880921:+:-83 | EDL933_RS29275(ssrS) |
| NZ_CP008957.1 | 1641589 | 1642008 | 420 | 1641810 | 1507 | 436.39792 | 4.13544 | 433.03638 | IP_DMC_vs_In_DMC_peak_159 | EDL933_RS08375:Promoter;EDL933_RS08390:Promoter;EDL933_RS30185:Promoter | EDL933_RS30185:NZ_CP008957.1:1642006-1642369:+:-208 | EDL933_RS30185(EDL933_RS30185) |
| NZ_CP008957.1 | 2850084 | 2850622 | 539 | 2850387 | 765 | 61.87441 | 1.948 | 59.74232 | IP_DMC_vs_In_DMC_peak_207 | EDL933_RS15115:Promoter;EDL933_RS15110:Promoter;EDL933_RS15120:Promoter;EDL933_RS30885:CDS;EDL933_RS15125:Promoter | EDL933_RS30885:NZ_CP008957.1:2850368-2850419:+:-15 | EDL933_RS30885(hisL) |
| NZ_CP008957.1 | 620492 | 620769 | 278 | 620617 | 468 | 7.16548 | 1.28615 | 5.70571 | IP_DMC_vs_In_DMC_peak_71 | EDL933_RS02870:CDS;EDL933_RS32000:Promoter | EDL933_RS32000:NZ_CP008957.1:620924-621185:+:-294 | EDL933_RS32000(EDL933_RS32000) |
| NZ_CP008957.1 | 3202054 | 3202277 | 224 | 3202157 | 457 | 5.96683 | 1.25599 | 4.56132 | IP_DMC_vs_In_DMC_peak_241 | EDL933_RS16850:Promoter;EDL933_RS32635:CDS | EDL933_RS32635:NZ_CP008957.1:3201866-3202484:+:299 | EDL933_RS32635(EDL933_RS32635) |
| NZ_CP008957.1 | 4137893 | 4139110 | 1218 | 4138687 | 1234 | 189.28551 | 2.63481 | 186.38766 | IP_DMC_vs_In_DMC_peak_349 | EDL933_RS21555:Promoter;EDL933_RS32785:Promoter;EDL933_RS21560:CDS | EDL933_RS32785:NZ_CP008957.1:4137976-4138057:-:-444 | EDL933_RS32785(yrbN) |
| NZ_CP008957.1 | 2917098 | 2917672 | 575 | 2917517 | 623 | 26.9432 | 1.59316 | 25.06808 | IP_DMC_vs_In_DMC_peak_215 | EDL933_RS15405:Promoter;EDL933_RS30905:Promoter;EDL933_RS15420:Promoter;EDL933_RS33635:Promoter;EDL933_RS15410:Promoter;EDL933_RS15415:Promoter;EDL933_RS33630:Promoter | EDL933_RS33635:NZ_CP008957.1:2917420-2917477:-:92 | EDL933_RS33635(EDL933_RS33635) |
| NZ_CP008957.1 | 3949911 | 3950749 | 839 | 3950078 | 501 | 8.098 | 1.29878 | 6.60313 | IP_DMC_vs_In_DMC_peak_330 | EDL933_RS20575:Promoter;EDL933_RS20570:CDS;EDL933_RS33775:Promoter | EDL933_RS33775:NZ_CP008957.1:3950849-3950999:+:-519 | EDL933_RS33775(EDL933_RS33775) |
| NZ_CP008957.1 | 4676578 | 4676841 | 264 | 4676660 | 554 | 8.68937 | 1.29534 | 7.17341 | IP_DMC_vs_In_DMC_peak_419 | intergenic | EDL933_RS33850:NZ_CP008957.1:4676333-4676649:+:376 | EDL933_RS33850(EDL933_RS33850) |
