# Supplementary material for: Genomic Island-Encoded Histidine Kinase and Response Regulator Coordinate Mannose Utilization with Virulence in Enterohemorrhagic Escherichia coli
Source: mBio. 2023 Feb 14;14(2):e03152-22. doi: 10.1128/mbio.03152-22 (PMC10128022; doi:10.1128/mbio.03152-22)
Supplement: TABLE S1 [file mbio.03152-22-s0001.docx]

| **Table S1. Bacterial strains and plasmids used in this study** | | |
| --- | --- | --- |
| **Strain or plasmid** | **Description** | **Source or reference** |
| Bacterial strains |  |  |
| DH5α | Cloning strain | Invitrogen |
| BL21(DE3) | Protein expression strain | Vazyme |
| EDL933(wild type) | EHEC isolate associated with outbreak | (1) |
| Δ*lmvR* | EDL933 background with *lmvR* deleted | This study |
| Δ*lmuK* | EDL933 background with *lmuK* deleted | This study |
| Δ*lumA* | EDL933 background with *lumA* deleted | This study |
| Δ*lumI* | EDL933 background with *lumI* deleted | This study |
| Δ*lmuXYZ* | EDL933 background with *lmuXYZ* deleted | This study |
| Δ*lmvK* | EDL933 background with *lmvK* deleted | This study |
| Δ*lmuKAIZYX* | EDL933 background with *lmuKAIZYX* deleted | This study |
| EDL933str | Spontaneous streptomycin resistant variant of EDL933 | This study |
| EDL933strΔ*lacZ* | EDL933str background with *lacZ* deleted, used in animal test (designated WT) | This study |
| EDL933strΔ*lmvR* | EDL933str background *lmvR* deleted, lacZ+ | This study |
| EDL933strΔ*lmvK* | EDL933str background *lmvK* deleted, lacZ+ | This study |
| EDL933strΔ*lmuKAIZYX* | EDL933str background *lmuKAIZYX* deleted, lacZ+ | This study |
| Δ*manA* | EDL933 background with *manA* deleted | This study |
| EDL933strΔlacZΔ*manA* | EDL933strΔlacZ background with *manA* deleted, used in animal test | This study |
| Δ*manA*Δ*lmuKAIZYX* | Δ*manA* background with *lmuKAIZYX* deleted | This study |
| EDL933strΔ*manA*Δ*lmuKAIZYX* | EDL933str background with *ΔmanAΔlmuKAIZYX*  deleted, , lacZ+, used in animal test | This study |
| Δ*manA*+pLmvR | Δ*manA* background carrying pLmvR plasmid | This study |
| Δ*manA*Δ*lmuKAIZYX*+pLmvR | Δ*manA*Δ*lmuKAIZYX* background carrying pLmvR plasmid | This study |
| Δ*ler* | EDL933 background with *ler* deleted | This study |
| ΔlmvR+pLmvR | ΔlmvR background carrying pLmvR plasmid, complemented strain | This study |
| ΔlmvK+pLmvK | ΔlmvK background carrying pLmvK plasmid, complemented strain | This study |
| WT-pLmvR | EDL933 background carrying pLmvR plasmid | This study |
| ΔlmvR+pLmvR-2Flag | ΔlmvR background carrying pLmvR-2Flag plasmid | This study |
| Plasmids |  |  |
| pET28a | Protein expression vector | Novagen |
| pGEN-Ptac | pGEN-MCS carrying *lacI*-Ptac from pMal-c2x | This study |
| pCJ112 | Vector carrying promoterless *lacZ* for reporter fusion assays | (2) |
| pLmvR | *lmvR* driven by Ptac on pGEN-Ptac | This study |
| pLmvK | *lmvK* driven by its native promoter on pCC1-BAC | This study |
| pLmvRD54Q | pLmvR with D54 mutated to Q | This study |
| pLmvKH410A | pLmvK with H410 mutated to A | This study |
| pLmvR-2Flag | Flag-tagged LmvR driven by Ptac on pGEN-Ptac used for ChIP-Seq | This study |
|  |  |  |
|  |  |  |
| (1) Riley LW, Remis RS, Helgerson SD, McGee HB, Wells JG, et al. (1983) Hemorrhagic colitis associated with a rare Escherichia coli serotype. N Engl J Med 308: 681-685. | | |
| (2) Cai W, Cai X, Yang Y, Yan S, Zhang H. (2017)Transcriptional Control of Dual Transporters Involved in alpha-Ketoglutarate Utilization Reveals Their Distinct Roles in Uropathogenic Escherichia coli. Front Microbiol. 8:275. | | |
