# Supplementary material for: Genomic Island-Encoded Histidine Kinase and Response Regulator Coordinate Mannose Utilization with Virulence in Enterohemorrhagic Escherichia coli
Source: mBio. 2023 Feb 14;14(2):e03152-22. doi: 10.1128/mbio.03152-22 (PMC10128022; doi:10.1128/mbio.03152-22)
Supplement: TABLE S2 [file mbio.03152-22-s0002.docx]

| **Table S2. Oligonucleotides used in this study.** | | |
| --- | --- | --- |
| **Assay** | **Primer** | **Sequence (5'-3')** |
| **qRT-PCR** | | |
|  | 5428-qPCR-F | CTGGCGGTTAATGACGAGAA |
|  | 5428-qPCR-R | CATGTTGCTGAAGCGGATTG |
|  | 5429-qPCR-F | GGATATGCCGGACGATGTTTA |
|  | 5429-qPCR-R | AGGTCGAGTGAGGTTTCAAAG |
|  | 5430-qPCR-F | TGGGATTCAGCTCGGTAATG |
|  | 5430-qPCR-R | CCAGTTCAGCTTCTACCGATAC |
|  | 5431-qPCR-F | GACGACCACGATAACCACTTT |
|  | 5431-qPCR-R | GAACTGGCGGTATTCGTTACA |
|  | 5432-qPCR-F | TGCTCTCTGACAACCAGAAC |
|  | 5432-qPCR-R | GTGGGATCGTTAATCGCAAATAC |
|  | 5433-qPCR-F | CTGGTGCTGCTGTTTGTTTATG |
|  | 5433-qPCR-R | GTTCGTAACCCATGCCTAAGT |
|  | 5434-qPCR-F | ATTCTGGTGATGGACGAACC |
|  | 5434-qPCR-R | CATGCGGTGGCTAATGTAGATA |
|  | rpoB-qPCR-F | GCATCATCCCTTACCGTGGTTC |
|  | rpoB-qPCR-R | GGATCTGCTCTGTGGTGTAGTTCA |
|  | ler-qPCR-F | CGAGAGCAGGAAGTTCAAAGT |
|  | ler-qPCR-R | AGTCCATCATCAGGCACATTAG |
|  | escC-qPCR-F | CTATGCGTCAGCGACAGATATAA |
|  | escC-qPCR-R | GGTACTGGGAAGTGAACCATT |
|  | cesT-qPCR-F | GTAGCATCATCGAGAGGGAAAC |
|  | cesT-qPCR-R | GCAAACTTATGGTTTGCAGAGAA |
|  | tir-qPCR-F | GAGGGAGTCAAATAGCGGTG |
|  | tir-qPCR-R | ATCTGAACGAAGGCTGGAAG |
|  | espA-qPCR-F | TCACTACCGTTGTCAGGTTATTC |
|  | espA-qPCR-R | AGTGTAACTGGTATTCGTGATCTT |
|  | espB-qPCR-F | TGCTGTGGTATTGGTCAGTTTA |
|  | espB-qPCR-R | GCCTCTGACTTTGCTGAAGA |
|  | eae-qPCR-F | TACGACACTGCCTTTACCATTC |
|  | eae-qPCR-R | GCGGTGGTGATGGTACATATT |
|  | escN-qPCR-F | GCTGAACCACCAGATCCTTTAT |
|  | escN-qPCR-R | GCCCAATACCGCAAGTTAGTA |
| **Co-transcription assay** |  |  |
| P2 | F1-5429-5431-F | GCGGCGGCGGTAATTTAATT |
| P1 | R1-5429-5431-R | CCGCTTGGGTCTGTCATCAC |
| P4 | F2-5430-5432-F | AAGATCGACGGCGTATTTGC |
| P3 | R2-5430-5432-R | CCAATCGCCACGGCCA |
| P6 | F3-5431-5433-R | GCTGGTGCTGCTGTTTGT |
| P5 | R3-5431-5433-R | AAAACCTCCTCCCACGC |
| P8 | F4-5432-5434-F | AACATTCGCGTGCCTCAT |
| P7 | R4-5432-5434-R | AAGTTGTCTATCTGCGACAC |
| **Gene deletion primers** |  |  |
| *del-EDL5428* | edl5428del-F | GATTAATCAGATGAATGATGCGGAGTGTAACCATGAAACCCGTCGTGCTGGTGTAGGCTGGAGCTGCTTCGA |
|  | edl5428del-R | CTGCAAACAGAAATGTCGGATTAATGCACGTCGGCAGCGAAGACATACCCCATATGAATATCCTCCTTAG |
|  | CK-5428-F | TGCGTGATACTGGCTTTGAAAC |
|  | CK-5428-R | ACGCTGGGCATCGTCAAAATCC |
| *del-EDL5429* |  |  |
|  | del-5429A-F | CTTGCATGCCTGCAGGTCGACCCGAAACTCGATTTCCCGC |
|  | del-5429A-R | TCCAGCCTACACTCATGCCTGCCCCGCACT |
|  | del-5429B-F | AGGCATGAGTGTAGGCTGGAGCTGCTTCG |
|  | del-5429B-R | CTGCCGACGTGCATTAACATATGAATATCCTCCTTAGTTCCTATTC |
|  | del-5429C-F | ATGTTAATGCACGTCGGCAGCG |
|  | del-5429C-R | AAAACGACGGCCAGTGAATTCTTCAATGGCTGGTATCTCGATACC |
|  | CK-5429-F | TACCGGCATTGACGCG |
|  | CK-5429-R | CTGAAAGCGGTGTTGCG |
| *del-EDL5430* |  |  |
|  | del-5430A-F | CTTGCATGCCTGCAGGTCGACGGCTGATAGTGACATTACGGTGG |
|  | del-5430A-R | CCAGCCTACACAACGTTATCCTTATTCAGCGGAA |
|  | del-5430B-F | GATAACGTTGTGTAGGCTGGAGCTGCTTCG |
|  | del-5430B-R | CCCTTTACGCTCCATCATCATATGAATATCCTCCTTAGTTCCTATTC |
|  | del-5430C-F | TGATGATGGAGCGTAAAGGGATTATC |
|  | del-5430C-R | AAAACGACGGCCAGTGAATTCCGACATCGACGTCGGGGC |
|  | CK-5430-F | TCAATCGCGGCGGCG |
|  | CK-5430-R | ACGCCGGAAGATCCAAAAG |
| *del-EDL5431* |  |  |
|  | del-5431-1 | CAGGTCGACTCTAGAGGATCCTGTCGCTACTCCGGCGCA |
|  | del-5431-2 | GCTGCAATCTCGAAAGCGTTCTCCTTAGCG |
|  | del-5431-3 | AACGCTTTCGAGATTGCAGCATTACACGTCTTGA |
|  | del-5431-4 | TTCGTGGCTGACATGGGAATTAGCCA |
|  | del-5431-5 | ATTCCCATGTCAGCCACGAACCTGCACAGTTGGTATTG |
|  | del-5431-6 | TCTGATATCATCGATGAATTCAGCGAGATATATTTAAAATGTACTTCGG |
|  | del-5431-KUO-F | TGTCGCTACTCCGGCGCA |
|  | del-5431-KUO-R | AGCGAGATATATTTAAAATGTACTTCGG |
| *del-EDL5432-5434* |  |  |
|  | del32-34-1F | CAGGTCGACTCTAGAGGATCCGCCACGCCGCTGACCATC |
|  | del32-34-1R | CAGCCTACACACCGTCAAATCCACCCCTTT |
|  | del32-34-2F | ATTTGACGGTGTGTAGGCTGGAGCTGCTTCG |
|  | del32-34-2R | TCGGCGCAGGCATATGAATATCCTCCTTAGTTCCTATTC |
|  | del32-34-3F | TATTCATATGCCTGCGCCGAAAGAGCCG |
|  | del32-34-3R | TCTGATATCATCGATGAATTCGCGGTAACATCCCAGCCG |
|  | DEL-KUO32-34F | GCCACGCCGCTGACCATC |
|  | DEL-KUO32-34R | GCGGTAACATCCCAGCCG |
| *del-EDL5436* |  |  |
|  | edl5436-del-F | TATTTGTCATATTGCGATTTTTCTTCACGGAGCGTGATATGGCGCACCCCGTGTAGGCTGGAGCTGCTTCGA |
|  | edl5436-del-R | CAAGGTAGGCAAAGGTGCCTGTTACGAGTAGAGGAGGATTCTCAATTCTCCATATGAATATCCTCCTTAG |
| *del-ler* |  |  |
|  | Ler-A-F | CAGGTCGACTCTAGAGGATCCTAATAGATATATATACTCGTCATACTTCAAGTTGC |
|  | Ler-A-R | GGAGGATATTCATATGGCTTTAATATTTTAAGCTATTAGCGACC |
|  | Ler-B-F | AAGCCATATGAATATCCTCCTTAGTTCCTATTC |
|  | Ler-B-R | TTTCATGGTGTAGGCTGGAGCTGCTTCG |
|  | Ler-C-F | CTCCAGCCTACACCATGAAATAATTAAATGATAACGATAACTGA |
|  | Ler-C-R | TCTGATATCATCGATGAATTCTGCTGGACTCAGTGTCTCTATTAGTTT |
|  | CK-Ler-F | CGCGGTTACTGTTCAGC |
|  | CK-Ler-R | CCGTTGATTCATTGTTGATTGG |
|  | Ler-KUO-F | GATATATATACTCGTCATACTTCAAGTTG |
|  | Ler-KUO-R | TGCTGGACTCAGTGTCTC |
| *del-manA* |  |  |
|  | CK-manA-del-F | GAAAGGGTTTGTTTGACATTG |
|  | CK-manA-del-R | TTGCCTGTATACCATGCGC |
|  | manA-del-F | TAGGATTCTTGCTTTAATAGCGGGATTAATTTCCACATTAAAACAGGGATTGATCGTGTAGGCTGGAGCTGCTTCGA |
|  | manA-del-R | GTGTTATAAGCCTTTAATAAGCTTAGCAAGAGATGTTAATTTTTTCAGTAAGCTCCATATGAATATCCTCCTTAG |
| *del-lacZ* |  |  |
|  | 933lacZ-A-F | CTTGCATGCCTGCAGGTCGACCCGCTTGCTGCAACTCTCTC |
|  | 933lacZ-A-R | TCCAGCCTACACAGCTGTATCCTGTGTGAAATTGTTATC |
|  | 933lacZ-B-F | ATACAGCTGTGTAGGCTGGAGCTGCTTCG |
|  | 933lacZ-B-R | GCCTGCCCGGTTATTACATATGAATATCCTCCTTAGTTCCTATTC |
|  | 933lacZ-C-F | TATGTAATAACCGGGCAGGCCAT |
|  | 933lacZ-C-R | AAAACGACGGCCAGTGAATTCCATGCCGGTAATAATCCACAGC |
|  | KUO-933lacZ-F | CCGCTTGCTGCAACTCT |
|  | KUO-933lacZ-R | CATGCCGGTAATAATCCACAGC |
|  | CK-933lacZ-F | TCGGTAGTGGGATACGACGA |
|  | CK-933lacZ-R | CCACCAACAATCGATCCTACTA |
| **ChIP-seq** |  |  |
|  | Ptac2Flg5428-Fn | AACAAGGACCATAGCATATGGACTACAAAGACCATGACGGTGATTATAAAGATCATGACATCAAACCCGTCGTGCTGGTG |
|  | Ptac2Flg5428-Rn | TGGGGCCCGCGGCCGCGGATCCAGAGCTGCAAACAGAAATGTC |
|  | Ptac2Flg5428-Fc | AACAAGGACCATAGCATATGAAACCCGTCGTGCTGGTG |
|  | Ptac2Flg5428-Rc | TGGGGCCCGCGGCCGCGGATCCTTAGATGTCATGATCTTTATAATCACCGTCATGGTCTTTGTAGTCATGCACGTCGGCAGCGAA |
| **Complementation** |  |  |
|  | pEDL5428-F | TTCACCAACAAGGACCATAGCATATGAAACCCGTCGTGCTGGTG |
|  | pEDL5428-R | TCCATGGGGCCCGCGGCCGCGGATCCTTAATGCACGTCGGCAGCGA |
|  | pCC1-5436-F | AAAACGACGGCCAGTGAATTCCTTGCCGTCTATGAGAATC |
|  | pCC1-5436-R | GACCATGATTACGCCGGATCCCAAAGTTAATACAAGGTAGGC |
| **5'-RACE assay** |  |  |
|  | 5434RACE-1st-R | TTGCGATGTAGTGCGCGGGCGATTTCGA |
|  | 5434RACE-2nd-R | CTGTTGCTCGGCAATGGTCAGCGTCATG |
| **EMSA** |  |  |
| *EDL5434*-EMSA | EMSA-5434-F | GACGAAAGCCCATTACCG |
|  | EMSA-5434-R | GCTGTTCATGGTGGACTC |
| *ler-*EMSA | EMSA-ler2-F | GTGTAAAATACATTATCATTAAATGTC |
|  | EMSA-ler2-R | ATTCACTCGCTTGCCGCC |
| *gadW-*EMSA | gadW-EMSA-F | GATTATCCCTTATATTTCATACTGCGA |
|  | gadW-EMSA-R | AAATATAACTTTTACTGGAAATAAGATCAGC |
| *garD-*EMSA | garD-EMSA-F | ATGCTAATTTAATTAATACTATTTAAATATTATTTTGAGCAT |
|  | garD-EMSA-R | TGTGCAATATTCTCCAGCCAG |
| *OXIDO-*EMSA | OXIDO-EMSA-F | GGTTATTTACACCTTAGCGCAA |
|  | OXIDO-EMSA-R | GGGGTAAATGTCCCTTTCAAC |
| *qseE-*EMSA | qseE-EMSA-F | GGTGTTACTCTCGTCAGACG |
|  | qseE-EMSA-R | TGGCTCATTCACCGACTT |
| *ripM-*EMSA | ripM-EMSA-F | AAGCCCACCCCCAAGACA |
|  | ripM-EMSA-R | AAGGTCGTCGGTTCAAATCC |
| *bla*-negative control | emsa-bla-F | CGGGCTTGTCTGCTCC |
|  | emsa-bla-R | ACTCTTCCTTTTTCAATATTATTGAAGCAT |
| *EDL5428-*EMSA | 5428EMSA-F | AGCGGTATTTACCATCATGCCG |
|  | 5428EMSA-R | CCTAATAAAATGGATCGTTCCGC |
| **LacZ activity assay** |  |  |
|  | P5428-lacZ-F | CCGGTACCAGCATGCGAATTCtcctgacgatcgctaaacg |
|  | P5428-lacZ-R | GTTTCCTGTCAGTCATCTAGAgatagccgtgtcatcatcca |
|  | P1P2-lacZ-F | GGCGCCTTTTATGGAGAATTCATGCACCCGTTCCAGG |
|  | P1-lacZ-R | GTTTCCTGTCAGTCATCTAGACCACCTTAAAATGTTATTATTCTC |
|  | P2-lacZ-F | GGCGCCTTTTATGGAGAATTCATTTAATGATAATGTATTTTACACATTAG |
|  | P1P2-lacZ-R | GTTTCCTGTCAGTCATCTAGAAATAAATAATCTCCGCATGCTTTA |
